# Structural Conservation and Functional Diversity of the Poxvirus Immune Evasion (PIE) Domain Superfamily

**DOI:** 10.3390/v7092848

**Published:** 2015-08-28

**Authors:** Christopher A. Nelson, Megan L. Epperson, Sukrit Singh, Jabari I. Elliott, Daved H. Fremont

**Affiliations:** 1 Department of Pathology and Immunology, Washington University School of Medicine, St. Louis, MO 63110, USA; mepperson@path.wustl.edu (M.L.E.); jieliott@wustl.edu (J.I.E.); 2 Department of Biochemistry and Molecular Biophysics, Washington University School of Medicine, St. Louis, MO 63110, USA; sukrit.singh@wustl.edu; 3 Department of Molecular Microbiology, Washington University School of Medicine, St. Louis, MO 63110, USA

**Keywords:** poxvirus, PIE domain, SECRET domain, viral immune evasion, chemokine and cytokine decoy receptors

## Abstract

Poxviruses encode a broad array of proteins that serve to undermine host immune defenses. Structural analysis of four of these seemingly unrelated proteins revealed the recurrent use of a conserved beta-sandwich fold that has not been observed in any eukaryotic or prokaryotic protein. Herein we propose to call this unique structural scaffolding the PIE (Poxvirus Immune Evasion) domain. PIE domain containing proteins are abundant in chordopoxvirinae, with our analysis identifying 20 likely PIE subfamilies among 33 representative genomes spanning 7 genera. For example, cowpox strain Brighton Red appears to encode 10 different PIEs: vCCI, A41, C8, M2, T4 (CPVX203), and the SECRET proteins CrmB, CrmD, SCP-1, SCP-2, and SCP-3. Characterized PIE proteins all appear to be nonessential for virus replication, and all contain signal peptides for targeting to the secretory pathway. The PIE subfamilies differ primarily in the number, size, and location of structural embellishments to the beta-sandwich core that confer unique functional specificities. Reported ligands include chemokines, GM-CSF, IL-2, MHC class I, and glycosaminoglycans. We expect that the list of ligands and receptors engaged by the PIE domain will grow as we come to better understand how this versatile structural architecture can be tailored to manipulate host responses to infection.

## 1. Introduction

Poxviridae comprise a diverse family of large double-stranded DNA viruses that undergo replication exclusively in the host–cell cytoplasm. Poxvirus virions are easily identified by their characteristic brick-shaped appearance in electron micrographs. Each virion contains a single linear genome that varies in length (130–360 Kb) depending on the virus strain [1]. The genomes are compact, with open reading frames (ORFs) being closely spaced and non-overlapping with no evidence of mRNA splicing. Although individual strains may contain more than 200 ORFs [1], only ~50 are thought to encode proteins essential for viral transcription, DNA replication, or the formation of new virions [2]. These ORFs cluster in the central region of the genome and are well conserved in sequence and position across different species. The remaining ORFs are more variable and tend to be distributed more towards the terminal ends of each genome [3,4]. These ORFs likely encode factors that confer virulence, tissue tropism, or serve to expand host range [5]. Ample evidence suggests that poxviruses have captured host genes during their evolution in order to evade immune detection and elimination (reviewed in [6,7]). Yet it is clear from evolutionary studies comparing the sequences of ORFs across different genomes that poxviruses also adapt to changes in host defense by altering their existing repertoire of factors [8,9]. Possible mechanisms to explain the observed alterations include accumulation of point mutations, the occurrence of unequal crossovers giving rise to chimeric factors [10], or transient genomic expansions that increase the number of targets available for mutation [11]. In support of the idea that poxvirus genomes are modified in response to evolutionary pressure, several poxvirus families show signs of ORF duplication and divergence. These include: the ankyrin-repeat proteins [12], the serpin family [13], the C7L family [14], the kelch-like proteins [15], and the Bcl-2-like proteins [16,17]. From a structural point of view, each of these families can be thought of as sharing an easily identified fold. Within each family, individual members likely derive from an ancestral factor that was used as a common structural scaffold and modified repeatedly to create different binding specificities for host molecules. These modifications were presumably driven by host-mediated selective pressure.

Structural studies have revealed that, despite very low sequence similarity, several seemingly unrelated poxviral proteins adopt a characteristic β-sandwich fold, a fold with no known resemblance to any eukaryotic or prokaryotic protein. This domain was first seen in the structure of the soluble secreted viral chemokine inhibitor from cowpox (vCCI) [18] and its orthologues from mousepox (EVM1) [19] and rabbitpox (T1) [20]. Chemokines (chemotactic cytokines) are a class of soluble inflammatory mediators that control the migration of leukocytes. At sites of injury or infection, chemokines form gradients by binding to cell surface or extracellular matrix glycosaminoglycans (GAGs) and so provide directional cues for receptor-bearing cells. Not surprisingly, poxviruses have evolved proteins to disrupt chemokine networks (reviewed in [21]). The structure of a second chemokine binding protein, A41 from vaccinia, was subsequently found to have a β-sandwich fold very similar to that of vCCI, the primary differences being in the size of some surface loops and the distribution of surface electrostatic charge [22]. From *de novo* modeling, the variola virus CrmB C-terminal domain was proposed to be similar to the vCCI and A41 families [23]. Because the C-terminal domain of CrmB binds chemokine, it was given the name smallpox virus-encoded chemokine receptor (SECRET) [24]. Poxviruses appear to encode a family of SECRET domain containing chemokine inhibitors, which, in addition to CrmB, includes CrmD, SCP-1, SCP-2 and SCP-3. SECRET domain containing proteins bind chemokine either independently (SCP-1, SCP-2, SCP-3) or when fused with viral tumor necrosis factor receptors (CrmB and CrmD). The structure of the CrmD SECRET domain was demonstrated to bear a striking resemblance to vCCI and A41 [25]. More recent crystallographic studies revealed that cowpox virus protein CPXV203, a member of the T4 family, also adopts a β-sandwich fold similar to poxvirus chemokine binding proteins. CPXV203 does not bind chemokine, but instead blocks MHC class I surface expression by exploiting the KDEL-receptor recycling pathway of the ER/Golgi network [26,27,28]. CPXV203 has been shown to directly bind a wide array of both mouse and human classical and non-classical MHC class I proteins [28].

In each of the cases described above, poxviruses use the shared β-sandwich domain for immune evasion. Because only a subset of these bind chemokine, we searched for a general domain name that would better represent the entire group. For this reason, we here propose calling it the poxvirus immune evasion (PIE) domain. The sorting of proteins by domain is an especially strong tool for organizing poxviral proteins, in which structure is often more highly conserved than sequence or function. It allows biophysical characteristics to be compared across protein families in order to identify features that differ and are therefore likely to confer unique ligand-binding specificities. It may also reveal inherited features that perform a conserved function. Because of the importance of the PIE domains in host immune response modulation, we set out to search for additional members of this family within the published genomes of poxviruses, especially among ORFs of still unknown function. We employed bioinformatics tools and an analysis of the published literature. We examined 33 representative chordopoxvirus genomes to find putative PIE-domain-containing proteins. These potential PIE proteins are extremely sequence diverse, dividing into 20 separate families across seven genera. All appear to contain a β-sandwich core domain, but each family is decorated by a unique set of insertions that encode secondary structural elements. Originally identified as chemokine binding proteins, it is clear the members of the PIE family are functionally diverse, and this diversity is likely to grow as more roles for these proteins are revealed. Finally, we explore the origins of the PIE domain by examining the distribution of PIE sequences across the chordopoxvirus subfamily.

## 2. PIE Domains of Known Structure

### 2.1. vCCI

Probably the most extensively studied member of the poxvirus PIE domain family is vCCI, a protein secreted from infected cells by nearly all orthopoxviruses and leporipoxviruses. Members of this family have been given different names depending on their species of origin (vCCI, EVM1, T1, 35 kDa, vCKBP, or CBP-II). The presence of the vCCI protein in infected cell supernatants was noted long before its function was identified [29]. The vCCI protein binds chemokines in solution, preventing them from reaching their cognate receptors on target cells, and so interferes with their capacity to establish leukocyte migration. It appears to act as a competitive inhibitor of chemokine function, binding the same determinants used to engage cellular chemokine receptors [30,31,32]. The chemokine superfamily can be divided into subfamilies (C, CC, CXC, and CX_3_C) based on the spacing of conserved N-terminal cysteine residues in each cytokine [33,34]. Members of the vCCI family generally bind with high affinity to most human and mouse CC-chemokines, but not C-, CXC-, or CX_3_C-chemokines [19,32,35,36,37,38]. Consistent with their binding capacity, members of the vCCI family have been shown to block CC-chemokine-induced calcium flux and cell migration *in vitro* [32,37,39], and cell migration *in vivo* [32,39,40,41,42,43,44]. This combination of potency and specificity has drawn considerable interest for their potential use as anti-inflammatory agents [43,45,46].

**Figure 1:**
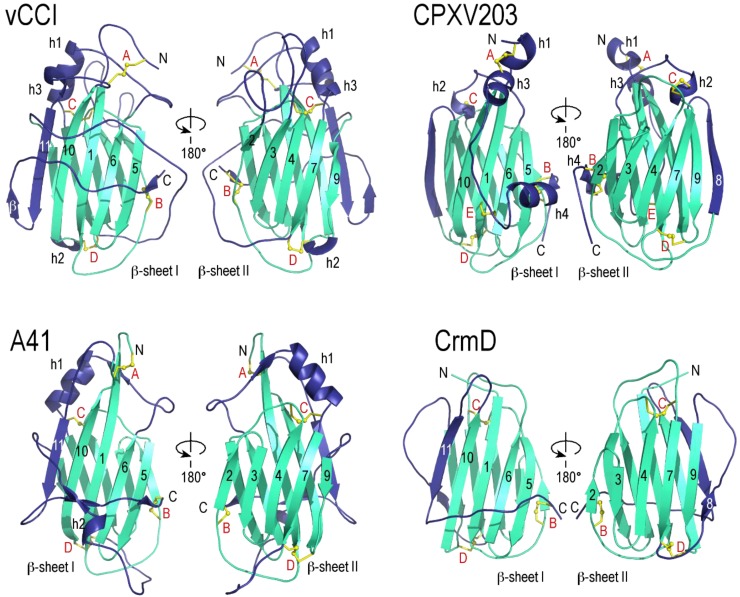
The poxvirus immune evasion (PIE) domain structures adopt a strikingly similar fold despite considerable sequence diversity. Strands in the β-sandwich core domain (cyan) are numbered the same for all four structures to aid comparison. The decorations unique to each structure (dark blue) are labeled (h for helix, β for strand). CPXV203 does not contain a strand β11 in sheet I. Similarly, strand β8 of sheet II is absent from vCCI and A41. The disulfide bonds are labeled in red (**A**–**E**). All ribbon diagrams are shown in the same orientation and at the same scale. Structures displayed include: rabbitpox vCCI (2FFK) [20], cowpox CPXV203 (4HKJ) [28], vaccinia A41(2VGA) [22], and ectromelia CrmD C-terminal SECRET domain (3ON9) [25]. Figure made in PyMol [47].

The structure of vCCI has now been determined for three different species: cowpox (alone, pdb code 1CQ3) [18], ectromelia (alone, 2GRK) [19], and rabbitpox (with MIP-1β/hCCL4, 2FFK) [20]. Each vCCI structure shares the characteristic PIE domain with very similar decorations. RMSDs between the three structures, after removing loops that adopt different conformations, are remarkably close, ranging from 0.45 Å to 1.33 Å. As shown in Figure 1, the core structure of the PIE domain consists of a compact globular β-sandwich formed from two nearly parallel β-sheets connected by loops that frequently contain short β-strands and α-helices.

**Figure 2:**
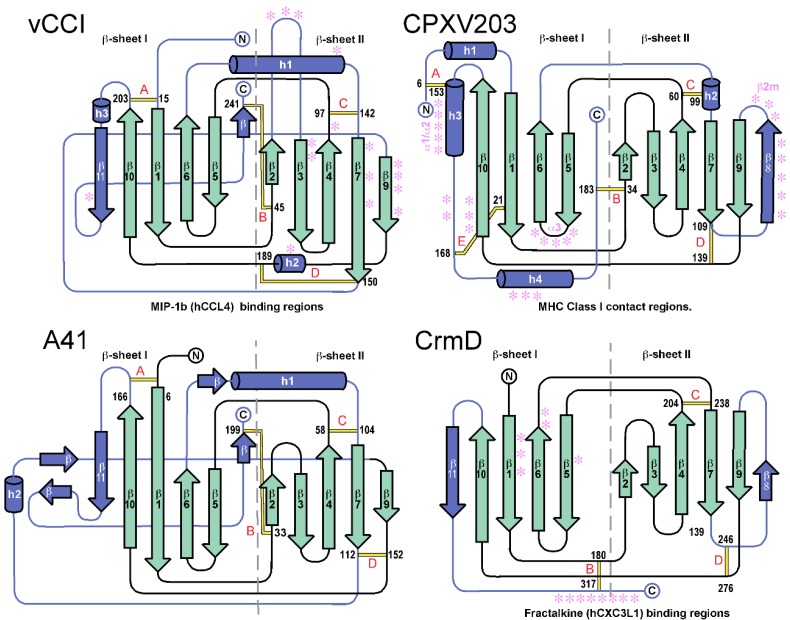
PIE domain connectivity diagrams highlighting the conserved core β-sheet architecture (cyan) and unique connecting decorations (dark blue). The disulfide bonds are labeled in red (A–E). Ligand contact regions are annotated with magenta stars for those PIE domains with structurally defined interactions.

For vCCI, β-sheet I consists of five large anti-parallel strands, here numbered β5, β6, β1, β10 and β11 (Figure 1). Likewise β-sheet II also consists of five strands, numbered β2, β3, β4, β7, and β9. β-sheet II divides along strands β7 and β9, which are the only two core strands that run parallel to each other. Four highly conserved disulfide bonds hold the β-sheets of vCCI together. These disulfides are labeled “A” through “D” in the connectivity diagram (see Figure 2). The A disulfide connects the N-terminus of the protein to the end of strand β10, the B disulfide connects the C-terminus to the start of strand β2, while C and D occur at the beginning and end of strand β7, respectively.

**Figure 3:**
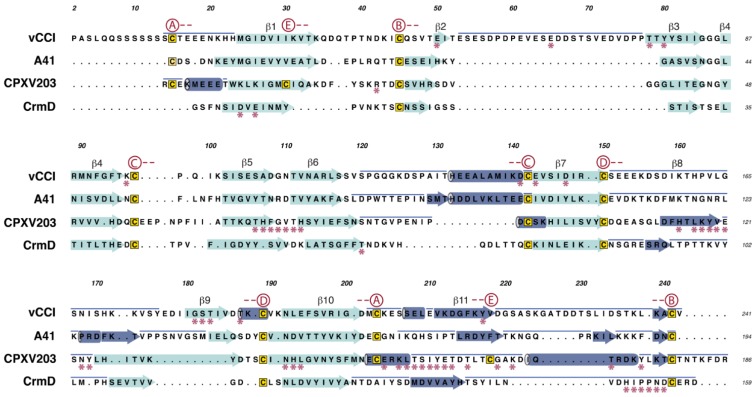
Structure based sequence alignment for the PIE domains of Figure 1. Numbering of the core β strands (cyan) is given above the sequences. The decorations are indicated (dark blue), with new strands as arrows, helices as cylinders, and extended coil as a blue line above the sequence. The decorations occur as insertions primarily to the β6–β7 loop, the β7–β9 loop, and at the C-terminus. Disulfide bond cysteines are marked above the alignment, with red circles containing letters of the different disulfide-bond pairs (A, B, C, D, or E) as indicated in Figure 1 and Figure 2. The contacts made by ligand are marked with stars (magenta) under each sequence.

**Figure 4:**
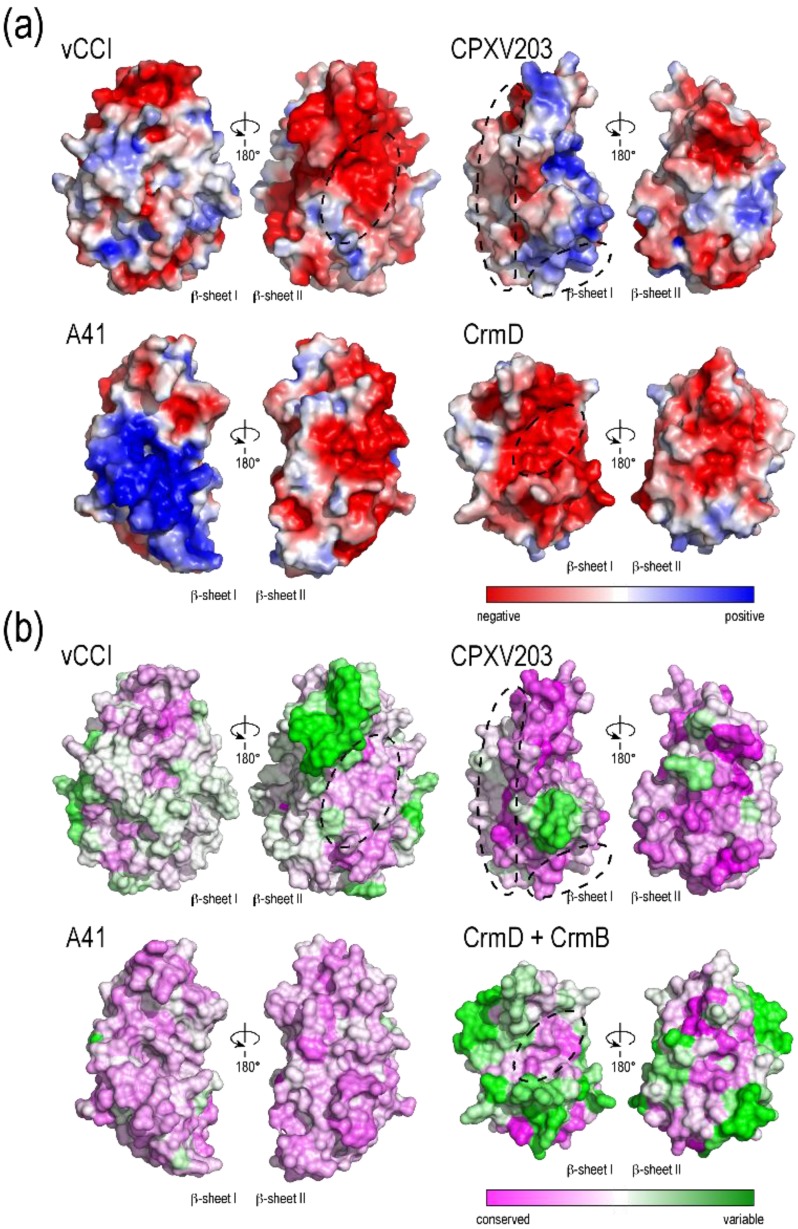
Comparison of PIE domain surface properties. The molecules and orientations are the same as in Figure 1. (**a**) Electrostatic potential surfaces calculated using APBS [52]. Negative charge in red and positive charge in blue from −3kT/e to +3kT/e. Crystallographically observed contact surfaces for ligand are circled; (**b**) Sequence conservation within individual families was mapped to the molecular surface and colored magenta for highly conserved and green for variable. Because so few CrmD exist and CrmB and CrmD are closely related, sequences for CrmB and CrmD SECRET domains were aligned and conservation mapped to the CrmD molecular surface.

The β6–β7 loop crosses between sheets I and II and contains a large α-helix. The face of vCCI β-sheet I is largely solvent inaccessible due to two large loops; the first being (β7–β9) and second being the C-terminus, which wrap around and occlude that side of the molecule. One of the most unique features of vCCI is the prominently extended β2–β3 loop that projects from β-sheet II. Both the length and sequence of this loop vary greatly between species (Figure 3 and Supplementary Figure S1), but the loop is always highly acidic, with ~50% of the residues being Glu or Asp. This loop is adjacent to a highly sequence-conserved patch of acidic residues on the solvent-exposed face of β-sheet II (Figure 4a,b). Structure-based mutational analysis was used to demonstrate that conserved residues within this patch are important to support high-affinity chemokine binding [19].

The specific molecular details of ligand binding were revealed by the NMR solution studies of Zhang *et al.* [20], who determined the structure of rabbitpox vCCI bound to human MIP-1β (hCCL4). Consistent with previous studies [18,19,20,30], the vCCI/chemokine complex formed with 1:1 stoichiometry. As predicted, the large negatively-charged sequence-conserved patch on β sheet II was found to accommodate the positively-charged chemokine (dashed oval in Figure 4). The binding by vCCI covers regions of the chemokine that are important for homodimerization, receptor binding, and GAG interactions. The negative charge and flexibility of the extended β2–β3 loop appear to allow it to interact well with different chemokines, favoring those with positive residues at certain positions, yet not excluding chemokines if large hydrophobics occupy those positions. The chemokine-binding profile of different vCCI proteins appears species independent [37]. One noteworthy difference is that lepopripovirus myoxma virus CC-chemokine inhibitor, M-T1, has been shown to interact with GAGs [48] using clusters of basic amino acid residues on the face opposite from the chemokine binding site. These basic residues are not found in orthopoxvirus versions of vCCI.

### 2.2. A41

Like vCCI, A41 is secreted from infected cells [49]. It was noted early on that, although the two proteins share little sequence identity (~22% over the entire sequence in vaccinia virus strain Lister), they are of similar size and all eight cysteines of A41 align with those of vCCI. A vaccinia mutant lacking the A41 ORF replicates normally in cell culture, yet in two different models of dermal infection it displayed an altered inflammatory response. The average lesion size for mice infected with the knockout virus was larger, and the influx of inflammatory cells greater, than for the wild-type or revertent control viruses [49]. Deletion of the A41 ORF both enhances vaccinia virus immunogenicity and increases its efficacy when used as a vaccine to immunize mice [50]. Vaccinia A41 binds a subset of CC-chemokines: CCL21, CCL25, CCL26, and CCL28. However, even the tightest of these bind with two orders of magnitude lower affinity than does vCCI to a wide range of CC-chemokines. Further, vaccinia A41 does not inhibit chemokine receptor binding, but instead blocks the GAG-binding domain on chemokines [22]. Addition of GAGs such as heparin or dextran at high concentration can disrupt the A41-chemokine interaction. Recombinant chemokine analogs with alterations in their GAG-binding domain fail to bind ectromelia encoded A41 (E163) [51]. In addition to the CC-chemokines bound by A41, ectromelia E163 has been shown to bind a limited set of CXC chemokines with high affinity and also to bind GAGs directly [51]. The current working model is that A41 prevents the establishment of the chemokine concentration gradients required for leukocyte migration, employing a mechanism that is different but complementary to that used by vCCI.

A crystal structure for A41 has been reported [22] showing a globular β-sandwich domain strikingly similar in fold to vCCI (Figure 1). Like vCCI, the β6–β7 loop of A41 contains a large α-helix. Also like vCCI, A41 has a large negatively charged patch in sheet II (Figure 4a), Although the sequence of this charged patch is not conserved with vCCI, the sequence of the nearby area is, leading to the suggestion that this region may contain the chemokine binding site [22]. There are a few notable differences between these stuctures. A41 has a much shorter β2–β3 loop than vCCI (Figure 3). Transfer of the extended loop from vCCI to A41 does not confer any additional ability to bind chemokine [49]. Also, the large β7–β9 loop that passes across the face of β-sheet I in vCCI adopts a different position in A41. Here the loop makes a unique decoration, and along with residues in the C-terminus forms a small anti-parallel β-sheet. Ectromelia E163 has been shown to bind GAGs [51]. The myoxma virus vCCI (M-T1) is known to interact with GAGs through a basic patch on β sheet I in the face opposite the chemokine binding site [48]. It is likely that all members of the A41 family conserve a large positively charged patch in that region (Figure 4a).

**Figure 5:**
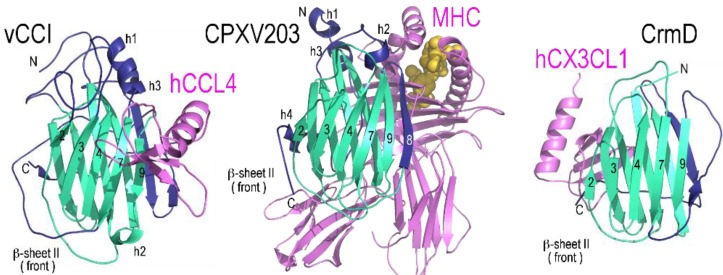
PIE domain proteins use unique determinants to engage ligands. vCCI primarily employs sheet II to bind CC-chemokines (2FFK) [20], CPV203 (T4) uses the edge of the β-sandwich plus part of sheet I to bind MHC class I/peptide complexes (4HKJ) [28], and CrmD appears to use sheet I for the binding of a low-affinity chemokine (3ON9) [25]. All ribbon diagrams are shown with the PIE domain in the same orientation.

### 2.3. CrmD C-Terminal Domain

Poxviruses encode a family of secreted immune evasion proteins with N-terminal sequence similarity to host tumor necrosis factor (TNF) receptors. Originally discovered in Shope fibroma virus, many other orthologues have since been identified in both leporipoxviruses and orthopoxviruses, and all have been shown to be sufficient for TNF binding [24,53,54,55,56]. Referred to as cytokine response modifiers (Crms), the family now includes four different proteins called CrmB, CrmC, CrmD, and CrmE. Data has shown that deletion of CrmD from ectromelia virus results in a severely attenuated virus in a mouse model with the median lethal dose increasing by six orders of magnitude. Interestingly, the mice given WT virus show no signs of inflammation at the site of infection, while mice given the CrmD-KO virus display a vigorous inflammatory response. This data clearly shows that CrmD is a potent anti-inflammatory factor [57].

While all Crm proteins display the characteristic cysteine-rich N-terminal domains common to TNF receptors, both CrmB and CrmD contain an approximately 160 amino acid C-terminal domain that is quite distinct from the TNF-binding region. It was later discovered that the C-terminal extension confers the ability to bind to a distinct set of chemokines [24]. Using a sequence alignment of the CrmB and CrmD C-terminal domains from VACV, CPXV, and ECTV, Alejo and coworkers identified three additional proteins containing similar domain sequences, which they termed the smallpox virus-encoded chemokine receptor or SECRET domain. The proteins were named SECRET-containing proteins SCP-1, SCP-2, and SCP-3. These SCPs bind the same set of chemokines (human and mouse CCL28, CCL25, CXCL12b, CXCL13, CXCL14, and mouse CCL27 and CXCL11) as the C-terminal domains from VACV CrmB and ECTV CrmD. Their data indicate that the chemokine-binding specificity profile of the SECRET domain may be similar for all members of the family [24]. Although no crystal structure has yet been reported for SCP-1, -2, or -3, the fact that all bind the same chemokines despite their relatively low sequence similarity suggests a structural similarity.

Based on de novo modeling, it was predicted that the SECRET domain of CrmB would have structural similarity to vCCI and A41 [23]. This was confirmed in a report describing the crystal structure of ECTV CrmD both alone and in complex with the chemokine CX_3_CL1 [25]. Like vCCI and A41, the outside of the β-sheet II surface is completely solvent exposed (Figure 1). On the opposite face, one half of β-sheet I is covered by a long C-terminal loop that follows from strand β11. But the long β7–β9 loop that spans the center of β-sheet I in vCCI stays on the β-sheet II side of CrmD, where it becomes a new strand anti-parallel to β9 (called β8). Also, the length of the β2–β3 loop is much shorter than in vCCI, closer in length to that of A41. Likewise, the β6–β7 loop that crosses between sheets I and II, and forms the distinctive α-helix found in both vCCI and A41, is much shorter, resulting in the helix being absent. Together, these differences make the CrmD structure appear more compact. CrmD has three of the four disulfides found in vCCI and A41, but is missing the one that normally connects the N-terminus to the end of β10. CrmD shares ~47% sequence identity with CrmB, suggesting that the respective PIE domains in CrmD and CrmB will be very similar in structure. The SCPs may also be similar in structure to CrmD. For example, even SCP-1, which is the most divergent of the SCPs in sequence from ectromelia virus CrmD, is predicted by the Phyre2 server [58] to resemble CrmD in structure—yielding a 93% confidence score with 79% coverage and 25% sequence identity over ectromelia virus CrmD (pdb 3ON9).

The structure of CrmD with a chemokine [25] indicates the chemokine-binding site is located on the face opposite that used by vCCI and A41 (on β-sheet I, see Figure 4 and Figure 5). This study was performed with human CX_3_CL1, a chemokine that binds CrmD with lower affinity (K_D_ = 0.68 uM) as compared to previously characterized chemokine ligands for the SECRET domain. Chemokines such as CCL28, CCL25, CXCL12b, CXCL13, CXCL14, XCL1 and CCL20 bind in the low nM range [24]. However, swapping three negatively charged binding site residues to alanine by site directed mutagenesis appeared to disrupt CrmD binding of several CC- and CXC chemokines, indicating that the SECRET domain may bind different chemokines in a similar manner. Still, the residues that contact CX_3_CL1 in the CrmD structure are not highly conserved in SCP-1, SCP-2, and SCP-3 despite their uniform chemokine binding profiles. Additional studies will be required to determine if all SECRET domains engage chemokine in the same way. Further, due to the apparent redundancy, it is worth considering that chemokine binding by SECRET domains may be a vestigial property and no longer their primary function.

### 2.4. CPXV203

Another PIE domain containing protein, CPXV203, is encoded by cowpox ORF CPXV_BR_203 and shares roughly ~25% sequence identity with cowpox vCCI over 69 of 231 residues. CPXV203 down regulates MHC class I in both murine and human cells during normal poxvirus infection [26,27]. CPXV203 works to prevent T-cell killing of infected cells in concert with another cowpox protein, CPXV12, a protein that effectively blocks the TAP-mediated transport of cytosolic peptides for MHC class I loading [59,60,61]. CPXV203 binds a wide array of both classical and non-classical MHC class I proteins and prevents them from trafficking to the plasma membrane by a mechanism dependent upon its C-terminal “KTEL” motif (recognized by the KDEL receptor) [27,62]. The KDEL-receptor recycling pathway normally functions as an ER-retrieval system, and is employed to capture defective chaperone-complexed MHC class I proteins in the Golgi and return them to the ER for new attempts at peptide-loading [63]. CPXV203 binds fully assembled MHC proteins in a highly pH dependent manner, with tighter complexes formed at the more acidic pH associated with the Golgi compartment. CPXV203 engages the underside of the MHC class I peptide-binding platform, contacting both the heavy chain α2 and α3 domains as well as β2m. These surfaces are extremely well conserved among MHC family members. In fact, elements of the MHC class I interface contacted by CPXV203 are required for tapasin, CD8, and natural killer (NK)-receptor engagement. Once back in the ER, CPXV203 releases MHC class I proteins due to the higher pH of that compartment in a process controlled by at least two His residues in CPXV203.

Our crystallographic analysis revealed that CPXV203 is structurally related to the poxvirus chemokine binding proteins vCCI and A41 (Figure 1) [28]. In contrast to vCCI and A41 which bind chemokines through β-sheet II, CPXV203 uses β-sheet I to bind MHC (see Figure 5). It divides the interface almost equally among the peptide-binding platform, β2m, and α3 domain. As in CrmD, the β7-β9 loop does not block accessibility to β-sheet I but remains in β-sheet II where if forms a new edge strand (β8). In addition to the four disulfides found in vCCI and A41, CPXV203 has one additional disulfide linking the β1 strand to a decoration of two α-helices located in the C-terminus. This decoration is used by CPXV203 to contact the MHC α2-domain. The β5-β6 loop is the source of nearly all α3 domain contacts. The β2m contacts come from the edge of the β-sandwich contributed by stands β8 and β10. Like CPXV203, other members of the orthopoxvirus T4 family contain a C-terminal KTEL motif, and so presumably interact with the KDEL-receptor. Members of the T4 family are also found in leporipoxvirus, cervidpoxvirus, and caporipoxvirus, and these share ~45% sequence identity with CPXV203. No greater than eight of the 22 CPXV203 residues known to make close contact with MHC class I in the co-complex structure (Figure 5) are conserved in any other member of the T4 family. Also missing are the two histidine residues that control the pH-dependent variation in MHC-binding affinity displayed by CPXV203. Therefore it appears unlikely that all T4 family members bind MHC. Further, it is also unclear if all members of the T4 family interact with the KDEL receptor. For example, cervidpoxvirus (DPXV W83-004) encodes a C-terminal YDEL, the capipoxviruses a C-terminal HNEL (LSDV_2490_003, SPPV_A_002, GTPV_G20_002, GTP PEL_002), while the leporipoxvirus contains an RDEL (MYXV LAU_M-T4). And although wildtype myxoma virus M-T4 is retained in the ER, deletion of the RDEL sequence did not alter its intercellular localization. However increased inflammation and edema at the site of injection was observed in rabbits infected with the deletion virus *versus* the parental control [64]. Although the T4 proteins are likely to prove functionally distinct, they do appear evolutionarily related and so are presented here as a single family.

## 3. Putative PIE Domains

### 3.1. ORF-GIF Family

The parapoxvirus orf virus causes a contagious pustular dermatitis, primarily in ruminants. Cells infected with orf virus secrete a 28-kDa GM-CSF inhibitory factor (GIF) displaying low sequence identity with cowpox A41 (~28% over 88 of 202 residues) suggesting that these proteins may be related [49,65]. Orf virus GIF from strain NZ2 binds and inhibits ovine GM-CSF and IL-2 and although these cytokines share little primary sequence similarity, they are both short-chain four helical bundle cytokines [66]. GIF binds ovine GM-CSF with a K_D_ of 0.4 nM and ovine IL-2 with K_D_ of 1.0 nM, but does not bind human GM-CSF or IL-2, despite the fact that orf virus can infect humans [65]. The protein is highly glycosylated, and believed to form dimers and tetramers in solution as assessed by size exclusion chromatography [65]. The GIF protein contains seven cysteine residues, six of which align well with vCCI and A41. These likely correspond to disulfides A, B, and D (Figure 2). The same six cysteine residues are conserved in the GIF proteins of pseudocowpox virus (PCPVgp121) and parapoxvirus red deer (SB87gp117), which are respectively 89% and 40% identical to orf GIF over the entire sequence. Although pseudocowpox GIF from strain BO74 has been shown to bind GM-CSF and IL-2 [67], the parapoxvirus red deer GIF remains to be tested. No GIF protein has been reported to bind chemokine.

### 3.2. ORF-CBP Family

Members of the genus parapoxvirus secrete chemokine-binding proteins (CBP) that are functionally similar to members of the vCCI family in their ability to bind with high affinity and inhibit many CC-chemokines. In addition, parapoxvirus CBPs can also bind C- [68] and some CXC-chemokines [69]. Although bovine papular stomatitis virus CBP (BPSVgORF112) and orf virus CBP (ORFVgORF112) share only 40% sequence identity, both have been reported to inhibit these three classes of chemokines. Functionally, orf virus CBP has been shown to inhibit the recruitment of pro-inflammatory monocytes into skin using a mouse model of lipopolysaccharide-induced inflammation [70], as well as dendritic cell trafficking and subsequent activation of T-cells [71]. Orf virus CBP shares 26% sequence identity with orf GIF, and conserves the positioning of the six cysteine residues within GIF that form three of the four disulfides found in vCCI and A41. Site-directed mutagenesis identified four residues of CCL2 that, when changed to alanine, alter orf CPB binding [68]. These residues lie within a region contacted by the CCL2 receptor CCR2 [72], and the same residues were previously shown to be required for high affinity interaction of CCL2 with vCCI [30,31]. Together, these data demonstrate that orf CBP inhibits chemokine activity by blocking the receptor-binding site on chemokines, in a manner similar to that employed by the vCCI family.

### 3.3. Search for Additional PIE Domains

The PIE and putative PIE proteins described above share some important attributes. All are soluble proteins having signal peptides for targeting to the secretory pathway. So far, all PIEs are noteworthy in that they lack obvious sequence relationships with any other eukaryotic or prokaryotic protein and all appear to be nonessential for virus replication. They are small in size, ranging between about 17 and 35-kDa. Additional information can be taken from a structure-based alignment of the four known PIE domains (Figure 3). The β-sandwich scaffold allows large insertions at only a few postitions. The most prominent insertions occur in the β6-β7 loop, the β7-β9 loop, and at the C-terminus. These contain decorations of short β-strands or α-helices, but in all cases are anchored to the scaffold by a pattern of disulfide bonds (labeled A-E in Figure 2 and Figure 3). The main structural differences between the PIE domain families occur in the length and placement of the decorations. Correspondingly, they may contain as few as a single disulfide bond, but three or four are more common. As the contact sites for the different ligands map mostly to the decorations (Figure 3 and Figure 4), it is clear their effect is to alter ligand specificity.

With this information in hand and a review of the literature, we set out to find other PIE-containing proteins in chordopoxvirus. A total of 33 genomes spanning 10 genera were examined (Table 1).

**Table 1:** Genomic sequences of the poxviruses used in this study.

Genus	Species	Strain	Abbreviation	GenBank #
Orthopoxvirus	Variola virus	Brazil 1966 (v66-39 São Paulo)	VARV BRZ66	DQ441419
		Congo 1970 v70-46 Kinshasa	VARV CNG70	DQ437583
		Garcia-1966	VARV GAR	Y16780
		Guinea 1969 (005)	VARV GUI69	DQ441426
		India 1964 7124 Vellore	VARV IND64	DQ437585
		Sierra Leone 1969 (V68-258)	VARV SLN68	DQ441437
	Monkeypox virus	Sierra Leone V70	MPXV SL	AY741551
		Zaire-96-I-16	MPXV Z96	AF380138
	Camelpox virus	M-96	CMLV M96	AF438165
	Taterapox virus	Dahomey 1968	TATV	NC_008291
	Horsepox virus	MNR-76	HPXV	DQ792504
	Cowpox virus	GRI-90	CPXV GRI	X94355
		Brighton Red	CPXV BRI	AF482758
		Germany 91-3	CPXV GER	DQ437593
	Ectromelia virus	Moscow	ECTV MOS	AF012825
	Vaccinia virus	Western Reserve	VACV WR	NC_006998
Unclassified	Yoka poxvirus	DakArB 4268	YOKA	NC_015960
Leporipoxvirus	Myxoma virus	Lausanne	MYXV LAU	AF170726
Yatapoxvirus	Yaba monkey tumor virus	YLD	YMTV YLD	AJ293568
Unclassified	Cotia virus	SPAn232	COTV	NC_016924
Cervidpoxvirus	Deerpox virus	W-848-83	DPXV W83	AY689436
Capripoxvirus	Lumpy skin disease virus	Neethling 2490	LSDV 2490	AF325528
	Sheeppox virus	A	SPPV A	AY077833
	Goatpox virus	G20-LKV	GTP G20	AY077836
		Pellor	GTP PEL	NC_004003
Suipoxvirus	Swinepox virus	17077-99	SWPV 99	AF410153
Parapoxvirus	Bovine papular stomatitis virus	BV-AR02	BPSV	NC_005337
	Orf virus	OV-SA00	ORF	NC_005336
	Pseudocowpox virus	VR634	PCPV	NC_013804
	Parapoxvirus red deer	HL953	SB87	NC_025963
Avipoxvirus	Fowlpox virus	FCV	FWPV FCV	AF198100
	Canarypox virus	Wheatley C93	CNPV WC93	NC_005309
Molluscipoxvirus	Molluscum contagiosum virus	Subtype 1	MOCV SB1	MCU60315

A combination of methods was used to detect possible PIE domains. First, a hidden Markov model of the PIE domain core was constructed by removing the decorations from the alignment in Figure 4. (HMMER program, available on the web at http://hmmer.janelia.org/) [73]. This Markov model was used to screen the genomic sequences. The genomes were also screened by Blastp [74] analysis using existing PIE amino acid sequences. Candidate ORFs were included as PIEs if they had a signal peptide and threaded to any of the known PIE structures using Phyre2 [75,76]. Additional Markov models were constructed as the set expanded, and the screening process was repeated. The resulting sequences were distributed into 20 families based on primary sequence similarity and available functional data (see Table 2).

**Figure 6:**
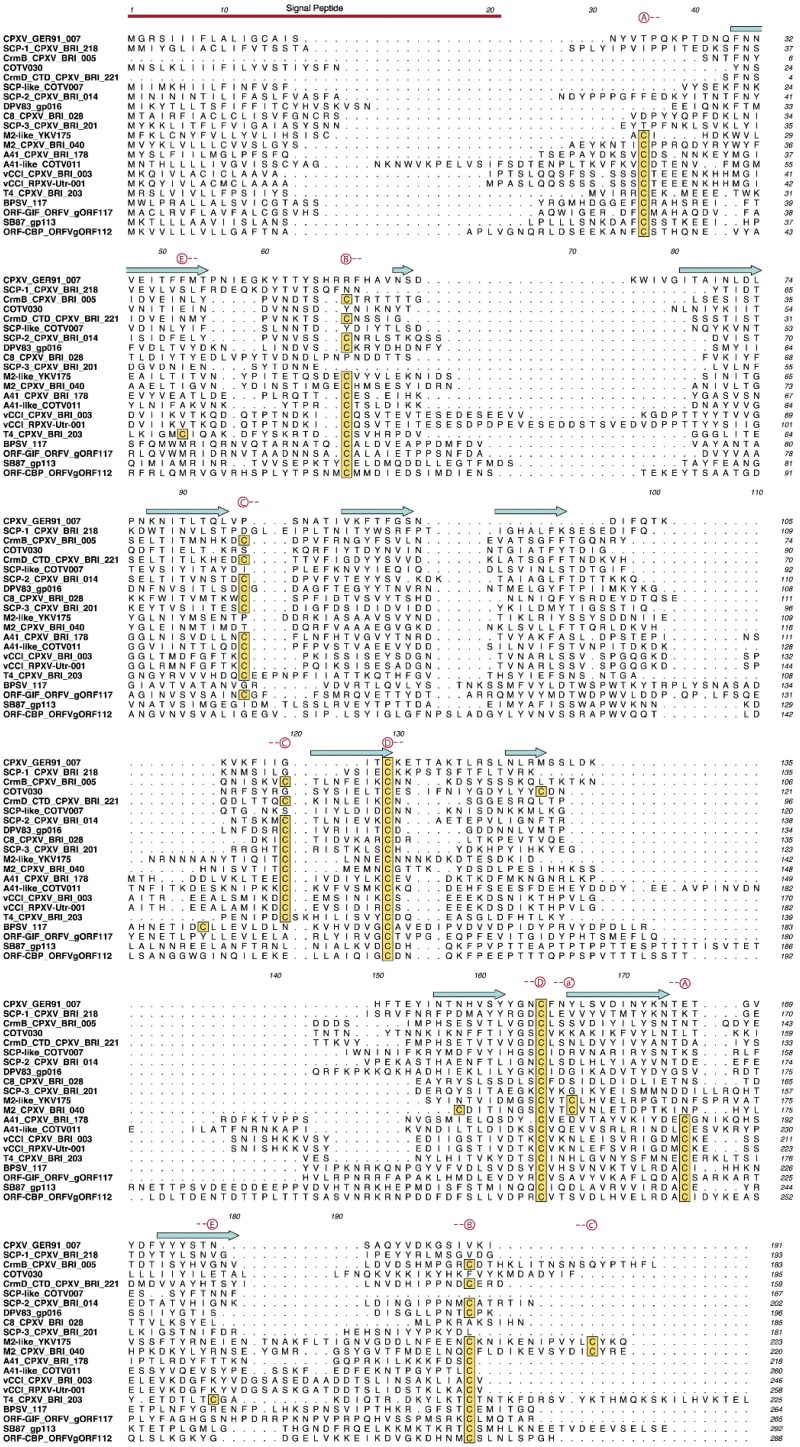
Sequence alignment of representative members of the PIE families. The positions of the vCCI core strands (cyan) are shown above the sequences. Cysteines are boxed in yellow. The predicted disulfide bonds are lettered in red. The predicted signal peptides are shown under the red bar. The PIE family name is given before the ORF name when they differ.

Table 2 provides a framework in which to begin the discussion of PIE domain variants. The list is likely incomplete as there may be PIE encoding ORFs that were too sequence diverse to be detected by these methods. Also, the family assignments should be considered tentative as functional and structural information is still scarce. The first 10 families across the top of the table are arranged in order of their position within the cowpox genome (vCCI-CrmD). For each strain, the number of ORFs in each PIE family is entered in the table. A number 2 in the table indicates that the virus contains two identical copies of that ORF, with one exception. The number 2 in the ORF-CBP column represents two closely related but distinct genes, SB87-111 and SB87-112. A number 0 indicates that less than half of that ORF is present, or that the ORF is reported to lack expression. Further investigation at the nucleotide level would detect additional ORF remnants [77] but was considered beyond the scope of this study. Figure 6 contains a sequence alignment showing a representative member from each PIE family.

When possible, the sequence from cowpox strain Brighton Red is shown in Figure 6, but sequences from other strains or viruses are shown when not in Brighton Red. The multiple sequence alignment was constructed in ClustalX [78] from the structure-based alignment of Figure 4 by matching all other sequences to that profile one at a time and merging the results. Hand editing was kept to a minimum. A summary of predicted physical properties for proteins in the representative sequence alignment is given in Table 3.

**Figure 7:**
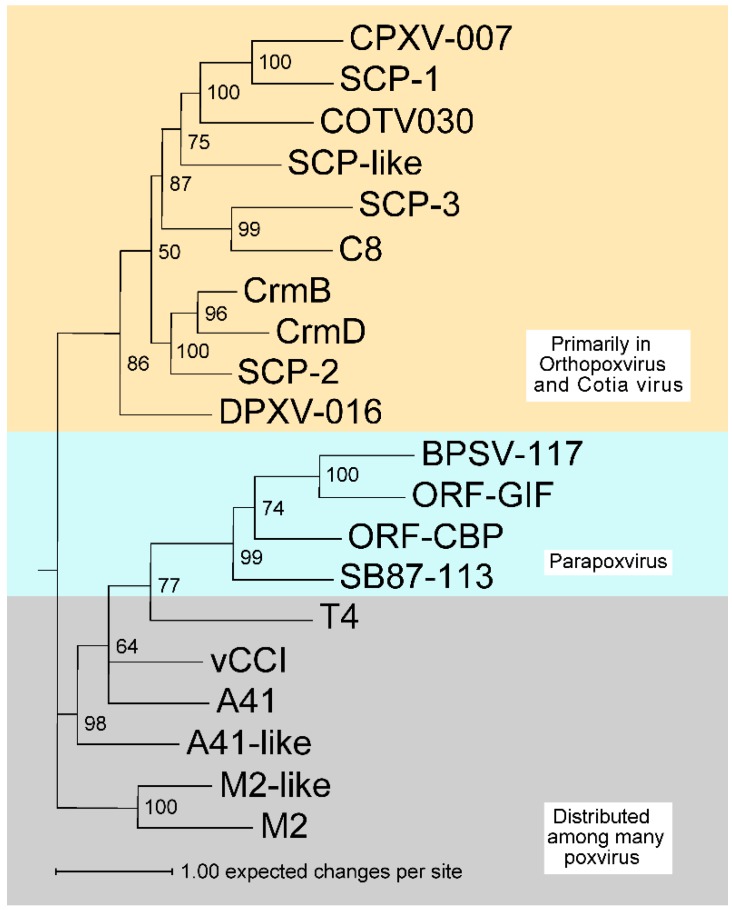
Dendrogram of PIE domain sequences showing relatedness among representative members of the PIE domain families. The tree is midpoint rooted for purposes of illustration. Values in percent at internal nodes indicate posterior probabilities calculated for the Bayesian inference of phylogeny from the alignment in Figure 6 using MrBayes v3.2.0 [81]. The scale bar relates branch lengths to the number of expected substitutions per site. The family names are shown at the terminal nodes.

Alignments of individual PIE families can be found in the supplementary online materials (Figures S1–S15). Figure 7 contains a midpoint-rooted phylogenetic tree representing the sequence relationships among the 20 families constructed using the PIE domain alignment of Figure 6 after removal of the signal peptide sequences (default settings at ViPR tools, http://www.viprbrc.org). PIE proteins share a core fold, and therefore patterns of secondary structure elements, hydrophobic packing residues, and disulfide bonds, but these properties do not always translate into similarities at the level of primary sequence. Consequently, many of the branch lengths between families in the dendrogram are large. A more detailed phylogram, employing all the PIE sequences from Table 2 is given in the supplement (Figure S15). The family assignments presented in Table 2 developed in large part from that analysis. It is worth noting that no PIE encoding ORFs appear in the species of Suipoxvirus, Avipoxvirus and Mullscipoxvirus used in this study. We detected three PIE domain families in leporipoxvius and orthopoxvirus (vCCI, CrmB, and M2), suggesting the PIE domain was likely present at the time of their divergence.

**Table 2:** Distribution of PIE and Putative PIE Domains.

Genus	Strain	vCCI	CrmB	SCP-2	C8	M2	A41	SCP-3	T4	SCP-1	CrmD	COTV030	CPXV-007	SCP-like	M2-like	A41-like	ORF-CBP	SB87-113	DPXV-016	BPSV-117	ORF-GIF
Orthopoxvirus	VARV BRZ66	1	1			1	1														
	VARV CNG70	1	1			1	1														
	VARV GAR	1	1			1	1														
	VARV GUI69	1	1			1	1														
	VARV IND64	1	1			1	1														
	VARV SLN68	1	1			1	1														
	MPXV SL	2	2			1	1	1		1											
	MPXV Z96	2	2			1	1	1	1	1											
	CMLV M96	2	2		1	1	1	0	1	1											
	TATV	0/0	0/0		0	1	1	1	1	1											
	HPXV	2	2	0	1	1	1	1	0	1											
	CPXV GRI	2	2	1	1	0	1	1	1	1	1										
	CPXV BRI	2	2	1	1	1	1	1	1	1	1										
	CPXV GER	2	2	1	1	1	1	1	1	1	0		1								
	ECTV MOS	2	0/0	1			1	1			2										
	VACV WR	0/0	0/0	0/0	1	1	1	1	0	1											
Unclassified	YOKA					1			2						1						
Leporipoxvirus	MYXV LAU	2	2			1			2												
Yatapoxvirus	YMTV YLD					1									1						
Unclassified	COTV			1		1						1		2		2					
Cervidpoxvirus	DPXV W83					1			2						1				1		
Caporipoxvirus	LSDV 2490								2												
	SPPV A								2												
	GTP G20								2												
	GTP PEL								2												
Parapoxvirus	BPSV																1			1	
	ORF																1				1
	SB87																2	1			1
	PCPV																1				1
Ligands		Chemokines	Chemokines	Chemokines	Unknown	Unknown	CK/GAGs	Chemokines	MHC class I	Chemokines	Chemokines	Unknown	Unknown	Unknown	Unknown	Unknown	Chemokines	Unknown	Unknown	Unknown	GM-CSF, IL-2

**Table 3:** Predicted Physical Properties of PIE and Putative PIE Domains. The amino acid length, molecular weight, isoelectric point, and number of cysteines for each protein were calculated using ProtParam [79]. The number of predicted N-linked carbohydrates was calculated using NetNGlyc1.0 [80]. The disulfide positions were taken from the alignment in Figure 6 and lettered as in Figure 2. The disulfides shown in bold typeface were determined from the structures in Figure 1.

Gene	Length (aa)	Mol wt (Da)	pI	N-linked	Cysteines	Disulfides
vCCI (CPXV-BRI-003)	226	24487	4.6	0	8	**A B C D**
CrmB-CTD (CPXV-BRI-005)	183	20567	4.8	2	6	_ B C D
SCP-2 (CPXV-BRI-014)	181	20208	4.5	5	6	_ B C D
C8 (CPXV-BRI-028)	164	19242	4.4	2	4	_ _ C D
M2 (CPXV-BRI-040)	203	23302	5.1	4	9	A' B C' D
A41 (CPXV-BRI-178)	202	22899	5.2	1	8	**A B C D**
SCP-3 (CPXV-BRI-201)	160	18793	5.3	0	4	_ _ C D
T4 (CPXV-BRI-203)	209	24027	6.8	1	10	**A B C D E**
SCP-1 (CPXV-BRI-218)	175	20133	5.0	2	2	_ _ _ D
CrmD-CTD (CPXV-BRI-221)	159	17648	4.5	1	6	**_ B C D**
COTV030	174	20738	9.2	3	3	_ _ _ D
CPXV_GER91_007	174	20070	9.1	2	2	_ _ _ D
SCP-like (COTV007)	151	17839	5.8	3	2	_ _ _ D
M2-like (YKV175)	203	23019	4.5	3	8	A' B C' D
A41-like(COTV011)	241	27586	4.7	1	8	A B C D
ORF-CBP (ORF-112)	272	29780	4.6	4	6	A B _ D
SB87-gp113	276	31204	4.6	4	6	A B _ D
DPXV-016	172	19713	5.3	1	6	_ B C D
BPSV-117	245	27765	6.1	4	7	A B _ D
ORF-GIF (ORFVgORF117)	246	28111	6.2	4	7	A B _ D

### 3.4. PIEs of Unknown Function

Survey of the published genomes revealed 10 potential PIE families of unknown function. The largest of these families is M2, found in almost all orthopoxvirus examined, and also in leporipoxvirus, yoka poxvirus, and cotia poxvirus. M2 is predicted to have an unusual disulfide-bonding pattern (Table 3). It appears to have four disufides like vCCI (ABCD). However, the second cysteine of bond A is predicted to occur 11 residues earlier than in vCCI, implying a difference near the C-terminus in the β10 strand. Also the first cysteine of bond C is missing and looks to have been replaced by a new cysteine about four residues from the C-terminus. Both new cysteine positions are conserved in nearly all members of the M2 family. While one study suggested M2 expression interferes with NF-kB activation [82], it appears likely the primary function of M2 remains unreported. Yoka poxvirus also contains a protein that appears to be a member of the M2 family (~69% to cowpox M2) and a second ORF with slightly less similarity to M2 (~34% identity). Presumably the two proteins, being in the same virus, serve different functions. We refer to the second protein as M2-like. Yaba monkey tumor virus and deerpox virus also encode M2-like proteins.

A second putative PIE family, C8, is found in orthopoxvirus. C8 bears a strong resemblance to SCP-3 (~28% identity in cowpox). C8 and SCP-3 are two of the smallest PIE proteins, and the only two predicted to have two disulfides.

The remaining families have only one or two members. The single ORF encoding CPXV-007 is unique to strain Germany 91-003 of cowpox, not being found in the strains Brighton Red or GRI-90. It shares ~17% identity with cowpox SCP-1 and is nearly identical in size. Both CPXV-007 and SCP-1 are predicted to contain a single disulfide. The CPXV-007 protein is also predicted to have one of the highest pI values (9.1) of all the PIE domains examined (Table 3). Cotia virus contains a protein, COTV030, that is very similar in size, disulfide-bonding pattern, and pI (9.2) to CPXV-007 but they share very little sequence similarity. Parapoxvirus of red deer strain HL953 contains two proteins of unknown function, SB87-112 and SB87-113. These are similar in size, sequence, and cysteine placement to that of orf GIF and orf CBP. By sequence similarity they have been placed in the ORF-CBP family. Cotia virus encodes two familes with duplicate ORFs. The first of these, the SCP-like family, contains COTV007 and COTV179. The second, the A41-like family, contains COTV011 and COTV175. Four other ORFs from Cotia virus may encode PIE domains but the signal peptides do not appear to be functional. These include COTV001, COTV004, COTV182, and COTV185. The cervidpoxvirus protein DVXV-016 looks very similar to the SECRET domains of orthopoxvirus. The BPSV-GIF protein was cloned from bovine papular stomatitis virus because it shared ~37% with orf GIF. It conserves the six cysteines and the WSXWX-like motif required for orf GIF function, but does not bind GM-CSF or IL-2 [67]. RANTES (CCL5) binding has been reported in the supernatant of PCPV-infected cells [67]. The ORF-CBP protein PCPV-116 is a likely candidate for this activity.

## 4. Conclusions

Our sequence analysis suggests that many poxvirus ORFs of unknown function likely encode members of the PIE domain superfamily. We predict that these proteins share a core structural scaffold, one that has been modified repeatedly to create different binding specificities for host molecules. Although the PIE domain was initially discovered in proteins that bind chemokines, it is now clear that it is functionally diverse and that decorations to the domain core confer unique binding specificities. This situation is reminiscent of the immunoglobulin (Ig) domain superfamily. Each Ig domain consists of two β-sheets, assembled as a β-sandwich, that are held together by an inner layer of buried hydrophobic residues. The binding specificity of each Ig domain is determined largely by the loops that connect its β-strands. These loops can vary in size, containing insertions ranging from a few residues to whole domains. Consequently, within the Ig domain superfamily, structure is often more conserved than primary sequence [83]. It is this ability to accommodate shifts in surface decoration that make the Ig fold a useful scaffold for generating new binding specificities. Ig domains are extremely versatile. They are found in: antibodies, cell surface receptors, extracellular matrix proteins, bacterial chaperones, intracellular regulatory proteins, and poxviral proteins, to name just a few [84,85]. The PIE fold is distinct from the Ig fold, both in strand organization and connectivity, but it similarly accommodates shifts in surface decoration. Judging from the extensive sequence diversity of the PIE-domains surveyed here, we predict cytokine and MHC binding to be just the tip of the PIE function iceberg.

## Supplementary Files

Supplementary File 1
