# Supplementary material for: Structural Conservation and Functional Diversity of the Poxvirus Immune Evasion (PIE) Domain Superfamily
Source: Viruses. 2015 Aug 28;7(9):4873–93. doi: 10.3390/v7092848 (PMC4584292; doi:10.3390/v7092848)
Supplement: Supplementary File 1 [file viruses-07-02848-s001.zip › Nelson_Supplements/Figure_S10_M2-like.pdf]

M2-like Family Alignment

```
M2-like_YKV175       MFKLCNYFVLLYVLIHSISC.................ACI...HDKWVLE   30
M2-like_YMTV_YLD_9L  MEGVKKLFNFLILFFFVSTTLSYI....LKQDHKDFGKCVSKKYRYWNLA   46
M2-like_DPXV_W83_17  MAKLFYFILISICISSCLS...SPLSNRNGKLSSSLGKCNSHRYRFWNLA   47

M2-like_YKV175       AILTITVNYPITETQSDECVYVLEKNIDSSINITGYGLNIYMSENTPDDR   80
M2-like_YMTV_YLD_9L  AVLTLGLTYTIPEKE..QCFAHIHL..DTTLVIG.YGLSIEIEITNKIDG   91
M2-like_DPXV_W83_17  SSLVIGLTYPIKEQE..ECKIDIIS..NSHISITGYGINVDFEFTTEITK   93

M2-like_YKV175       KIASAAVSVYND.TIKLRIYSSYSDDNIIENRNNNANYTIQITCLNNECN  129
M2-like_YMTV_YLD_9L  QVVSVVEGLYNNNTIILLLFIAN.DRSDYEN.SVIPNTKISVTCTDIDCD  139
M2-like_DPXV_W83_17  PLVASAEGFVNN.TLVILIFMAD.DRSSNAN.NSIPDTKVTITCLDVDCD  140

M2-like_YKV175       NNKDKDTESDKIDSYINTVIDMGSCVTCLHVELRPGTDNFSPRVATVSSF  179
M2-like_YMTV_YLD_9L  NNPTRQVLNGKVSK..NELIIFGSCLTCVLLDTYPNSIKGFLPPSGIVAK  187
M2-like_DPXV_W83_17  IVSKRQNIDNLSTK..NELIIVGSCVTCVDLNVYPNSLYGFKSANSIIAR  188

M2-like_YKV175       TYRNEIENTNAKFLTIGNVGDDLNFEENCKNIKENIPVYLCYKQ..  223
M2-like_YMTV_YLD_9L  PYSKGVNEDNGYLWYRHKKD.......SCNVDFLNTSYSICSKIRR  226
M2-like_DPXV_W83_17  PYSGTSG...GYTWRNLNTSK.....RQCDVDTEKIDYYVCDKKSN  226
```

Figure S10. Sequence alignment of the M2-like family members. Cysteine residues are boxed in yellow. Predicted N-linked glycosylation sites are boxed in blue.
