# Supplementary material for: Structural Conservation and Functional Diversity of the Poxvirus Immune Evasion (PIE) Domain Superfamily
Source: Viruses. 2015 Aug 28;7(9):4873–93. doi: 10.3390/v7092848 (PMC4584292; doi:10.3390/v7092848)
Supplement: Supplementary File 1 [file viruses-07-02848-s001.zip › Nelson_Supplements/Figure_S11_C8.pdf]

C8L Family Alignment

```
                 1        10        20        30        40        50        60
CMLV_M96_18      MSSIRFIACIYLISVFGNCHE.DPYYQPFDKLNITLDIYTYEDLVPYTVDNDLPNPNDDTTSFVK   64
HSPV9_023        MSAIRFIACLCLIFVLGNCHD.DPYYQPFNKLNITLDIYTYEDLVPYTVDN.......DTTSFVK   57
CPXV_GRI_C12L    MSSIRFIACLCLISVLGNCHD.DPYYQPFDKLNITLDIYTYEDLVPYTVDNDLPRPNDDTTSFVK   64
CPXV_BRI_028     MTAIRFIACLCLISVFGNCRSVDPYYQPFDKLNITLDIYTYEDLVPYTVDNDLPNPNDDTTSFVK   65
CPXV_GER91_026   MSSIRFIACLYLISIFGNCHE.DPYYQPFDKLNITLDIYTYEDLVPYTVDNDLPNPNDDTTSFVK   64
VACV_WR_C8L      MSSIRFIACLYLISIFGNCHE.DPYYQPFNKLNITLDIYTYEDLVPYTVDN.......DTTSFVK   57

                      70        80        90        100       110       120
CMLV_M96_18      IYFKNFWITVMTKWCASFIDTVSLYTSHDNLNIQFYSRDEYDTQSEDKICTIDVKARCKHLTKRE   129
HSPV9_023        IYFKKFWITVMTKWCAPFIDTVSVYTSHDNLNIEFYTRDEYDTQSEDKICTIDVKARCNHLTKPE   122
CPXV_GRI_C12L    IYFKNFWITVMTKWCAPFIDTVSVYTSHDNLNIQFYTRDEYDTQSEDKICTIDVKARCKHRTKRE   129
CPXV_BRI_028     IYFKKFWITVMTKWCSPFIDTVSVYTSHDNLNIQFYSRDEYDTQSEDKICTIDVKARCDRLTKPE   130
CPXV_GER91_026   IYFKNFWITVMTKWCAPFIDTVSVYTSHDNLNIQFYNRDEYDTQSEDKICTIDVKARCKHLTKRE   129
VACV_WR_C8L      IYFKKFWITVMTKWCAPFIDTVSVYTSHDNLNIEFYTRDEYDTQSEDKICTIDVKARCNHLTKPE   122

                 130       140       150       160       170       180
CMLV_M96_18      VIVQQEAYRYSLSSDLSCFDSIDLEIDLIETNSTDTTVLKSYDLMLPARTKST..   182
HSPV9_023        VTVQKEAYRYSLSSDLSCFDSIDLNIDLIETNSTDTTVLKSYELMLPKRAKSIHN   177
CPXV_GRI_C12L    VTVQQEAYRYSLSSDLSCFDSIDLEIDLIETNRTDTTVLKSYELMLPARTKST..   182
CPXV_BRI_028     VTVQEEAYRYSLSSDLSCFDSIDLDIDLIETNSTDTTVLKSYELMLPKRAKSIHN   185
CPXV_GER91_026   VTVQQEAYRYSLSSDLSCFDSIDLEIDLIETNSTDTTVLKSYELMLPKRAKSIHN   184
VACV_WR_C8L      VTVQKEAYRYSLSSDLSCFDSIDLDIDLIETNSTDTTVLKSYELMLPKRAKSIHN   177
```

Figure S11. Sequence alignment of the C8 family members. Cysteine residues are boxed in yellow. Predicted N-linked glycosylation sites are boxed in blue.
