# Supplementary material for: Structural Conservation and Functional Diversity of the Poxvirus Immune Evasion (PIE) Domain Superfamily
Source: Viruses. 2015 Aug 28;7(9):4873–93. doi: 10.3390/v7092848 (PMC4584292; doi:10.3390/v7092848)
Supplement: Supplementary File 1 [file viruses-07-02848-s001.zip › Nelson_Supplements/Figure_S12_ORF-CBP.pdf]

ORF-CBP Family Alignment

```
BPSVgORF112   .MKSTYLLFALSLVLSAALSAPMN....TDEQRNWCRIHKDKLYSRFRLYMKITR   50
ORFVgORF112   .MKVVLLLVLLGAFTNAAPLVGNQ.RLDSEEKANFCSTHQNEVYARFRLQMRVGV   53
SB87_gp112    ..MKRFIFAALCLLLSSVQPAPVVTY..TSEKENFCHAYPDHMYARFRLYMKIQR   51
SB87_gp111    .MRCLILTAVLAAALALIGAAPSS.....PEKKEFCEAHPYDLYARLRLFMKIER   49
PCPV_gp116    MRAFVLLLALVGAFAHAVPMREHYGNPDSAEKQKFCLTHNTEMYAKFRLYMRIQV   55

BPSVgORF112   ..TTVTPPTGMCMLDIENSNMDLDETDEVTNVTSTISFRDSTATFEASGVNGSIT   103
ORFVgORF112   RHSPLYTPSNMCMMDIEDSIMDIENSTEKE........YTSAATGDANGVNVSVA   100
SB87_gp112    ..NLYSSVKAQCTLDIETEKFESLD..............SSSAYVDASGINASIS   90
SB87_gp111    ..TLHSEVSSQCVLDIQNNRTGGLD..............WTEAYVEASGINVSVN   88
PCPV_gp116    RHSPMYEPSNMCMLDLETSRLDTDD..............LSMAKFEANGINGSIA   96

BPSVgORF112   LMGDEVHIPMSYIGVSFDPSSAY..VFVNVSSWTPWEKSTTDLAANDFEGLKNII   156
ORFVgORF112   LIGEGVSIPLSYIGLGFNPSLADGYLYVNVSSRAPWVQQTLDLSANGGWGINQIL   155
SB87_gp112    IMGENVYIPINYVGIAYDLKDEN..VYVNVSSFRPWIQTTKNLAVDGNKGLEELL   143
SB87_gp111    VFGDEVYIPLSYVSVAYDPTKMDGFVYVNVSSFRPWEQYTKNLSDEGYHGLKQIL   143
PCPV_gp116    LIGEDVSIPFSYIGVGFNPLLSR.YVYVNVSSWSPWDQLTAELTSYDFWGLRQIL   150

BPSVgORF112   NHQILAINLGCDHQYPAPKPTPK..PEKISTPST.TPGPTLLETEAPTTVPPVDT   208
ORFVgORF112   EKELLAIQIGCDNQKFPEEPTTTQPPSPVTTTLSSTTLDLTDENTDTTPLTTTSA   210
SB87_gp112    KNVMVSIDIGCDSQIITPGPYPTPPPPKVHTLSADEIEDLENS....TPPTPDVT   194
SB87_gp111    QYGVVLINVGCDGQAILTTPAPPTTPP..STETTTIEHDYVYA....SPTPPDTQ   192
PCPV_gp116    AKERLIIQLGCDHQKFPEEPTPRTTPAPQTTPEE.ELEEEDYEYYDELKPTPPNI   204

BPSVgORF112   SSDRKKKPDPVDFSLLVDTRCIKNVDLYFELKDACLAYKSQSPFKLVGQHGTGQQ   263
ORFVgORF112   SVNRKRNPDDFDFSLLVDPRCVTSVDLHVELRDACIDYKEASQLSLKGKYGDGEL   265
SB87_gp112    K.DRKRKPDKLEFSLLRDPACIKNVEVYFELMDACLAHKKETPLRFVGKTDNGEH   248
SB87_gp111    R.DRKKNPDSVTVIMDFDKTCIKDVQVYFEIRDACLDHKEKSPLRIVGKTDRGDG   246
PCPV_gp116    YVDRKRNPGELDFSLLADSRCIESVDLYVELKDACINYRHTSPLRLKGEHNNGEA   259

BPSVgORF112   VRQDMKSLGTSYRTCKLKTSSTADNDMESAKDEE   297
ORFVgORF112   VKKEIKDVGKDHNMCSLNLSPGH...........   288
SB87_gp112    VRKEVKQLD.GKRRCSMNLDSDYDDEELDP....   277
SB87_gp111    ILKEIKRLESGNRVCSMHLDQDSGNETFDV....   276
PCPV_gp116    VRKEIKTLTDSHTICSMNMNPYDK..........   283
```

Figure S12. Sequence alignment of the ORF-CBP family members. Cysteine residues are boxed in yellow. Predicted N-linked glycosylation sites are boxed in blue.
