# Supplementary material for: Structural Conservation and Functional Diversity of the Poxvirus Immune Evasion (PIE) Domain Superfamily
Source: Viruses. 2015 Aug 28;7(9):4873–93. doi: 10.3390/v7092848 (PMC4584292; doi:10.3390/v7092848)
Supplement: Supplementary File 1 [file viruses-07-02848-s001.zip › Nelson_Supplements/Figure_S15_phylogram.pdf]

Summary Phylogram of PIE and Putative PIE Domains

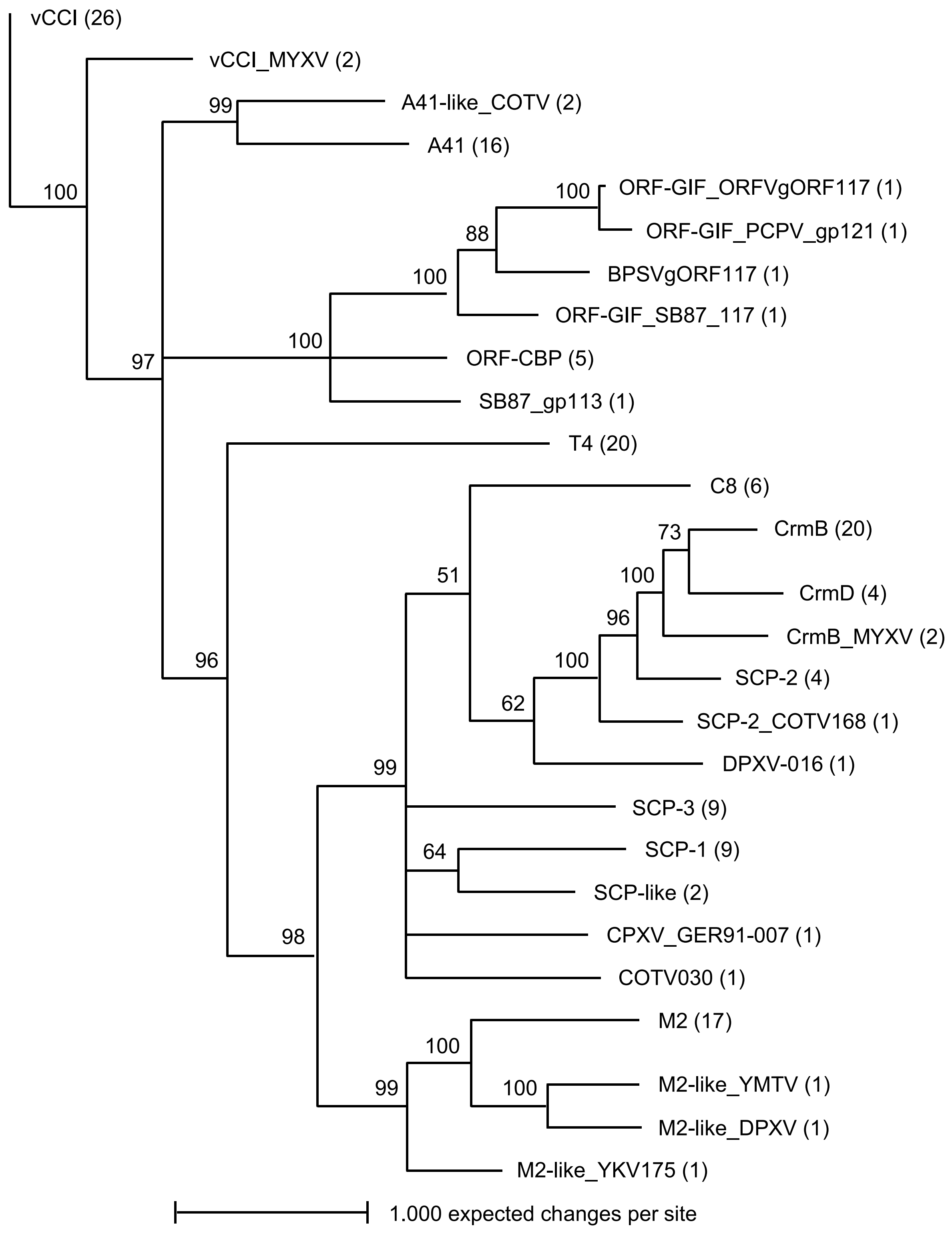

Figure S15. Summary of the phylogenetic analysis detailed in Figure S16. The tree is unrooted and displays an average of branch length for each group. Clade credibility values are given as percentages above the internal nodes. Values in parenthesis, at the terminal nodes, indicate the total number of ORFs in each catagory.
By similar analysis, the 156 PIE protein sequences were grouped into the 20 families shown in Table 2 of the manuscript. Some families contain duplicate sequences, due in part because many of the ORFs occur in terminal repeat regions, but also because many of these virus strains are closely related.
