# Supplementary material for: Structural Conservation and Functional Diversity of the Poxvirus Immune Evasion (PIE) Domain Superfamily
Source: Viruses. 2015 Aug 28;7(9):4873–93. doi: 10.3390/v7092848 (PMC4584292; doi:10.3390/v7092848)
Supplement: Supplementary File 1 [file viruses-07-02848-s001.zip › Nelson_Supplements/Figure_S16_mrbayes.pdf]

Amino Acid Relatedness of the PIE Domain Sequences

Although the PIE proteins are similar in structure, they share very little primary sequence similarity. Here, a total of 156 PIE protein sequences were grouped into 20 families as shown in Table 2 of the text. These families contained many duplicate sequences, due in part because of orfs located in ITR regions of the different poxvirus, but also because some of the virus strains are closely related. The signal peptides were removed (as predicted by SignalP4.1 server, http://www.cbs.dtu.dk/services/SignalP/). Each family was aligned using the MUSCLE server (http://www.ebi.ac.uk/Tools/msa/muscle/). Next poorly aligned positions and divergent regions were excised with Gblocks (http://molevol.cmima.csic.es/castresana/Gblocks.html). The trimmed sequences were aligned as a pool using MUSCLE and the resulting alignment passed for analysis to the program MrBayes, available as part of the Phylemon 2.0 package (http://phylemon.bioinfo.cipf.es/). The output from MrBayes is given below.

```
                            MrBayes v3.2.0
                    (Bayesian Analysis of Phylogeny)
              Distributed under the GNU General Public License
               Type "help" or "help command>" for information
                     on the commands that are available.
                   Type "about" for authorship and general
                       information about the program.

   Executing file "/infile_converted.phy"
   UNIX line termination
   Longest line length = 70
   Parsing file
   Expecting NEXUS formatted file
   Reading data block
      Allocated taxon set
      Allocated matrix
      Defining new matrix with 156 taxa and 350 characters
      Data is Protein
      Data matrix is interleaved
      Gaps coded as -
      Taxon   1 -> 139_vCCI_VARV_GAR_G3R
      Taxon   2 -> 140_vCCI_VARV_CNG70_206
      Taxon   3 -> 129_vCCI_VARV_BRZ66_206
      Taxon   4 -> 130_vCCI_VARV_SLN68_206
      Taxon   5 -> 155_vCCI_VARV_GUI69_206
      Taxon   6 -> 156_vCCI_VARV_IND64_206
      Taxon   7 -> 143_vCCI_CMLV_M96_211
      Taxon   8 -> 144_vCCI_CMLV_M96_
      Taxon   9 -> 133_vCCI_CPXV_GRI_D1L
      Taxon  10 -> 141_vCCI_CPXV_GRI_I5R
      Taxon  11 -> 134_vCCI_VACV_WR_B29R
      Taxon  12 -> 135_vCCI_VACV_WR_C23L
      Taxon  13 -> 142_vCCI_RPXV_Utrecht_35kDa
      Taxon  14 -> 136_vCCI_VACV_Lister_vCCI
      Taxon  15 -> 131_vCCI_ECTV_MOS_K2R
      Taxon  16 -> 132_vCCI_ECTV_MOS_001
      Taxon  17 -> 149_vCCI_CPXV_BRI_003
      Taxon  18 -> 152_vCCI_CPXV_BRI_227
      Taxon  19 -> 153_vCCI_CPXV_GER91_001
      Taxon  20 -> 154_vCCI_CPXV_GER91_217
      Taxon  21 -> 137_vCCI_HSPV002
      Taxon  22 -> 138_vCCI_HSPV206
      Taxon  23 -> 150_vCCI_MPXV_Z96_J3R
      Taxon  24 -> 151_vCCI_MPXVSL_001
      Taxon  25 -> 147_vCCI_MPXVSL_177
      Taxon  26 -> 148_vCCI_MPXV_Z96_J1L
```

```
Taxon  27 -> 145_vCCI_MYXV_LAU_MT1
Taxon  28 -> 146_vCCI_MYXV_LAU_m001L
Taxon  29 -> 3_A41like_COTV011
Taxon  30 -> 4_A41like_COTV175
Taxon  31 -> 5_A41_MPXV_SL_145
Taxon  32 -> 6_A41_MPXV_Z96_A41L
Taxon  33 -> 17_A41_CPXV_BRI_178
Taxon  34 -> 18_A41_CPXV_GER91_166
Taxon  35 -> 7_A41_VARV_GAR_A49L
Taxon  36 -> 10_A41_VARV_BRZ66_157
Taxon  37 -> 11_A41_VARV_IND64_158
Taxon  38 -> 12_A41_VARV_SLN68_157
Taxon  39 -> 9_A41_VARV_CNG70_157
Taxon  40 -> 8_A41_VARV_GUI69_157
Taxon  41 -> 13_A41_TATV_DAH68_A43L
Taxon  42 -> 16_A41_CPXV_GRI_A43L
Taxon  43 -> 15_A41_HSPV_163
Taxon  44 -> 20_A41_VACV_WR_A41
Taxon  45 -> 14_A41_CMLV_M96_160
Taxon  46 -> 19_A41_ECTV_MOS_140
Taxon  47 -> 80_GIF_ORFVgORF117
Taxon  48 -> 82_GIF_PCPV_gp121
Taxon  49 -> 21_BPSVgORF117
Taxon  50 -> 81_GIF_SB87_117
Taxon  51 -> 75_CBP_ORFVgORF112
Taxon  52 -> 79_CPB_PCPV_gp116
Taxon  53 -> 76_CBP_BPSVgORF112
Taxon  54 -> 77_CPB_SB87_gp112
Taxon  55 -> 78_CPB_SB87_gp111
Taxon  56 -> 109_T4like_MPXV_Z96_B10R
Taxon  57 -> 112_T4like_CPXV_GRI_B8R
Taxon  58 -> 110_T4like_CMLV_M96_MT4like
Taxon  59 -> 111_T4like_TATV_DAH68_195
Taxon  60 -> 113_T4like_CPXV_BRI_203
Taxon  61 -> 114_T4like_CPXV_GER91_191
Taxon  62 -> 115_T4like_YKV002c
Taxon  63 -> 116_T4like_YKV185
Taxon  64 -> 117_T4_MYXV_LAU_MT4
Taxon  65 -> 118_T4_MYXV_LAU_m004L
Taxon  66 -> 119_T4_DPXV_W83_004
Taxon  67 -> 120_T4_DPXV_W83_166
Taxon  68 -> 121_T4_LSDV_2490_003
Taxon  69 -> 122_T4_LSDV_2490_154
Taxon  70 -> 123_T4_SPPV_A_002
Taxon  71 -> 124_T4_SPPV_A_129
Taxon  72 -> 125_T4_GTPV_G20_002
Taxon  73 -> 126_T4_GTPV_G20_130
Taxon  74 -> 127_T4_GTP_PEL_002
Taxon  75 -> 128_T4_GTP_PEL_146
Taxon  76 -> 24_C8_VACV_WR_C8L
Taxon  77 -> 25_C8_HSPV9_023
Taxon  78 -> 22_C8_CPXV_BRI_028
Taxon  79 -> 23_C8_CMLV_M96_18
Taxon  80 -> 26_C8_CPXV_GER91_026
Taxon  81 -> 27_C8_CPXV_GRI_C12L
Taxon  82 -> 98_SCP3_MPXVSL161
Taxon  83 -> 99_SCP3_MPXV_Z96_B8R
Taxon  84 -> 101_SCP3_HSPV184
Taxon  85 -> 106_SCP3_VACV_WR_B7R
Taxon  86 -> 102_SCP3_CPXV_GRI_B6R
Taxon  87 -> 105_SCP3_ECTV_MOS_C3R
Taxon  88 -> 100_SCP3_TATV_DAH68_190
```

```
Taxon  89 -> 104_SCP3_CPXV_GER91_188
Taxon  90 -> 103_SCP3_CPXV_BRI_201
Taxon  91 -> 84_SCP1_MPXV_SL_170
Taxon  92 -> 85_SCP1_MPXV_Z96_B20R
Taxon  93 -> 90_SCP1_CPXV_GRI_B21R
Taxon  94 -> 87_SCP1_VACV_WR_C13L
Taxon  95 -> 86_SCP1_CPXV_GER91_204
Taxon  96 -> 91_SCP1_HSPV199
Taxon  97 -> 88_SCP1_CPXV_BRI_218
Taxon  98 -> 89_SCP1_TATV_DAH68_218
Taxon  99 -> 92_SCP1_CMLV_M96_206
Taxon 100 -> 107_SCPlike_COTV179
Taxon 101 -> 108_SCPlike_COTV007
Taxon 102 -> 30_CrmB_MPXV_SL_176
Taxon 103 -> 31_CrmB_MPXV_SL_002
Taxon 104 -> 32_CrmB_MPXV_Z96_J2R
Taxon 105 -> 33_CrmB_MPXV_Z96_J2L
Taxon 106 -> 40_CrmB_CMLV_M96_CrmB
Taxon 107 -> 41_CrmB_CMLV_M96a_CrmB
Taxon 108 -> 44_CrmB_CPXV_GRI_D2L
Taxon 109 -> 45_CrmB_CPXV_GRI_I4R
Taxon 110 -> 42_CrmB_HSPV_205
Taxon 111 -> 43_CrmB_HSPV_003
Taxon 112 -> 48_CrmB_CPXV_GER91_216
Taxon 113 -> 49_CrmB_CPXV_GER91_002
Taxon 114 -> 36_CrmB_VARV_GUI69_205
Taxon 115 -> 38_CrmB_VARV_SLN68_205
Taxon 116 -> 34_CrmB_VARV_GAR_G2R
Taxon 117 -> 37_CrmB_VARV_BRZ66_205
Taxon 118 -> 35_CrmB_VARV_IND64_205
Taxon 119 -> 39_CrmB_VARV_CNG70_205
Taxon 120 -> 46_CrmB_CPXV_BRI_230
Taxon 121 -> 47_CrmB_CPXV_BRI_005
Taxon 122 -> 52_CrmD_CPXV_GRI_K2R
Taxon 123 -> 53_CrmD_CPXV_BRI_221
Taxon 124 -> 54_CrmD_ECTV_MOS_I1R
Taxon 125 -> 55_CrmD_ECTV_MOS_003
Taxon 126 -> 93_SCP2_CPXV_GRI_D12L
Taxon 127 -> 95_SCP2_CPXV_GER_12
Taxon 128 -> 96_SCP2_CPXV_BRI_014
Taxon 129 -> 94_SCP2_ECTV_MOS_008
Taxon 130 -> 50_CrmB_MYXV_LAU_MT2
Taxon 131 -> 51_CrmB_MYXV_LAU_m002L
Taxon 132 -> 97_SCP2_COTV16897_SCP2_CO
Taxon 133 -> 1_M2_CPXV_BRI_040
Taxon 134 -> 2_M2_CPXV_GER91_038
Taxon 135 -> 61_M2_VARV_IND64_022
Taxon 136 -> 64_M2_VARV_CNG70_022
Taxon 137 -> 63_M2_VARV_SLN68_22
Taxon 138 -> 71_M2_VARV_GUI69_22
Taxon 139 -> 69_M2_VARV_BRZ66_26
Taxon 140 -> 70_M2_VARV_GAR_Q2L
Taxon 141 -> 65_M2_TATV_DAH68_033
Taxon 142 -> 62_M2_CMLV_M96_029
Taxon 143 -> 66_M2_MPXVSL21
Taxon 144 -> 67_M2_MPXV_Z96_O2L
Taxon 145 -> 68_M2_HSPV_034
Taxon 146 -> 72_M2_VACV_WR_M2L
Taxon 147 -> 60_M2_COTV028
Taxon 148 -> 73_M2_YKV_170
Taxon 149 -> 74_M2_MYXV_LAU_m154L
Taxon 150 -> 58_M2like_YMTV_YLD_9L
```

```
      Taxon 151 -> 59_M2like_DPXV_W83_17
      Taxon 152 -> 83_SB87_gp113
      Taxon 153 -> 29_CPXV_GER91_3_007
      Taxon 154 -> 57_M2like_YKV175
      Taxon 155 -> 28_COTV030
      Taxon 156 -> 56_DPXV_W83_gp016
      Successfully read matrix
      Setting default partition (does not divide up characters)
      Setting model defaults
      Seed (for generating default start values) = 1431529782
      Setting output file names to "/infile_converted.phy.run>.p|t>"
   Exiting data block
   Reached end of file

MrBayes >

RECEIVED CMD:  mcmc


   Running Markov chain
   MCMC stamp = 6113221175
   Seed = 1143393248
   Swapseed = 1431529782
   Model settings:

      Data not partitioned --
         Datatype  = Protein
         Aamodel   = Poisson
                     Substitution rates are fixed to be equal
         Covarion  = No
         # States  = 20
                     State frequencies are fixed to be equal
         Rates     = Equal

   Active parameters:

      Parameters
      ------------------
      Statefreq        1
      Ratemultiplier   2
      Topology         3
      Brlens           4
      ------------------

      1 --  Parameter  = Pi
            Type       = Stationary state frequencies
            Prior      = Fixed (equal frequencies)

      2 --  Parameter  = Ratemultiplier
            Type       = Partition-specific rate multiplier
            Prior      = Fixed(1.0)

      3 --  Parameter  = Tau
            Type       = Topology
            Prior      = All topologies equally probable a priori
            Subparam.  = V

      4 --  Parameter  = V
            Type       = Branch lengths
            Prior      = Unconstrained:Exponential(10.0)
```

```
 Number of taxa = 156
 Number of characters = 350

 The MCMC sampler will use the following moves:
    With prob.  Chain will use move
      11.11 %   ExtSPR(Tau,V)
      11.11 %   ExtTBR(Tau,V)
      11.11 %   NNI(Tau,V)
      11.11 %   ParsSPR(Tau,V)
      44.44 %   Multiplier(V)
      11.11 %   Nodeslider(V)

 Division 1 has 336 unique site patterns
 Initializing conditional likelihoods
 Using standard non-SSE likelihood calculator for division 1 (single-precision)

 Initial log likelihoods and log prior probs for run 1:
    Chain 1 -- -100337.123130 -- -326.282876
    Chain 2 -- -98656.446447 -- -326.282876
    Chain 3 -- -95354.126426 -- -326.282876
    Chain 4 -- -99303.131451 -- -326.282876

 Initial log likelihoods and log prior probs for run 2:
    Chain 1 -- -101624.184772 -- -326.282876
    Chain 2 -- -99273.283493 -- -326.282876
    Chain 3 -- -99216.246530 -- -326.282876
    Chain 4 -- -98804.553615 -- -326.282876


 Using a relative burnin of 25.0 % for diagnostics

Chain results (1000000 generations requested):

    0 -- [-100337.123] (-98656.446) (-95354.126) (-99303.131) * [-101624.185] (-99273.283) (-99216.247) (-98804.554)
  500 -- (-76079.549) (-74756.608) [-69722.762] (-77597.318) * [-71391.180] (-73689.689) (-75133.148) (-72921.178) -- 10:33:01
 1000 -- (-59918.974) (-58862.674) [-55333.571] (-61856.405) * (-57083.811) (-56805.983) (-55539.747) [-53893.312] -- 10:16:03
 1500 -- (-50123.307) [-47597.865] (-47894.361) (-50767.879) * (-46853.779) (-48664.701) [-45337.903] (-45621.931) -- 12:12:14
 2000 -- (-43487.255) (-42961.693) [-41511.751] (-42238.992) * (-39969.492) (-41158.081) [-38298.402] (-40870.138) -- 11:30:17
 2500 -- [-33813.726] (-40099.040) (-37107.456) (-38914.673) * (-35891.858) (-37451.359) [-33483.004] (-37279.273) -- 11:24:57
 3000 -- [-31038.816] (-36134.217) (-34566.992) (-34697.463) * (-34188.157) (-34012.835) [-31645.756] (-32008.981) -- 11:21:17
 3500 -- [-29203.196] (-33337.178) (-31942.543) (-32740.889) * (-31618.155) (-31136.717) (-29677.261) [-29326.265] -- 17:04:58
 4000 -- [-27740.877] (-29923.538) (-30645.752) (-31045.572) * (-30424.765) (-28891.710) [-27860.974] (-27899.851) -- 16:19:24
 4500 -- [-26544.345] (-27935.541) (-28877.304) (-29172.319) * (-28593.371) (-27453.323) (-27126.181) [-26612.200] -- 15:43:52
 5000 -- [-25887.752] (-27075.350) (-28223.995) (-27462.534) * (-27265.680) (-26630.886) [-25866.598] (-26094.593) -- 15:15:24

Average standard deviation of split frequencies: 0.096728

 5500 -- [-25460.360] (-26116.658) (-26942.240) (-26581.954) * (-26698.688) (-25884.951) [-25316.628] (-25282.910) -- 14:52:02
 6000 -- [-25197.010] (-25679.858) (-26375.174) (-26115.931) * (-25866.500) (-25348.414) (-25055.180) [-24886.920] -- 14:29:45
 6500 -- [-24834.658] (-25254.586) (-26126.558) (-25634.403) * (-25293.242) (-24957.651) [-24658.187] (-24663.740) -- 14:15:56
 7000 -- [-24527.854] (-24914.555) (-25768.800) (-25335.911) * (-24821.481) (-24737.873) [-24398.373] (-24424.433) -- 14:01:41
 7500 -- [-24290.520] (-24503.697) (-24979.619) (-25012.150) * (-24536.397) (-24471.252) [-24166.035] (-24265.436) -- 13:49:17
 8000 -- [-23974.207] (-24346.959) (-24714.387) (-24690.817) * (-24367.528) (-24352.364) [-24009.992] (-24114.704) -- 13:36:20
 8500 -- [-23840.788] (-24199.283) (-24539.082) (-24636.090) * (-24154.126) (-24223.660) [-23928.866] (-24029.526) -- 13:24:51
 9000 -- [-23746.926] (-24051.007) (-24287.372) (-24527.304) * (-24017.599) (-24041.053) [-23797.811] (-23953.643) -- 13:14:38
 9500 -- [-23689.827] (-23948.307) (-24146.959) (-24372.814) * (-23855.631) (-23950.561) [-23707.445] (-23869.552) -- 13:03:42
10000 -- [-23649.610] (-23860.551) (-24090.895) (-24305.035) * (-23757.466) (-23820.719) [-23603.724] (-23733.286) -- 12:53:51

Average standard deviation of split frequencies: 0.146933

10500 -- [-23569.224] (-23809.297) (-23969.477) (-24170.264) * (-23670.216) (-23762.559) [-23512.477] (-23671.180) -- 12:43:19
11000 -- [-23535.645] (-23794.611) (-23786.553) (-24078.001) * (-23614.370) (-23668.873) [-23448.725] (-23603.356) -- 12:36:44
11500 -- [-23473.689] (-23765.928) (-23680.600) (-24024.911) * (-23589.572) (-23594.839) [-23397.570] (-23554.109) -- 12:30:41
12000 -- [-23436.710] (-23699.380) (-23574.643) (-24011.282) * (-23573.423) (-23529.456) [-23386.568] (-23525.771) -- 12:25:07
12500 -- [-23440.409] (-23692.964) (-23514.650) (-23913.908) * (-23530.652) (-23498.369) [-23370.876] (-23499.989) -- 12:19:58
13000 -- [-23415.370] (-23669.410) (-23492.331) (-23759.177) * (-23506.737) (-23468.809) [-23351.945] (-23497.693) -- 12:15:11
13500 -- [-23352.798] (-23655.237) (-23450.594) (-23735.492) * (-23463.237) (-23438.666) [-23344.451] (-23510.857) -- 12:09:31
14000 -- [-23322.474] (-23646.184) (-23423.372) (-23677.621) * (-23428.926) (-23412.857) [-23317.048] (-23476.227) -- 12:05:24
14500 -- [-23322.853] (-23634.698) (-23405.328) (-23588.845) * (-23409.349) (-23409.173) [-23303.137] (-23465.176) -- 12:01:34
15000 -- [-23311.096] (-23600.081) (-23394.994) (-23580.165) * (-23400.358) (-23408.303) [-23295.107] (-23470.851) -- 11:56:51

Average standard deviation of split frequencies: 0.155555

15500 -- [-23295.231] (-23570.366) (-23386.001) (-23549.540) * (-23387.244) (-23387.936) [-23295.274] (-23450.236) -- 11:53:29
16000 -- [-23293.716] (-23584.550) (-23374.016) (-23530.793) * (-23408.073) (-23359.962) [-23299.852] (-23450.730) -- 11:50:19
16500 -- [-23297.623] (-23597.250) (-23371.978) (-23500.534) * (-23379.533) (-23342.845) [-23295.087] (-23436.922) -- 11:48:19
17000 -- [-23293.003] (-23599.385) (-23357.377) (-23507.345) * (-23383.816) (-23332.550) [-23292.312] (-23420.229) -- 11:44:29
17500 -- [-23286.719] (-23579.983) (-23366.523) (-23476.255) * (-23373.321) (-23319.606) [-23292.813] (-23396.242) -- 11:41:47
18000 -- [-23304.391] (-23561.843) (-23352.584) (-23466.369) * (-23347.056) (-23312.127) [-23285.216] (-23421.276) -- 11:38:18
18500 -- [-23298.806] (-23583.626) (-23348.459) (-23456.851) * (-23347.376) (-23318.628) [-23261.268] (-23425.029) -- 11:35:53
19000 -- [-23287.430] (-23580.502) (-23326.781) (-23449.971) * (-23329.938) (-23300.159) [-23257.599] (-23431.766) -- 11:32:43
19500 -- [-23295.926] (-23563.952) (-23328.850) (-23441.129) * (-23336.412) (-23304.337) [-23254.383] (-23431.909) -- 11:29:42
20000 -- [-23280.191] (-23578.103) (-23328.679) (-23430.000) * (-23327.787) (-23300.619) [-23249.808] (-23425.515) -- 11:26:49

Average standard deviation of split frequencies: 0.148075
```

```
20500 -- [-23270.789] (-23554.286) (-23331.474) (-23437.792) * (-23330.768) (-23320.304) [-23261.445] (-23423.357) -- 11:23:15
21000 -- [-23273.115] (-23545.294) (-23326.745) (-23437.293) * (-23307.173) (-23294.875) [-23246.149] (-23393.031) -- 11:21:24
21500 -- [-23268.072] (-23539.629) (-23328.656) (-23452.648) * (-23318.085) (-23285.453) [-23264.146] (-23387.488) -- 11:18:52
22000 -- [-23264.434] (-23538.124) (-23328.495) (-23448.693) * (-23329.490) (-23269.082) [-23275.839] (-23380.972) -- 11:17:11
22500 -- [-23254.883] (-23544.442) (-23313.917) (-23443.991) * (-23324.461) (-23285.982) [-23263.380] (-23376.915) -- 11:14:50
23000 -- [-23242.307] (-23533.523) (-23322.114) (-23433.505) * (-23348.397) (-23286.405) [-23250.951] (-23401.039) -- 11:11:51
23500 -- [-23249.441] (-23536.160) (-23331.105) (-23443.102) * (-23336.567) (-23273.924) [-23262.222] (-23382.438) -- 11:09:41
24000 -- [-23242.450] (-23524.887) (-23322.053) (-23446.348) * (-23325.670) [-23265.959] [-23282.044] (-23392.387) -- 11:06:56
24500 -- [-23224.513] (-23518.631) (-23358.754) (-23443.401) * (-23321.678) (-23266.203) [-23284.177] (-23388.667) -- 11:04:55
25000 -- [-23225.534] (-23529.130) (-23347.453) (-23424.855) * (-23332.033) [-23273.998] (-23271.335) (-23381.460) -- 11:02:21

Average standard deviation of split frequencies: 0.123784

25500 -- [-23224.229] (-23525.422) (-23343.522) (-23399.918) * (-23327.664) [-23252.878] (-23263.038) (-23402.089) -- 11:00:29
26000 -- [-23234.941] (-23555.049) (-23321.296) (-23413.102) * (-23333.199) [-23257.645] (-23252.264) (-23413.767) -- 10:58:41
26500 -- [-23242.817] (-23545.151) (-23315.938) (-23412.658) * (-23315.214) (-23255.011) [-23257.920] (-23386.255) -- 10:56:57
27000 -- [-23241.479] (-23547.844) (-23321.907) (-23400.910) * (-23317.684) [-23252.069] (-23255.633) (-23408.459) -- 10:55:16
27500 -- [-23245.409] (-23536.055) (-23333.495) (-23409.380) * (-23334.549) [-23246.137] (-23254.459) (-23404.292) -- 10:53:38
28000 -- [-23251.138] (-23539.397) (-23301.335) (-23433.031) * (-23318.265) [-23229.190] (-23251.969) (-23403.518) -- 10:52:03
28500 -- [-23251.142] (-23529.585) (-23300.833) (-23425.442) * (-23321.276) [-23254.312] (-23248.055) (-23409.329) -- 10:49:56
29000 -- [-23240.583] (-23518.762) (-23302.232) (-23406.482) * (-23314.939) (-23246.033) [-23233.583] (-23415.475) -- 10:48:26
29500 -- [-23257.771] (-23516.601) (-23308.363) (-23412.676) * (-23331.279) (-23251.800) [-23231.703] (-23395.759) -- 10:47:00
30000 -- [-23259.976] (-23508.167) (-23333.645) (-23402.765) * (-23312.126) [-23235.769] (-23232.195) (-23417.189) -- 10:45:35

Average standard deviation of split frequencies: 0.110779

30500 -- [-23250.545] (-23537.425) (-23323.950) (-23399.932) * (-23330.735) [-23240.293] (-23237.282) (-23380.284) -- 10:43:41
31000 -- [-23247.523] (-23545.233) (-23325.946) (-23403.828) * (-23293.823) [-23235.204] (-23243.263) (-23384.018) -- 10:42:21
31500 -- [-23245.406] (-23550.538) (-23321.617) (-23392.019) * (-23313.985) [-23240.047] (-23255.238) (-23385.136) -- 10:40:32
32000 -- [-23256.533] (-23548.124) (-23329.003) (-23386.871) * (-23303.586) [-23245.804] (-23271.572) (-23393.019) -- 10:38:46
32500 -- [-23254.801] (-23526.620) (-23328.981) (-23399.379) * (-23303.369) [-23241.201] (-23281.309) (-23391.421) -- 10:37:33
33000 -- [-23252.611] (-23526.176) (-23325.742) (-23393.672) * (-23313.974) [-23273.868] (-23280.903) (-23381.271) -- 10:35:52
33500 -- [-23258.012] (-23516.462) (-23323.902) (-23371.009) * (-23334.049) (-23262.696) [-23267.841] (-23370.926) -- 10:34:42
34000 -- [-23254.745] (-23524.067) (-23316.089) (-23371.322) * (-23320.121) [-23254.560] (-23268.247) (-23361.823) -- 10:33:34
34500 -- [-23260.769] (-23527.676) (-23300.725) (-23393.849) * (-23304.179) [-23266.675] (-23288.333) (-23369.394) -- 10:32:28
35000 -- [-23232.162] (-23503.986) (-23286.415) (-23384.584) * (-23297.726) [-23255.649] (-23282.221) (-23352.481) -- 10:30:55

Average standard deviation of split frequencies: 0.099419

35500 -- [-23242.743] (-23506.644) (-23296.047) (-23391.392) * (-23301.190) [-23244.405] (-23278.079) (-23364.889) -- 10:29:52
36000 -- [-23238.772] (-23506.581) (-23309.886) (-23398.989) * (-23315.293) [-23241.143] (-23283.733) (-23365.951) -- 10:28:23
36500 -- [-23252.028] (-23455.375) (-23316.547) (-23398.456) * (-23295.079) [-23252.326] (-23273.594) (-23365.781) -- 10:27:22
37000 -- [-23248.286] (-23467.225) (-23318.006) (-23400.824) * (-23296.284) [-23256.054] (-23285.729) (-23359.612) -- 10:25:57
37500 -- [-23249.853] (-23455.903) (-23308.728) (-23384.347) * (-23296.531) [-23242.977] (-23290.458) (-23383.119) -- 10:24:59
38000 -- [-23237.215] (-23471.774) (-23299.904) (-23378.176) * (-23303.022) [-23239.279] (-23300.333) (-23398.520) -- 10:24:02
38500 -- [-23244.219] (-23463.906) (-23323.040) (-23382.154) * (-23297.920) [-23245.082] (-23311.243) (-23390.570) -- 10:23:06
39000 -- [-23254.059] (-23474.669) (-23307.714) (-23386.132) * (-23311.572) [-23244.394] (-23305.082) (-23386.144) -- 10:22:11
39500 -- [-23235.958] (-23443.901) (-23307.312) (-23370.469) * (-23304.808) [-23244.185] (-23293.320) (-23391.041) -- 10:21:17
40000 -- [-23240.706] (-23449.546) (-23314.117) (-23357.246) * [-23284.259] (-23265.392) (-23301.930) (-23396.850) -- 10:20:24

Average standard deviation of split frequencies: 0.093195

40500 -- [-23244.863] (-23447.577) (-23313.008) (-23359.198) * (-23285.876) [-23267.358] (-23319.255) (-23395.424) -- 10:19:31
41000 -- [-23255.715] (-23454.976) (-23300.254) (-23374.128) * [-23259.922] (-23271.837) (-23302.551) (-23389.744) -- 10:18:40
41500 -- [-23275.054] (-23481.370) (-23314.853) (-23384.178) * [-23268.662] (-23284.715) (-23292.403) (-23389.803) -- 10:17:49
42000 -- (-23285.379) (-23485.774) [-23308.981] (-23385.154) * [-23284.003] (-23292.060) (-23298.263) (-23407.771) -- 10:16:59
42500 -- [-23274.839] (-23505.481) (-23301.716) (-23386.400) * [-23267.061] (-23281.940) (-23306.817) (-23416.927) -- 10:16:10
43000 -- [-23279.999] (-23496.181) (-23302.617) (-23383.211) * [-23263.679] (-23265.452) (-23298.603) (-23406.191) -- 10:15:22
43500 -- [-23262.934] (-23507.012) (-23290.777) (-23364.668) * [-23266.764] (-23271.176) (-23277.723) (-23391.226) -- 10:14:34
44000 -- [-23263.830] (-23506.114) (-23273.552) (-23366.612) * (-23271.192) [-23250.157] (-23279.320) (-23397.702) -- 10:13:47
44500 -- [-23257.834] (-23481.619) (-23292.243) (-23386.490) * (-23244.578) [-23229.986] (-23282.794) (-23394.669) -- 10:13:01
45000 -- [-23246.921] (-23477.191) (-23312.009) (-23389.989) * (-23266.882) [-23241.783] (-23274.595) (-23390.976) -- 10:12:36

Average standard deviation of split frequencies: 0.087227

45500 -- [-23249.112] (-23477.350) (-23302.680) (-23389.973) * (-23271.820) [-23240.330] (-23285.185) (-23394.573) -- 10:11:51
46000 -- [-23249.127] (-23495.516) (-23299.341) (-23393.762) * [-23253.567] (-23262.467) (-23289.949) (-23362.297) -- 10:11:27
46500 -- [-23270.313] (-23491.127) (-23308.471) (-23405.646) * (-23253.436) [-23251.169] (-23268.283) (-23377.527) -- 10:10:43
47000 -- [-23268.080] (-23500.808) (-23296.771) (-23391.463) * [-23244.592] (-23282.008) (-23256.219) (-23384.371) -- 10:10:19
47500 -- [-23253.990] (-23476.544) (-23294.022) (-23381.498) * [-23245.410] (-23269.903) (-23252.554) (-23390.913) -- 10:09:36
48000 -- [-23249.232] (-23465.697) (-23310.851) (-23375.466) * [-23248.896] (-23289.947) (-23269.027) (-23397.914) -- 10:09:12
48500 -- [-23243.445] (-23469.197) (-23315.655) (-23381.527) * [-23241.833] (-23289.277) (-23279.366) (-23400.828) -- 10:08:30
49000 -- [-23244.954] (-23460.050) (-23309.472) (-23390.917) * [-23230.164] (-23290.636) (-23282.076) (-23387.555) -- 10:07:47
49500 -- [-23233.676] (-23461.945) (-23309.230) (-23402.870) * [-23229.071] (-23285.345) (-23282.192) (-23379.098) -- 10:07:06
50000 -- [-23245.922] (-23471.850) (-23307.579) (-23408.055) * [-23233.776] (-23280.824) (-23280.911) (-23380.011) -- 10:06:25

Average standard deviation of split frequencies: 0.082068

50500 -- [-23245.155] (-23480.011) (-23300.894) (-23397.201) * [-23239.327] (-23287.326) (-23285.064) (-23394.698) -- 10:05:44
51000 -- [-23241.973] (-23475.168) (-23318.578) (-23398.622) * [-23257.350] (-23275.147) (-23276.788) (-23385.153) -- 10:04:45
51500 -- [-23213.546] (-23480.431) (-23318.057) (-23405.723) * [-23268.379] (-23274.627) (-23291.933) (-23370.941) -- 10:04:05
52000 -- [-23215.870] (-23446.997) (-23331.281) (-23398.369) * [-23250.741] (-23300.727) (-23276.926) (-23385.180) -- 10:03:26
52500 -- [-23243.085] (-23457.055) (-23330.281) (-23397.527) * [-23253.047] (-23288.028) (-23277.377) (-23359.842) -- 10:02:47
53000 -- [-23244.841] (-23470.065) (-23340.728) (-23380.201) * [-23233.012] (-23292.515) (-23284.877) (-23341.173) -- 10:02:08
53500 -- [-23234.334] (-23469.542) (-23331.015) (-23389.311) * [-23235.287] (-23279.281) (-23277.449) (-23340.306) -- 10:01:48
54000 -- [-23229.933] (-23454.248) (-23325.551) (-23383.559) * [-23218.288] (-23288.319) (-23283.698) (-23358.247) -- 10:01:10
54500 -- [-23234.696] (-23457.093) (-23324.141) (-23359.856) * [-23215.869] (-23300.521) (-23287.969) (-23355.875) -- 10:00:33
55000 -- [-23237.908] (-23433.354) (-23334.237) (-23379.073) * [-23212.622] (-23297.752) (-23264.596) (-23350.051) -- 9:59:55

Average standard deviation of split frequencies: 0.074835

55500 -- [-23255.969] (-23459.731) (-23309.444) (-23373.452) * [-23224.926] (-23280.372) (-23257.377) (-23350.613) -- 9:59:36
56000 -- [-23234.104] (-23470.843) (-23302.028) (-23372.252) * (-23225.430) (-23299.044) [-23242.412] (-23347.731) -- 9:58:59
56500 -- [-23241.169] (-23463.917) (-23302.799) (-23357.834) * [-23229.193] (-23314.495) [-23255.031] (-23366.731) -- 9:58:39
57000 -- [-23221.859] (-23470.459) (-23306.316) (-23361.263) * [-23240.431] (-23311.605) (-23269.223) (-23374.128) -- 9:58:03
57500 -- [-23227.505] (-23438.564) (-23300.750) (-23381.562) * [-23230.070] (-23304.874) (-23260.956) (-23357.845) -- 9:57:27
58000 -- [-23213.068] (-23421.613) (-23312.014) (-23392.349) * [-23222.496] (-23276.266) (-23263.185) (-23352.854) -- 9:57:08
58500 -- [-23217.823] (-23423.165) (-23305.962) (-23391.803) * [-23217.785] (-23261.476) (-23289.988) (-23356.619) -- 9:56:33
59000 -- [-23234.307] (-23443.182) (-23289.747) (-23395.816) * [-23216.011] (-23257.145) (-23285.095) (-23342.903) -- 9:56:13
59500 -- [-23240.393] (-23457.176) (-23291.471) (-23393.380) * [-23216.191] (-23266.687) (-23291.049) (-23366.241) -- 9:55:39
60000 -- [-23232.874] (-23453.740) (-23296.112) (-23393.821) * [-23232.557] (-23283.688) (-23310.534) (-23348.985) -- 9:55:20

Average standard deviation of split frequencies: 0.070203

60500 -- [-23211.161] (-23450.972) (-23263.162) (-23407.330) * [-23215.061] (-23289.581) (-23317.502) (-23368.880) -- 9:54:45
61000 -- [-23214.833] (-23453.762) (-23270.834) (-23366.690) * [-23210.514] (-23288.590) (-23315.777) (-23368.422) -- 9:53:55
61500 -- [-23221.792] (-23465.205) (-23269.502) (-23374.145) * [-23216.007] (-23283.577) (-23327.576) (-23386.358) -- 9:53:21
```

```
62000 -- [-23214.034] (-23454.492) (-23278.415) (-23377.180) * [-23209.740] (-23286.331) (-23337.517) (-23382.663) -- 9:52:48
62500 -- [-23218.369] (-23439.341) (-23276.714) (-23376.384) * [-23220.001] (-23296.053) (-23329.812) (-23394.158) -- 9:52:00
63000 -- [-23225.481] (-23446.847) (-23282.327) (-23392.713) * [-23225.304] (-23275.667) (-23330.337) (-23367.320) -- 9:50:57
63500 -- [-23229.048] (-23450.298) (-23289.572) (-23380.216) * [-23238.250] (-23282.270) (-23324.132) (-23362.681) -- 9:50:24
64000 -- [-23221.827] (-23436.369) (-23289.447) (-23377.284) * [-23240.643] (-23288.457) (-23326.350) (-23363.940) -- 9:49:37
64500 -- [-23229.762] (-23437.571) (-23296.924) (-23378.350) * [-23227.967] (-23278.556) (-23315.441) (-23389.224) -- 9:49:05
65000 -- [-23223.546] (-23414.538) (-23277.147) (-23360.887) * [-23232.388] (-23260.257) (-23303.579) (-23412.394) -- 9:48:34

Average standard deviation of split frequencies: 0.066035

65500 -- [-23217.798] (-23425.979) (-23284.461) (-23367.901) * [-23238.513] (-23251.955) (-23301.547) (-23401.192) -- 9:48:02
66000 -- [-23231.138] (-23426.289) (-23286.045) (-23373.514) * [-23249.888] [-23241.714] (-23296.606) (-23407.695) -- 9:47:31
66500 -- [-23231.189] (-23429.191) (-23287.262) (-23373.581) * (-23228.092) [-23234.081] (-23291.601) (-23428.074) -- 9:47:00
67000 -- [-23227.977] (-23453.323) (-23290.068) (-23393.906) * (-23227.203) [-23244.654] (-23273.612) (-23410.710) -- 9:46:29
67500 -- [-23238.690] (-23455.077) (-23320.121) (-23414.379) * (-23250.828) [-23236.728] (-23283.396) (-23407.884) -- 9:45:58
68000 -- [-23230.549] (-23453.695) (-23300.289) (-23415.080) * (-23256.939) [-23227.991] (-23283.368) (-23395.635) -- 9:45:28
68500 -- [-23216.938] (-23457.394) (-23320.798) (-23408.372) * (-23264.222) [-23208.474] (-23280.423) (-23390.494) -- 9:44:57
69000 -- [-23228.025] (-23438.479) (-23301.979) (-23401.809) * (-23279.951) [-23209.671] (-23300.536) (-23399.442) -- 9:44:27
69500 -- [-23229.198] (-23416.608) (-23292.415) (-23396.814) * (-23282.822) [-23244.228] (-23298.225) (-23388.159) -- 9:43:44
70000 -- [-23221.205] (-23407.645) (-23289.626) (-23400.511) * (-23271.031) [-23242.155] (-23306.552) (-23374.811) -- 9:43:14

Average standard deviation of split frequencies: 0.065922

70500 -- [-23232.337] (-23379.339) (-23313.715) (-23379.085) * (-23296.158) [-23247.283] (-23297.286) (-23393.864) -- 9:42:45
71000 -- [-23239.113] (-23393.063) (-23318.046) (-23378.814) * (-23283.135) [-23251.157] (-23294.463) (-23384.736) -- 9:42:15
71500 -- [-23228.969] (-23414.623) (-23315.204) (-23385.286) * (-23283.249) [-23258.743] (-23288.954) (-23389.304) -- 9:41:46
72000 -- [-23221.418] (-23407.331) (-23321.344) (-23378.648) * (-23288.084) [-23238.408] (-23274.272) (-23382.525) -- 9:41:04
72500 -- [-23225.332] (-23419.821) (-23323.832) (-23351.438) * (-23292.066) (-23255.123) [-23243.786] (-23371.285) -- 9:40:48
73000 -- [-23235.954] (-23444.762) (-23329.182) (-23349.603) * (-23290.888) (-23245.280) [-23250.532] (-23370.510) -- 9:40:19
73500 -- [-23231.168] (-23427.276) (-23304.634) (-23369.704) * (-23292.527) (-23264.533) [-23243.580] (-23367.615) -- 9:39:51
74000 -- [-23253.396] (-23415.782) (-23334.212) (-23349.663) * (-23284.397) [-23268.078] (-23265.879) (-23388.414) -- 9:39:22
74500 -- [-23248.661] (-23387.017) (-23317.504) (-23376.421) * (-23287.949) [-23259.114] (-23260.738) (-23362.162) -- 9:38:54
75000 -- [-23248.807] (-23342.046) (-23316.670) (-23386.784) * (-23300.535) (-23258.793) [-23250.132] (-23354.619) -- 9:38:38

Average standard deviation of split frequencies: 0.064068

75500 -- [-23270.025] (-23351.820) (-23297.451) (-23401.045) * (-23290.431) (-23268.126) [-23262.729] (-23347.241) -- 9:38:22
76000 -- [-23271.627] (-23304.282) (-23301.752) (-23413.448) * (-23285.195) [-23255.492] (-23259.860) (-23354.541) -- 9:37:54
76500 -- [-23265.335] (-23267.306) (-23318.870) (-23419.897) * (-23271.625) [-23264.724] (-23265.660) (-23360.028) -- 9:37:38
77000 -- (-23285.073) [-23267.616] (-23314.074) (-23418.843) * [-23263.558] (-23256.257) (-23266.900) (-23353.065) -- 9:37:10
77500 -- (-23280.922) [-23264.715] (-23322.283) (-23409.971) * (-23269.123) [-23251.660] (-23281.452) (-23368.682) -- 9:36:42
78000 -- [-23258.305] (-23272.007) (-23307.376) (-23392.354) * [-23255.539] (-23262.090) (-23266.633) (-23370.667) -- 9:36:15
78500 -- [-23244.878] (-23260.581) (-23300.069) (-23398.308) * (-23266.236) [-23256.232] (-23275.714) (-23359.749) -- 9:35:35
79000 -- [-23261.536] (-23270.567) (-23296.923) (-23381.134) * (-23297.352) [-23251.818] (-23265.392) (-23357.049) -- 9:35:08
79500 -- [-23259.485] (-23263.475) (-23303.398) (-23400.745) * (-23290.151) [-23252.594] [-23255.504] (-23329.866) -- 9:34:41
80000 -- [-23255.773] (-23258.759) (-23317.181) (-23389.560) * (-23315.178) [-23241.289] (-23262.102) (-23333.167) -- 9:34:14

Average standard deviation of split frequencies: 0.061816

80500 -- [-23256.682] (-23265.333) (-23329.564) (-23402.691) * (-23287.089) [-23233.341] (-23263.363) (-23342.995) -- 9:33:58
81000 -- [-23259.894] (-23271.289) (-23329.029) (-23409.769) * (-23281.610) [-23232.194] (-23260.043) (-23364.830) -- 9:33:31
81500 -- [-23258.094] (-23255.998) (-23359.048) (-23432.939) * (-23273.056) (-23255.359) [-23254.905] (-23371.758) -- 9:33:15
82000 -- [-23255.547] (-23247.426) (-23359.773) (-23409.446) * (-23283.391) [-23254.633] (-23261.338) (-23378.355) -- 9:32:49
82500 -- [-23257.367] (-23255.800) (-23339.632) (-23394.503) * (-23275.722) (-23262.862) [-23253.460] (-23382.309) -- 9:32:22
83000 -- [-23253.891] (-23251.194) (-23329.237) (-23380.774) * (-23286.766) (-23277.547) [-23272.180] (-23387.862) -- 9:32:06
83500 -- [-23243.934] (-23268.368) (-23344.570) (-23380.564) * (-23298.265) [-23258.672] [-23268.368] (-23385.773) -- 9:31:40
84000 -- [-23233.395] (-23264.790) (-23352.938) (-23366.970) * (-23281.450) [-23250.621] (-23263.859) (-23393.568) -- 9:31:13
84500 -- [-23234.869] (-23260.361) (-23345.526) (-23378.207) * (-23279.688) [-23255.413] (-23267.422) (-23383.113) -- 9:30:47
85000 -- (-23256.664) [-23259.985] (-23310.362) (-23399.434) * (-23305.452) [-23254.787] (-23273.489) (-23407.317) -- 9:30:31

Average standard deviation of split frequencies: 0.060088

85500 -- [-23247.469] (-23257.673) (-23317.931) (-23388.014) * (-23296.865) [-23247.109] (-23268.981) (-23420.630) -- 9:29:54
86000 -- [-23245.347] (-23255.683) (-23306.687) (-23384.386) * (-23296.121) [-23255.628] (-23250.953) (-23405.435) -- 9:29:28
86500 -- [-23249.636] (-23257.145) (-23325.667) (-23382.208) * (-23292.104) (-23264.162) [-23255.386] (-23397.660) -- 9:29:02
87000 -- [-23254.767] (-23245.814) (-23317.894) (-23384.886) * (-23298.078) (-23267.219) [-23262.094] (-23393.903) -- 9:28:36
87500 -- [-23258.221] (-23253.174) (-23298.455) (-23384.752) * (-23302.069) (-23283.578) [-23252.326] (-23372.649) -- 9:28:11
88000 -- (-23238.348) [-23254.322] (-23328.973) (-23415.808) * (-23301.796) (-23284.008) [-23258.689] (-23389.281) -- 9:27:45
88500 -- (-23254.443) [-23250.806] (-23335.864) (-23407.255) * (-23293.703) (-23290.016) [-23260.901] (-23363.289) -- 9:27:19
89000 -- (-23269.856) [-23245.286] (-23351.587) (-23407.814) * [-23253.097] (-23300.241) (-23261.412) (-23376.169) -- 9:26:54
89500 -- (-23272.568) [-23250.312] (-23349.607) (-23391.661) * (-23253.836) (-23295.579) [-23246.727] (-23402.402) -- 9:26:38
90000 -- (-23253.859) [-23242.923] (-23343.736) (-23408.652) * (-23268.086) (-23304.084) [-23234.731] (-23390.744) -- 9:26:23

Average standard deviation of split frequencies: 0.056611

90500 -- [-23248.789] (-23253.943) (-23337.663) (-23417.964) * (-23273.752) (-23295.690) [-23237.417] (-23394.580) -- 9:25:58
91000 -- [-23261.750] (-23257.397) (-23323.027) (-23401.860) * (-23255.936) (-23286.526) [-23234.893] (-23389.425) -- 9:25:32
91500 -- [-23268.431] (-23258.451) (-23323.680) (-23384.947) * (-23252.652) (-23274.031) [-23236.407] (-23395.223) -- 9:25:17
92000 -- [-23269.509] (-23263.461) (-23324.399) (-23387.497) * (-23250.409) (-23280.143) [-23249.493] (-23395.815) -- 9:24:52
92500 -- [-23270.448] (-23261.636) (-23320.275) (-23410.024) * (-23250.463) (-23294.030) [-23243.602] (-23393.853) -- 9:24:26
93000 -- (-23272.247) [-23253.138] (-23326.913) (-23404.590) * [-23255.227] (-23293.732) (-23271.340) (-23399.946) -- 9:24:11
93500 -- (-23277.668) [-23250.455] (-23342.438) (-23390.690) * [-23254.600] (-23290.573) (-23275.190) (-23386.848) -- 9:23:56
94000 -- (-23264.637) [-23257.474] (-23338.280) (-23399.349) * [-23246.010] (-23280.438) [-23272.390] (-23364.447) -- 9:23:40
94500 -- (-23285.487) [-23248.095] (-23329.096) (-23361.360) * (-23263.329) (-23263.248) [-23256.787] (-23367.619) -- 9:23:25
95000 -- (-23279.357) [-23254.517] (-23330.002) (-23366.307) * (-23252.118) (-23272.718) [-23257.741] (-23359.477) -- 9:23:09

Average standard deviation of split frequencies: 0.054643

95500 -- (-23277.055) [-23240.482] (-23342.751) (-23373.604) * (-23266.388) (-23284.916) [-23262.225] (-23365.091) -- 9:22:44
96000 -- (-23262.235) [-23243.464] (-23354.918) (-23353.057) * (-23281.392) (-23262.479) [-23240.636] (-23369.322) -- 9:22:29
96500 -- (-23247.834) [-23244.269] (-23337.517) (-23322.205) * (-23293.471) (-23276.385) [-23244.309] (-23352.447) -- 9:22:04
97000 -- (-23253.474) [-23261.331] (-23350.793) (-23327.271) * (-23294.620) (-23282.594) [-23230.680] (-23364.752) -- 9:21:39
97500 -- [-23241.114] (-23252.067) (-23355.915) (-23331.945) * (-23279.253) (-23254.313) [-23238.566] (-23366.728) -- 9:21:24
98000 -- (-23248.266) [-23247.271] (-23362.310) (-23335.675) * (-23293.316) [-23244.744] (-23245.872) (-23363.619) -- 9:21:08
98500 -- (-23239.059) [-23227.216] (-23349.470) (-23330.950) * (-23295.935) (-23254.517) [-23233.414] (-23376.226) -- 9:20:43
99000 -- (-23257.018) [-23231.985] (-23343.092) (-23327.147) * (-23275.678) (-23255.895) [-23241.342] (-23359.248) -- 9:20:28
99500 -- (-23237.092) [-23237.494] (-23351.163) (-23303.788) * (-23281.595) (-23273.979) [-23240.861] (-23367.414) -- 9:20:12
100000 -- (-23235.283) [-23228.297] (-23332.940) (-23296.122) * (-23274.875) (-23256.879) [-23220.407] (-23361.855) -- 9:19:48

Average standard deviation of split frequencies: 0.054997

100500 -- (-23254.201) [-23238.147] (-23326.724) (-23312.910) * (-23282.189) (-23271.868) [-23209.330] (-23347.834) -- 9:19:32
101000 -- [-23265.977] (-23242.990) (-23348.082) (-23316.523) * (-23259.339) (-23284.877) [-23214.042] (-23362.135) -- 9:19:16
101500 -- (-23268.984) [-23246.111] (-23348.004) (-23342.222) * (-23245.123) (-23301.031) [-23220.001] (-23373.818) -- 9:18:52
102000 -- (-23250.610) [-23248.686] (-23336.379) (-23327.139) * (-23250.288) (-23288.838) [-23223.016] (-23395.035) -- 9:18:27
102500 -- (-23254.254) [-23244.180] (-23328.023) (-23336.565) * [-23236.556] (-23280.433) (-23239.172) (-23386.758) -- 9:18:03
103000 -- (-23248.302) [-23239.996] (-23353.520) (-23335.875) * [-23243.991] (-23273.234) (-23251.336) (-23377.527) -- 9:17:47
103500 -- (-23255.910) [-23241.219] (-23383.910) (-23334.307) * [-23247.644] (-23263.188) (-23256.933) (-23373.673) -- 9:17:32
```

```
104000 -- (-23239.314) [-23230.408] (-23356.650) (-23340.581) * (-23279.035) [-23246.892] (-23255.541) (-23387.056) -- 9:17:16
104500 -- [-23236.182] (-23250.727) (-23349.382) (-23350.459) * (-23279.818) (-23256.116) [-23240.709] (-23391.769) -- 9:16:52
105000 -- (-23260.357) [-23256.899] (-23351.777) (-23341.224) * (-23276.760) (-23262.405) [-23255.268] (-23405.430) -- 9:16:36

Average standard deviation of split frequencies: 0.053950

105500 -- (-23250.185) [-23246.242] (-23323.963) (-23325.927) * (-23275.241) (-23280.348) [-23276.580] (-23389.563) -- 9:16:12
106000 -- (-23264.102) [-23255.343] (-23347.892) (-23324.525) * (-23279.367) (-23293.333) [-23285.608] (-23385.961) -- 9:15:56
106500 -- (-23269.180) [-23232.860] (-23337.822) (-23318.317) * (-23277.572) (-23276.933) [-23270.044] (-23397.419) -- 9:15:40
107000 -- (-23269.642) [-23241.014] (-23318.947) (-23312.204) * (-23268.923) (-23263.757) (-23264.878) (-23394.330) -- 9:15:16
107500 -- [-23249.427] (-23260.298) (-23353.835) (-23308.459) * (-23284.313) (-23261.003) [-23255.792] (-23372.069) -- 9:14:52
108000 -- [-23236.764] (-23269.949) (-23348.581) (-23308.245) * (-23304.200) (-23254.051) [-23254.877] (-23385.999) -- 9:14:28
108500 -- [-23233.400] (-23282.704) (-23334.740) (-23328.215) * (-23288.875) (-23266.546) [-23265.488] (-23401.175) -- 9:14:04
109000 -- [-23228.578] (-23271.636) (-23327.480) (-23341.635) * (-23271.751) [-23256.572] (-23267.833) (-23370.839) -- 9:13:48
109500 -- [-23231.618] (-23258.170) (-23343.920) (-23311.637) * [-23287.231] (-23254.769) (-23273.745) (-23387.351) -- 9:13:24
110000 -- [-23232.022] (-23266.829) (-23329.219) (-23308.913) * (-23259.451) [-23253.438] (-23253.222) (-23399.084) -- 9:13:00

Average standard deviation of split frequencies: 0.052258

110500 -- [-23229.755] (-23286.477) (-23317.878) (-23299.053) * (-23258.672) (-23244.494) [-23263.669] (-23391.312) -- 9:12:37
111000 -- [-23231.038] (-23271.354) (-23316.979) (-23292.474) * (-23261.558) [-23238.690] (-23258.680) (-23387.383) -- 9:12:13
111500 -- [-23236.247] (-23251.455) (-23319.025) (-23297.175) * (-23257.984) [-23244.180] (-23262.883) (-23385.484) -- 9:11:49
112000 -- [-23236.736] (-23259.472) (-23308.174) (-23296.337) * (-23275.949) [-23258.101] (-23272.091) (-23357.095) -- 9:11:25
112500 -- [-23235.948] (-23267.718) (-23322.278) (-23308.259) * (-23282.776) [-23246.574] (-23270.650) (-23369.964) -- 9:11:10
113000 -- (-23260.077) [-23247.120] (-23339.917) (-23332.648) * (-23293.490) [-23237.521] (-23286.097) (-23384.841) -- 9:10:46
113500 -- [-23262.009] (-23269.633) (-23323.192) (-23309.316) * (-23289.094) [-23246.048] (-23267.254) (-23392.535) -- 9:10:23
114000 -- (-23255.298) [-23273.978] (-23308.494) (-23298.459) * (-23282.060) (-23266.666) [-23257.772] (-23359.492) -- 9:09:59
114500 -- (-23259.602) [-23267.507] (-23334.412) (-23301.013) * (-23300.424) (-23275.029) [-23252.843] (-23367.669) -- 9:09:28
115000 -- (-23257.978) [-23261.216] (-23335.810) (-23303.567) * (-23312.690) [-23269.068] (-23255.666) (-23356.518) -- 9:09:05

Average standard deviation of split frequencies: 0.050855

115500 -- (-23271.943) [-23240.486] (-23341.615) (-23313.567) * (-23324.591) (-23264.599) [-23252.210] (-23348.399) -- 9:08:41
116000 -- (-23267.527) [-23258.168] (-23335.819) (-23330.191) * (-23293.652) [-23258.861] (-23260.939) (-23353.939) -- 9:08:10
116500 -- (-23252.249) [-23259.364] (-23317.693) (-23315.823) * (-23291.646) [-23261.411] (-23266.181) (-23355.681) -- 9:07:47
117000 -- (-23241.246) [-23264.926] (-23318.863) (-23317.709) * (-23290.880) [-23254.196] (-23247.283) (-23382.281) -- 9:07:24
117500 -- [-23265.377] [-23252.899] (-23331.169) (-23333.515) * (-23279.425) (-23268.685) [-23249.158] (-23396.126) -- 9:07:01
118000 -- (-23264.210) [-23239.731] (-23328.119) (-23326.382) * (-23294.547) (-23253.929) [-23238.793] (-23384.452) -- 9:06:45
118500 -- (-23271.764) [-23239.820] (-23327.112) (-23328.131) * (-23302.536) (-23247.703) [-23255.627] (-23371.383) -- 9:06:22
119000 -- (-23266.813) [-23235.774] (-23347.529) (-23325.960) * (-23287.599) [-23247.336] (-23272.551) (-23370.191) -- 9:05:59
119500 -- (-23279.598) [-23244.056] (-23359.042) (-23332.529) * (-23281.007) [-23228.948] (-23286.328) (-23348.178) -- 9:05:29
120000 -- (-23281.579) [-23248.417] (-23339.080) (-23320.187) * [-23266.351] (-23249.717) (-23281.919) (-23348.719) -- 9:05:06

Average standard deviation of split frequencies: 0.050501

120500 -- (-23276.109) [-23241.496] (-23319.491) (-23326.435) * [-23271.691] (-23256.291) (-23302.915) (-23359.280) -- 9:04:51
121000 -- (-23270.261) [-23241.314] (-23312.310) (-23314.123) * [-23264.422] (-23253.317) (-23313.082) (-23382.142) -- 9:04:28
121500 -- (-23255.356) [-23251.990] (-23321.864) (-23310.224) * [-23259.234] (-23247.891) (-23304.742) (-23386.387) -- 9:04:05
122000 -- [-23259.800] (-23253.747) (-23319.832) (-23306.823) * (-23258.450) [-23224.171] (-23312.375) (-23381.638) -- 9:03:42
122500 -- (-23259.095) [-23249.827] (-23307.992) (-23328.237) * (-23261.044) [-23234.280] (-23281.651) (-23375.952) -- 9:03:20
123000 -- (-23263.613) [-23252.987] (-23337.479) (-23298.453) * (-23254.920) [-23242.305] (-23278.373) (-23378.780) -- 9:02:57
123500 -- (-23266.323) [-23248.228] (-23345.582) (-23294.736) * (-23270.035) [-23222.931] (-23295.411) (-23379.719) -- 9:02:27
124000 -- (-23277.280) [-23247.611] (-23331.276) (-23296.036) * (-23266.224) [-23224.719] (-23301.611) (-23355.824) -- 9:02:05
124500 -- (-23276.807) [-23242.444] (-23334.191) (-23328.560) * (-23255.548) [-23233.560] (-23300.356) (-23372.663) -- 9:01:42
125000 -- (-23266.173) [-23251.118] (-23344.455) (-23319.466) * [-23234.103] (-23235.018) (-23298.132) (-23378.620) -- 9:01:20

Average standard deviation of split frequencies: 0.050566

125500 -- (-23275.836) [-23250.967] (-23346.483) (-23296.649) * (-23256.164) [-23224.328] (-23288.151) (-23383.759) -- 9:00:57
126000 -- (-23256.230) [-23239.843] (-23351.871) (-23288.716) * (-23277.941) [-23229.413] (-23289.454) (-23360.592) -- 9:00:35
126500 -- (-23273.605) [-23247.964] (-23349.050) (-23276.160) * (-23268.076) [-23217.771] (-23282.190) (-23392.882) -- 9:00:12
127000 -- (-23263.272) [-23254.777] (-23375.618) (-23299.243) * (-23278.205) [-23229.065] (-23306.659) (-23384.314) -- 8:59:43
127500 -- (-23277.119) [-23247.118] (-23362.075) (-23316.967) * (-23250.996) [-23221.925] (-23299.560) (-23368.004) -- 8:59:21
128000 -- [-23256.561] (-23262.566) (-23340.747) (-23297.823) * (-23270.840) [-23210.672] (-23303.649) (-23375.960) -- 8:58:52
128500 -- (-23251.736) [-23244.967] (-23353.635) (-23281.623) * (-23258.176) [-23226.711] (-23290.475) (-23375.660) -- 8:58:23
129000 -- (-23263.655) [-23262.139] (-23347.579) (-23288.424) * (-23252.583) [-23234.848] (-23258.967) (-23370.139) -- 8:58:01
129500 -- (-23279.649) [-23248.255] (-23347.369) (-23297.944) * (-23271.514) [-23233.676] (-23248.866) (-23373.500) -- 8:57:25
130000 -- (-23285.841) [-23240.621] (-23344.886) (-23282.121) * (-23251.614) [-23228.639] (-23258.438) (-23357.775) -- 8:56:50

Average standard deviation of split frequencies: 0.049686

130500 -- (-23276.956) [-23238.773] (-23343.324) (-23275.187) * (-23252.962) [-23238.142] (-23262.229) (-23352.041) -- 8:56:21
131000 -- (-23262.552) [-23244.726] (-23316.310) (-23276.306) * (-23237.477) [-23234.202] (-23263.957) (-23360.682) -- 8:55:46
131500 -- (-23235.588) [-23239.312] (-23308.628) (-23291.261) * (-23253.555) [-23244.214] (-23247.156) (-23364.629) -- 8:55:17
132000 -- (-23248.270) [-23237.822] (-23315.286) (-23306.133) * (-23281.529) [-23240.650] (-23252.066) (-23376.851) -- 8:54:43
132500 -- (-23244.463) [-23245.868] (-23315.446) (-23289.050) * (-23300.048) [-23216.613] (-23250.174) (-23382.061) -- 8:54:14
133000 -- [-23245.115] (-23267.691) (-23302.847) (-23287.443) * (-23307.185) [-23232.154] (-23261.226) (-23397.118) -- 8:53:46
133500 -- [-23243.721] (-23239.112) (-23315.178) (-23287.776) * (-23309.124) [-23240.893] (-23263.767) (-23406.475) -- 8:53:12
134000 -- (-23254.462) [-23261.127] (-23310.625) (-23293.223) * (-23310.083) [-23230.600] (-23255.874) (-23400.370) -- 8:52:44
134500 -- (-23268.257) [-23266.233] (-23327.612) (-23306.962) * (-23313.966) [-23239.068] (-23257.099) (-23380.446) -- 8:52:16
135000 -- (-23280.511) [-23240.672] (-23336.329) (-23307.045) * (-23319.181) [-23253.587] (-23263.945) (-23385.671) -- 8:51:48

Average standard deviation of split frequencies: 0.047586

135500 -- (-23271.028) [-23251.735] (-23334.849) (-23308.845) * (-23318.403) (-23248.983) [-23234.862] (-23383.659) -- 8:51:21
136000 -- (-23255.714) [-23222.704] (-23309.253) (-23293.835) * (-23302.346) (-23266.981) [-23259.294] (-23406.895) -- 8:50:53
136500 -- (-23255.748) [-23221.970] (-23287.839) (-23296.379) * (-23293.612) [-23254.616] (-23274.358) (-23412.442) -- 8:50:26
137000 -- (-23259.242) (-23220.872) [-23271.495] (-23282.430) * (-23297.695) [-23245.299] (-23280.500) (-23432.054) -- 8:49:58
137500 -- (-23258.959) [-23211.439] (-23292.869) (-23310.697) * (-23317.632) [-23253.637] (-23275.850) (-23420.996) -- 8:49:31
138000 -- (-23256.093) [-23215.360] (-23300.542) (-23301.867) * (-23328.887) [-23243.270] (-23265.680) (-23398.466) -- 8:49:04
138500 -- [-23252.991] (-23225.872) (-23300.142) (-23322.619) * (-23302.245) (-23239.612) [-23249.052] (-23395.474) -- 8:48:36
139000 -- (-23255.358) [-23216.278] (-23294.840) (-23315.720) * (-23311.056) (-23256.167) [-23237.575] (-23390.053) -- 8:48:09
139500 -- (-23235.823) [-23230.401] (-23298.238) (-23324.713) * (-23275.895) (-23254.582) [-23227.134] (-23413.262) -- 8:47:42
140000 -- [-23229.616] (-23240.716) (-23273.321) (-23341.519) * (-23272.443) (-23251.615) [-23221.775] (-23400.991) -- 8:47:15

Average standard deviation of split frequencies: 0.045397

140500 -- [-23233.884] (-23239.779) (-23267.078) (-23306.442) * (-23263.374) (-23251.059) [-23224.397] (-23389.366) -- 8:46:54
141000 -- [-23231.479] (-23237.953) (-23289.879) (-23302.518) * (-23257.479) (-23286.888) [-23239.129] (-23396.269) -- 8:46:28
141500 -- [-23247.430] (-23254.455) (-23288.628) (-23291.908) * (-23270.158) [-23266.844] (-23249.363) (-23392.323) -- 8:46:01
142000 -- [-23252.149] (-23262.050) (-23285.245) (-23293.343) * (-23266.782) (-23259.975) [-23239.018] (-23393.975) -- 8:45:40
142500 -- [-23246.789] (-23283.717) (-23292.978) (-23287.355) * (-23263.860) [-23252.718] (-23253.467) (-23375.879) -- 8:45:13
143000 -- [-23243.929] (-23278.950) (-23304.731) (-23284.520) * (-23235.682) [-23237.797] (-23258.887) (-23385.265) -- 8:44:53
143500 -- [-23235.326] (-23264.300) (-23318.233) (-23301.164) * [-23242.225] (-23237.167) (-23265.252) (-23362.673) -- 8:44:26
144000 -- [-23236.007] (-23275.584) (-23303.163) (-23302.721) * (-23251.396) [-23230.316] (-23280.519) (-23343.358) -- 8:44:06
144500 -- [-23239.667] (-23263.949) (-23281.604) (-23297.921) * (-23251.966) [-23245.875] (-23289.480) (-23347.550) -- 8:43:45
145000 -- [-23251.422] (-23295.174) (-23282.760) (-23290.142) * (-23265.653) [-23265.392] (-23275.386) (-23342.793) -- 8:43:25
```

```
Average standard deviation of split frequencies: 0.043040

145500 -- [-23251.219] (-23302.715) (-23258.908) (-23312.537) * [-23261.471] (-23256.146) (-23282.144) (-23350.352) -- 8:42:58
146000 -- [-23265.244] (-23279.774) (-23255.931) (-23326.492) * [-23253.572) [-23264.427] (-23256.637) (-23351.642) -- 8:42:32
146500 -- (-23273.077) [-23269.896] (-23254.920) (-23340.088) * (-23253.498) [-23248.261] (-23262.402) (-23382.118) -- 8:42:11
147000 -- (-23276.032) [-23267.156] (-23268.062) (-23337.228) * (-23259.891) [-23266.182] (-23277.291) (-23380.280) -- 8:41:45
147500 -- [-23251.656] (-23258.337) (-23255.917) (-23344.754) * (-23279.145) [-23256.373] (-23255.774) (-23362.874) -- 8:41:19
148000 -- (-23253.201) (-23283.623) [-23235.349] (-23310.224) * (-23264.755) [-23250.239] (-23255.687) (-23365.695) -- 8:40:53
148500 -- (-23272.691) [-23267.075] (-23235.870) (-23300.015) * (-23275.617) [-23251.848] (-23251.177) (-23365.777) -- 8:40:27
149000 -- (-23283.094) (-23275.909) [-23219.403] (-23329.127) * (-23297.957) [-23249.179] (-23236.670) (-23365.110) -- 8:40:01
149500 -- (-23284.671) (-23268.778) [-23239.878] (-23322.404) * (-23308.033) (-23248.717) [-23232.067] (-23384.892) -- 8:39:35
150000 -- (-23284.540) (-23290.150) [-23231.366] (-23306.464) * (-23296.910) (-23262.842) [-23227.363] (-23383.196) -- 8:39:15

Average standard deviation of split frequencies: 0.042770

150500 -- (-23258.275) (-23294.767) [-23250.925] (-23310.336) * (-23304.971) (-23263.668) [-23229.520] (-23379.980) -- 8:38:49
151000 -- (-23246.833) (-23263.136) [-23262.693] (-23314.270) * (-23306.330) (-23271.117) [-23233.146] (-23377.883) -- 8:38:23
151500 -- (-23254.965) (-23259.075) [-23263.177] (-23307.755) * (-23305.025) (-23264.200) [-23232.083] (-23374.565) -- 8:37:58
152000 -- (-23268.632) [-23243.043] (-23256.806) (-23300.680) * (-23306.209) (-23254.014) [-23239.980] (-23368.370) -- 8:37:32
152500 -- (-23277.453) (-23250.182) [-23247.962] (-23293.284) * (-23308.499) [-23235.297] (-23237.113) (-23384.258) -- 8:37:06
153000 -- (-23271.679) [-23251.312] (-23251.559) (-23298.191) * (-23313.432) [-23219.483] (-23237.066) (-23360.261) -- 8:36:41
153500 -- (-23263.938) (-23229.018) [-23239.326] (-23303.700) * (-23318.794) [-23221.218] (-23244.207) (-23352.811) -- 8:36:21
154000 -- (-23270.127) [-23217.554] [-23247.840] (-23308.476) * (-23301.366) [-23211.638] (-23243.908) (-23367.919) -- 8:35:55
154500 -- (-23289.525) [-23234.157] (-23245.242) (-23304.432) * (-23305.614) [-23229.078] (-23226.773) (-23358.482) -- 8:35:30
155000 -- (-23283.426) (-23247.968) [-23246.827] (-23309.817) * (-23305.629) [-23250.474] (-23230.795) (-23361.076) -- 8:35:05

Average standard deviation of split frequencies: 0.042730

155500 -- (-23279.664) (-23263.338) [-23233.243] (-23310.112) * (-23308.535) (-23246.159) [-23247.744] (-23338.247) -- 8:34:39
156000 -- (-23264.661) [-23244.575] (-23226.441) (-23327.104) * (-23323.971) (-23253.943) [-23252.804] (-23347.069) -- 8:34:14
156500 -- (-23264.664) [-23257.298] (-23243.597) (-23322.852) * (-23319.277) (-23237.370) [-23238.921] (-23329.330) -- 8:33:54
157000 -- [-23247.546] (-23288.608) (-23232.638) (-23313.174) * (-23311.772) (-23274.831) [-23237.283] (-23319.708) -- 8:33:29
157500 -- (-23257.066) (-23292.038) [-23236.342] (-23304.520) * (-23279.997) (-23290.735) [-23262.650] (-23359.069) -- 8:33:04
158000 -- (-23261.958) (-23307.857) [-23237.876] (-23330.634) * (-23290.621) (-23283.405) [-23255.964] (-23338.578) -- 8:32:39
158500 -- (-23282.455) (-23319.069) [-23247.195] (-23308.729) * (-23298.920) (-23269.475) [-23252.216] (-23353.384) -- 8:32:14
159000 -- (-23287.894) (-23289.330) [-23235.392] (-23325.260) * (-23274.497) (-23282.205) [-23239.890] (-23372.908) -- 8:31:49
159500 -- (-23271.449) (-23288.033) [-23229.674] (-23300.575) * (-23277.700) (-23270.553) [-23228.199] (-23369.219) -- 8:31:24
160000 -- (-23269.790) (-23272.073) [-23245.001] (-23303.442) * (-23286.837) (-23258.079) [-23220.677] (-23363.454) -- 8:30:59

Average standard deviation of split frequencies: 0.042454

160500 -- (-23273.156) (-23260.959) [-23246.255] (-23295.393) * (-23253.442) (-23270.453) [-23214.650] (-23388.375) -- 8:30:40
161000 -- (-23280.587) (-23260.132) [-23248.071] (-23295.718) * (-23268.540) (-23272.445) [-23222.690] (-23378.719) -- 8:30:15
161500 -- (-23296.977) (-23242.624) [-23231.809] (-23290.794) * (-23276.932) (-23273.884) [-23225.244] (-23359.011) -- 8:29:45
162000 -- (-23289.553) (-23253.129) [-23240.277] (-23300.834) * (-23291.876) (-23258.301) [-23224.298] (-23343.770) -- 8:29:21
162500 -- (-23298.742) (-23264.267) [-23243.045] (-23325.483) * (-23284.765) (-23264.721) [-23237.315] (-23343.060) -- 8:28:56
163000 -- (-23305.356) (-23286.085) [-23232.736] (-23339.788) * (-23290.657) (-23278.810) [-23226.290] (-23321.277) -- 8:28:37
163500 -- (-23270.017) (-23281.012) [-23239.633] (-23348.461) * (-23277.219) (-23296.528) [-23249.181] (-23309.824) -- 8:28:17
164000 -- (-23273.293) (-23288.919) [-23239.170] (-23355.522) * (-23280.739) (-23276.698) [-23241.642] (-23309.805) -- 8:27:53
164500 -- (-23273.584) (-23295.499) [-23233.563] (-23336.409) * [-23285.756] (-23305.141) (-23241.275) (-23322.064) -- 8:27:33
165000 -- (-23269.445) (-23286.952) [-23242.548] (-23351.354) * (-23272.268) (-23287.712) [-23242.232] (-23327.363) -- 8:27:14

Average standard deviation of split frequencies: 0.041213

165500 -- (-23270.431) (-23289.094) [-23225.938] (-23347.204) * (-23272.452) (-23282.281) [-23242.808] (-23347.006) -- 8:26:55
166000 -- (-23259.490) (-23307.940) [-23249.390] (-23344.293) * (-23283.354) (-23265.986) [-23250.404] (-23351.364) -- 8:26:35
166500 -- (-23243.653) (-23281.799) [-23243.543] (-23320.488) * (-23293.050) (-23275.736) [-23266.268] (-23342.814) -- 8:26:16
167000 -- [-23232.871] (-23286.210) (-23246.895) (-23347.716) * (-23306.440) (-23269.457) [-23261.865] (-23350.614) -- 8:25:57
167500 -- (-23227.103] (-23257.752) (-23239.087) (-23319.073) * (-23298.935) (-23284.190) [-23261.394] (-23342.801) -- 8:25:37
168000 -- [-23231.329] (-23265.577) (-23233.941) (-23326.460) * (-23296.603) (-23281.394) [-23264.825] (-23352.194) -- 8:25:18
168500 -- [-23210.136] (-23271.664) (-23239.540) (-23329.870) * [-23286.545] (-23286.278) (-23263.711) (-23371.795) -- 8:24:54
169000 -- [-23223.495] (-23279.093) (-23243.802) (-23327.037) * [-23252.444] (-23264.213) (-23283.809) (-23383.278) -- 8:24:34
169500 -- [-23223.055] (-23261.822) (-23269.160) (-23327.040) * [-23239.655] (-23248.269) (-23275.279) (-23372.193) -- 8:24:10
170000 -- [-23229.166] (-23265.772) (-23286.768) (-23340.299) * (-23249.387) [-23235.976] (-23259.680) (-23352.682) -- 8:23:46

Average standard deviation of split frequencies: 0.039856

170500 -- [-23222.365] (-23263.912) (-23262.779) (-23331.624) * (-23245.093) [-23237.607] (-23281.413) (-23364.617) -- 8:23:27
171000 -- [-23232.929] (-23245.212) (-23268.618) (-23313.923) * [-23237.053] (-23231.946) (-23300.395) (-23370.903) -- 8:23:08
171500 -- [-23238.068] (-23252.068) (-23280.062) (-23330.361) * (-23254.851) [-23242.316] (-23306.543) (-23367.057) -- 8:22:48
172000 -- [-23241.740] (-23260.178) (-23265.213) (-23341.118) * (-23260.428) [-23248.819] (-23304.197) (-23364.387) -- 8:22:34
172500 -- [-23246.857] (-23263.344) (-23266.278) (-23353.904) * (-23259.536) [-23239.887] (-23314.157) (-23367.184) -- 8:22:15
173000 -- [-23230.908] (-23253.475) (-23258.785) (-23347.307) * [-23254.498] (-23233.420) (-23299.634) (-23359.805) -- 8:21:56
173500 -- [-23242.054] (-23241.551) (-23242.409) (-23324.168) * (-23259.939) [-23236.875] (-23292.738) (-23350.865) -- 8:21:36
174000 -- (-23240.718) (-23246.226) [-23250.622] (-23340.426) * (-23257.215) [-23242.589] (-23309.780) (-23367.939) -- 8:21:22
174500 -- (-23242.726) (-23275.212) [-23254.693] (-23346.892) * (-23249.502) [-23241.700] (-23299.548) (-23379.091) -- 8:21:03
175000 -- (-23240.810) (-23268.136) [-23258.500] (-23348.916) * (-23260.292) [-23261.815] (-23297.005) (-23348.120) -- 8:20:48

Average standard deviation of split frequencies: 0.038916

175500 -- [-23253.282] (-23278.560) (-23255.854) (-23331.735) * (-23261.320) [-23272.599] (-23315.637) (-23340.529) -- 8:20:39
176000 -- (-23272.510) (-23293.414) [-23257.565] (-23296.041) * (-23269.235) [-23278.756] (-23286.802) (-23333.772) -- 8:20:29
176500 -- [-23264.409] (-23285.301) (-23260.891) (-23285.700) * [-23261.280] (-23277.237) (-23309.570) (-23347.594) -- 8:20:14
177000 -- (-23286.818) (-23287.289) [-23261.798] (-23274.245) * (-23262.964) [-23285.748] (-23302.066) (-23335.768) -- 8:19:59
177500 -- (-23264.167) (-23269.191) [-23284.594] (-23273.922) * [-23276.368] (-23297.618) (-23302.908) (-23344.726) -- 8:19:45
178000 -- [-23266.627] (-23260.937) (-23306.878) (-23290.042) * [-23268.420] (-23304.944) (-23312.011) (-23349.026) -- 8:19:30
178500 -- [-23241.412] (-23265.204) (-23279.938) (-23283.534) * (-23275.949) [-23290.417] (-23315.090) (-23357.691) -- 8:19:15
179000 -- [-23230.637] (-23268.681) (-23280.938) (-23306.094) * [-23264.169] (-23294.585) (-23301.271) (-23354.105) -- 8:18:56
179500 -- [-23225.500] (-23265.854) (-23276.993) (-23319.863) * (-23272.574] (-23284.817) (-23296.869) (-23355.975) -- 8:18:37
180000 -- [-23232.046] (-23243.332) (-23266.768) (-23331.868) * [-23267.356] (-23266.585) (-23280.058) (-23360.294) -- 8:18:18

Average standard deviation of split frequencies: 0.038348

180500 -- (-23253.491) (-23254.773) [-23241.858] (-23322.734) * (-23271.710) [-23257.549] (-23277.574) (-23363.307) -- 8:17:58
181000 -- (-23257.580) (-23259.750) [-23251.892] (-23340.901) * [-23272.806] (-23295.596) (-23279.409) (-23340.351) -- 8:17:39
181500 -- (-23276.235) (-23277.261) [-23259.696] (-23351.332) * [-23254.771] (-23318.197) (-23270.634) (-23320.421) -- 8:17:24
182000 -- (-23288.346) (-23253.457) [-23249.748] (-23336.607) * [-23253.984] (-23325.116) (-23268.204) (-23323.019) -- 8:17:05
182500 -- (-23266.569) [-23248.161] (-23270.149) (-23311.900) * (-23271.831) (-23312.141) [-23259.174] (-23339.468) -- 8:16:46
183000 -- (-23263.427) [-23253.817] (-23292.458) (-23334.273) * (-23284.727) (-23310.316) [-23249.498] (-23337.797) -- 8:16:27
183500 -- (-23277.996) [-23255.102] (-23294.734) (-23322.479) * (-23272.936) (-23294.562) [-23252.198] (-23337.927) -- 8:16:12
184000 -- (-23262.642) [-23244.410] (-23299.734) (-23313.549) * (-23296.109) (-23307.254) [-23250.186] (-23347.817) -- 8:15:57
184500 -- (-23281.068) [-23246.544] (-23284.046) (-23302.428) * (-23305.667) (-23292.016) [-23255.315] (-23352.816) -- 8:15:38
185000 -- (-23291.728) [-23241.545] (-23291.737) (-23290.384) * (-23289.470) (-23287.577) [-23255.997] (-23342.989) -- 8:15:23

Average standard deviation of split frequencies: 0.037101

185500 -- (-23308.385) [-23252.097] (-23278.356) (-23297.386) * (-23276.512) [-23254.335] (-23263.996) (-23363.929) -- 8:15:04
186000 -- (-23320.039) [-23249.562] (-23277.234) (-23316.672) * (-23272.989) [-23250.644] (-23271.627) (-23355.091) -- 8:14:44
```

```
186500 -- (-23314.255) [-23264.421] (-23285.706) (-23326.606) * (-23245.334) [-23237.889] (-23260.427) (-23338.332) -- 8:14:25
187000 -- (-23323.407) (-23262.202) [-23248.717] (-23312.252) * (-23250.268) [-23246.152] (-23253.319) (-23345.391) -- 8:14:06
187500 -- (-23325.643) [-23263.160] (-23253.242) (-23295.073) * (-23243.541) [-23251.664] (-23244.419) (-23340.425) -- 8:13:42
188000 -- (-23336.446) (-23269.628) [-23246.599] (-23310.163) * (-23256.291) (-23246.959) [-23238.569] (-23325.805) -- 8:13:23
188500 -- (-23331.068) (-23279.488) [-23228.119] (-23281.450) * (-23248.445) [-23246.541] (-23243.820) (-23329.401) -- 8:13:04
189000 -- (-23306.527) (-23262.202) [-23233.182] (-23293.437) * (-23242.752) [-23251.170] (-23248.079) (-23339.961) -- 8:12:40
189500 -- (-23312.783) (-23261.040) [-23244.819] (-23292.264) * [-23245.384] (-23255.909) (-23267.366) (-23321.916) -- 8:12:17
190000 -- (-23289.093) (-23247.675) [-23243.439] (-23297.413) * (-23254.047) [-23252.360] (-23273.883) (-23324.776) -- 8:11:58

Average standard deviation of split frequencies: 0.036494

190500 -- (-23275.169) (-23248.741) [-23239.666] (-23289.921) * (-23263.334) [-23238.804] (-23292.723) (-23313.501) -- 8:11:34
191000 -- (-23288.762) [-23257.194] (-23253.318) (-23301.828) * (-23267.066) [-23235.703] (-23259.946) (-23316.726) -- 8:11:15
191500 -- (-23288.508) [-23264.982] (-23254.000) (-23303.763) * (-23269.029) [-23238.000] (-23272.493) (-23315.127) -- 8:10:52
192000 -- (-23288.452) (-23265.767) [-23257.829] (-23303.035) * [-23235.158] (-23251.625) (-23276.789) (-23307.670) -- 8:10:33
192500 -- [-23280.633] (-23266.008) (-23261.635) (-23289.283) * [-23246.082] (-23256.026) (-23277.149) (-23348.998) -- 8:10:09
193000 -- (-23277.851) (-23255.258) [-23238.188] (-23266.277) * [-23249.382] (-23250.600) (-23284.219) (-23346.555) -- 8:09:54
193500 -- (-23289.935) (-23264.374) [-23236.058] (-23271.797) * [-23269.838] (-23251.378) (-23273.850) (-23361.972) -- 8:09:35
194000 -- (-23313.024) (-23270.750) [-23231.246] (-23255.185) * (-23280.228) (-23252.004) [-23263.491] (-23370.373) -- 8:09:12
194500 -- (-23307.841) (-23285.010) [-23238.394] (-23255.392) * (-23296.784) (-23253.004) [-23256.949] (-23343.170) -- 8:08:49
195000 -- (-23306.727) (-23279.944) [-23245.161] (-23264.688) * (-23292.367) (-23250.704) [-23242.432] (-23348.828) -- 8:08:30

Average standard deviation of split frequencies: 0.035065

195500 -- (-23312.225) (-23260.136) [-23246.365] (-23253.153) * (-23293.221) (-23267.262) [-23226.324] (-23338.904) -- 8:08:11
196000 -- (-23321.433) (-23267.099) (-23248.317) [-23243.819] * (-23285.342) (-23272.923) [-23224.606] (-23338.810) -- 8:07:52
196500 -- (-23324.501) (-23275.458) (-23262.775) [-23244.533] * (-23284.339) (-23265.023) [-23225.686] (-23358.405) -- 8:07:33
197000 -- (-23321.218) (-23267.365) (-23282.648) [-23240.735] * (-23284.731) (-23271.104) [-23214.759] (-23384.874) -- 8:07:10
197500 -- (-23313.252) (-23260.039) (-23279.213) [-23248.020] * (-23300.370) (-23264.079) [-23222.228] (-23393.575) -- 8:06:51
198000 -- (-23327.033) [-23246.011] (-23271.835) (-23253.703) * (-23285.194) [-23252.822] (-23241.010) (-23359.800) -- 8:06:36
198500 -- (-23302.844) (-23236.928) (-23288.313) [-23248.453] * (-23288.034) (-23239.820) [-23245.508] (-23366.577) -- 8:06:17
199000 -- (-23312.722) [-23234.276] (-23278.327) (-23242.793) * (-23291.996) [-23233.903] (-23245.172) (-23345.505) -- 8:05:58
199500 -- (-23305.964) (-23255.369) (-23266.587) [-23244.034] * (-23272.511) (-23244.316) [-23225.065] (-23347.873) -- 8:05:43
200000 -- (-23311.019) (-23246.688) (-23261.884) [-23241.103] * (-23276.284) (-23244.637) [-23253.853] (-23355.749) -- 8:05:24

Average standard deviation of split frequencies: 0.034040

200500 -- (-23324.366) [-23267.555] (-23271.138) (-23254.522) * (-23267.739) [-23237.635] (-23266.884) (-23378.938) -- 8:05:04
201000 -- (-23322.740) (-23283.907) (-23274.097) (-23246.484) * (-23283.575) [-23234.474] (-23262.106) (-23359.392) -- 8:04:45
201500 -- (-23341.552) (-23279.495) (-23259.205) [-23229.891] * (-23278.361) [-23225.847] (-23252.156) (-23354.102) -- 8:04:30
202000 -- (-23320.703) (-23279.019) (-23281.392) [-23246.333] * (-23266.899) [-23227.933] (-23251.915) (-23379.142) -- 8:04:11
202500 -- (-23318.790) (-23285.322) (-23270.046) [-23253.923] * (-23266.979) [-23207.228] (-23250.511) (-23377.214) -- 8:03:56
203000 -- (-23315.533) (-23277.164) (-23279.277) [-23261.936] * (-23260.027) [-23209.233] (-23258.317) (-23369.023) -- 8:03:37
203500 -- (-23293.449) [-23256.873] (-23292.567) (-23273.418) * (-23244.142) [-23214.094] (-23259.675) (-23360.325) -- 8:03:18
204000 -- (-23297.410) [-23247.326] (-23288.067) (-23283.741) * (-23252.243) [-23233.319] (-23270.888) (-23366.768) -- 8:02:55
204500 -- (-23308.747) [-23240.280] (-23273.973) (-23271.189) * [-23245.983] (-23243.005) (-23259.445) (-23360.103) -- 8:02:40
205000 -- (-23315.113) [-23226.428] (-23302.005) (-23252.849) * (-23245.297) (-23272.183) [-23261.872] (-23357.866) -- 8:02:21

Average standard deviation of split frequencies: 0.033468

205500 -- (-23310.336) [-23226.198] (-23296.574) (-23258.963) * (-23262.648) [-23263.527] (-23261.300) (-23350.369) -- 8:02:06
206000 -- (-23310.084) [-23234.725] (-23312.116) (-23246.233) * (-23259.317) [-23263.354] (-23276.347) (-23364.227) -- 8:01:51
206500 -- (-23318.040) [-23249.119] (-23307.281) (-23251.295) * [-23264.727] (-23270.965) (-23283.406) (-23335.033) -- 8:01:32
207000 -- (-23323.740) [-23255.698] (-23300.110) (-23243.663) * [-23242.077] (-23273.269) (-23272.532) (-23329.952) -- 8:01:13
207500 -- (-23292.597) [-23257.375] (-23289.606) [-23237.026] * [-23240.829] (-23262.909) (-23276.180) (-23328.650) -- 8:00:54
208000 -- (-23278.058) [-23248.389] (-23303.416) (-23242.204) * [-23248.207] (-23270.390) (-23259.415) (-23354.407) -- 8:00:35
208500 -- (-23280.231) (-23256.490) (-23280.419) [-23245.979] * [-23248.622] (-23288.798) (-23259.632) (-23333.909) -- 8:00:16
209000 -- (-23269.827) [-23246.063] (-23293.399) (-23259.418) * [-23232.007] (-23295.258) (-23243.728) (-23340.288) -- 7:59:53
209500 -- (-23270.460) [-23250.001] (-23303.925) (-23278.711) * [-23228.387] (-23309.515) (-23258.094) (-23332.830) -- 7:59:34
210000 -- (-23256.409) [-23253.109] (-23297.920) (-23279.547) * [-23231.012] (-23289.871) (-23246.291) (-23330.975) -- 7:59:16

Average standard deviation of split frequencies: 0.031386

210500 -- [-23241.349] (-23260.294) (-23291.404) (-23299.793) * [-23238.539] (-23283.290) (-23249.588) (-23330.810) -- 7:58:57
211000 -- [-23240.250] (-23251.507) (-23306.076) (-23296.972) * [-23233.158] (-23288.821) (-23263.723) (-23320.241) -- 7:58:38
211500 -- [-23228.006] (-23272.899) (-23295.808) (-23275.556) * [-23256.876] (-23290.017) (-23274.917) (-23333.742) -- 7:58:19
212000 -- [-23222.126] (-23294.295) (-23290.581) (-23279.675) * [-23251.544] (-23288.980) (-23301.633) (-23328.359) -- 7:58:00
212500 -- [-23224.512] (-23289.174) (-23292.859) (-23276.271) * [-23236.875] (-23293.564) (-23323.839) (-23319.857) -- 7:57:41
213000 -- [-23242.196] (-23266.524) (-23295.911) (-23263.775) * [-23248.427] (-23290.105) (-23299.876) (-23319.810) -- 7:57:22
213500 -- (-23260.031) (-23285.131) (-23287.682) [-23249.800] * [-23245.394] (-23284.531) (-23299.006) (-23310.039) -- 7:56:59
214000 -- (-23271.047) [-23281.286] (-23303.127) (-23278.047) * (-23241.302) [-23265.769] (-23314.695) (-23322.449) -- 7:56:40
214500 -- (-23282.774) (-23275.365) (-23296.819) [-23264.241] * [-23252.586] (-23256.595) (-23293.231) (-23344.089) -- 7:56:18
215000 -- (-23327.661) (-23279.479) (-23287.385) [-23249.164] * (-23250.468) [-23255.908] (-23271.821) (-23330.225) -- 7:55:55

Average standard deviation of split frequencies: 0.030341

215500 -- (-23313.182) (-23273.555) (-23285.271) [-23238.223] * (-23253.386) [-23241.454] (-23279.625) (-23328.154) -- 7:55:33
216000 -- (-23299.664) (-23289.054) (-23296.703) [-23232.719] * [-23260.190] (-23254.285) (-23274.146) (-23361.865) -- 7:55:10
216500 -- (-23310.797) (-23296.752) (-23299.288) [-23251.028] * (-23240.489) [-23248.166] (-23274.961) (-23367.738) -- 7:54:48
217000 -- (-23293.370) (-23287.467) (-23293.710) [-23243.441] * [-23240.302] (-23253.620) (-23282.957) (-23352.645) -- 7:54:25
217500 -- (-23279.215) (-23285.931) (-23286.533) [-23244.462] * (-23242.385) [-23263.930] (-23270.111) (-23354.971) -- 7:54:07
218000 -- (-23277.038) (-23294.907) (-23279.903) [-23239.198] * (-23261.939) [-23257.599] (-23300.587) (-23344.379) -- 7:53:44
218500 -- (-23264.647) (-23299.684) (-23271.420) [-23241.268] * (-23247.118) [-23255.805] (-23291.873) (-23350.707) -- 7:53:22
219000 -- (-23267.252) (-23319.071) (-23261.632) [-23228.289] * [-23236.072] (-23273.582) (-23300.399) (-23351.417) -- 7:52:59
219500 -- (-23279.738) (-23307.765) (-23265.564) [-23228.890] * (-23238.807) [-23255.379] (-23285.296) (-23344.877) -- 7:52:41
220000 -- (-23282.381) (-23323.471) (-23254.329) [-23237.432] * (-23238.777) [-23248.930] (-23290.018) (-23334.302) -- 7:52:18

Average standard deviation of split frequencies: 0.029585

220500 -- (-23262.897) (-23304.232) (-23248.491) [-23219.596] * (-23251.401) [-23249.327] (-23278.506) (-23323.942) -- 7:52:00
221000 -- (-23260.748) (-23304.706) (-23270.632) [-23252.694] * (-23260.196) [-23243.148] (-23280.438) (-23358.887) -- 7:51:44
221500 -- (-23254.607) (-23286.527) (-23287.644) [-23237.867] * (-23268.720) [-23239.733] (-23274.948) (-23341.887) -- 7:51:26
222000 -- (-23246.834) (-23309.513) (-23302.579) [-23224.975] * (-23277.063) [-23253.050] (-23260.592) (-23364.149) -- 7:51:07
222500 -- (-23258.596) (-23314.277) (-23311.418) [-23226.147] * (-23265.990) (-23263.892) [-23257.492] (-23340.817) -- 7:50:48
223000 -- (-23249.505) (-23279.594) (-23305.853) [-23242.292] * (-23260.975) (-23266.501) [-23250.137] (-23328.989) -- 7:50:29
223500 -- [-23256.556] (-23278.993) (-23314.585) (-23262.416) * (-23257.174) (-23258.833) [-23243.518] (-23332.737) -- 7:50:14
224000 -- (-23256.229) (-23270.629) (-23288.705) [-23259.879] * (-23254.251) (-23264.901) [-23243.173] (-23318.933) -- 7:49:55
224500 -- (-23257.408) (-23277.529) (-23265.467) [-23260.059] * (-23256.288) (-23250.533) [-23214.957] (-23346.564) -- 7:49:37
225000 -- [-23230.839] (-23283.100) (-23268.358) (-23257.593) * (-23264.815) (-23240.473) [-23230.164] (-23343.180) -- 7:49:14

Average standard deviation of split frequencies: 0.028742

225500 -- [-23226.739] (-23293.724) (-23284.848) (-23276.581) * (-23280.882) (-23248.351) [-23222.536] (-23342.214) -- 7:48:52
226000 -- [-23223.347] (-23283.553) (-23267.632) (-23287.093) * (-23282.311) (-23263.470) [-23229.131] (-23345.519) -- 7:48:34
226500 -- [-23226.076] (-23285.291) (-23283.183) (-23290.451) * (-23251.899) (-23252.028) [-23238.077] (-23347.983) -- 7:48:11
227000 -- [-23239.534] (-23281.869) (-23313.434) (-23295.468) * (-23253.449) (-23260.376) [-23231.275] (-23333.230) -- 7:47:49
227500 -- [-23229.440] (-23281.697) (-23294.509) (-23280.031) * (-23254.322) (-23262.311) [-23239.374] (-23320.648) -- 7:47:31
228000 -- [-23244.948] (-23274.795) (-23308.338) (-23269.498) * (-23267.365) (-23263.013) [-23249.980] (-23338.961) -- 7:47:09
```

```
228500 -- (-23240.882) [-23262.793] (-23302.952) (-23264.843) * [-23246.512] (-23263.249) (-23258.255) (-23346.174) -- 7:46:50
229000 -- [-23250.831] (-23265.383) (-23281.212) (-23285.790) * [-23244.143] (-23272.843) (-23265.471) (-23348.427) -- 7:46:28
229500 -- [-23258.446] [-23258.091] (-23273.229) (-23272.134) * [-23253.572] (-23278.591) [-23255.069] (-23338.848) -- 7:46:09
230000 -- (-23284.913) (-23281.512) [-23271.515] (-23260.535) * (-23237.649) (-23280.048) [-23245.410] (-23309.635) -- 7:45:51

Average standard deviation of split frequencies: 0.027583

230500 -- (-23292.326) (-23266.017) (-23262.130) [-23249.545] * (-23248.110) (-23285.395) [-23255.438] (-23341.306) -- 7:45:32
231000 -- (-23307.878) (-23267.046) (-23284.294) [-23239.584] * (-23267.423) (-23292.862) [-23251.325] (-23362.563) -- 7:45:10
231500 -- (-23302.092) (-23275.014) (-23282.739) [-23242.117] * (-23272.205) (-23265.548) [-23235.670] (-23359.496) -- 7:44:48
232000 -- (-23320.327) (-23284.043) (-23293.492) [-23224.748] * (-23256.263) (-23274.258) [-23239.164] (-23369.387) -- 7:44:26
232500 -- (-23327.959) (-23269.515) (-23293.449) [-23242.627] * (-23251.818) (-23271.162) [-23251.613] (-23383.514) -- 7:44:04
233000 -- (-23334.107) (-23253.948) (-23294.384) [-23258.945] * (-23259.493) [-23258.148] (-23249.335) (-23381.367) -- 7:43:39
233500 -- (-23299.844) (-23265.320) (-23290.310) [-23273.472] * (-23250.673) (-23285.637) [-23253.581] (-23372.755) -- 7:43:14
234000 -- (-23292.434) (-23263.376) (-23279.672) [-23255.062] * (-23254.567) (-23314.571) [-23259.471] (-23372.011) -- 7:42:49
234500 -- (-23295.655) (-23269.808) (-23307.850) [-23249.648] * [-23265.107] (-23297.501) (-23242.210) (-23364.139) -- 7:42:27
235000 -- (-23299.766) [-23266.163] (-23317.255) (-23269.732) * (-23262.497) (-23287.982) [-23236.592] (-23376.075) -- 7:42:02

Average standard deviation of split frequencies: 0.027048

235500 -- (-23304.550) (-23256.982) (-23312.562) [-23256.039] * (-23275.469) (-23293.027) [-23238.198] (-23376.985) -- 7:41:40
236000 -- (-23308.833) [-23256.359] (-23290.939) (-23284.942) * (-23291.632) (-23285.323) [-23248.589] (-23370.593) -- 7:41:18
236500 -- (-23318.134) [-23253.282] (-23294.474) [-23259.364] * (-23308.418) (-23281.178) [-23257.051] (-23347.523) -- 7:40:57
237000 -- (-23330.874) (-23244.993) (-23319.139) [-23263.455] * (-23306.922) (-23283.164) [-23238.708] (-23341.756) -- 7:40:35
237500 -- (-23329.312) (-23262.768) (-23299.136) [-23266.635] * (-23301.131) (-23274.851) [-23245.658] (-23336.840) -- 7:40:16
238000 -- (-23322.979) (-23259.806) (-23291.600) [-23264.044] * (-23302.237) (-23264.023) [-23227.492] (-23318.425) -- 7:39:58
238500 -- (-23308.717) (-23273.989) (-23285.400) [-23253.288] * (-23292.276) (-23254.011) [-23226.141] (-23341.386) -- 7:39:40
239000 -- (-23313.954) (-23287.286) (-23303.671) [-23245.238] * (-23273.626) (-23263.204) [-23228.606] (-23356.923) -- 7:39:18
239500 -- (-23317.905) (-23284.270) (-23311.899) [-23248.677] * (-23254.633) (-23270.603) [-23216.486] (-23363.000) -- 7:38:56
240000 -- (-23317.083) (-23284.233) (-23321.956) [-23252.419] * (-23278.114) (-23285.432) [-23222.178] (-23370.696) -- 7:38:35

Average standard deviation of split frequencies: 0.026440

240500 -- (-23309.462) (-23275.844) (-23304.545) [-23249.828] * (-23258.113) (-23284.922) [-23233.318] (-23366.784) -- 7:38:16
241000 -- (-23326.472) [-23264.859] (-23326.975) (-23246.016) * [-23240.095] (-23290.281) (-23236.745) (-23372.585) -- 7:37:55
241500 -- (-23329.814) (-23256.982) (-23319.329) [-23255.033] * [-23245.431] (-23303.939) (-23250.101) (-23349.027) -- 7:37:33
242000 -- (-23317.813) (-23256.993) (-23316.492) [-23255.138] * [-23245.147] (-23309.742) (-23260.086) (-23362.325) -- 7:37:12
242500 -- (-23295.102) (-23245.457) (-23313.113) [-23246.106] * (-23247.577) (-23301.668) [-23250.957] (-23360.520) -- 7:36:47
243000 -- (-23307.437) (-23242.914) (-23316.658) [-23244.042] * [-23228.906] (-23287.423) (-23264.905) (-23355.047) -- 7:36:25
243500 -- (-23288.619) (-23259.244) (-23297.087) [-23233.536] * [-23225.627] (-23298.517) (-23261.527) (-23360.033) -- 7:36:04
244000 -- (-23277.557) (-23245.586) (-23292.529) [-23227.488] * [-23231.585] (-23285.869) (-23271.918) (-23350.834) -- 7:35:39
244500 -- (-23258.064) (-23249.399) (-23284.340) [-23236.292] * [-23239.524] (-23291.819) (-23289.768) (-23350.309) -- 7:35:18
245000 -- (-23278.418) [-23249.507] (-23294.340) (-23231.620) * [-23229.891] (-23273.800) (-23288.460) (-23357.106) -- 7:34:54

Average standard deviation of split frequencies: 0.025972

245500 -- (-23298.852) (-23271.662) (-23286.045) [-23228.656] * [-23222.794] (-23284.849) (-23288.764) (-23349.119) -- 7:34:32
246000 -- (-23288.284) (-23272.206) (-23311.747) [-23228.581] * [-23225.459] (-23284.392) (-23287.883) (-23347.886) -- 7:34:14
246500 -- (-23302.473) (-23289.668) (-23318.675) [-23227.666] * [-23227.981] (-23284.262) (-23283.615) (-23343.940) -- 7:33:52
247000 -- (-23293.042) (-23299.238) (-23335.942) [-23214.359] * [-23223.171] (-23264.093) (-23280.928) (-23346.623) -- 7:33:31
247500 -- (-23293.669) (-23296.168) (-23319.321) [-23223.886] * [-23236.003] (-23267.323) (-23303.649) (-23337.394) -- 7:33:10
248000 -- (-23292.679) (-23297.306) (-23319.913) [-23220.959] * [-23231.270] (-23260.246) (-23291.715) (-23334.643) -- 7:32:49
248500 -- (-23281.913) (-23287.627) (-23325.498) [-23226.550] * [-23240.165] (-23281.268) (-23303.820) (-23341.307) -- 7:32:27
249000 -- (-23296.412) (-23266.700) (-23328.717) [-23221.904] * (-23250.626) [-23271.415] (-23298.501) (-23351.728) -- 7:32:06
249500 -- (-23293.498) (-23273.610) (-23321.518) [-23234.224] * [-23258.395] (-23263.710) (-23302.291) (-23353.344) -- 7:31:48
250000 -- (-23287.617) (-23283.883) (-23332.841) [-23249.950] * (-23269.980) [-23261.494] (-23292.301) (-23346.477) -- 7:31:27

Average standard deviation of split frequencies: 0.025613

250500 -- (-23262.979) (-23292.267) (-23346.828) [-23268.867] * [-23269.456] (-23267.120) (-23286.845) (-23372.094) -- 7:31:08
251000 -- [-23269.225] (-23285.565) (-23322.208) (-23268.640) * [-23265.075] (-23266.786) (-23292.625) (-23367.140) -- 7:30:47
251500 -- [-23262.089] (-23273.646) (-23326.817) (-23255.442) * [-23250.896] (-23272.523) (-23282.216) (-23351.966) -- 7:30:26
252000 -- (-23247.404) (-23286.723) (-23322.068) [-23243.863] * [-23237.375] (-23271.581) (-23291.023) (-23327.159) -- 7:30:05
252500 -- [-23258.122] (-23279.912) (-23312.378) (-23242.375) * (-23272.162) [-23254.603] (-23289.402) (-23332.939) -- 7:29:41
253000 -- (-23258.542) (-23287.488) (-23311.733) [-23239.366] * (-23265.242) [-23246.590] (-23280.526) (-23324.573) -- 7:29:19
253500 -- (-23265.411) (-23289.683) (-23318.187) [-23249.379] * (-23252.958) [-23248.775] (-23277.181) (-23315.108) -- 7:28:55
254000 -- (-23286.764) (-23267.698) (-23308.963) [-23233.420] * (-23244.885) [-23248.061] (-23270.674) (-23350.108) -- 7:28:31
254500 -- (-23280.050) (-23269.616) (-23314.631) [-23227.377] * (-23255.428) [-23238.618] (-23278.043) (-23345.500) -- 7:28:10
255000 -- (-23259.398) (-23260.461) (-23313.345) [-23242.125] * (-23279.457) [-23238.269] (-23279.122) (-23370.162) -- 7:27:49

Average standard deviation of split frequencies: 0.025166

255500 -- (-23268.581) (-23268.213) (-23311.169) [-23242.687] * (-23280.353) [-23230.007] (-23278.455) (-23387.993) -- 7:27:28
256000 -- (-23267.259) [-23266.897] (-23280.441) (-23238.967) * (-23276.679) [-23242.449] (-23285.460) (-23376.257) -- 7:27:07
256500 -- (-23276.749) [-23276.644] (-23271.528) (-23243.370) * (-23268.516) [-23239.276] (-23285.036) (-23346.660) -- 7:26:49
257000 -- (-23272.754) (-23275.805) (-23295.081) [-23237.776] * (-23272.789) [-23235.481] (-23269.267) (-23366.929) -- 7:26:28
257500 -- (-23273.322) (-23276.583) (-23322.106) [-23233.630] * (-23274.584) [-23230.811] (-23267.692) (-23370.200) -- 7:26:10
258000 -- (-23283.106) (-23272.425) (-23315.685) [-23230.739] * (-23272.058) [-23227.414] (-23305.451) (-23374.016) -- 7:25:49
258500 -- (-23297.188) (-23247.977) (-23321.376) [-23234.278] * (-23272.333) [-23221.695] (-23295.161) (-23341.849) -- 7:25:28
259000 -- (-23278.939) (-23249.973) (-23335.257) [-23228.612] * (-23271.694) [-23236.112] (-23289.177) (-23357.370) -- 7:25:07
259500 -- (-23269.583) (-23264.668) (-23343.646) [-23224.772] * [-23259.990] (-23243.701) (-23282.350) (-23361.739) -- 7:24:49
260000 -- (-23270.540) (-23285.079) (-23329.888) [-23233.792] * [-23246.800] (-23255.666) (-23279.303) (-23366.431) -- 7:24:28

Average standard deviation of split frequencies: 0.024710

260500 -- (-23298.780) (-23294.504) (-23326.879) [-23218.060] * [-23249.481] (-23255.397) (-23280.531) (-23367.852) -- 7:24:07
261000 -- (-23323.673) (-23284.788) (-23313.872) [-23225.060] * [-23248.790] (-23274.207) (-23283.040) (-23366.702) -- 7:23:49
261500 -- (-23314.902) (-23280.611) (-23287.505) [-23233.821] * [-23251.902] (-23264.393) (-23294.447) (-23374.368) -- 7:23:28
262000 -- (-23327.098) (-23260.127) (-23298.113) [-23235.259] * [-23250.927] [-23258.018] (-23285.646) (-23384.273) -- 7:23:07
262500 -- (-23304.575) (-23277.996) (-23308.782) [-23226.608] * [-23254.586] (-23263.986) (-23287.205) (-23389.370) -- 7:22:44
263000 -- (-23308.856) (-23260.944) (-23311.696) [-23225.622] * [-23254.780] [-23258.393] (-23299.413) (-23406.051) -- 7:22:23
263500 -- (-23305.832) (-23276.835) (-23320.216) [-23224.652] * [-23237.446] (-23264.841) (-23315.011) (-23390.243) -- 7:22:05
264000 -- (-23313.216) (-23271.785) (-23309.184) [-23237.824] * [-23251.907] (-23257.790) (-23299.836) (-23361.605) -- 7:21:44
264500 -- (-23320.325) (-23274.354) (-23312.722) [-23236.256] * [-23249.456] (-23279.540) (-23310.951) (-23376.322) -- 7:21:26
265000 -- (-23340.264) (-23298.680) (-23288.426) [-23249.291] * [-23251.532] (-23286.112) (-23286.824) (-23375.932) -- 7:21:05

Average standard deviation of split frequencies: 0.024451

265500 -- (-23327.924) (-23304.433) (-23291.584) [-23252.061] * [-23260.073] (-23302.324) (-23306.189) (-23382.933) -- 7:20:44
266000 -- (-23318.125) (-23287.940) (-23295.679) [-23253.300] * [-23276.401] (-23288.028) (-23308.842) (-23361.893) -- 7:20:23
266500 -- (-23309.996) (-23297.993) (-23308.176) [-23269.191] * (-23265.078) [-23287.925] (-23285.931) (-23362.419) -- 7:20:03
267000 -- (-23314.211) (-23288.314) (-23306.656) [-23248.729] * [-23261.940] (-23307.748) (-23286.191) (-23346.233) -- 7:19:42
267500 -- (-23312.466) (-23298.471) (-23306.656) [-23251.069] * (-23247.671) (-23281.512) [-23254.692] (-23327.739) -- 7:19:21
268000 -- (-23317.633) (-23313.785) (-23300.533) [-23247.349] * (-23251.307) (-23283.783) [-23243.621] (-23334.534) -- 7:19:01
268500 -- (-23307.476) (-23277.470) (-23295.117) [-23231.614] * (-23257.520) (-23281.376) [-23246.967] (-23324.824) -- 7:18:43
269000 -- (-23315.729) (-23289.943) (-23304.788) [-23236.947] * (-23256.793) (-23293.017) [-23252.232] (-23346.023) -- 7:18:22
269500 -- (-23316.481) (-23271.451) (-23326.762) [-23232.849] * [-23276.085] (-23277.793) (-23248.784) (-23339.201) -- 7:18:01
270000 -- (-23319.863) (-23285.498) (-23333.774) [-23223.910] * [-23268.237] (-23282.536) (-23252.193) (-23362.575) -- 7:17:41
```

```
Average standard deviation of split frequencies: 0.025076

270500 -- (-23314.085) (-23270.446) (-23323.843) [-23240.305] * (-23276.538) (-23284.050) [-23263.726] (-23360.616) -- 7:17:20
271000 -- (-23305.821) (-23267.278) (-23332.070) [-23230.542] * (-23296.104) (-23288.899) [-23245.870] (-23377.971) -- 7:16:59
271500 -- (-23305.230) (-23265.942) (-23329.303) [-23222.434] * (-23276.362) (-23297.321) [-23245.112] (-23366.198) -- 7:16:41
272000 -- (-23284.345) (-23279.285) (-23349.171) [-23208.387] * (-23265.737) (-23315.984) [-23250.633] (-23370.775) -- 7:16:21
272500 -- (-23266.291) (-23261.838) (-23342.268) [-23228.791] * [-23261.454] (-23324.420) (-23259.584) (-23386.067) -- 7:16:00
273000 -- (-23272.414) (-23287.379) (-23350.762) [-23219.767] * (-23265.035) (-23307.894) [-23245.048] (-23385.626) -- 7:15:42
273500 -- (-23281.631) (-23279.653) (-23350.147) [-23224.932] * (-23266.154) (-23297.148) [-23244.787] (-23372.507) -- 7:15:24
274000 -- (-23281.342) (-23275.455) (-23348.137) [-23228.079] * [-23256.910] (-23292.033) (-23259.498) (-23374.248) -- 7:15:06
274500 -- (-23275.321) (-23269.191) (-23328.533) [-23237.908] * [-23273.540] (-23289.641) (-23254.489) (-23359.555) -- 7:14:48
275000 -- (-23270.078) (-23289.313) (-23345.176) [-23245.060] * (-23266.809) (-23308.880) [-23245.483] (-23376.849) -- 7:14:31

Average standard deviation of split frequencies: 0.025270

275500 -- (-23263.594) (-23299.060) (-23325.677) [-23223.258] * (-23259.316) (-23307.478) [-23244.951] (-23383.764) -- 7:14:10
276000 -- (-23279.218) (-23301.580) (-23341.456) [-23216.954] * [-23268.970] (-23299.686) (-23247.000) (-23383.410) -- 7:13:52
276500 -- (-23284.253) (-23289.838) (-23320.659) [-23213.612] * [-23256.460] (-23304.658) (-23264.688) (-23366.366) -- 7:13:31
277000 -- (-23273.467) (-23288.573) (-23324.869) [-23234.871] * [-23266.527] (-23298.021) (-23273.952) (-23380.989) -- 7:13:11
277500 -- (-23281.937) (-23295.809) (-23308.936) [-23235.468] * [-23257.717] (-23293.234) (-23264.888) (-23380.416) -- 7:12:50
278000 -- (-23274.398) (-23300.961) (-23304.627) [-23242.939] * [-23258.692] (-23281.440) (-23279.455) (-23397.847) -- 7:12:30
278500 -- (-23266.717) (-23317.443) (-23309.403) [-23248.959] * [-23241.497] (-23284.167) (-23281.266) (-23403.154) -- 7:12:09
279000 -- (-23267.333) (-23316.405) (-23298.709) [-23253.775] * [-23238.883] (-23270.361) (-23281.950) (-23395.853) -- 7:11:49
279500 -- (-23255.219) (-23314.017) (-23296.229) [-23240.825] * [-23239.500] (-23276.201) (-23272.760) (-23392.772) -- 7:11:29
280000 -- (-23262.946) (-23305.948) (-23326.692) [-23244.770] * [-23239.111] (-23255.808) (-23264.256) (-23388.726) -- 7:11:11

Average standard deviation of split frequencies: 0.025156

280500 -- (-23272.619) (-23306.950) (-23287.703) [-23246.757] * [-23229.473] (-23266.458) (-23255.970) (-23361.598) -- 7:10:50
281000 -- (-23296.454) (-23297.895) (-23280.896) [-23250.822] * [-23251.688] (-23287.529) (-23243.852) (-23364.209) -- 7:10:32
281500 -- (-23287.803) (-23298.106) (-23280.231) [-23257.314] * (-23262.081) (-23286.126) [-23240.990] (-23369.666) -- 7:10:12
282000 -- (-23274.984) (-23291.217) (-23287.406) [-23243.290] * (-23265.032) (-23269.891) [-23233.825] (-23360.001) -- 7:09:54
282500 -- (-23272.048) (-23317.659) (-23294.270) [-23242.969] * (-23266.709) (-23277.547) [-23235.946] (-23364.310) -- 7:09:34
283000 -- (-23266.347) (-23322.260) (-23288.686) [-23250.880] * (-23260.317) (-23257.898) [-23240.463] (-23358.576) -- 7:09:16
283500 -- (-23263.022) (-23321.294) (-23315.502) [-23254.572] * (-23247.243) (-23264.402) [-23227.367] (-23345.783) -- 7:08:55
284000 -- (-23274.483) (-23310.848) (-23294.439) [-23248.978] * (-23243.331) (-23273.243) [-23221.246] (-23360.416) -- 7:08:38
284500 -- (-23269.356) (-23291.399) (-23288.659) [-23244.671] * (-23260.496) (-23272.253) [-23210.005] (-23366.104) -- 7:08:17
285000 -- (-23279.023) (-23290.942) (-23295.572) [-23242.855] * (-23259.634) (-23265.914) [-23218.072] (-23338.278) -- 7:07:59

Average standard deviation of split frequencies: 0.025231

285500 -- (-23283.187) (-23304.157) (-23279.113) [-23253.731] * (-23265.151) (-23265.977) [-23233.362] (-23350.593) -- 7:07:41
286000 -- (-23289.686) (-23328.205) (-23299.264) [-23253.331] * (-23269.516) (-23241.576) [-23242.728] (-23354.017) -- 7:07:21
286500 -- [-23281.494] (-23328.317) (-23307.578) (-23246.766) * (-23292.804) (-23248.694) [-23231.902] (-23337.747) -- 7:07:03
287000 -- (-23270.885) (-23312.446) (-23310.893) [-23250.756] * (-23296.352) (-23235.038) [-23224.660] (-23358.159) -- 7:06:45
287500 -- (-23273.732) (-23327.853) (-23306.308) [-23251.564] * (-23300.077) (-23234.656) [-23254.542] (-23354.357) -- 7:06:28
288000 -- (-23267.082) (-23338.866) (-23299.626) [-23254.929] * (-23319.620) (-23246.362) [-23239.463] (-23369.743) -- 7:06:07
288500 -- (-23261.671) (-23324.144) (-23285.797) [-23248.313] * (-23319.715) (-23256.339) [-23234.039] (-23336.807) -- 7:05:47
289000 -- (-23250.252) (-23343.902) (-23292.800) [-23241.328] * (-23307.909) (-23245.841) [-23238.814] (-23335.433) -- 7:05:29
289500 -- [-23246.914] (-23338.195) (-23282.788) (-23232.861) * (-23299.668) (-23242.648) [-23230.227] (-23342.398) -- 7:05:09
290000 -- (-23262.039) (-23343.277) (-23285.180) [-23254.170] * (-23293.820) (-23264.134) [-23244.299] (-23362.488) -- 7:04:51

Average standard deviation of split frequencies: 0.025019

290500 -- (-23258.425) (-23327.549) (-23283.724) [-23273.470] * [-23287.362] (-23280.455) (-23237.239) (-23368.110) -- 7:04:33
291000 -- (-23252.301) (-23325.628) (-23278.876) [-23252.027] * (-23278.245) (-23286.064) [-23236.308] (-23354.875) -- 7:04:13
291500 -- (-23266.242) (-23298.497) (-23264.219) [-23239.679] * (-23278.346) (-23280.239) [-23238.587] (-23363.052) -- 7:03:55
292000 -- (-23283.618) (-23294.306) (-23249.847) [-23248.805] * (-23260.857) (-23276.953) [-23243.123] (-23353.231) -- 7:03:37
292500 -- (-23295.132) (-23302.485) (-23254.105) [-23251.155] * (-23260.210) (-23279.902) [-23241.190] (-23345.967) -- 7:03:19
293000 -- (-23305.520) (-23326.586) (-23265.017) [-23229.154] * (-23240.041) (-23280.375) [-23238.025] (-23334.757) -- 7:03:02
293500 -- (-23314.891) (-23305.945) (-23285.270) [-23230.820] * [-23242.076] (-23278.949) (-23257.596) (-23340.435) -- 7:02:41
294000 -- (-23307.671) (-23304.955) (-23284.502) [-23229.012] * [-23244.109] (-23273.872) (-23235.487) (-23321.389) -- 7:02:23
294500 -- (-23304.273) (-23285.609) (-23265.136) [-23226.782] * (-23259.661) (-23273.171) [-23232.995] (-23315.554) -- 7:02:03
295000 -- (-23322.747) (-23283.313) (-23261.955) [-23237.883] * [-23234.607] (-23269.498) (-23231.551) (-23314.390) -- 7:01:45

Average standard deviation of split frequencies: 0.025004

295500 -- (-23338.746) (-23270.511) (-23265.396) [-23247.683] * [-23238.811] (-23268.998) (-23241.501) (-23322.531) -- 7:01:25
296000 -- (-23334.525) (-23282.965) (-23263.626) [-23241.011] * [-23252.065] (-23291.772) (-23222.035) (-23308.495) -- 7:01:07
296500 -- (-23323.121) (-23283.053) (-23282.731) [-23244.077] * (-23274.405) (-23278.641) [-23237.918] (-23322.504) -- 7:00:50
297000 -- (-23322.541) (-23289.863) [-23274.970] (-23247.619) * (-23266.287) (-23273.713) [-23233.678] (-23333.832) -- 7:00:32
297500 -- (-23322.654) (-23280.592) (-23268.040) [-23237.474] * (-23256.751) (-23266.370) [-23236.645] (-23339.676) -- 7:00:12
298000 -- (-23327.470) (-23271.671) (-23273.708) [-23240.876] * (-23265.972) (-23290.195) [-23223.824] (-23326.097) -- 6:59:49
298500 -- (-23329.141) (-23257.063) (-23264.335) [-23238.048] * (-23288.369) (-23279.616) [-23244.774] (-23337.699) -- 6:59:27
299000 -- (-23313.031) (-23253.814) (-23274.340) [-23228.947] * (-23287.072) (-23263.763) [-23252.774] (-23327.767) -- 6:59:06
299500 -- (-23300.310) (-23267.658) (-23268.036) [-23238.470] * (-23280.591) (-23272.214) [-23242.781] (-23344.765) -- 6:58:44
300000 -- (-23294.981) (-23292.396) [-23280.180] (-23240.817) * (-23280.882) [-23259.664] (-23254.748) (-23343.884) -- 6:58:24

Average standard deviation of split frequencies: 0.025086

300500 -- (-23303.014) (-23300.233) (-23273.295) [-23245.117] * (-23277.067) [-23249.325] (-23256.867) (-23356.282) -- 6:58:01
301000 -- (-23317.421) (-23307.639) (-23272.163) [-23248.414] * (-23276.386) (-23262.055) [-23253.769] (-23329.370) -- 6:57:41
301500 -- (-23285.213) (-23322.051) (-23279.653) [-23249.573] * (-23295.650) (-23263.685) [-23243.538] (-23324.544) -- 6:57:19
302000 -- (-23284.738) (-23318.186) (-23281.397) [-23244.538] * (-23291.979) (-23268.700) [-23242.466] (-23307.164) -- 6:56:59
302500 -- (-23269.998) (-23296.928) (-23276.857) [-23248.358] * (-23303.172) (-23268.055) [-23222.642] (-23302.784) -- 6:56:39
303000 -- [-23252.228] (-23335.964) (-23287.065) (-23251.976) * (-23281.074) (-23277.981) [-23238.339] (-23298.887) -- 6:56:19
303500 -- (-23262.694) (-23303.271) (-23278.603) [-23263.678] * (-23286.910) (-23267.655) [-23254.802] (-23304.950) -- 6:55:59
304000 -- [-23242.356] (-23288.667) (-23290.438) (-23261.875) * (-23274.334) (-23257.266) [-23252.722] (-23295.324) -- 6:55:41
304500 -- [-23237.746] (-23271.337) (-23295.928) (-23240.955) * (-23268.281) (-23267.789) [-23251.612] (-23319.290) -- 6:55:23
305000 -- [-23241.834] (-23267.891) (-23310.669) (-23249.314) * (-23254.222) (-23281.198) [-23245.408] (-23324.808) -- 6:55:06

Average standard deviation of split frequencies: 0.024528

305500 -- [-23259.308] (-23265.675) (-23295.892) (-23255.856) * (-23258.997) (-23287.937) [-23241.727] (-23326.361) -- 6:54:46
306000 -- (-23257.139) (-23263.440) (-23318.266) [-23251.426] * (-23238.170) (-23285.211) [-23241.523] (-23304.430) -- 6:54:26
306500 -- [-23255.588] (-23263.770) (-23292.478) (-23260.532) * (-23249.459) (-23282.454) [-23247.663] (-23295.169) -- 6:54:08
307000 -- [-23265.771] (-23307.547) (-23282.554) (-23260.721) * (-23256.546) (-23279.910) [-23253.595] (-23302.999) -- 6:53:48
307500 -- [-23253.047] (-23294.293) (-23283.459) (-23260.560) * (-23270.699) (-23294.335) [-23241.901] (-23296.255) -- 6:53:30
308000 -- (-23263.823) (-23309.800) (-23295.772) [-23257.026] * (-23261.012) (-23303.549) [-23250.977] (-23297.417) -- 6:53:12
308500 -- [-23275.113] (-23308.970) (-23285.486) (-23255.993) * (-23265.454) (-23295.345) [-23236.265] (-23315.714) -- 6:52:52
309000 -- (-23299.188) (-23297.343) (-23280.865) [-23269.477] * (-23260.928) (-23311.474) [-23236.411] (-23325.408) -- 6:52:33
309500 -- (-23279.352) (-23309.088) (-23295.154) [-23270.518] * (-23252.686) (-23324.268) [-23250.074] (-23316.189) -- 6:52:17
310000 -- [-23273.450] (-23324.099) (-23300.607) (-23288.916) * (-23260.872) (-23334.371) [-23241.053] (-23332.083) -- 6:51:59

Average standard deviation of split frequencies: 0.023712

310500 -- [-23268.235] (-23350.822) (-23286.226) (-23276.068) * (-23271.385) (-23298.057) [-23261.155] (-23322.335) -- 6:51:42
```

```
311000 -- (-23265.431) (-23345.172) (-23296.378) [-23265.379] * (-23262.120) (-23316.176) [-23241.952] (-23288.536) -- 6:51:26
311500 -- [-23264.066] (-23334.335) (-23303.763) (-23248.037) * (-23276.720) (-23285.678) [-23256.320] (-23286.113) -- 6:51:06
312000 -- (-23263.146) (-23319.419) (-23293.160) [-23248.026] * (-23286.048) (-23309.872) [-23257.059] (-23290.122) -- 6:50:48
312500 -- (-23261.607) (-23310.453) (-23296.548) [-23240.169] * (-23278.427) (-23301.922) [-23244.489] (-23306.250) -- 6:50:29
313000 -- (-23255.633) (-23318.370) (-23290.226) [-23235.059] * (-23281.745) (-23313.142) [-23240.128] (-23311.390) -- 6:50:11
313500 -- [-23236.871] (-23314.894) (-23285.511) (-23226.987) * (-23244.239) (-23327.204) [-23246.186] (-23313.820) -- 6:49:51
314000 -- [-23247.163] (-23301.988) (-23259.809) (-23228.875) * (-23247.338) (-23313.617) [-23239.212] (-23318.046) -- 6:49:31
314500 -- [-23243.488] (-23305.185) (-23259.401) (-23240.244) * [-23229.864] (-23303.169) (-23252.135) (-23308.794) -- 6:49:13
315000 -- (-23262.796) (-23300.425) [-23254.934] (-23247.446) * (-23235.360) (-23304.903) [-23243.673] (-23315.217) -- 6:48:56

Average standard deviation of split frequencies: 0.023645

315500 -- (-23283.678) (-23300.179) [-23256.709] (-23261.603) * [-23225.211] (-23294.867) (-23272.594) (-23305.202) -- 6:48:36
316000 -- (-23284.914) (-23296.894) [-23266.851] (-23267.412) * [-23234.567] (-23327.063) (-23292.391) (-23296.454) -- 6:48:18
316500 -- [-23257.808] (-23293.035) (-23278.086) (-23283.590) * [-23232.834] (-23301.092) (-23292.956) (-23293.818) -- 6:48:00
317000 -- (-23261.887) (-23279.931) [-23261.923] (-23299.484) * [-23241.543] (-23310.098) (-23278.062) (-23305.191) -- 6:47:40
317500 -- [-23247.238] (-23295.947) (-23284.125) (-23294.464) * [-23232.942] (-23340.714) (-23284.590) (-23308.756) -- 6:47:21
318000 -- (-23256.683) [-23267.517] (-23301.496) (-23309.494) * [-23236.718] (-23342.975) (-23279.982) (-23310.167) -- 6:47:03
318500 -- [-23253.086] (-23268.615) (-23294.108) (-23314.027) * [-23242.655] (-23314.001) (-23268.129) (-23312.896) -- 6:46:43
319000 -- [-23251.353] (-23263.926) (-23286.613) (-23316.026) * (-23244.038) (-23319.722) [-23264.364] (-23286.075) -- 6:46:23
319500 -- [-23236.941] (-23268.582) (-23268.047) (-23296.316) * (-23260.317) (-23304.992) [-23266.187] (-23277.660) -- 6:46:03
320000 -- [-23248.461] (-23269.392) (-23277.256) (-23339.354) * [-23248.616] (-23284.489) (-23266.815) (-23282.394) -- 6:45:44

Average standard deviation of split frequencies: 0.023042

320500 -- [-23229.023] (-23271.226) (-23273.518) (-23330.996) * (-23248.154) (-23282.665) [-23252.927] (-23316.699) -- 6:45:24
321000 -- [-23243.014] (-23274.569) (-23267.030) (-23318.083) * (-23257.655) (-23295.149) [-23251.261] (-23328.953) -- 6:45:06
321500 -- [-23248.460] (-23282.253) (-23290.243) (-23306.198) * (-23263.644) (-23278.281) [-23254.375] (-23347.824) -- 6:44:46
322000 -- [-23242.276] (-23268.707) (-23271.018) (-23304.652) * (-23280.026) (-23282.553) [-23245.403] (-23331.250) -- 6:44:26
322500 -- [-23234.086] (-23268.643) (-23274.078) (-23302.205) * (-23277.192) (-23283.760) [-23231.894] (-23328.525) -- 6:44:05
323000 -- [-23235.882] (-23276.897) (-23289.928) (-23301.490) * (-23277.016) (-23284.266) [-23234.437] (-23303.657) -- 6:43:47
323500 -- [-23244.922] (-23270.761) (-23282.645) (-23306.125) * (-23301.343) (-23270.446) [-23234.889] (-23338.923) -- 6:43:27
324000 -- (-23260.138) (-23263.212) [-23260.797] (-23331.331) * (-23313.292) (-23275.725) [-23237.307] (-23338.192) -- 6:43:09
324500 -- [-23249.451] (-23262.470) (-23271.774) (-23314.167) * (-23329.206) (-23262.252) [-23231.771] (-23335.001) -- 6:42:50
325000 -- (-23269.153) [-23253.411] (-23259.316) (-23317.409) * (-23319.610) (-23274.337) [-23235.202] (-23325.966) -- 6:42:30

Average standard deviation of split frequencies: 0.022564

325500 -- (-23271.326) (-23262.350) [-23251.402] (-23327.306) * (-23312.924) (-23241.083) [-23221.419] (-23323.013) -- 6:42:10
326000 -- (-23270.749) (-23267.075) [-23239.901] (-23308.874) * (-23292.555) (-23263.628) [-23229.531] (-23307.908) -- 6:41:51
326500 -- (-23273.511) (-23301.776) [-23228.825] (-23306.637) * (-23283.458) (-23272.929) [-23244.650] (-23310.695) -- 6:41:33
327000 -- (-23247.967) (-23317.100) [-23216.976] (-23286.823) * (-23272.921) (-23281.780) [-23245.436] (-23323.244) -- 6:41:13
327500 -- (-23256.918) (-23327.586) [-23216.541] (-23298.374) * (-23278.834) (-23289.392) [-23237.036] (-23302.377) -- 6:40:53
328000 -- (-23248.283) (-23330.743) [-23223.985] (-23302.447) * (-23284.356) (-23299.898) [-23219.440] (-23305.652) -- 6:40:36
328500 -- (-23275.540) (-23336.636) [-23216.481] (-23301.701) * (-23288.756) (-23327.703) [-23219.578] (-23297.110) -- 6:40:16
329000 -- (-23279.735) (-23313.265) [-23208.413] (-23309.329) * (-23286.785) (-23324.277) [-23224.359] (-23272.438) -- 6:39:58
329500 -- (-23283.470) (-23310.864) [-23197.855] (-23323.533) * (-23282.077) (-23325.914) [-23226.969] (-23265.591) -- 6:39:39
330000 -- (-23285.704) (-23309.649) [-23214.513] (-23326.976) * (-23295.299) (-23334.959) [-23226.938] (-23258.941) -- 6:39:19

Average standard deviation of split frequencies: 0.022211

330500 -- (-23282.852) (-23304.091) [-23225.155] (-23304.549) * (-23290.416) (-23340.705) [-23218.326] (-23273.898) -- 6:38:59
331000 -- (-23268.995) (-23301.549) [-23227.928] (-23295.909) * (-23297.179) (-23355.338) [-23232.090] (-23272.508) -- 6:38:38
331500 -- (-23260.674) (-23319.603) [-23229.506] (-23307.811) * (-23290.219) (-23360.775) [-23220.250] (-23266.712) -- 6:38:20
332000 -- (-23257.630) (-23330.046) [-23246.354] (-23298.023) * (-23299.639) (-23353.936) [-23229.841] (-23267.222) -- 6:38:00
332500 -- (-23253.072) (-23330.439) (-23260.267) (-23299.781) * (-23295.219) (-23329.302) [-23235.550] (-23288.602) -- 6:37:43
333000 -- (-23264.136) (-23323.839) [-23256.430] (-23303.463) * (-23284.977) (-23352.702) [-23230.457] (-23302.074) -- 6:37:25
333500 -- (-23264.770) (-23328.504) [-23260.408] (-23319.555) * (-23281.045) (-23358.122) [-23239.921] (-23294.116) -- 6:37:10
334000 -- (-23258.836) (-23339.352) [-23247.765] (-23317.892) * (-23270.156) (-23339.428) [-23246.246] (-23275.832) -- 6:36:52
334500 -- [-23242.215] (-23306.845) (-23255.770) (-23310.670) * (-23287.846) (-23317.895) [-23243.163] (-23268.342) -- 6:36:34
335000 -- (-23261.670) (-23312.546) [-23265.660] (-23318.926) * (-23273.462) (-23311.698) [-23232.342] (-23258.842) -- 6:36:17

Average standard deviation of split frequencies: 0.021903

335500 -- [-23259.167] (-23311.217) (-23285.822) (-23325.738) * (-23264.997) (-23318.195) [-23234.260] (-23266.755) -- 6:35:59
336000 -- (-23251.876) (-23316.643) [-23265.938] (-23338.653) * [-23264.423] (-23309.745) (-23242.303) (-23277.877) -- 6:35:41
336500 -- [-23246.262] (-23286.255) (-23269.064) (-23341.077) * (-23267.372) (-23292.499) [-23237.175] (-23266.242) -- 6:35:24
337000 -- [-23222.138] (-23283.225) (-23282.257) (-23321.998) * (-23284.145) (-23283.557) [-23238.245] (-23280.550) -- 6:35:04
337500 -- [-23238.765] (-23278.643) (-23270.945) (-23324.048) * (-23262.039) (-23322.601) [-23242.869] (-23276.649) -- 6:34:47
338000 -- [-23234.471] (-23273.659) (-23263.123) (-23318.514) * (-23265.129) (-23329.116) [-23243.945] (-23291.945) -- 6:34:29
338500 -- [-23246.432] (-23279.953) (-23251.075) (-23313.323) * [-23263.524] (-23329.426) (-23238.494) (-23246.927) -- 6:34:11
339000 -- (-23250.325) (-23280.094) [-23247.277] (-23310.865) * (-23238.006) (-23332.880) [-23229.148] (-23258.005) -- 6:33:54
339500 -- [-23226.275] (-23299.980) (-23246.727) (-23312.585) * [-23244.769] (-23331.749) (-23240.855) (-23265.853) -- 6:33:36
340000 -- [-23236.116] (-23298.236) (-23252.102) (-23315.262) * (-23264.351) (-23317.409) [-23243.089] (-23278.355) -- 6:33:18

Average standard deviation of split frequencies: 0.022445

340500 -- [-23241.099] (-23312.062) (-23247.311) (-23296.893) * [-23256.874] (-23308.768) (-23252.553) (-23277.403) -- 6:33:01
341000 -- (-23252.990) (-23318.322) [-23241.413] (-23288.416) * [-23253.127] (-23305.447) (-23253.793) (-23264.557) -- 6:32:45
341500 -- [-23243.541] (-23311.497) (-23243.037) (-23286.642) * (-23287.586) (-23311.265) [-23250.982] (-23259.314) -- 6:32:29
342000 -- [-23229.444] (-23329.737) (-23263.625) (-23297.563) * (-23289.939) (-23312.936) [-23251.813] (-23266.256) -- 6:32:14
342500 -- [-23243.291] (-23315.825) (-23247.569) (-23293.300) * (-23297.338) (-23301.238) [-23239.835] (-23295.367) -- 6:31:56
343000 -- (-23254.796) (-23303.878) [-23248.828] (-23293.767) * (-23318.462) (-23287.084) [-23259.383] (-23280.222) -- 6:31:40
343500 -- (-23259.717) (-23301.182) [-23247.079] (-23312.826) * (-23324.298) (-23288.134) [-23270.688] (-23263.681) -- 6:31:23
344000 -- [-23245.218] (-23305.270) (-23242.659) (-23275.649) * (-23329.421) (-23274.309) [-23277.639] (-23260.288) -- 6:31:07
344500 -- [-23251.957] (-23288.542) (-23250.206) (-23290.467) * (-23322.254) (-23278.416) (-23282.756) [-23261.042] -- 6:30:49
345000 -- [-23263.130] (-23270.918) (-23256.821) (-23272.697) * (-23304.001) (-23297.622) (-23271.954) [-23242.216] -- 6:30:31

Average standard deviation of split frequencies: 0.022433

345500 -- (-23257.260) (-23279.371) [-23262.994] (-23306.739) * (-23291.004) (-23338.682) [-23275.152] (-23247.799) -- 6:30:14
346000 -- (-23261.568) (-23291.328) [-23234.636] (-23301.960) * (-23290.326) (-23331.771) (-23294.371) [-23236.506] -- 6:29:56
346500 -- (-23262.746) (-23273.542) [-23247.361] (-23298.752) * (-23300.670) (-23339.140) (-23282.506) [-23243.009] -- 6:29:37
347000 -- (-23274.704) (-23291.289) [-23243.375] (-23289.312) * (-23290.327) (-23331.736) (-23287.244) [-23243.335] -- 6:29:21
347500 -- (-23277.338) (-23275.138) [-23246.728] (-23291.347) * (-23256.682) (-23349.231) (-23299.183) [-23250.840] -- 6:29:03
348000 -- (-23273.487) (-23253.936) [-23230.802] (-23300.074) * [-23264.575] (-23347.728) (-23289.426) (-23255.991) -- 6:28:45
348500 -- (-23253.490) (-23249.642) [-23242.236] (-23301.022) * (-23277.898) (-23326.672) (-23291.301) [-23252.496] -- 6:28:30
349000 -- (-23270.519) (-23245.063) [-23241.755] (-23303.412) * [-23247.861] (-23322.653) (-23297.958) (-23259.890) -- 6:28:12
349500 -- (-23252.762) (-23229.947) [-23237.567] (-23291.994) * (-23258.257) (-23320.867) (-23279.731) [-23247.926] -- 6:27:56
350000 -- (-23263.584) [-23243.534] (-23251.794) (-23295.311) * (-23270.649) (-23314.249) (-23290.033) [-23247.686] -- 6:27:40

Average standard deviation of split frequencies: 0.022362

350500 -- (-23276.971) [-23241.322] (-23248.267) (-23330.008) * (-23270.375) (-23307.510) (-23269.220) [-23231.508] -- 6:27:24
351000 -- (-23265.560) [-23252.284] (-23243.803) (-23314.089) * (-23266.205) (-23322.701) (-23255.486) [-23221.371] -- 6:27:09
351500 -- (-23275.734) [-23248.766] (-23248.044) (-23289.200) * (-23249.725) (-23320.690) (-23275.117) [-23234.147] -- 6:26:53
352000 -- (-23263.154) [-23242.120] (-23265.037) (-23278.530) * (-23255.290) (-23290.417) (-23262.785) [-23234.802] -- 6:26:35
352500 -- (-23257.644) [-23242.664] (-23263.199) (-23266.918) * (-23249.771) (-23299.531) (-23253.789) [-23226.832] -- 6:26:17
```

353000 -- (-23262.160) [-23251.708] (-23251.007) (-23281.265) * (-23250.671) (-23293.330) (-23257.774) [-23219.938] -- 6:26:00
353500 -- (-23249.307) (-23241.694) [-23244.178] (-23259.870) * (-23251.587) (-23323.404) (-23250.360) [-23249.964] -- 6:25:44
354000 -- (-23271.426) (-23241.104) [-23245.306] (-23263.924) * [-23262.641] (-23333.178) (-23267.197) (-23254.983) -- 6:25:28
354500 -- (-23262.190) [-23252.341] (-23244.948] (-23265.006) * (-23261.866) (-23313.953) (-23257.795) [-23250.891] -- 6:25:12
355000 -- (-23273.727) (-23269.566) [-23244.066] (-23266.371) * (-23266.851) (-23310.004) (-23273.934) [-23233.364] -- 6:24:56

Average standard deviation of split frequencies: 0.021580

355500 -- (-23279.500) [-23252.364] (-23245.435) (-23280.431) * (-23253.969) (-23326.052) (-23278.154) [-23243.404] -- 6:24:40
356000 -- (-23279.955) (-23240.458) [-23268.481] (-23286.001) * (-23269.275) (-23328.477) (-23275.915) [-23249.527] -- 6:24:24
356500 -- (-23273.147) [-23227.592] (-23266.584) (-23290.085) * (-23247.688) (-23306.446) (-23277.421) [-23245.712] -- 6:24:08
357000 -- (-23265.100) (-23248.585) [-23261.773] (-23293.671) * (-23246.996) (-23301.586) [-23271.331] (-23266.609) -- 6:23:50
357500 -- (-23279.233) (-23248.273) [-23245.913] (-23293.563) * [-23233.801] (-23326.727) (-23272.489) (-23267.518) -- 6:23:34
358000 -- (-23282.012) [-23237.206] (-23253.702) (-23286.474) * (-23255.276) (-23334.987) [-23262.527] (-23260.245) -- 6:23:17
358500 -- (-23271.189) [-23224.694] (-23253.169) (-23282.361) * (-23257.671) (-23341.937) [-23250.530] (-23264.973) -- 6:23:01
359000 -- (-23266.156) [-23242.717] (-23256.598) (-23281.206) * (-23247.221) (-23336.411) [-23254.502) [-23249.415] -- 6:22:43
359500 -- (-23274.604) [-23249.848] (-23256.435] (-23278.702) * [-23248.731] (-23323.967) (-23253.145) (-23262.629) -- 6:22:29
360000 -- (-23291.234) [-23261.477] (-23268.180) (-23286.505) * [-23248.955] (-23318.791) (-23279.578) (-23281.903) -- 6:22:11

Average standard deviation of split frequencies: 0.021402

360500 -- (-23278.138) [-23253.863] (-23257.577) (-23291.777) * [-23251.898] (-23312.434) (-23293.270) (-23281.257) -- 6:21:55
361000 -- (-23283.481) (-23252.739) [-23265.497] (-23299.331) * [-23253.217] (-23298.227) (-23300.161) (-23273.753) -- 6:21:37
361500 -- (-23296.594) [-23238.320] [-23252.781] (-23299.975) * (-23251.855) (-23306.630) [-23276.878] (-23289.192) -- 6:21:20
362000 -- (-23304.070) [-23222.174] (-23229.120) (-23301.690) * (-23262.744) (-23319.504) [-23269.025] (-23279.302) -- 6:21:02
362500 -- (-23298.196) [-23214.507] (-23228.803) (-23312.819) * [-23271.566] (-23333.160) (-23259.203) (-23276.277) -- 6:20:44
363000 -- (-23274.538) [-23239.905] (-23242.974) (-23299.857) * [-23270.175] (-23299.396) (-23253.613) (-23295.412) -- 6:20:26
363500 -- (-23266.005) (-23233.442) [-23260.318] (-23313.701) * (-23273.860) (-23283.403) [-23254.149] (-23304.646) -- 6:20:05
364000 -- (-23273.940) [-23227.829] (-23249.116) (-23307.294) * (-23273.209) (-23273.784) [-23258.231] (-23298.124) -- 6:19:47
364500 -- (-23291.641) (-23228.243) [-23251.417] (-23311.472) * (-23268.801) (-23267.963) [-23251.039] (-23290.714) -- 6:19:28
365000 -- (-23284.212) [-23215.743] (-23275.336) (-23306.450) * [-23256.221] (-23302.325) (-23266.426) (-23317.150) -- 6:19:06

Average standard deviation of split frequencies: 0.021194

365500 -- (-23306.479) [-23227.830] (-23278.654) (-23316.441) * (-23264.433) (-23284.484) [-23247.609] (-23307.113) -- 6:18:49
366000 -- (-23299.585) [-23226.498] (-23261.692) (-23288.816) * (-23263.634) (-23280.449) [-23248.289] (-23295.483) -- 6:18:29
366500 -- (-23299.842) [-23220.276] (-23260.086) (-23288.072) * (-23260.388) (-23292.693) [-23247.101] (-23279.895) -- 6:18:10
367000 -- (-23298.138) [-23227.106] (-23283.660) (-23273.596) * (-23257.403) (-23297.796) [-23252.850] (-23286.168) -- 6:17:52
367500 -- (-23297.450) [-23237.447] (-23294.432) (-23271.269) * [-23256.960] (-23307.672) (-23247.063) (-23270.488) -- 6:17:32
368000 -- (-23295.522) [-23232.138] (-23290.216) (-23271.888) * (-23266.074) (-23290.442) [-23235.792] (-23269.124) -- 6:17:15
368500 -- (-23285.453) (-23250.231) (-23305.979) [-23267.549] * (-23286.190) (-23320.720) (-23240.667) [-23253.256] -- 6:16:57
369000 -- (-23294.953) (-23249.583) (-23315.267) [-23268.726) * (-23264.111) (-23309.101) (-23244.582) [-23232.909] -- 6:16:39
369500 -- (-23294.552) [-23232.972] (-23304.000) (-23264.129) * [-23252.626) (-23313.733) (-23273.674) [-23230.742] -- 6:16:21
370000 -- (-23321.380) [-23230.483] (-23299.494) (-23266.570) * (-23266.189) (-23329.078) (-23279.240) [-23248.301] -- 6:16:02

Average standard deviation of split frequencies: 0.021353

370500 -- (-23313.674) [-23238.175] (-23306.067) (-23269.624) * [-23280.322] (-23320.783) (-23259.457) (-23245.764) -- 6:15:44
371000 -- (-23311.998) [-23245.613] (-23303.528) (-23262.106) * (-23284.183) (-23298.285) (-23279.731) [-23233.280] -- 6:15:27
371500 -- (-23327.458) [-23242.596] (-23296.469) [-23237.008] * (-23289.780) (-23295.140) [-23269.563] (-23238.837) -- 6:15:10
372000 -- (-23341.984) (-23236.068) (-23292.271) [-23257.812] * (-23289.630) (-23310.726) (-23255.992) [-23237.353] -- 6:14:53
372500 -- (-23305.884) [-23242.231] (-23294.673) (-23238.128) * (-23304.404) (-23291.905) (-23244.842) [-23229.593] -- 6:14:37
373000 -- (-23320.016) [-23245.893] (-23297.607) [-23242.971] * (-23301.138) (-23290.240) (-23275.017) [-23227.137] -- 6:14:19
373500 -- (-23336.604) [-23229.135] (-23303.797) (-23256.226) * (-23281.427) (-23306.371) (-23286.378) [-23234.529] -- 6:14:03
374000 -- (-23326.465) [-23228.242] (-23291.643) (-23251.638) * (-23298.131) (-23312.573) (-23280.226) [-23222.117] -- 6:13:47
374500 -- (-23324.096) [-23221.638] (-23298.600) (-23253.871) * (-23286.618) (-23308.534) (-23302.761) [-23226.367] -- 6:13:31
375000 -- (-23318.114) [-23232.789] (-23294.828) (-23252.915) * (-23274.880) (-23319.527) (-23306.351) [-23220.742] -- 6:13:15

Average standard deviation of split frequencies: 0.021234

375500 -- (-23301.704) [-23224.590] (-23296.057) (-23253.506) * (-23278.137) (-23291.443) (-23283.284) [-23226.930] -- 6:13:00
376000 -- (-23281.507) [-23233.346] (-23332.583) (-23246.693) * (-23269.384) (-23309.626) (-23289.623) [-23225.622] -- 6:12:44
376500 -- (-23273.526) (-23234.627) (-23350.861) [-23252.799] * (-23279.491) (-23294.035) (-23288.074) [-23228.326] -- 6:12:28
377000 -- (-23284.633) (-23251.267) (-23348.925) [-23237.802] * (-23265.965) (-23292.201) (-23284.100) [-23234.927] -- 6:12:13
377500 -- (-23297.783) (-23261.452) (-23349.631) [-23240.499] * (-23259.619) (-23277.045) (-23269.260) [-23227.369] -- 6:11:57
378000 -- (-23286.553) (-23261.199) (-23297.824) [-23232.029] * [-23247.911] (-23284.487) (-23302.201) (-23254.084) -- 6:11:41
378500 -- (-23272.935) (-23267.784) (-23291.460) [-23228.226] * (-23241.504) (-23280.180) (-23307.431) [-23256.525] -- 6:11:23
379000 -- (-23278.459) (-23257.025) (-23294.748) [-23235.174] * [-23240.909] (-23299.290) (-23309.104) (-23265.509) -- 6:11:07
379500 -- (-23275.112) (-23268.439) (-23291.660) [-23249.135] * [-23242.888] (-23311.865) (-23300.567) (-23276.140) -- 6:10:49
380000 -- (-23279.295) (-23277.952) (-23300.375) [-23261.849] * (-23236.003) (-23310.337) (-23297.604) [-23269.124] -- 6:10:31

Average standard deviation of split frequencies: 0.021618

380500 -- (-23281.035) (-23262.114) (-23269.956) [-23244.394] * [-23235.387] (-23324.257) (-23298.025) (-23264.753) -- 6:10:15
381000 -- (-23279.178) (-23268.933) (-23276.349) [-23235.595] * [-23231.310] (-23282.382) (-23320.517) (-23273.248) -- 6:09:57
381500 -- (-23286.848) (-23273.228) (-23280.375) [-23238.377] * [-23236.634] (-23260.497) (-23304.280) (-23289.048) -- 6:09:40
382000 -- (-23285.300) (-23287.194) (-23266.542) [-23231.205] * [-23233.380] (-23254.284) (-23301.529) (-23282.524) -- 6:09:22
382500 -- (-23289.532) (-23271.231) (-23263.528) [-23222.594] * [-23226.339] (-23256.741) (-23327.901) (-23289.357) -- 6:09:06
383000 -- (-23306.296) (-23269.770) (-23241.207) [-23234.015] * [-23221.653] (-23254.437) (-23331.620) (-23288.388) -- 6:08:46
383500 -- (-23324.244) (-23265.654) (-23246.090) [-23222.949] * [-23240.421] [-23243.580] (-23311.536) (-23284.172) -- 6:08:28
384000 -- (-23310.597) (-23264.468) (-23236.941) [-23221.725] * [-23235.389] (-23241.108) (-23283.857) (-23278.071) -- 6:08:10
384500 -- (-23313.662) (-23273.420) (-23263.884) [-23246.804] * (-23257.521) [-23254.816] (-23325.408) (-23281.887) -- 6:07:53
385000 -- (-23300.427) (-23267.406) (-23265.151) [-23259.636] * (-23273.954) (-23266.557) (-23318.159) [-23272.985] -- 6:07:33

Average standard deviation of split frequencies: 0.021512

385500 -- (-23290.671) (-23263.753) (-23270.132) [-23243.732] * (-23278.657) [-23246.379] (-23315.327) (-23253.182) -- 6:07:17
386000 -- (-23308.309) (-23239.959) (-23286.174) (-23234.869) * (-23298.296) (-23240.794) (-23332.032) [-23260.648] -- 6:06:59
386500 -- (-23297.609) [-23231.206] (-23282.165) (-23253.910) * (-23277.181) [-23236.565] (-23325.150) (-23267.052) -- 6:06:41
387000 -- (-23290.552) [-23236.625] (-23282.075) (-23260.609) * (-23296.035) [-23238.909] (-23339.666) (-23257.401) -- 6:06:25
387500 -- (-23311.297) [-23231.296] (-23278.556) (-23257.178) * (-23279.698) [-23251.006] (-23331.648) (-23263.262) -- 6:06:06
388000 -- (-23303.827) [-23225.640] (-23275.366) (-23245.096) * (-23290.993) [-23251.885] (-23326.730) (-23265.412) -- 6:05:48
388500 -- (-23308.548) (-23236.410) (-23280.979) [-23224.321] * (-23308.674) (-23250.730) (-23314.963) [-23258.623] -- 6:05:30
389000 -- (-23305.530) (-23230.457) (-23272.969) [-23232.370] * (-23321.777) (-23251.737) (-23295.218) [-23267.683] -- 6:05:12
389500 -- (-23302.394) (-23235.237) (-23266.008) [-23220.044] * (-23316.975) [-23246.702] (-23288.218) (-23295.521) -- 6:04:53
390000 -- (-23318.407) (-23224.221) (-23276.264) [-23228.455] * (-23317.088) [-23235.045] (-23275.365) (-23305.133) -- 6:04:35

Average standard deviation of split frequencies: 0.021287

390500 -- (-23321.064) (-23241.362) (-23285.818) [-23236.591] * (-23323.802) [-23219.041] (-23275.746) (-23299.382) -- 6:04:16
391000 -- (-23283.806) (-23243.965) (-23280.886) [-23256.754] * (-23334.049) [-23211.666] (-23269.035) (-23289.781) -- 6:03:58
391500 -- (-23279.983) (-23238.382) (-23288.770) (-23270.487) * (-23305.919) [-23227.735] (-23253.667) (-23295.060) -- 6:03:40
392000 -- (-23294.891) [-23227.697] (-23280.590) (-23282.702) * (-23337.418) [-23244.508] (-23269.948) (-23298.995) -- 6:03:21
392500 -- (-23308.911) [-23256.574] (-23291.547) (-23269.968) * (-23336.665) [-23254.198] (-23278.483) (-23265.014) -- 6:03:03
393000 -- (-23336.838) [-23271.321] (-23297.441) (-23306.204) * (-23311.274) [-23259.379] (-23295.820) (-23259.031) -- 6:02:43
393500 -- (-23364.275) [-23260.990] (-23300.144) (-23281.726) * (-23316.346) (-23272.994) (-23293.474) [-23248.467] -- 6:02:24
394000 -- (-23346.975) [-23241.713] (-23275.309) (-23279.713) * (-23315.237) (-23279.697) (-23267.600) [-23242.489] -- 6:02:05
394500 -- (-23352.264) [-23236.536] (-23275.990) (-23280.908) * (-23306.627) (-23287.885) (-23272.201) [-23248.202] -- 6:01:45

```
395000 -- (-23335.485) [-23238.141] (-23294.878) (-23273.522) * (-23292.716) (-23273.772) (-23266.747) [-23239.792] -- 6:01:26

Average standard deviation of split frequencies: 0.021004

395500 -- (-23329.444) [-23249.187] (-23265.120) (-23275.866) * (-23271.417) [-23253.357] (-23270.591) (-23240.763) -- 6:01:08
396000 -- (-23318.031) (-23250.083) [-23257.856] (-23283.484) * (-23270.903) (-23272.056) (-23267.631) [-23253.035] -- 6:00:49
396500 -- (-23310.176) [-23261.700] (-23273.104) (-23287.297) * (-23259.135) (-23273.516) (-23276.847) [-23265.376] -- 6:00:31
397000 -- (-23293.807) [-23255.111] (-23268.238) (-23289.821) * (-23263.754) (-23278.440) (-23269.317) [-23238.904] -- 6:00:12
397500 -- (-23315.706) [-23268.957] (-23276.025) (-23260.166) * (-23263.731) (-23283.328) (-23286.975) [-23244.061] -- 5:59:52
398000 -- (-23305.259) (-23287.063) [-23260.704] (-23284.543) * [-23249.713] (-23264.357) (-23290.165) (-23260.725) -- 5:59:33
398500 -- (-23305.802) (-23288.434) (-23263.457) [-23282.709] * (-23260.709) (-23277.327) (-23270.441) (-23269.194] -- 5:59:14
399000 -- (-23320.056) (-23295.524) [-23264.229] (-23263.058) * [-23246.791] (-23290.241) (-23273.168) (-23269.250) -- 5:58:56
399500 -- (-23314.912) (-23295.567) [-23266.668] [-23251.046] * [-23253.990] (-23275.499) (-23274.018) (-23273.370) -- 5:58:37
400000 -- (-23315.889) (-23295.073) [-23267.520] (-23255.061) * [-23259.999] (-23298.360) (-23272.470) (-23274.108) -- 5:58:19

Average standard deviation of split frequencies: 0.020896

400500 -- (-23296.453) (-23289.771) (-23269.553) [-23239.975] * [-23249.954] (-23297.181) (-23254.392) (-23271.429) -- 5:58:00
401000 -- (-23293.673) (-23290.530) (-23282.708) [-23250.831] * [-23259.328] (-23304.608) (-23273.722) (-23267.574) -- 5:57:42
401500 -- (-23279.449) (-23301.226) (-23318.118) [-23248.725] * [-23246.337] (-23284.384) (-23269.570) (-23257.582) -- 5:57:24
402000 -- (-23267.978) (-23308.123) (-23302.534) [-23231.007] * (-23246.367) (-23276.536) [-23255.235] (-23268.130) -- 5:57:05
402500 -- (-23245.376) (-23312.728) (-23297.563) [-23235.990] * [-23243.575] (-23279.871) (-23250.857) (-23286.920) -- 5:56:47
403000 -- (-23261.527) (-23313.520) (-23305.976) [-23229.375] * [-23234.715] (-23329.313) (-23255.709) (-23300.530) -- 5:56:28
403500 -- (-23268.202) (-23308.071) (-23295.406) [-23216.121] * [-23264.518] (-23305.397) (-23265.609) (-23293.262) -- 5:56:10
404000 -- (-23269.598) (-23272.605) (-23286.929) [-23221.047] * [-23220.611] (-23291.750) (-23265.248) (-23291.867) -- 5:55:52
404500 -- (-23275.537) (-23280.475) (-23285.794) [-23251.074] * [-23222.856] (-23287.577) (-23241.079) (-23296.679) -- 5:55:33
405000 -- (-23270.008) (-23298.438) (-23301.347) [-23255.745] * [-23223.354] (-23277.073) (-23251.900) (-23274.753) -- 5:55:15

Average standard deviation of split frequencies: 0.020473

405500 -- [-23257.872] (-23283.956) (-23297.227) (-23255.214) * [-23224.985] (-23285.286) (-23275.595) (-23281.215) -- 5:54:57
406000 -- [-23237.919] (-23275.138) (-23286.780) (-23249.594) * [-23245.933] (-23285.202) (-23281.043) (-23302.392) -- 5:54:40
406500 -- [-23235.646] (-23280.966) (-23310.828) (-23268.946) * [-23245.334] (-23274.349) (-23296.049) (-23328.419) -- 5:54:22
407000 -- (-23254.803) (-23297.911) (-23297.093) [-23257.632] * [-23230.761] (-23264.007) (-23299.613) (-23315.298) -- 5:54:03
407500 -- [-23263.858] (-23313.621) (-23326.165) (-23249.239) * [-23224.051] [-23245.443] (-23289.594) (-23316.517) -- 5:53:45
408000 -- [-23240.840] (-23317.635) (-23350.424) (-23263.101) * [-23231.988] (-23257.482) (-23300.555) (-23294.413) -- 5:53:26
408500 -- [-23258.315] (-23299.559) (-23372.552) [-23263.713] * [-23229.088] (-23245.983) (-23298.571) (-23293.872) -- 5:53:08
409000 -- [-23246.338] (-23305.831) (-23351.785) (-23264.093) * [-23249.583] (-23264.078) (-23296.061) (-23304.819) -- 5:52:50
409500 -- (-23253.273) (-23294.677) (-23365.060) [-23256.556] * [-23250.255] (-23253.847) (-23284.300) (-23295.506) -- 5:52:31
410000 -- (-23257.077) (-23294.144) (-23345.596) [-23249.945] * [-23266.938] (-23272.900) (-23284.799) (-23273.499) -- 5:52:12

Average standard deviation of split frequencies: 0.020643

410500 -- (-23257.319) (-23285.533) (-23337.335) [-23255.865] * [-23261.891] (-23286.466) (-23297.104) (-23306.128) -- 5:51:54
411000 -- [-23256.295] (-23282.643) (-23302.473) (-23270.720) * [-23266.175] (-23287.378) (-23288.876) (-23310.585) -- 5:51:35
411500 -- (-23252.481) (-23281.171) (-23311.699) [-23254.169] * [-23273.085] (-23280.510) (-23309.246) (-23305.195) -- 5:51:17
412000 -- [-23262.487] (-23263.522) (-23310.840) (-23265.431) * [-23274.304] (-23268.722) (-23294.294) (-23317.034) -- 5:50:58
412500 -- [-23244.692] (-23255.813) (-23337.542) (-23284.167) * [-23255.213] (-23255.608) (-23286.887) (-23293.304) -- 5:50:38
413000 -- [-23248.194] (-23256.782) (-23319.825) (-23278.284) * [-23269.732] (-23246.607) (-23294.641) (-23291.113) -- 5:50:19
413500 -- [-23246.740] (-23288.580) (-23305.654) (-23291.671) * (-23267.730) [-23229.433] (-23289.451) (-23279.492) -- 5:50:01
414000 -- [-23242.673] (-23297.864) (-23309.904) (-23273.617) * (-23255.774) [-23219.794] (-23296.468) (-23268.982) -- 5:49:44
414500 -- [-23225.072] (-23291.710) (-23310.603) (-23272.925) * (-23278.369) [-23222.303] (-23293.195) (-23255.921) -- 5:49:24
415000 -- [-23237.335] (-23298.726) (-23317.418) (-23275.005) * (-23284.571) [-23211.756] (-23303.235) (-23241.374) -- 5:49:07

Average standard deviation of split frequencies: 0.020382

415500 -- [-23247.386] (-23281.521) (-23326.111) (-23278.858) * (-23285.757) [-23233.846] (-23303.442) (-23253.049) -- 5:48:49
416000 -- [-23230.144] (-23289.024) (-23326.948) (-23302.991) * (-23287.183) [-23228.855] (-23305.867) (-23238.554) -- 5:48:31
416500 -- [-23234.304] (-23278.529) (-23305.871) (-23312.856) * (-23276.598) [-23247.141] (-23306.704) (-23259.298) -- 5:48:13
417000 -- [-23246.069] (-23257.948) (-23297.417) (-23304.137) * (-23274.256) [-23248.581] (-23297.989) (-23283.827) -- 5:47:54
417500 -- (-23255.197) (-23265.125) (-23295.541) (-23297.623) * (-23278.515) [-23252.004] (-23287.880) (-23282.408) -- 5:47:36
418000 -- [-23247.864] (-23274.006) (-23273.725) (-23303.984) * [-23273.624] (-23250.920) (-23300.610) (-23269.793) -- 5:47:17
418500 -- [-23244.092] (-23278.299) (-23273.959) (-23319.879) * (-23289.369) [-23228.086] (-23297.558) (-23291.241) -- 5:47:00
419000 -- [-23239.632] (-23273.692) (-23273.649) (-23307.227) * [-23282.138] (-23239.683) (-23292.933) (-23288.526) -- 5:46:40
419500 -- [-23245.877] (-23286.863) (-23259.215) (-23307.918) * (-23292.542) [-23249.750] (-23304.399) (-23276.794) -- 5:46:23
420000 -- [-23256.060] (-23284.620) (-23271.738) (-23309.443) * (-23285.696) [-23239.346] (-23293.421) (-23260.641) -- 5:46:05

Average standard deviation of split frequencies: 0.020556

420500 -- (-23257.724) (-23295.143) [-23269.562] (-23316.124) * (-23285.324) [-23249.057] (-23285.354) (-23267.708) -- 5:45:46
421000 -- (-23241.778) (-23319.125) [-23281.473] (-23292.510) * (-23287.877) [-23235.227] (-23277.543) (-23311.492) -- 5:45:28
421500 -- [-23231.783] (-23306.255) (-23281.293) (-23281.182) * (-23280.966) [-23224.208] (-23266.254) (-23309.129) -- 5:45:09
422000 -- [-23210.899] (-23289.072) (-23285.091) (-23283.398) * (-23286.980) [-23212.760] (-23281.866) (-23329.331) -- 5:44:51
422500 -- [-23211.567] (-23289.178) (-23272.796) (-23272.282) * (-23282.717) [-23213.958] (-23310.197) (-23325.441) -- 5:44:33
423000 -- [-23215.527] (-23300.580) (-23259.177) (-23283.570) * (-23277.826) [-23209.659] (-23319.188) (-23302.417) -- 5:44:16
423500 -- [-23228.343] (-23304.038) (-23251.173) (-23302.495) * (-23278.965) [-23214.728] (-23315.145) (-23296.191) -- 5:43:58
424000 -- (-23235.393) (-23309.892) [-23251.153] (-23310.935) * (-23271.329) [-23225.282] (-23302.694) (-23300.203) -- 5:43:39
424500 -- [-23250.890] (-23301.806) (-23245.156) (-23306.743) * (-23272.877) [-23229.078] (-23302.930) (-23303.177) -- 5:43:21
425000 -- (-23254.362) (-23309.299) [-23242.679] (-23297.471) * [-23241.584] (-23221.339) (-23298.507) (-23301.576) -- 5:43:03

Average standard deviation of split frequencies: 0.020941

425500 -- (-23283.139) (-23342.859) [-23254.080] (-23303.404) * (-23245.333) [-23231.144] (-23308.477) (-23309.270) -- 5:42:44
426000 -- (-23281.315) (-23330.881) [-23257.239] (-23288.516) * (-23274.005) [-23233.230] (-23320.559) (-23323.535) -- 5:42:26
426500 -- (-23257.321) (-23331.124) [-23248.744] (-23281.826) * (-23276.710) [-23235.007] (-23330.105) (-23309.061) -- 5:42:09
427000 -- [-23258.609] (-23310.215) (-23261.773) (-23291.325) * (-23283.300) [-23244.843] (-23328.956) (-23307.080) -- 5:41:51
427500 -- (-23245.968) (-23301.401) [-23257.740] (-23267.813) * (-23267.675) [-23239.155] (-23328.582) (-23305.156) -- 5:41:32
428000 -- (-23236.410) (-23302.101) [-23258.807] (-23289.587) * (-23273.169) [-23233.583] (-23324.389) (-23317.637) -- 5:41:13
428500 -- (-23268.027) (-23310.383) [-23259.104] (-23290.748) * (-23278.395) [-23238.945] (-23319.961) (-23332.996) -- 5:40:53
429000 -- (-23272.699) (-23300.022) [-23256.893] (-23303.532) * (-23281.992) [-23248.327] (-23331.070) (-23330.464) -- 5:40:36
429500 -- (-23294.505) (-23319.311) [-23260.028] (-23323.708) * (-23250.274) [-23231.280] (-23327.498) (-23316.743) -- 5:40:17
430000 -- (-23294.478) (-23315.654) [-23243.133] (-23294.159) * [-23239.363] (-23222.548) (-23317.405) (-23322.458) -- 5:39:58

Average standard deviation of split frequencies: 0.021086

430500 -- (-23302.831) (-23317.389) [-23224.428] (-23286.168) * [-23245.042] (-23255.881) (-23320.863) (-23302.466) -- 5:39:38
431000 -- (-23310.026) (-23296.678) [-23235.504] (-23276.523) * (-23232.250) [-23269.743] (-23323.287) (-23302.791) -- 5:39:19
431500 -- (-23303.096) (-23296.259) [-23243.031] (-23267.608) * [-23244.364] (-23256.326) (-23322.292) (-23309.171) -- 5:39:00
432000 -- (-23282.852) (-23321.293) [-23258.686] (-23271.916) * (-23237.086) [-23249.744] (-23310.332) (-23285.259) -- 5:38:43
432500 -- (-23267.612) (-23304.159) [-23240.682] (-23263.003) * [-23248.503] (-23244.580) (-23296.129) (-23310.303) -- 5:38:24
433000 -- (-23274.201) (-23312.398) [-23231.963] (-23302.026) * [-23238.587] (-23258.302) (-23293.620) (-23343.520) -- 5:38:06
433500 -- (-23289.875) (-23322.598) [-23231.660] (-23285.947) * [-23229.594] (-23250.635) (-23284.837) (-23338.940) -- 5:37:47
434000 -- (-23274.440) (-23301.467) [-23233.454] (-23265.219) * [-23222.131] (-23262.004) (-23283.090) (-23323.836) -- 5:37:29
434500 -- (-23266.019) (-23286.915) [-23252.792] (-23279.022) * (-23228.203) [-23253.464] (-23281.270) (-23334.382) -- 5:37:11
435000 -- (-23276.193) (-23292.231) [-23264.401] (-23290.122) * (-23237.430) [-23237.421] (-23275.047) (-23330.022) -- 5:36:54

Average standard deviation of split frequencies: 0.021091
```

```
435500 -- (-23284.034) (-23299.306) [-23246.094] (-23286.079) * (-23249.136) [-23235.654] (-23280.391) (-23305.295) -- 5:36:34
436000 -- (-23295.015) (-23288.553) [-23246.449] (-23272.311) * (-23244.809) [-23227.588] (-23268.300) (-23325.112) -- 5:36:17
436500 -- (-23302.278) (-23308.161) [-23251.600] (-23283.390) * (-23255.003) [-23237.516] (-23267.345) (-23318.875) -- 5:35:59
437000 -- (-23298.090) (-23318.858) [-23272.623] (-23273.291) * (-23242.892) [-23230.132] (-23269.467) (-23310.574) -- 5:35:41
437500 -- [-23282.182] (-23322.690) (-23278.174) (-23266.009) * [-23237.005] (-23246.530) (-23269.937) (-23315.092) -- 5:35:25
438000 -- (-23301.842) (-23342.843) [-23266.993] (-23253.989) * [-23235.832] (-23237.511) (-23258.070) (-23311.542) -- 5:35:07
438500 -- (-23280.834) (-23333.291) (-23265.262) [-23250.139] * (-23247.520) [-23238.317] (-23259.298) (-23311.525) -- 5:34:51
439000 -- (-23283.141) (-23333.004) (-23266.976) [-23247.651] * (-23261.354) [-23235.633] (-23255.909) (-23333.471) -- 5:34:33
439500 -- (-23272.775) (-23348.054) (-23283.904) [-23239.140] * (-23273.346) [-23218.233] (-23275.513) (-23329.087) -- 5:34:15
440000 -- (-23269.531) (-23352.602) (-23290.764) [-23237.396] * (-23274.467) [-23233.488] (-23263.455) (-23312.550) -- 5:33:59

Average standard deviation of split frequencies: 0.021140

440500 -- (-23298.925) (-23338.312) (-23288.481) [-23242.535] * (-23280.705) [-23233.918] (-23260.505) (-23297.018) -- 5:33:41
441000 -- (-23308.822) (-23337.747) (-23275.165) [-23227.755] * (-23285.978) [-23225.095] (-23286.062) (-23304.207) -- 5:33:24
441500 -- (-23316.298) (-23319.759) (-23265.816) [-23238.814] * (-23265.157) [-23231.991] (-23280.604) (-23312.987) -- 5:33:07
442000 -- (-23297.053) (-23298.675) [-23267.593] (-23243.102) * (-23264.226) [-23240.427] (-23294.084) (-23331.602) -- 5:32:49
442500 -- (-23273.283) (-23320.214) (-23257.987) [-23256.946] * (-23259.131) [-23244.221] (-23304.965) (-23318.424) -- 5:32:31
443000 -- (-23300.134) (-23302.973) [-23261.826] (-23261.655) * (-23253.185) [-23243.086] (-23295.279) (-23312.521) -- 5:32:13
443500 -- (-23284.919) (-23301.569) [-23248.888] (-23258.988) * (-23246.801) [-23234.792] (-23294.121) (-23310.643) -- 5:31:56
444000 -- (-23288.957) (-23321.959) [-23245.075] (-23265.539) * (-23257.950) [-23231.775] (-23305.128) (-23321.913) -- 5:31:37
444500 -- (-23284.338) (-23328.776) [-23249.543] (-23248.092) * (-23254.897) [-23237.202] (-23299.898) (-23305.517) -- 5:31:18
445000 -- (-23303.291) (-23324.977) (-23258.491) [-23247.297] * (-23261.267) [-23241.248] (-23299.972) (-23298.305) -- 5:31:00

Average standard deviation of split frequencies: 0.021227

445500 -- (-23318.740) (-23322.607) (-23266.175) [-23259.031] * [-23272.495] (-23245.749) (-23303.526) (-23301.478) -- 5:30:41
446000 -- (-23303.909) (-23323.889) [-23259.223] (-23268.319) * [-23281.797] (-23246.253) (-23311.538) (-23306.731) -- 5:30:23
446500 -- (-23310.247) (-23335.701) [-23262.320] (-23279.659) * (-23294.529) [-23251.957] (-23307.426) (-23292.934) -- 5:30:04
447000 -- (-23290.220) (-23346.399) [-23253.115] (-23277.673) * (-23275.659) [-23248.606] (-23284.150) (-23273.001) -- 5:29:46
447500 -- (-23273.713) (-23335.855) [-23230.386] (-23293.666) * (-23283.477) [-23252.172] (-23302.187) (-23277.839) -- 5:29:29
448000 -- (-23260.205) (-23302.175) [-23232.714] (-23310.360) * (-23280.000) [-23248.873] (-23301.338) (-23260.271) -- 5:29:10
448500 -- (-23271.886) (-23300.594) [-23230.932] (-23309.012) * (-23287.427) [-23236.587] (-23327.144) (-23267.086) -- 5:28:51
449000 -- (-23270.562) (-23305.624) [-23243.309] (-23305.437) * (-23300.638) [-23248.219] (-23313.699) (-23270.319) -- 5:28:32
449500 -- (-23255.593) (-23307.001) [-23243.696] (-23307.702) * (-23294.257) [-23232.243] (-23315.711) (-23275.425) -- 5:28:14
450000 -- (-23260.952) (-23302.123) [-23232.793] (-23317.708) * (-23290.574) [-23233.164] (-23305.244) (-23280.745) -- 5:27:55

Average standard deviation of split frequencies: 0.021994

450500 -- (-23272.105) (-23288.028) [-23258.016] (-23320.632) * (-23310.706) [-23231.305] (-23322.484) (-23267.633) -- 5:27:37
451000 -- (-23268.448) (-23296.580) [-23269.707] (-23310.813) * (-23305.536) [-23234.125] (-23317.534) (-23263.770) -- 5:27:18
451500 -- (-23273.806) (-23281.738) [-23254.277] (-23287.585) * (-23303.384) [-23253.698] (-23324.925) (-23284.347) -- 5:26:59
452000 -- (-23261.117) (-23311.831) [-23255.555] (-23299.065) * (-23296.255) [-23245.332] (-23341.420) (-23284.204) -- 5:26:40
452500 -- (-23269.658) (-23290.999) [-23253.645] (-23312.351) * (-23276.411) [-23237.196] (-23329.545) (-23308.582) -- 5:26:22
453000 -- (-23273.351) (-23293.619) [-23239.303] (-23305.369) * (-23285.053) [-23252.746] (-23327.895) (-23283.742) -- 5:26:04
453500 -- (-23271.890) (-23315.698) [-23235.444] (-23269.950) * (-23295.597) [-23251.872] (-23317.974) (-23275.222) -- 5:25:45
454000 -- (-23269.802) (-23308.192) [-23248.698] (-23288.753) * (-23281.080) [-23258.235] (-23310.422) (-23272.725) -- 5:25:26
454500 -- [-23257.852] (-23297.445) (-23245.342) (-23274.399) * (-23263.533) [-23273.888] (-23325.973) (-23276.204) -- 5:25:08
455000 -- (-23263.900) (-23293.527) [-23245.242] (-23286.844) * [-23254.275] (-23268.312) (-23339.385) (-23261.214) -- 5:24:49

Average standard deviation of split frequencies: 0.022579

455500 -- (-23262.900) (-23319.266) [-23246.456] (-23275.673) * [-23261.525] (-23269.029) (-23338.365) (-23276.890) -- 5:24:31
456000 -- (-23258.773) (-23316.868) [-23225.261] (-23307.752) * [-23244.415] (-23248.783) (-23338.551) (-23274.451) -- 5:24:12
456500 -- [-23259.320] (-23306.577) (-23245.600) (-23293.511) * (-23245.102) [-23252.463] (-23328.394) (-23279.164) -- 5:23:53
457000 -- (-23258.374) (-23299.000) [-23249.765] (-23293.970) * (-23249.414) [-23238.032] (-23325.076) (-23294.750) -- 5:23:34
457500 -- (-23263.140) (-23294.084) [-23247.136] (-23290.558) * (-23249.899) [-23231.162] (-23295.527) (-23283.396) -- 5:23:16
458000 -- (-23270.297) (-23278.506) [-23234.737] (-23288.176) * (-23241.549) [-23224.423] (-23295.083) (-23274.358) -- 5:22:57
458500 -- (-23253.154) (-23300.109) [-23234.712] (-23299.832) * [-23244.411] (-23231.876) (-23302.655) (-23258.958) -- 5:22:38
459000 -- (-23256.578) (-23306.238) [-23237.921] (-23293.549) * (-23273.105) [-23240.962] (-23301.105) (-23275.630) -- 5:22:20
459500 -- (-23252.333) (-23331.409) [-23247.948] (-23307.771) * (-23278.636) [-23242.118] (-23286.958) (-23299.834) -- 5:22:02
460000 -- (-23260.010) (-23310.424) [-23243.464] (-23288.813) * (-23270.553) [-23235.962] (-23289.499) (-23291.725) -- 5:21:45

Average standard deviation of split frequencies: 0.023255

460500 -- (-23251.468) (-23326.349) [-23237.282] (-23282.834) * (-23276.019) [-23230.324] (-23285.842) (-23297.451) -- 5:21:28
461000 -- [-23257.433] (-23338.372) (-23226.364) (-23301.073) * (-23287.194) [-23217.681] (-23266.645) (-23308.020) -- 5:21:10
461500 -- (-23264.490) (-23324.464) [-23223.956] (-23328.129) * (-23276.723) [-23232.541] (-23269.917) (-23295.004) -- 5:20:52
462000 -- (-23260.399) (-23315.282) [-23226.719] (-23324.272) * (-23270.293) [-23249.949] (-23266.236) (-23282.652) -- 5:20:34
462500 -- (-23260.005) (-23313.914) [-23231.033] (-23311.776) * (-23260.025) [-23241.685] (-23260.746) (-23283.767) -- 5:20:16
463000 -- (-23267.317) (-23294.547) [-23242.128] (-23299.762) * (-23278.038) [-23228.842] (-23242.828) (-23295.369) -- 5:19:57
463500 -- (-23262.161) (-23303.136) [-23242.413] (-23302.934) * (-23261.292) [-23230.253] (-23251.877) (-23295.401) -- 5:19:39
464000 -- (-23259.696) (-23309.388) [-23245.332] (-23325.007) * (-23251.962) [-23236.431] (-23261.882) (-23289.667) -- 5:19:20
464500 -- [-23253.082] (-23318.056) (-23269.834) (-23294.096) * [-23243.858] (-23270.222) (-23254.902) (-23291.867) -- 5:19:03
465000 -- [-23251.501] (-23295.200) (-23287.748) (-23286.129) * [-23240.772] (-23267.142) (-23257.014) (-23309.992) -- 5:18:44

Average standard deviation of split frequencies: 0.023635

465500 -- (-23244.964) (-23308.391) [-23287.566] (-23276.722) * (-23245.356) (-23273.325) [-23265.779] (-23315.675) -- 5:18:25
466000 -- [-23253.777] (-23298.016) (-23298.647) (-23271.034) * [-23245.580] (-23272.866) (-23258.552) (-23323.997) -- 5:18:07
466500 -- [-23253.908] (-23302.820) (-23310.468) (-23272.743) * [-23269.222] (-23268.900) (-23275.660) (-23332.080) -- 5:17:48
467000 -- [-23245.714] (-23288.813) (-23296.191) (-23282.642) * [-23244.497] (-23273.496) (-23270.460) (-23335.430) -- 5:17:31
467500 -- [-23245.899] (-23313.122) (-23288.211) (-23282.929) * [-23251.295] (-23273.945) [-23265.763] (-23325.030) -- 5:17:13
468000 -- [-23237.252] (-23328.645) (-23279.048) (-23278.953) * [-23253.940] (-23289.509) (-23276.388) (-23300.822) -- 5:16:54
468500 -- [-23239.632] (-23336.572) (-23263.788) (-23288.093) * [-23256.908] (-23276.326) (-23262.380) (-23302.352) -- 5:16:36
469000 -- (-23255.619) (-23333.787) [-23265.612] (-23291.374) * (-23277.168) (-23297.609) [-23246.538] (-23306.055) -- 5:16:17
469500 -- [-23246.510] (-23309.509) (-23275.863) (-23288.762) * (-23290.756) (-23285.361) [-23234.575] (-23305.899) -- 5:16:00
470000 -- [-23256.586] (-23315.178) (-23256.654) (-23282.656) * (-23301.229) (-23274.101) [-23238.884] (-23319.346) -- 5:15:42

Average standard deviation of split frequencies: 0.023724

470500 -- (-23261.687) (-23329.094) [-23247.623] (-23287.115) * (-23298.667) [-23241.309] (-23238.469) (-23319.811) -- 5:15:23
471000 -- (-23260.977) (-23316.130) [-23257.713] (-23283.603) * (-23291.586) [-23245.966] (-23248.046) (-23325.579) -- 5:15:05
471500 -- (-23265.958) (-23305.080) [-23250.711] (-23289.358) * (-23270.540) (-23262.520) [-23232.401] (-23327.789) -- 5:14:48
472000 -- (-23255.005) (-23322.707) [-23251.782] (-23289.722) * (-23270.914) [-23252.660] (-23245.136) (-23299.076) -- 5:14:31
472500 -- (-23251.376) (-23303.203) [-23240.683] (-23293.367) * [-23256.388] (-23259.187) (-23246.501) (-23311.584) -- 5:14:13
473000 -- (-23258.796) (-23286.263) [-23237.439] (-23320.543) * (-23263.264) (-23253.708) [-23240.853] (-23323.233) -- 5:13:56
473500 -- [-23251.500] (-23290.056) (-23232.084) (-23315.501) * (-23239.524) (-23262.871) [-23245.354] (-23334.371) -- 5:13:38
474000 -- (-23256.869) (-23296.842) [-23232.538] (-23326.503) * [-23250.151] (-23271.035) (-23258.727) (-23327.054) -- 5:13:20
474500 -- [-23256.709] (-23305.468) (-23268.384) (-23316.726) * (-23274.129) (-23285.944) [-23247.460] (-23319.595) -- 5:13:02
475000 -- [-23254.273] (-23285.374) (-23282.666) (-23336.738) * (-23276.109) (-23301.702) [-23229.081] (-23315.477) -- 5:12:45

Average standard deviation of split frequencies: 0.023614

475500 -- [-23261.760] (-23301.210) (-23260.926) (-23320.463) * (-23272.952) (-23278.935) [-23233.308] (-23339.241) -- 5:12:27
476000 -- [-23261.620] (-23306.682) (-23255.984) (-23309.976) * (-23269.308) (-23304.247) [-23238.853] (-23308.005) -- 5:12:09
476500 -- (-23274.974) (-23300.329) [-23255.699] (-23334.774) * [-23257.551] (-23290.458) (-23256.487) (-23305.560) -- 5:11:50
477000 -- (-23282.812) (-23279.750) [-23242.656] (-23332.582) * [-23254.303] (-23292.434) (-23255.088) (-23285.020) -- 5:11:33
```

```
477500 -- (-23276.119) (-23266.351) [-23237.897] (-23344.060) * (-23255.759) (-23299.564) [-23243.398] (-23279.253) -- 5:11:15
478000 -- (-23283.432) (-23303.765) [-23231.272] (-23336.880) * [-23245.366] (-23297.866) (-23222.534) (-23283.047) -- 5:10:56
478500 -- (-23280.705) (-23298.181) [-23244.409] (-23330.742) * [-23238.610] (-23301.392) (-23238.924) (-23283.597) -- 5:10:38
479000 -- (-23281.435) (-23290.116) [-23240.187] (-23311.909) * [-23250.157] (-23293.361) (-23247.301) (-23320.333) -- 5:10:21
479500 -- (-23279.193) (-23293.662) [-23246.996] (-23322.539) * [-23241.776] (-23279.428) (-23249.620) (-23306.659) -- 5:10:03
480000 -- (-23276.543) (-23291.053) [-23243.568] (-23292.228) * [-23227.227] (-23300.240) (-23254.342) (-23304.439) -- 5:09:45

Average standard deviation of split frequencies: 0.023589

480500 -- (-23263.327) (-23276.711) [-23240.340] (-23319.588) * [-23217.012] (-23287.678) (-23279.164) (-23314.876) -- 5:09:27
481000 -- (-23276.845) (-23295.034) [-23227.077] (-23332.097) * [-23213.447] (-23281.644) (-23271.018) (-23316.916) -- 5:09:10
481500 -- (-23259.915) (-23303.873) [-23244.426] (-23302.916) * [-23227.099] (-23273.638) (-23275.127) (-23316.644) -- 5:08:52
482000 -- (-23267.259) (-23300.866) [-23254.438] (-23307.805) * [-23227.171] (-23260.946) (-23270.453) (-23326.617) -- 5:08:35
482500 -- (-23262.913) (-23305.760) [-23256.231] (-23329.156) * [-23225.758] (-23264.123) (-23255.401) (-23340.685) -- 5:08:17
483000 -- (-23263.021) (-23296.600) [-23269.524] (-23329.659) * [-23235.925] (-23288.686) (-23251.953) (-23352.679) -- 5:08:00
483500 -- (-23254.091) (-23294.725) [-23258.527] (-23318.955) * [-23244.132] (-23285.541) (-23256.146) (-23352.578) -- 5:07:41
484000 -- [-23238.674] (-23293.386) (-23262.623) (-23325.974) * [-23247.184] (-23275.617) (-23269.943) (-23345.324) -- 5:07:22
484500 -- [-23238.613] (-23287.563) (-23264.265) (-23299.069) * [-23245.089] (-23293.218) (-23271.141) (-23316.506) -- 5:07:05
485000 -- [-23235.194] (-23290.880) (-23250.461) (-23294.746) * [-23230.472] (-23290.133) (-23270.261) (-23326.802) -- 5:06:47

Average standard deviation of split frequencies: 0.023699

485500 -- [-23234.498] (-23302.816) (-23247.085) (-23299.089) * [-23241.151] (-23272.992) (-23280.436) (-23331.067) -- 5:06:29
486000 -- [-23242.407] (-23307.741) (-23264.628) (-23304.522) * [-23238.037] (-23263.571) (-23297.412) (-23326.529) -- 5:06:11
486500 -- (-23282.764) (-23288.391) [-23266.900] (-23281.319) * [-23233.752] (-23267.473) (-23312.304) (-23313.161) -- 5:05:54
487000 -- (-23270.463) (-23293.207) [-23255.644] (-23332.964) * [-23228.747] (-23260.873) (-23316.181) (-23306.508) -- 5:05:36
487500 -- (-23290.414) (-23289.908) [-23255.675] (-23318.951) * [-23226.540] (-23274.260) (-23318.977) (-23307.960) -- 5:05:19
488000 -- (-23295.876) (-23273.255) [-23259.114] (-23320.595) * [-23242.102] (-23268.236) (-23321.318) (-23298.968) -- 5:05:01
488500 -- (-23284.628) (-23284.584) [-23257.602] (-23318.388) * [-23223.216] (-23277.399) (-23318.381) (-23324.004) -- 5:04:44
489000 -- (-23312.625) (-23275.930) [-23240.412] (-23292.515) * [-23238.498] (-23274.541) (-23303.921) (-23322.625) -- 5:04:26
489500 -- (-23324.545) [-23250.111] (-23249.796) (-23286.515) * [-23238.369] (-23273.518) (-23299.333) (-23336.262) -- 5:04:08
490000 -- (-23336.405) (-23236.355) [-23236.227] (-23282.986) * [-23238.073] (-23276.482) (-23290.045) (-23322.740) -- 5:03:50

Average standard deviation of split frequencies: 0.024149

490500 -- (-23334.904) (-23241.916) [-23236.776] (-23290.291) * [-23227.003] (-23268.028) (-23295.766) (-23314.931) -- 5:03:32
491000 -- (-23350.542) (-23232.881) [-23243.934] (-23275.874) * [-23232.624] (-23291.669) (-23297.370) (-23285.232) -- 5:03:14
491500 -- (-23330.403) [-23230.176] (-23247.949) (-23264.900) * [-23231.117] (-23315.826) (-23298.045) (-23276.143) -- 5:02:56
492000 -- (-23344.034) [-23242.445] (-23263.679) (-23273.006) * [-23229.806] (-23312.242) (-23294.259) (-23284.523) -- 5:02:37
492500 -- (-23350.582) [-23233.281] (-23252.421) (-23280.525) * [-23243.828] (-23309.439) (-23277.885) (-23306.153) -- 5:02:20
493000 -- (-23358.858) [-23230.833] (-23234.393) (-23291.217) * [-23252.991] (-23313.018) (-23289.178) (-23305.268) -- 5:02:01
493500 -- (-23334.605) [-23212.125] (-23227.014) (-23296.612) * [-23260.593] (-23310.377) (-23282.666) (-23295.993) -- 5:01:42
494000 -- (-23327.526) [-23210.896] [-23229.758] (-23279.593) * [-23258.218] (-23316.929) [-23269.900] (-23298.248) -- 5:01:24
494500 -- (-23316.722) (-23240.030) [-23217.041] (-23272.693) * [-23254.074] (-23300.655) (-23266.616) (-23307.828) -- 5:01:06
495000 -- (-23325.859) (-23243.377) [-23199.899] (-23276.375) * (-23228.959) (-23273.349) [-23261.962] (-23291.525) -- 5:00:48

Average standard deviation of split frequencies: 0.024610

495500 -- (-23327.892) (-23241.813) [-23218.783] (-23288.777) * [-23232.935] (-23275.263) (-23270.274) (-23300.387) -- 5:00:30
496000 -- (-23320.599) (-23235.902) [-23222.910] (-23289.686) * [-23223.937] (-23274.720) (-23268.410) (-23296.744) -- 5:00:11
496500 -- (-23305.306) (-23240.412) [-23222.181] (-23313.269) * [-23222.533] (-23276.780) (-23267.232) (-23293.463) -- 4:59:54
497000 -- (-23286.064) [-23225.552] (-23232.047) (-23314.437) * [-23218.145] (-23277.527) (-23278.929) (-23269.107) -- 4:59:36
497500 -- (-23295.202) [-23237.440] (-23245.269) (-23319.178) * [-23223.856] (-23278.293) (-23303.708) (-23274.983) -- 4:59:18
498000 -- (-23291.416) [-23226.192] (-23251.587) (-23307.635) * [-23221.816] (-23274.573) (-23298.532) (-23273.482) -- 4:59:00
498500 -- (-23299.747) [-23223.410] (-23243.741) (-23291.437) * [-23218.861] (-23287.146) (-23315.400) (-23255.448) -- 4:58:42
499000 -- (-23324.138) (-23241.970) [-23239.247] (-23282.641) * [-23224.347] (-23303.667) (-23317.722) (-23268.410) -- 4:58:24
499500 -- (-23338.618) [-23234.483] (-23240.097) (-23325.903) * [-23220.514] (-23317.416) (-23289.586) (-23259.195) -- 4:58:06
500000 -- (-23307.762) [-23220.704] (-23241.806) (-23320.658) * [-23224.464] (-23303.556) (-23286.365) (-23261.243) -- 4:57:48

Average standard deviation of split frequencies: 0.024887

500500 -- (-23279.868) (-23224.505) [-23247.884] (-23308.701) * [-23227.289] (-23313.184) (-23319.993) (-23288.774) -- 4:57:30
501000 -- (-23259.231) [-23221.816] (-23253.932) (-23304.741) * [-23242.760] (-23312.431) (-23346.766) (-23291.918) -- 4:57:12
501500 -- (-23250.179) [-23220.306] (-23262.098) (-23307.752) * [-23239.038] (-23300.032) (-23348.648) (-23280.332) -- 4:56:53
502000 -- (-23277.817) [-23222.881] (-23278.677) (-23313.124) * [-23231.648] (-23319.226) (-23360.015) (-23289.871) -- 4:56:36
502500 -- (-23277.543) [-23233.227] (-23269.555) (-23286.178) * [-23237.299] (-23312.258) (-23337.913) (-23292.632) -- 4:56:17
503000 -- (-23286.560) [-23243.166] (-23291.545) (-23296.862) * [-23242.289] (-23300.507) (-23305.056) (-23291.007) -- 4:55:59
503500 -- (-23306.274) [-23239.040] (-23280.922) (-23299.351) * [-23261.042] (-23288.138) (-23296.833) (-23298.900) -- 4:55:41
504000 -- (-23292.348) [-23243.230] (-23272.568) (-23313.177) * [-23257.882] (-23292.358) (-23297.929) (-23284.581) -- 4:55:24
504500 -- (-23298.737) [-23224.343] (-23256.730) (-23302.553) * [-23257.534] (-23289.969) (-23313.648) (-23293.757) -- 4:55:06
505000 -- (-23307.791) [-23217.454] (-23255.902) (-23281.405) * [-23258.896] (-23257.254) (-23297.870) (-23311.655) -- 4:54:48

Average standard deviation of split frequencies: 0.025154

505500 -- (-23310.675) [-23236.224] (-23276.576) (-23290.593) * (-23257.510) [-23257.056] (-23298.277) (-23300.696) -- 4:54:30
506000 -- (-23323.339) [-23229.324] (-23269.499) (-23295.443) * (-23266.837) [-23258.384] (-23281.508) (-23296.473) -- 4:54:14
506500 -- (-23323.003) [-23226.330] [-23257.277] (-23287.909) * (-23273.838) [-23259.961] (-23295.001) (-23318.700) -- 4:53:56
507000 -- (-23320.016) [-23242.952] [-23256.197] (-23285.682) * [-23239.845] (-23254.790) (-23286.852) (-23323.353) -- 4:53:37
507500 -- (-23319.533) [-23223.793] (-23250.585) (-23282.258) * [-23257.572] (-23240.327) (-23309.066) (-23316.818) -- 4:53:19
508000 -- (-23333.614) [-23214.684] (-23272.480) (-23287.018) * [-23256.525] (-23235.158) (-23308.817) (-23320.244) -- 4:53:02
508500 -- (-23330.661) [-23216.839] (-23287.921) (-23289.622) * [-23266.269] (-23247.502) (-23305.328) (-23330.822) -- 4:52:43
509000 -- (-23334.668) [-23218.501] (-23281.330) (-23299.644) * [-23262.085] (-23236.932) (-23299.955) (-23322.524) -- 4:52:25
509500 -- (-23313.778) [-23226.407] (-23273.324) (-23302.761) * [-23275.889] (-23243.937) (-23276.727) (-23329.595) -- 4:52:08
510000 -- (-23335.749) [-23207.785] (-23261.441) (-23284.783) * (-23282.600) [-23240.537] (-23266.233) (-23330.677) -- 4:51:51

Average standard deviation of split frequencies: 0.025211

510500 -- (-23335.416) [-23206.143] (-23275.305) (-23282.592) * (-23289.137) [-23243.094] (-23261.137) (-23320.530) -- 4:51:33
511000 -- (-23297.880) [-23212.523] (-23266.051) (-23281.896) * (-23300.406) [-23240.856] (-23270.685) (-23306.637) -- 4:51:15
511500 -- (-23306.093) [-23199.002] (-23256.693) (-23283.926) * (-23309.029) [-23241.046] (-23276.935) (-23320.507) -- 4:50:58
512000 -- (-23307.841) [-23200.157] (-23266.073) (-23295.303) * (-23297.464) [-23236.669] (-23271.073) (-23348.619) -- 4:50:41
512500 -- (-23307.416) [-23197.074] (-23276.698) (-23284.545) * (-23285.004) [-23258.655] (-23280.782) (-23326.149) -- 4:50:23
513000 -- (-23301.785) [-23227.089] (-23258.000) (-23284.221) * (-23284.108) [-23258.182] (-23282.870) (-23326.545) -- 4:50:06
513500 -- (-23308.052) [-23220.524] (-23244.639) (-23285.980) * (-23278.672) [-23263.638] (-23269.999) (-23333.928) -- 4:49:49
514000 -- (-23310.204) [-23213.884] (-23250.643) (-23288.267) * (-23289.301) [-23248.665] (-23260.917) (-23341.920) -- 4:49:33
514500 -- (-23293.905) [-23193.964] (-23259.988) (-23299.261) * (-23290.534) (-23260.419) [-23263.880] (-23321.557) -- 4:49:15
515000 -- (-23301.369) [-23206.994] (-23265.228) (-23300.542) * (-23295.261) [-23257.922] (-23285.803) (-23311.535) -- 4:48:58

Average standard deviation of split frequencies: 0.024850

515500 -- (-23317.490) [-23218.435] (-23287.286) (-23297.989) * (-23289.830) [-23244.695] (-23283.975) (-23312.684) -- 4:48:40
516000 -- (-23295.566) [-23229.495] (-23269.407) (-23279.124) * (-23301.916) [-23245.200] (-23282.483) (-23316.021) -- 4:48:23
516500 -- (-23289.227) [-23210.905] (-23288.474) (-23295.131) * (-23298.888) [-23253.970] (-23277.372) (-23312.902) -- 4:48:06
517000 -- (-23296.193) [-23214.418] (-23280.235) (-23308.097) * (-23292.865) [-23250.128] (-23278.617) (-23325.237) -- 4:47:48
517500 -- (-23328.992) [-23214.486] (-23267.323) (-23301.952) * (-23292.828) [-23259.451] (-23263.507) (-23308.121) -- 4:47:31
518000 -- (-23351.290) [-23210.949] (-23277.120) (-23312.099) * (-23306.134) [-23253.005] (-23263.303) (-23307.334) -- 4:47:14
518500 -- (-23311.220) [-23216.394] (-23253.750) (-23321.748) * (-23301.903) (-23251.231) [-23236.170] (-23304.217) -- 4:46:56
519000 -- (-23313.033) [-23217.249] (-23253.327) (-23306.742) * (-23308.219) (-23255.149) [-23227.477] (-23308.465) -- 4:46:39
```

519500 -- (-23306.874) [-23221.076] (-23269.955) (-23294.842) * (-23306.071) (-23256.986) [-23240.540] (-23276.369) -- 4:46:22
520000 -- (-23328.439) [-23247.480] (-23272.807) (-23288.860) * (-23282.654) (-23258.729) [-23242.800] (-23294.540) -- 4:46:05

Average standard deviation of split frequencies: 0.024370

520500 -- (-23327.093) [-23231.600] (-23265.361) (-23296.406) * (-23297.135) (-23260.161) [-23236.472] (-23311.429) -- 4:45:47
521000 -- (-23309.294) [-23246.471] (-23268.077) (-23324.007) * (-23284.937) (-23282.110) [-23233.408] (-23318.172) -- 4:45:30
521500 -- (-23306.834) [-23254.730] (-23265.762) (-23328.900) * (-23289.388) (-23272.333) [-23225.586] (-23330.026) -- 4:45:12
522000 -- (-23312.956) (-23261.287) [-23262.066] (-23318.624) * (-23285.299) (-23270.271) [-23221.901] (-23313.831) -- 4:44:54
522500 -- (-23294.469) [-23253.726] (-23258.373) (-23314.418) * (-23293.711) (-23274.111) [-23240.316] (-23297.624) -- 4:44:36
523000 -- (-23286.676) (-23254.058) [-23247.151] (-23287.166) * (-23278.879) (-23281.494) [-23256.668] (-23288.874) -- 4:44:17
523500 -- (-23277.641) (-23279.338) [-23255.114] (-23305.926) * (-23275.129) (-23299.095) [-23255.013] (-23292.537) -- 4:44:00
524000 -- [-23254.195] (-23266.918) (-23255.143) (-23294.583) * (-23283.284) (-23281.596) [-23249.974] (-23279.889) -- 4:43:43
524500 -- [-23256.892] (-23273.272) (-23259.038) (-23318.474) * (-23304.132) (-23284.812) [-23242.014] (-23313.517) -- 4:43:25
525000 -- (-23248.198) [-23252.513] (-23255.564) (-23338.366) * (-23308.619) (-23284.106) [-23239.923] (-23307.854) -- 4:43:07

Average standard deviation of split frequencies: 0.023985

525500 -- (-23253.547) (-23255.236) [-23244.381] (-23361.668) * (-23299.644) (-23275.801) [-23235.130] (-23298.263) -- 4:42:50
526000 -- (-23259.818) [-23252.831] (-23244.093) (-23343.516) * (-23298.602) (-23275.890) [-23237.499] (-23323.031) -- 4:42:34
526500 -- (-23275.854) (-23248.455) [-23239.835] (-23329.991) * (-23297.328) (-23269.505) [-23242.905] (-23315.930) -- 4:42:16
527000 -- (-23290.439) (-23271.343) [-23251.249] (-23334.292) * (-23286.186) [-23268.021] (-23252.981) (-23310.388) -- 4:41:58
527500 -- (-23304.027) (-23264.819) [-23259.183] (-23315.510) * (-23301.974) [-23263.965] (-23250.186) (-23314.251) -- 4:41:40
528000 -- (-23321.454) (-23260.735) [-23244.259] (-23295.611) * (-23296.498) (-23266.300) [-23238.959] (-23318.777) -- 4:41:22
528500 -- (-23341.195) (-23254.735) [-23239.712] (-23299.773) * (-23304.380) (-23272.690) [-23217.011] (-23305.452) -- 4:41:05
529000 -- (-23317.901) (-23257.799) [-23242.975] (-23325.389) * (-23287.116) (-23258.606) [-23219.193] (-23300.308) -- 4:40:47
529500 -- (-23300.890) (-23246.905) [-23257.744] (-23341.075) * (-23284.597) (-23244.384) [-23210.163] (-23306.890) -- 4:40:29
530000 -- (-23301.430) [-23260.790] (-23260.928) (-23330.927) * (-23260.532) (-23237.363) [-23230.749] (-23320.186) -- 4:40:11

Average standard deviation of split frequencies: 0.023782

530500 -- (-23302.964) [-23250.501] (-23288.817) (-23332.333) * (-23288.398) (-23244.692) [-23243.472] (-23327.215) -- 4:39:54
531000 -- (-23306.322) [-23238.133] (-23283.717) (-23332.586) * (-23284.726) (-23254.089) [-23230.700] (-23337.338) -- 4:39:36
531500 -- (-23293.054) [-23249.308] (-23273.248) (-23327.679) * (-23288.106) (-23246.548) [-23224.314] (-23342.611) -- 4:39:18
532000 -- (-23291.281) [-23250.914] (-23247.186) (-23335.594) * (-23311.172) (-23256.827) [-23250.125] (-23344.602) -- 4:39:01
532500 -- (-23298.395) [-23245.793] (-23246.574) (-23318.839) * (-23296.438) (-23248.415) [-23235.370] (-23334.327) -- 4:38:43
533000 -- (-23281.850) (-23249.502) [-23242.704] (-23313.084) * (-23286.207) (-23255.272) [-23235.256] (-23351.323) -- 4:38:25
533500 -- (-23261.695) [-23244.450] (-23237.021) (-23319.160) * (-23296.750) [-23235.972] (-23243.673) (-23360.982) -- 4:38:08
534000 -- (-23269.805) (-23263.625) [-23247.534] (-23327.100) * (-23273.661) (-23246.845) [-23234.470] (-23336.864) -- 4:37:51
534500 -- (-23260.924) (-23264.259) [-23242.174] (-23337.749) * (-23267.770) [-23231.355] (-23252.710) (-23305.384) -- 4:37:33
535000 -- (-23285.726) (-23276.809) [-23254.855] (-23336.079) * (-23275.859) (-23241.163) [-23264.905] (-23345.881) -- 4:37:15

Average standard deviation of split frequencies: 0.023842

535500 -- (-23297.419) (-23268.178) [-23256.260] (-23325.068) * (-23260.562) [-23232.072] (-23262.243) (-23331.606) -- 4:36:57
536000 -- (-23298.316) (-23259.012) [-23263.013] (-23310.771) * (-23271.883) [-23228.105] (-23256.189) (-23303.917) -- 4:36:40
536500 -- (-23296.489) [-23237.769] (-23247.326) (-23311.928) * (-23270.384) [-23235.189] (-23252.200) (-23289.269) -- 4:36:22
537000 -- (-23270.433) [-23259.694] (-23241.597) (-23331.154) * (-23267.164) [-23241.690] (-23239.675) (-23292.027) -- 4:36:04
537500 -- (-23276.107) (-23261.384) [-23248.890] (-23330.452) * (-23283.807) [-23247.770] (-23244.519) (-23283.704) -- 4:35:47
538000 -- (-23288.259) (-23264.910) [-23250.443] (-23316.795) * (-23282.410) [-23251.148] (-23278.329) (-23260.892) -- 4:35:28
538500 -- (-23294.782) [-23254.080] (-23246.400) (-23310.935) * (-23276.086) (-23254.284) (-23274.960) [-23266.991] -- 4:35:11
539000 -- (-23302.830) [-23247.675] (-23257.457) (-23317.369) * (-23282.917) (-23286.428) (-23275.604) [-23258.507] -- 4:34:53
539500 -- (-23276.419) [-23255.737] (-23264.445) (-23321.694) * (-23265.784) (-23293.586) (-23276.986) [-23256.527] -- 4:34:35
540000 -- (-23282.085) (-23254.898) [-23258.347] (-23320.861) * (-23261.943) (-23304.381) (-23270.169) [-23267.580] -- 4:34:18

Average standard deviation of split frequencies: 0.023610

540500 -- (-23275.753) (-23257.912) [-23246.985] (-23321.350) * (-23246.916) (-23303.401) (-23278.911) [-23256.897] -- 4:34:00
541000 -- (-23311.654) (-23275.945) [-23243.837] (-23324.424) * (-23254.869) (-23280.883) (-23297.282) [-23236.855] -- 4:33:43
541500 -- (-23293.020) (-23269.140) [-23249.992] (-23320.453) * (-23276.667) (-23263.014) (-23269.694) [-23215.971] -- 4:33:26
542000 -- (-23293.070) (-23269.687) [-23256.239] (-23305.608) * (-23292.278) (-23259.524) (-23280.524) [-23216.237] -- 4:33:09
542500 -- (-23299.407) (-23259.223) [-23234.448] (-23298.314) * (-23275.205) (-23265.439) (-23287.874) [-23221.422] -- 4:32:50
543000 -- (-23295.331) (-23284.813) [-23231.791] (-23297.185) * (-23296.361) (-23246.041) (-23298.622) [-23219.641] -- 4:32:32
543500 -- (-23308.073) (-23276.758) [-23235.486] (-23303.256) * (-23288.922) (-23276.247) (-23296.766) [-23215.157] -- 4:32:14
544000 -- (-23278.518) (-23282.766) [-23237.635] (-23298.074) * (-23298.646) (-23283.122) (-23309.624) [-23218.959] -- 4:31:56
544500 -- (-23257.707) (-23271.922) [-23224.455] (-23298.228) * (-23317.705) (-23275.330) (-23337.691) [-23242.952] -- 4:31:38
545000 -- (-23245.361) (-23279.944) [-23218.944] (-23301.012) * (-23319.112) (-23260.562) (-23313.839) [-23232.537] -- 4:31:19

Average standard deviation of split frequencies: 0.023852

545500 -- (-23248.248) (-23289.700) [-23233.129] (-23313.507) * (-23318.176) (-23277.576) (-23313.088) [-23220.582] -- 4:31:02
546000 -- (-23238.095) (-23300.574) [-23246.678] (-23317.100) * (-23330.825) (-23288.458) (-23283.435) [-23234.478] -- 4:30:44
546500 -- [-23226.557] (-23304.767) (-23260.369) (-23317.273) * (-23317.090) (-23278.399) (-23292.630) [-23245.225] -- 4:30:26
547000 -- [-23232.457] (-23322.431) (-23250.650) (-23286.850) * (-23318.006) (-23269.908) (-23287.178) [-23255.838] -- 4:30:08
547500 -- [-23235.260] (-23326.053) (-23263.918) (-23299.204) * (-23328.727) (-23281.170) (-23294.546) [-23252.712] -- 4:29:50
548000 -- [-23240.182] (-23303.755) (-23255.920) (-23301.176) * (-23334.982) (-23269.642) (-23295.433) [-23245.322] -- 4:29:33
548500 -- [-23246.946] (-23315.826) (-23277.737) (-23299.601) * (-23317.071) (-23253.934) (-23299.704) [-23260.166] -- 4:29:15
549000 -- (-23267.911) (-23293.049) [-23263.821] (-23314.516) * (-23336.601) (-23256.197) (-23305.140) [-23249.633] -- 4:28:57
549500 -- (-23267.601) (-23305.048) [-23242.853] (-23330.977) * (-23319.760) (-23262.297) (-23316.371) [-23248.858] -- 4:28:39
550000 -- (-23263.180) (-23291.526) [-23243.092] (-23331.329) * (-23321.095) (-23244.989) (-23313.722) [-23237.714] -- 4:28:21

Average standard deviation of split frequencies: 0.024041

550500 -- (-23267.665) (-23287.995) [-23236.201] (-23336.033) * (-23331.873) (-23246.597) (-23301.687) [-23242.396] -- 4:28:03
551000 -- (-23262.538) (-23272.694) [-23238.289] (-23340.395) * (-23318.168) (-23238.609) (-23310.901) [-23243.452] -- 4:27:45
551500 -- [-23268.056] (-23276.420) (-23239.239) (-23324.265) * (-23314.118) [-23240.486] (-23323.851) (-23251.864) -- 4:27:26
552000 -- (-23295.441) (-23249.799) [-23238.282] (-23328.162) * (-23312.581) [-23243.440] (-23324.688) (-23260.608) -- 4:27:08
552500 -- (-23297.130) (-23249.068) [-23233.825] (-23312.450) * (-23323.753) [-23239.394] (-23319.580) (-23233.059) -- 4:26:51
553000 -- (-23279.219) (-23248.202) [-23228.319] (-23312.519) * (-23309.825) [-23233.025] (-23314.921) (-23254.905) -- 4:26:34
553500 -- (-23278.835) [-23245.164] (-23247.305) (-23295.295) * (-23307.761) [-23231.642] (-23317.600) (-23251.696) -- 4:26:16
554000 -- (-23291.162) [-23246.691] (-23229.020) (-23277.361) * (-23303.633) [-23222.044] (-23302.039) (-23255.523) -- 4:25:59
554500 -- (-23304.157) [-23234.464] (-23252.478) (-23298.034) * (-23299.150) [-23224.858] (-23300.068) (-23252.625) -- 4:25:41
555000 -- (-23310.320) [-23254.033] [-23254.858] (-23286.070) * (-23298.128) [-23217.569] (-23285.685) (-23260.870) -- 4:25:23

Average standard deviation of split frequencies: 0.023804

555500 -- (-23321.904) [-23254.419] (-23263.993) (-23280.140) * (-23319.754) [-23210.337] (-23277.822) (-23265.445) -- 4:25:05
556000 -- (-23302.259) [-23241.605] (-23269.778) (-23283.404) * (-23321.778) [-23225.488] (-23278.848) (-23257.275) -- 4:24:48
556500 -- (-23296.556) [-23259.739] (-23242.147) (-23245.276) * (-23322.787) [-23235.176] (-23286.095) (-23261.211) -- 4:24:31
557000 -- (-23299.942) (-23278.260) (-23249.925) [-23247.869] * (-23340.469) [-23251.202] (-23274.518) (-23262.103) -- 4:24:13
557500 -- (-23310.374) (-23284.590) [-23243.029] (-23239.926) * (-23352.544) [-23243.822] (-23265.516) (-23263.829) -- 4:23:56
558000 -- (-23296.194) (-23284.923) (-23242.831) [-23228.136] * (-23332.785) [-23228.616] (-23286.276) (-23271.775) -- 4:23:38
558500 -- (-23289.329) (-23282.961) [-23236.944] (-23236.371) * (-23338.258) [-23225.362] (-23279.460) (-23271.790) -- 4:23:20
559000 -- (-23298.590) (-23269.181) [-23234.818] (-23245.489) * (-23329.963) [-23217.608] (-23270.163) (-23262.350) -- 4:23:02
559500 -- (-23320.928) (-23266.258) [-23242.807] (-23233.961) * (-23322.016) [-23230.005] (-23260.614) (-23267.322) -- 4:22:45
560000 -- (-23336.631) [-23252.154] (-23247.068) (-23268.642) * (-23324.553) [-23230.001] (-23249.852) (-23270.773) -- 4:22:27

Average standard deviation of split frequencies: 0.023366

```
560500 -- (-23317.330) (-23238.116) [-23245.858] (-23289.089) * (-23316.023) [-23216.309] (-23252.455) (-23288.268) -- 4:22:08
561000 -- (-23315.236) (-23257.383) [-23245.904] (-23286.856) * (-23312.958) [-23229.425] (-23259.716) (-23281.788) -- 4:21:51
561500 -- (-23293.738) (-23252.220) [-23241.541] (-23297.770) * (-23324.880) [-23215.760] (-23274.191) (-23296.921) -- 4:21:33
562000 -- (-23298.782) (-23248.119) [-23243.970] (-23327.801) * (-23310.627) [-23220.341] (-23279.304) (-23290.585) -- 4:21:16
562500 -- (-23282.640) [-23238.513] (-23247.134) (-23303.092) * (-23288.796) [-23242.159] (-23292.877) (-23275.046) -- 4:20:58
563000 -- (-23274.542) (-23246.901) [-23258.513] (-23320.240) * (-23313.401) [-23241.278] (-23289.549) (-23265.686) -- 4:20:39
563500 -- (-23284.860) [-23243.110] (-23256.939) (-23316.290) * (-23284.095) [-23229.667] (-23305.859) (-23268.843) -- 4:20:21
564000 -- (-23279.964) [-23242.596] (-23257.749) (-23331.075) * (-23284.200) [-23219.769] (-23312.219) (-23287.639) -- 4:20:04
564500 -- (-23282.082) (-23253.729) [-23240.932] (-23324.834) * (-23285.957) [-23232.113] (-23299.281) (-23298.257) -- 4:19:46
565000 -- (-23264.622) (-23262.922) [-23237.256] (-23308.980) * (-23297.285) [-23238.608] (-23290.965) (-23287.495) -- 4:19:28

Average standard deviation of split frequencies: 0.022681

565500 -- (-23267.675) [-23252.393] (-23226.442) (-23319.866) * (-23291.602) [-23219.232] (-23295.139) (-23286.095) -- 4:19:09
566000 -- (-23261.861) (-23264.467) [-23216.433] (-23327.124) * (-23293.471) [-23233.141] (-23280.592) (-23286.346) -- 4:18:51
566500 -- (-23273.127) (-23258.479) [-23219.125] (-23305.266) * (-23265.611) [-23233.851] (-23277.030) (-23278.921) -- 4:18:31
567000 -- (-23262.079) [-23249.848] (-23240.184) (-23306.589) * (-23269.882) [-23234.946] (-23295.580) (-23294.259) -- 4:18:14
567500 -- [-23258.960] (-23248.271) (-23244.105) (-23309.458) * (-23272.153) [-23232.695] (-23313.204) (-23293.208) -- 4:17:55
568000 -- (-23259.399) [-23246.092] (-23261.670) (-23305.499) * (-23264.761) [-23226.800] (-23307.176) (-23271.639) -- 4:17:36
568500 -- [-23243.857] (-23242.543) (-23277.211) (-23304.624) * (-23279.152) [-23236.189] (-23293.166) (-23292.566) -- 4:17:19
569000 -- [-23236.052] (-23250.143) (-23268.246) (-23309.054) * (-23279.379) [-23231.451] (-23303.520) (-23308.077) -- 4:17:00
569500 -- [-23247.473] (-23269.542) (-23260.495) (-23305.173) * (-23271.832) [-23227.853] (-23306.433) (-23291.504) -- 4:16:42
570000 -- [-23250.134] (-23273.803) (-23265.269) (-23309.152) * (-23265.678) [-23238.002] (-23317.694) (-23275.552) -- 4:16:24

Average standard deviation of split frequencies: 0.022342

570500 -- [-23267.141] (-23282.701) (-23270.563) (-23338.248) * (-23266.269) [-23233.799] (-23343.057) (-23283.777) -- 4:16:05
571000 -- [-23239.739] (-23286.270) (-23260.005) (-23302.283) * (-23256.608) [-23253.254] (-23320.291) (-23274.865) -- 4:15:47
571500 -- [-23242.692] (-23290.496) (-23266.328) (-23303.109) * (-23273.320) [-23255.399] (-23305.303) (-23286.033) -- 4:15:29
572000 -- [-23261.135] (-23296.413) (-23261.552) (-23293.335) * (-23260.886) [-23234.185] (-23328.225) (-23280.857) -- 4:15:11
572500 -- [-23266.325] (-23277.221) (-23254.471) (-23319.251) * (-23246.643) [-23232.988] (-23318.390) (-23291.064) -- 4:14:53
573000 -- [-23263.537] (-23275.132) (-23255.577) (-23313.162) * (-23254.809) [-23237.893] (-23300.734) (-23281.613) -- 4:14:35
573500 -- (-23275.176) (-23288.559) [-23264.978] (-23303.364) * (-23267.144) [-23237.987] (-23307.640) (-23286.402) -- 4:14:18
574000 -- (-23272.608) (-23285.258) [-23270.543] (-23303.019) * (-23267.076) [-23230.115] (-23326.004) (-23299.418) -- 4:14:00
574500 -- (-23280.629) (-23290.594) [-23257.477] (-23287.988) * (-23279.235) [-23244.361] (-23321.947) (-23284.175) -- 4:13:42
575000 -- (-23277.946) (-23284.870) [-23253.317] (-23298.268) * (-23294.842) [-23240.171] (-23347.191) (-23271.017) -- 4:13:24

Average standard deviation of split frequencies: 0.022166

575500 -- (-23280.339) (-23276.816) [-23265.312] (-23284.921) * (-23282.151) [-23246.762] (-23351.833) (-23271.298) -- 4:13:06
576000 -- (-23286.291) (-23296.496) [-23262.089] (-23300.690) * (-23278.804) [-23260.644] (-23331.812) (-23267.124) -- 4:12:49
576500 -- (-23304.461) (-23282.590) [-23267.462] (-23282.653) * (-23271.925) [-23250.540] (-23337.893) (-23268.623) -- 4:12:31
577000 -- (-23304.920) (-23268.291) [-23266.282] (-23278.245) * (-23273.822) [-23271.536] (-23324.965) (-23267.876) -- 4:12:14
577500 -- (-23288.539) (-23260.691) [-23281.771] (-23285.675) * (-23266.220) [-23274.613] (-23327.880) (-23271.550) -- 4:11:57
578000 -- (-23303.052) (-23268.779) (-23269.772) [-23272.326] * (-23290.783) [-23263.455] (-23290.293) (-23268.370) -- 4:11:39
578500 -- (-23279.900) [-23252.168] (-23289.564) (-23257.382) * (-23303.613) (-23263.064) (-23286.083) [-23247.191] -- 4:11:22
579000 -- (-23290.596) (-23246.099) (-23279.661) [-23265.849] * (-23296.522) [-23244.980] (-23290.651) (-23255.878) -- 4:11:05
579500 -- (-23296.352) (-23285.349) [-23260.466] (-23267.882) * (-23294.355) [-23244.422] (-23273.183) (-23250.827) -- 4:10:47
580000 -- (-23286.178) (-23295.330) (-23254.875) [-23255.616] * (-23296.660) (-23267.290) (-23291.338) [-23249.798] -- 4:10:29

Average standard deviation of split frequencies: 0.021979

580500 -- (-23290.213) (-23295.085) [-23252.574] (-23266.188) * (-23298.248) (-23253.642) (-23278.338) [-23241.150] -- 4:10:12
581000 -- (-23312.802) (-23291.967) [-23256.118] (-23281.950) * (-23304.377) (-23244.411) (-23279.274) (-23246.132) -- 4:09:54
581500 -- (-23303.561) (-23295.068) [-23253.640] (-23275.065) * (-23281.153) (-23252.946) [-23261.698] (-23266.875) -- 4:09:37
582000 -- (-23303.607) (-23279.520) [-23255.926] (-23270.508) * (-23293.050) [-23240.240] (-23281.105) (-23261.781) -- 4:09:19
582500 -- (-23306.565) (-23278.064) [-23244.542] (-23265.328) * (-23288.541) [-23208.251] (-23281.670) (-23260.026) -- 4:09:02
583000 -- (-23335.160) (-23279.194) [-23242.545] (-23262.645) * (-23279.902) [-23228.680] (-23303.155) (-23261.435) -- 4:08:44
583500 -- (-23334.200) (-23281.091) [-23258.161] (-23277.302) * (-23300.809) [-23239.638] (-23292.466) (-23251.668) -- 4:08:26
584000 -- (-23316.734) (-23286.385) [-23261.928] (-23281.969) * (-23280.331) [-23243.952] (-23288.440) (-23265.681) -- 4:08:09
584500 -- (-23314.668) (-23293.940) (-23258.470) [-23267.118] * (-23268.366) [-23242.007] (-23290.942) (-23254.452) -- 4:07:51
585000 -- (-23309.110) (-23295.164) [-23264.553] (-23250.382) * (-23258.730) (-23271.990) (-23319.043) [-23233.883] -- 4:07:34

Average standard deviation of split frequencies: 0.021602

585500 -- (-23302.520) (-23280.706) [-23252.751] (-23265.675) * (-23255.841) (-23270.391) (-23319.707) [-23227.931] -- 4:07:17
586000 -- (-23320.279) (-23277.497) [-23259.590] (-23256.838) * (-23268.603) (-23270.665) (-23313.513) [-23223.318] -- 4:06:58
586500 -- (-23311.026) (-23264.287) (-23267.280) [-23251.461] * (-23234.582) (-23276.671) (-23313.431) [-23217.805] -- 4:06:40
587000 -- (-23312.554) [-23247.692] (-23264.029) (-23252.381) * (-23262.848) (-23264.527) (-23329.387) [-23231.207] -- 4:06:22
587500 -- (-23334.232) (-23272.133) [-23242.144] (-23237.776) * (-23258.625) (-23291.883) (-23317.710) [-23218.078] -- 4:06:05
588000 -- (-23329.818) (-23270.812) (-23248.101) [-23230.198] * (-23268.162) (-23279.663) (-23285.856) [-23242.143] -- 4:05:47
588500 -- (-23341.286) (-23250.116) (-23251.777) [-23241.121] * (-23263.074) (-23285.195) (-23293.269) [-23244.457] -- 4:05:29
589000 -- (-23326.129) (-23238.970) (-23255.300) [-23228.449] * (-23260.715) (-23267.549) (-23280.612) [-23266.108] -- 4:05:11
589500 -- (-23312.833) (-23246.093) (-23246.394) [-23226.457] * (-23264.278) [-23273.047] (-23307.380) (-23272.987) -- 4:04:53
590000 -- (-23297.725) (-23261.819) (-23252.270) [-23222.189] * (-23281.601) [-23268.029] (-23304.044) (-23281.025) -- 4:04:35

Average standard deviation of split frequencies: 0.021567

590500 -- (-23283.902) (-23264.851) (-23255.622) [-23225.898] * (-23277.001) [-23259.558] (-23316.255) (-23275.633) -- 4:04:17
591000 -- (-23293.076) (-23273.965) (-23258.759) [-23241.038] * (-23260.387) (-23270.917) (-23307.266) [-23257.158] -- 4:04:00
591500 -- (-23319.127) (-23270.076) (-23262.846) [-23232.400] * (-23271.253) (-23273.654) (-23300.778) (-23272.323) -- 4:03:42
592000 -- (-23304.212) (-23264.921) (-23266.084) [-23246.469] * (-23242.272) (-23277.746) (-23323.253) [-23266.230] -- 4:03:24
592500 -- (-23295.604) (-23278.928) (-23251.247) [-23231.027] * [-23247.170] (-23283.683) (-23334.593) (-23275.704) -- 4:03:06
593000 -- (-23300.705) (-23252.330) (-23245.401) (-23242.802) * [-23249.463] (-23268.680) (-23344.152) (-23265.591) -- 4:02:48
593500 -- (-23323.279) (-23259.145) [-23250.840] (-23234.084) * (-23274.072) (-23257.782) (-23333.262) [-23248.291] -- 4:02:31
594000 -- (-23356.065) (-23262.869) (-23261.121) [-23233.320] * (-23294.353) (-23285.398) (-23329.545) [-23250.613] -- 4:02:13
594500 -- (-23352.091) (-23275.834) (-23262.161) [-23238.078] * (-23291.296) (-23289.299) (-23314.141) [-23254.691] -- 4:01:55
595000 -- (-23367.020) (-23289.083) (-23275.199) [-23237.768] * (-23287.079) (-23278.523) (-23324.227) [-23234.900] -- 4:01:37

Average standard deviation of split frequencies: 0.021557

595500 -- (-23333.525) (-23273.480) [-23278.854] (-23266.849) * (-23276.589) (-23252.064) (-23329.488) [-23236.008] -- 4:01:19
596000 -- (-23336.843) (-23268.077) (-23277.428) [-23256.274] * (-23278.591) (-23262.724) (-23309.912) [-23236.223] -- 4:01:01
596500 -- (-23294.466) (-23262.352) [-23287.261] [-23252.892] * (-23282.561) (-23267.681) (-23313.267) [-23233.452] -- 4:00:44
597000 -- (-23299.753) (-23265.658) (-23282.442) (-23263.622) * (-23274.981) (-23269.679) (-23314.192) [-23244.382] -- 4:00:25
597500 -- (-23290.080) [-23265.633] (-23307.067) (-23263.814) * (-23284.346) [-23256.433] (-23313.016) (-23265.074) -- 4:00:07
598000 -- (-23298.362) (-23260.237) (-23312.534) [-23272.765] * (-23291.666) (-23257.835) (-23321.775) [-23248.335] -- 3:59:48
598500 -- (-23291.568) [-23270.376] (-23297.921) (-23284.791) * (-23274.975) (-23271.561) (-23314.541) [-23229.575] -- 3:59:30
599000 -- (-23286.355) (-23258.154) (-23282.724) [-23282.178] * (-23288.432) (-23287.009) (-23324.822) [-23224.509] -- 3:59:12
599500 -- (-23293.593) (-23271.611) (-23292.277) [-23288.623] * (-23278.834) (-23297.237) (-23302.224) [-23231.333] -- 3:58:54
600000 -- (-23292.980) (-23275.857) (-23304.117) [-23259.061] * (-23291.682) (-23285.972) (-23296.861) [-23228.938] -- 3:58:36

Average standard deviation of split frequencies: 0.021182

600500 -- (-23297.207) (-23279.632) (-23293.953) [-23242.695] * (-23272.975) (-23283.152) (-23328.387) [-23249.528] -- 3:58:18
601000 -- (-23278.308) (-23279.850) (-23300.372) [-23254.390] * (-23264.161) (-23279.489) (-23324.746) [-23242.778] -- 3:57:59
601500 -- [-23277.859] (-23273.967) (-23292.465) (-23271.403) * (-23260.869) (-23271.196) (-23306.161) [-23238.387] -- 3:57:41
```

602000 -- [-23257.179] (-23276.685) (-23310.221) (-23276.200) * (-23258.307) (-23281.653) (-23299.834) [-23235.034] -- 3:57:22
602500 -- [-23240.209] (-23283.373) (-23315.125) (-23291.592) * (-23248.579) (-23295.652) (-23285.651) [-23235.056] -- 3:57:04
603000 -- [-23248.245] (-23278.776) (-23317.359) (-23298.795) * [-23237.173] (-23298.827) (-23280.489) (-23228.607) -- 3:56:46
603500 -- [-23241.895] (-23282.750) (-23328.998) (-23317.392) * (-23247.122) (-23331.852) (-23264.569) [-23237.090] -- 3:56:27
604000 -- [-23239.789] (-23261.729) (-23313.509) (-23286.962) * [-23243.930] (-23332.250) (-23239.079) (-23264.679) -- 3:56:09
604500 -- [-23246.276] [-23258.828] (-23303.584) (-23290.715) * [-23236.202] (-23304.989) (-23261.462) (-23260.433) -- 3:55:50
605000 -- [-23248.300] (-23260.982) (-23306.131) (-23277.137) * [-23241.082] (-23300.203) (-23246.594) (-23270.575) -- 3:55:31

Average standard deviation of split frequencies: 0.021016

605500 -- [-23234.319] (-23274.249) (-23308.312) (-23295.051) * (-23239.733) (-23290.516) [-23230.772] (-23287.086) -- 3:55:14
606000 -- [-23239.747] (-23266.618) (-23288.717) (-23275.489) * (-23247.434) (-23290.784) [-23227.888] (-23294.775) -- 3:54:55
606500 -- [-23235.056] (-23288.510) (-23294.164) (-23270.950) * [-23223.846] (-23308.402) (-23241.991) (-23308.101) -- 3:54:37
607000 -- [-23235.475] (-23280.137) (-23273.632) (-23263.906) * [-23234.170] (-23311.356) (-23260.608) (-23312.969) -- 3:54:19
607500 -- [-23232.510] (-23254.604) (-23290.219) (-23271.316) * [-23220.222] (-23301.686) (-23257.807) (-23294.638) -- 3:54:02
608000 -- [-23221.763] (-23250.232) (-23288.901) (-23268.747) * [-23219.797] (-23308.644) (-23249.203) (-23294.330) -- 3:53:44
608500 -- [-23211.216] (-23242.099) (-23278.221) (-23289.031) * [-23216.349] (-23304.115) (-23272.233) (-23283.643) -- 3:53:26
609000 -- [-23207.138] (-23249.092) (-23270.546) (-23300.443) * [-23218.035] (-23284.957) (-23274.409) (-23264.201) -- 3:53:08
609500 -- (-23230.014) [-23256.988] (-23275.053) (-23312.397) * (-23211.724) (-23284.119) (-23290.724) [-23269.142] -- 3:52:50
610000 -- [-23238.184] (-23265.597) (-23266.860) (-23305.681) * [-23216.494] (-23289.435) (-23292.241) (-23286.614) -- 3:52:31

Average standard deviation of split frequencies: 0.020955

610500 -- [-23253.162] (-23265.958) (-23284.710) (-23323.832) * [-23226.494] (-23316.330) (-23269.976) (-23254.059) -- 3:52:13
611000 -- [-23237.642] (-23264.168) (-23288.282) (-23303.942) * [-23220.413] (-23289.378) (-23293.777) (-23251.068) -- 3:51:56
611500 -- [-23241.652] (-23254.982) (-23296.865) (-23290.766) * [-23212.978] (-23316.246) (-23287.298) (-23246.224) -- 3:51:37
612000 -- (-23249.006) [-23252.259] (-23290.638) (-23277.146) * [-23225.386] (-23312.955) (-23286.486) (-23263.404) -- 3:51:19
612500 -- (-23258.430) [-23251.493] (-23270.643) (-23272.432) * [-23221.336] (-23317.036) (-23271.990) (-23268.437) -- 3:51:02
613000 -- (-23257.528) [-23233.435] (-23278.193) (-23281.131) * [-23233.433] (-23311.412) (-23287.185) (-23271.386) -- 3:50:44
613500 -- (-23269.830) [-23225.640] (-23302.517) (-23291.166) * [-23215.579] (-23301.761) (-23281.942) (-23272.536) -- 3:50:26
614000 -- (-23294.254) [-23225.144] (-23313.598) (-23300.257) * [-23224.744] (-23291.670) (-23276.232) (-23250.010) -- 3:50:08
614500 -- (-23270.496) [-23241.642] (-23319.593) (-23312.636) * (-23227.192) (-23279.980) (-23279.799) [-23246.665] -- 3:49:50
615000 -- (-23273.526) [-23236.717] (-23288.969) (-23320.627) * [-23235.290] (-23294.649) (-23263.648) (-23267.069) -- 3:49:32

Average standard deviation of split frequencies: 0.020711

615500 -- (-23280.046) [-23259.227] (-23285.007) (-23313.145) * [-23241.438] (-23308.671) (-23265.208) (-23258.141) -- 3:49:15
616000 -- (-23271.976) [-23248.190] (-23296.088) (-23291.151) * [-23246.225] (-23302.283) (-23295.791) [-23248.995] -- 3:48:57
616500 -- (-23270.407) [-23250.230] (-23301.133) (-23298.433) * [-23243.925] (-23311.427) (-23289.104) [-23244.600] -- 3:48:39
617000 -- (-23260.823) [-23251.737] (-23308.419) (-23301.183) * [-23226.903] (-23298.027) (-23293.357) (-23241.815) -- 3:48:21
617500 -- [-23268.951] (-23259.483) (-23309.814) (-23303.802) * (-23230.397) (-23289.311) (-23280.430) [-23269.990] -- 3:48:03
618000 -- (-23264.770) [-23279.154] (-23325.197) (-23299.655) * [-23227.871] (-23295.029) (-23306.482) [-23262.889] -- 3:47:46
618500 -- (-23252.074) [-23282.961] (-23308.622) (-23308.061) * [-23229.766] (-23321.752) (-23298.282) (-23262.552) -- 3:47:28
619000 -- [-23244.479] (-23262.838) (-23312.793) (-23290.521) * [-23229.115] (-23324.488) (-23295.796) (-23262.746) -- 3:47:10
619500 -- (-23255.710) [-23249.102] (-23319.632) (-23269.011) * [-23226.485] (-23316.551) (-23291.938) (-23269.197) -- 3:46:52
620000 -- (-23250.348) [-23242.794] (-23329.498) (-23284.110) * [-23241.688] (-23304.736) (-23313.605) (-23262.926) -- 3:46:34

Average standard deviation of split frequencies: 0.020570

620500 -- (-23249.990) [-23246.681] (-23317.644) (-23292.939) * [-23239.138] (-23303.687) (-23305.778) (-23260.246) -- 3:46:16
621000 -- [-23252.695] (-23245.655) (-23331.315) (-23303.840) * [-23223.864] (-23292.283) (-23323.756) (-23248.097) -- 3:45:59
621500 -- (-23270.962) [-23236.525] (-23322.560) (-23308.903) * [-23239.497] (-23299.137) (-23316.587) (-23260.610) -- 3:45:40
622000 -- (-23258.537) [-23222.952] (-23335.573) (-23284.128) * (-23225.542) (-23288.099) (-23296.993) [-23254.326] -- 3:45:22
622500 -- (-23272.789) [-23212.999] (-23335.578) (-23277.306) * [-23220.457] (-23288.128) (-23284.882) (-23240.445) -- 3:45:05
623000 -- [-23272.793] (-23232.074) (-23317.444) (-23276.833) * [-23240.769] (-23267.879) (-23286.258) (-23247.967) -- 3:44:46
623500 -- (-23268.425) [-23240.083] (-23316.625) (-23308.938) * [-23236.012] (-23266.806) (-23285.780) (-23241.611) -- 3:44:28
624000 -- (-23249.543) [-23249.978] (-23290.023) (-23280.082) * [-23231.281] (-23259.119) (-23291.026) (-23251.321) -- 3:44:11
624500 -- (-23263.239) [-23243.314] (-23292.961) (-23249.713) * [-23233.196] (-23259.046) (-23296.219) (-23247.963) -- 3:43:53
625000 -- (-23272.186) [-23241.863] (-23296.042) (-23261.443) * [-23218.541] (-23243.936) (-23298.406) (-23263.788) -- 3:43:36

Average standard deviation of split frequencies: 0.020462

625500 -- (-23264.851) [-23230.721] (-23298.761) (-23272.101) * [-23214.300] (-23267.754) (-23311.655) (-23259.917) -- 3:43:17
626000 -- (-23265.192) [-23237.585] (-23304.204) (-23285.522) * [-23226.173] (-23284.487) (-23308.719) (-23262.628) -- 3:42:59
626500 -- (-23276.530) [-23239.652] (-23277.749) (-23292.895) * [-23234.651] (-23286.211) (-23327.229) (-23266.244) -- 3:42:41
627000 -- (-23303.552) [-23240.108] (-23295.429) (-23300.631) * [-23231.672] (-23271.293) (-23343.889) (-23273.894) -- 3:42:23
627500 -- (-23305.146) [-23240.922] (-23307.051) (-23292.036) * [-23244.720] (-23272.983) (-23343.470) (-23277.841) -- 3:42:05
628000 -- (-23299.694) [-23254.401] (-23307.634) (-23288.797) * [-23223.807] (-23274.943) (-23333.999) (-23260.143) -- 3:41:47
628500 -- (-23313.891) [-23235.550] (-23296.545) (-23286.050) * [-23238.595] (-23267.453) (-23342.154) (-23260.273) -- 3:41:29
629000 -- (-23317.961) [-23225.567] (-23287.057) (-23299.960) * (-23263.283) (-23253.443) (-23332.835) [-23264.987] -- 3:41:11
629500 -- (-23321.866) [-23227.280] (-23278.039) (-23275.866) * (-23275.987) [-23259.240] (-23341.826) (-23246.872) -- 3:40:53
630000 -- (-23309.530) [-23229.419] (-23286.833) (-23306.120) * (-23273.946) (-23251.524) (-23318.097) [-23255.648] -- 3:40:35

Average standard deviation of split frequencies: 0.020264

630500 -- (-23305.832) [-23222.319] (-23271.251) (-23295.822) * (-23272.771) (-23262.184) (-23325.172) [-23251.870] -- 3:40:17
631000 -- (-23294.295) [-23213.387] (-23251.049) (-23287.292) * (-23299.192) [-23252.431] (-23319.309) (-23250.289) -- 3:39:59
631500 -- (-23285.791) [-23217.381] (-23245.712) (-23282.357) * (-23299.802) [-23261.416] (-23313.525) (-23249.571) -- 3:39:41
632000 -- (-23277.128) [-23203.551] (-23242.278) (-23307.588) * (-23308.507) [-23244.450] (-23316.609) (-23248.381) -- 3:39:23
632500 -- (-23278.571) [-23208.246] (-23256.217) (-23315.128) * (-23302.243) (-23254.182) (-23316.360) [-23244.514] -- 3:39:05
633000 -- (-23265.138) [-23229.182] (-23262.135) (-23300.755) * (-23303.694) (-23255.591) (-23320.736) [-23209.979] -- 3:38:47
633500 -- (-23270.774) [-23235.261] (-23256.237) (-23302.738) * (-23295.076) (-23279.372) (-23343.358) [-23198.920] -- 3:38:29
634000 -- (-23301.909) [-23220.652] (-23253.461) (-23271.481) * (-23280.065) (-23267.840) (-23349.385) [-23197.854] -- 3:38:11
634500 -- (-23304.065) [-23228.277] (-23266.083) (-23284.219) * (-23295.143) (-23258.383) (-23360.748) [-23208.475] -- 3:37:53
635000 -- (-23298.056) [-23226.937] (-23251.196) (-23289.448) * (-23259.952) (-23245.268) (-23346.063) [-23225.547] -- 3:37:35

Average standard deviation of split frequencies: 0.020161

635500 -- (-23294.475) (-23236.360) [-23258.237] (-23292.858) * (-23263.862) (-23253.471) (-23339.267) [-23250.159] -- 3:37:17
636000 -- (-23296.107) [-23231.790] (-23286.996) (-23279.197) * (-23273.444) (-23258.893) (-23320.866) [-23252.559] -- 3:36:59
636500 -- (-23290.402) [-23236.236] (-23281.996) (-23293.162) * (-23267.204) (-23253.245) (-23316.736) [-23239.742] -- 3:36:42
637000 -- (-23282.784) [-23225.874] (-23265.960) (-23288.353) * (-23270.773) (-23263.333) (-23301.344) [-23261.621] -- 3:36:24
637500 -- (-23278.431) [-23240.030] (-23258.756) (-23282.844) * (-23267.202) (-23280.125) (-23291.552) [-23242.313] -- 3:36:05
638000 -- (-23275.835) (-23251.897) [-23261.940] (-23294.428) * [-23255.856] (-23304.919) (-23278.051) (-23259.599) -- 3:35:48
638500 -- (-23280.088) [-23243.662] (-23268.002) (-23302.142) * [-23246.545] (-23286.743) (-23296.431) (-23258.935) -- 3:35:30
639000 -- (-23276.515) [-23247.890] (-23268.095) (-23309.818) * [-23224.368] (-23289.575) (-23288.838) (-23250.984) -- 3:35:12
639500 -- (-23313.865) [-23223.987] (-23273.847) (-23312.726) * (-23239.127) (-23281.573) (-23304.966) [-23235.935] -- 3:34:54
640000 -- (-23289.262) [-23224.079] (-23286.576) (-23308.247) * [-23235.977] (-23287.058) (-23330.578) (-23244.945) -- 3:34:36

Average standard deviation of split frequencies: 0.019791

640500 -- (-23286.957) [-23228.634] (-23282.396) (-23311.874) * (-23254.636) (-23276.367) (-23342.576) [-23223.181] -- 3:34:18
641000 -- (-23276.017) [-23222.067] (-23278.348) (-23306.110) * (-23243.998) (-23283.111) (-23358.738) [-23219.114] -- 3:34:00
641500 -- (-23273.707) [-23221.190] (-23272.851) (-23299.479) * (-23230.985) (-23290.721) (-23324.775) [-23222.026] -- 3:33:42
642000 -- (-23272.235) [-23233.475] (-23275.761) (-23277.651) * (-23233.062) (-23315.737) (-23333.166) [-23218.151] -- 3:33:24
642500 -- (-23266.651) [-23245.255] (-23294.350) (-23274.064) * [-23226.227] (-23305.510) (-23352.935) (-23233.001) -- 3:33:06
643000 -- (-23276.025) [-23241.214] (-23275.393) (-23295.485) * (-23242.676) (-23297.270) (-23359.567) [-23222.593] -- 3:32:48
643500 -- (-23293.930) (-23244.583) [-23262.895] (-23313.126) * (-23245.471) (-23277.889) (-23329.877) [-23216.448] -- 3:32:30

```
644000 -- (-23304.971) [-23238.391] (-23277.099) (-23301.640) * (-23266.016) (-23280.090) (-23326.773) [-23220.294] -- 3:32:12
644500 -- (-23302.695) [-23239.187] (-23252.766) (-23302.260) * (-23245.073) (-23271.394) (-23320.572) [-23239.177] -- 3:31:54
645000 -- (-23297.408) [-23240.319] (-23253.369) (-23296.691) * (-23253.013) (-23269.970) (-23315.941) [-23226.601] -- 3:31:35

Average standard deviation of split frequencies: 0.019461

645500 -- (-23322.041) (-23245.617) [-23235.539] (-23293.707) * (-23265.870) (-23260.913) (-23319.544) [-23221.749] -- 3:31:17
646000 -- (-23297.865) (-23254.398) [-23227.648] (-23282.518) * (-23272.318) (-23270.502) (-23316.124) [-23237.501] -- 3:30:59
646500 -- (-23291.267) (-23258.437) [-23231.945] (-23273.765) * (-23262.521) (-23245.776) (-23331.818) [-23252.988] -- 3:30:41
647000 -- (-23296.311) (-23274.461) (-23249.848) [-23253.872] * (-23280.327) (-23263.700) (-23344.013) [-23232.549] -- 3:30:23
647500 -- (-23269.080) (-23273.764) [-23257.568] (-23248.881) * (-23286.574) (-23253.002) (-23340.813) [-23246.987] -- 3:30:05
648000 -- (-23272.454) (-23274.936) [-23255.171] (-23246.269) * (-23289.462) (-23267.204) (-23344.335) [-23228.426] -- 3:29:47
648500 -- [-23252.101] (-23272.297) (-23267.402) (-23262.228) * (-23275.741) (-23267.263) (-23364.253) [-23236.856] -- 3:29:29
649000 -- (-23273.152) (-23257.070) [-23259.880] (-23256.507) * (-23296.249) (-23260.854) (-23348.658) [-23232.337] -- 3:29:12
649500 -- (-23272.232) [-23238.069] (-23247.649) (-23245.689) * (-23274.840) (-23274.097) (-23337.231) [-23230.589] -- 3:28:54
650000 -- (-23261.431) (-23259.384) [-23244.116] (-23256.173) * (-23267.036) (-23273.042) (-23316.755) [-23255.827] -- 3:28:37

Average standard deviation of split frequencies: 0.019319

650500 -- (-23282.444) (-23275.256) [-23245.842] (-23266.614) * (-23269.119) (-23278.885) (-23322.564) [-23258.534] -- 3:28:19
651000 -- (-23264.145) (-23278.234) [-23248.165] (-23296.691) * (-23274.845) (-23283.081) (-23318.620) [-23262.089] -- 3:28:01
651500 -- (-23261.849) (-23270.524) [-23243.198] (-23299.524) * (-23266.161) (-23291.861) (-23344.479) [-23253.740] -- 3:27:44
652000 -- (-23259.256) (-23267.941) [-23244.145] (-23308.994) * (-23259.336) (-23278.939) (-23339.648) [-23254.064] -- 3:27:26
652500 -- (-23248.568) (-23276.030) [-23217.421] (-23313.825) * (-23250.093) (-23273.019) (-23335.115) [-23276.277] -- 3:27:09
653000 -- (-23246.747) (-23262.303) [-23224.251] (-23283.252) * (-23249.594) (-23270.615) (-23350.531) [-23261.182] -- 3:26:52
653500 -- (-23255.854) (-23264.790) [-23239.084] (-23300.931) * [-23257.089] (-23267.510) (-23335.152) (-23267.918) -- 3:26:34
654000 -- (-23257.382) (-23258.835) [-23243.278] (-23308.907) * (-23253.878) (-23258.888) (-23317.232) [-23248.557] -- 3:26:17
654500 -- (-23255.365) (-23249.112) [-23239.303] (-23313.708) * (-23242.417) (-23270.696) (-23316.097) [-23251.583] -- 3:26:00
655000 -- (-23250.733) [-23234.862] (-23241.195) (-23299.757) * (-23242.653) (-23284.663) (-23302.981) [-23244.311] -- 3:25:43

Average standard deviation of split frequencies: 0.019603

655500 -- (-23246.438) (-23248.855) [-23237.673] (-23297.382) * (-23254.504) (-23282.107) (-23290.615) [-23233.802] -- 3:25:26
656000 -- (-23254.723) (-23245.855) [-23236.475] (-23304.294) * (-23257.375) (-23278.157) (-23295.349) [-23247.050] -- 3:25:09
656500 -- (-23268.076) (-23238.321) [-23247.975] (-23283.039) * (-23258.859) (-23264.150) (-23313.509) [-23238.722] -- 3:24:51
657000 -- [-23253.882] (-23271.904) (-23236.040) (-23293.612) * [-23242.990] (-23273.558) (-23286.436) (-23249.913) -- 3:24:33
657500 -- (-23261.645) (-23277.966) [-23226.572] (-23289.781) * (-23244.304) (-23257.475) (-23300.334) [-23253.290] -- 3:24:16
658000 -- (-23258.962) (-23278.372) [-23239.315] (-23301.479) * [-23247.552] (-23273.900) (-23308.022) (-23258.867) -- 3:23:57
658500 -- (-23269.939) (-23272.691) [-23238.761] (-23301.118) * [-23253.395] (-23273.110) (-23314.221) (-23261.853) -- 3:23:39
659000 -- (-23262.267) (-23270.048) [-23239.524] (-23317.485) * [-23241.483] (-23279.751) (-23284.392) (-23242.200) -- 3:23:22
659500 -- (-23284.708) (-23268.460) [-23246.704] (-23317.697) * [-23237.269] (-23273.546) (-23293.717) (-23233.406) -- 3:23:04
660000 -- (-23300.084) (-23256.914) [-23258.379] (-23319.397) * [-23238.076] (-23289.171) (-23301.703) (-23233.330) -- 3:22:46

Average standard deviation of split frequencies: 0.019665

660500 -- (-23282.604) [-23256.719] (-23255.640) (-23313.490) * (-23230.934) (-23277.733) (-23326.177) [-23237.779] -- 3:22:28
661000 -- [-23277.150] (-23272.247) (-23265.473) (-23311.117) * (-23224.457) (-23292.577) (-23317.127) [-23242.984] -- 3:22:10
661500 -- [-23258.076] (-23265.706) (-23243.376) (-23305.453) * [-23223.128] (-23280.772) (-23325.124) (-23263.478) -- 3:21:52
662000 -- [-23255.076] (-23274.598) (-23229.134) (-23317.195) * [-23217.503] (-23265.661) (-23314.819) (-23283.349) -- 3:21:34
662500 -- (-23252.593) (-23310.934) [-23237.188] (-23315.129) * [-23216.292] (-23306.194) (-23312.053) (-23284.433) -- 3:21:16
663000 -- (-23253.738) (-23305.986) [-23247.796] (-23303.110) * [-23233.011] (-23304.414) (-23296.925) (-23284.098) -- 3:20:57
663500 -- (-23262.292) (-23317.608) [-23253.888] (-23305.587) * [-23233.075] (-23303.879) (-23280.964) (-23279.204) -- 3:20:39
664000 -- (-23256.217) (-23319.925) [-23265.904] (-23286.868) * [-23224.982] (-23304.230) (-23263.983) (-23291.424) -- 3:20:21
664500 -- (-23255.973) (-23319.017) [-23259.960] (-23310.029) * [-23245.467] (-23302.918) (-23268.031) (-23275.194) -- 3:20:03
665000 -- (-23246.931) (-23301.469) [-23256.516] (-23306.368) * [-23253.732] (-23294.348) (-23277.861) (-23313.623) -- 3:19:45

Average standard deviation of split frequencies: 0.019632

665500 -- [-23246.770] (-23299.580) (-23264.608) (-23285.436) * (-23248.188) (-23292.181) [-23280.962] (-23314.888) -- 3:19:27
666000 -- [-23245.758] (-23291.202) (-23270.556) (-23312.426) * [-23247.522] (-23305.864) (-23274.300) (-23320.984) -- 3:19:09
666500 -- [-23227.566] (-23272.834) (-23277.452) (-23309.017) * [-23264.160] (-23296.058) (-23273.847) (-23297.972) -- 3:18:52
667000 -- [-23228.189] (-23275.972) (-23274.969) (-23300.839) * (-23268.319) (-23297.113) [-23272.133] (-23295.961) -- 3:18:34
667500 -- [-23230.186] (-23278.456) (-23250.846) (-23316.516) * (-23277.148) (-23284.041) [-23263.939] (-23281.194) -- 3:18:16
668000 -- [-23226.605] (-23282.025) (-23249.650) (-23332.469) * (-23277.093) (-23293.301) [-23254.180] (-23275.583) -- 3:17:59
668500 -- [-23229.566] (-23286.548) (-23280.907) (-23329.482) * (-23289.115) (-23309.141) [-23268.242] (-23278.042) -- 3:17:41
669000 -- [-23225.816] (-23293.382) (-23276.228) (-23329.545) * (-23263.072) (-23303.871) [-23263.247] (-23279.210) -- 3:17:24
669500 -- [-23229.345] (-23304.520) (-23269.499) (-23312.887) * (-23240.899) (-23301.786) [-23248.530] (-23268.161) -- 3:17:06
670000 -- [-23226.774] (-23283.835) (-23259.559) (-23308.425) * [-23243.794] (-23301.235) (-23251.424) (-23267.889) -- 3:16:49

Average standard deviation of split frequencies: 0.019411

670500 -- [-23227.999] (-23278.524) (-23265.247) (-23306.725) * [-23240.398] (-23285.733) (-23282.816) (-23271.645) -- 3:16:31
671000 -- [-23222.717] (-23267.336) (-23268.448) (-23314.119) * [-23249.649] (-23300.096) (-23268.834) (-23283.350) -- 3:16:13
671500 -- [-23221.481] (-23281.641) (-23277.942) (-23308.612) * [-23244.847] (-23308.057) (-23279.976) (-23278.896) -- 3:15:55
672000 -- (-23230.365) (-23294.252) [-23269.512] (-23276.004) * [-23242.824] (-23321.538) (-23283.819) (-23278.259) -- 3:15:36
672500 -- [-23228.096] (-23324.015) (-23268.296) (-23274.109) * (-23236.560) (-23296.812) [-23265.276] (-23280.887) -- 3:15:18
673000 -- [-23239.004] (-23330.926) (-23278.906) (-23280.933) * (-23251.941) (-23306.876) [-23245.027] (-23282.520) -- 3:15:00
673500 -- [-23233.610] (-23296.794) (-23289.571) (-23287.799) * [-23254.761] (-23328.092) (-23249.973) (-23296.425) -- 3:14:42
674000 -- [-23230.533] (-23302.638) (-23277.260) (-23307.396) * [-23263.223] (-23348.364) (-23247.830) (-23306.308) -- 3:14:24
674500 -- [-23209.321] (-23305.320) (-23284.600) (-23297.232) * (-23260.808) (-23335.914) [-23257.634] (-23287.528) -- 3:14:06
675000 -- [-23223.682] (-23299.586) (-23281.108) (-23281.774) * [-23239.873] (-23334.807) (-23256.886) (-23286.086) -- 3:13:49

Average standard deviation of split frequencies: 0.018933

675500 -- [-23236.253] (-23303.571) (-23269.491) (-23300.402) * [-23226.873] (-23320.132) (-23256.898) (-23282.474) -- 3:13:30
676000 -- [-23228.756] (-23290.266) (-23254.207) (-23289.322) * [-23224.362] (-23339.657) (-23266.837) (-23304.246) -- 3:13:13
676500 -- [-23229.608] (-23293.556) (-23258.792) (-23277.441) * [-23220.094] (-23323.883) (-23266.152) (-23299.215) -- 3:12:55
677000 -- (-23241.843) (-23272.621) (-23262.662) (-23273.054) * [-23212.283] (-23305.609) (-23305.893) (-23316.002) -- 3:12:37
677500 -- (-23240.003) (-23282.038) [-23260.350] (-23250.905) * [-23211.123] (-23307.691) (-23303.323) (-23334.981) -- 3:12:19
678000 -- [-23238.611] (-23284.133) (-23276.898) (-23260.708) * [-23216.970] (-23315.591) (-23309.724) (-23332.402) -- 3:12:01
678500 -- [-23244.608] (-23288.754) (-23306.241) (-23269.683) * [-23226.318] (-23316.035) (-23303.543) (-23319.930) -- 3:11:42
679000 -- [-23224.630] (-23279.349) (-23294.436) (-23257.097) * [-23239.498] (-23314.832) (-23302.051) (-23330.840) -- 3:11:24
679500 -- [-23234.829] (-23269.637) (-23285.565) (-23239.232) * [-23231.231] (-23298.484) (-23286.559) (-23333.086) -- 3:11:06
680000 -- (-23238.838) (-23280.833) (-23307.911) [-23226.772] * [-23246.338] (-23299.388) (-23276.553) (-23313.925) -- 3:10:48

Average standard deviation of split frequencies: 0.018598

680500 -- [-23244.942] (-23290.138) (-23296.936) (-23248.548) * [-23248.071] (-23282.080) (-23280.911) (-23339.444) -- 3:10:30
681000 -- (-23245.376) (-23292.320) (-23297.536) [-23227.899] * [-23236.417] (-23281.773) (-23276.303) (-23333.644) -- 3:10:12
681500 -- (-23227.680) (-23281.481) (-23286.377) [-23225.709] * [-23231.164] (-23279.475) (-23286.545) (-23335.025) -- 3:09:54
682000 -- (-23248.954) (-23282.014) (-23282.144) [-23206.507] * [-23248.785] (-23291.105) (-23279.870) (-23330.760) -- 3:09:36
682500 -- (-23270.411) (-23285.990) (-23300.033) [-23213.100] * [-23244.581] (-23278.381) (-23273.765) (-23314.380) -- 3:09:18
683000 -- (-23268.694) (-23284.305) (-23297.469) [-23200.940] * (-23256.637) [-23281.839] (-23280.073) (-23319.750) -- 3:09:00
683500 -- (-23256.658) (-23283.280) (-23303.961) [-23214.697] * (-23271.546) [-23278.140] (-23276.111) (-23348.515) -- 3:08:42
684000 -- (-23269.129) (-23270.319) (-23315.146) [-23228.532] * [-23258.606] (-23282.662) (-23286.024) (-23331.724) -- 3:08:24
684500 -- (-23272.033) (-23281.175) (-23317.479) [-23227.609] * [-23241.760] (-23267.634) (-23305.442) (-23313.628) -- 3:08:06
685000 -- (-23266.406) (-23278.934) (-23335.792) [-23225.055] * [-23248.905] (-23259.688) (-23291.638) (-23298.304) -- 3:07:47
```

```
Average standard deviation of split frequencies: 0.018299

685500 -- (-23286.489) (-23255.429) (-23317.072) [-23204.840] * (-23258.898) [-23248.669] (-23293.860) (-23306.635) -- 3:07:29
686000 -- (-23282.333) (-23266.889) (-23330.948) [-23221.342] * [-23242.450] (-23240.097) (-23296.413) (-23302.180) -- 3:07:11
686500 -- (-23277.324) (-23285.753) (-23353.329) [-23230.965] * [-23226.751] (-23263.243) (-23267.073) (-23299.761) -- 3:06:53
687000 -- (-23270.519) (-23296.174) (-23352.132) [-23231.403] * [-23237.035] (-23259.236) (-23272.592) (-23282.618) -- 3:06:35
687500 -- (-23263.019) (-23273.728) (-23333.226) [-23237.965] * [-23251.139] (-23259.540) (-23290.792) (-23274.465) -- 3:06:17
688000 -- (-23265.589) (-23272.597) (-23323.582) [-23242.834] * [-23234.629] (-23256.047) (-23274.673) (-23299.426) -- 3:05:58
688500 -- (-23275.419) [-23244.354] (-23316.085) (-23268.375) * [-23238.153] (-23271.807) (-23261.515) (-23302.285) -- 3:05:40
689000 -- (-23287.140) [-23256.108] (-23305.113) (-23264.463) * [-23222.916] (-23255.261) (-23267.666) (-23322.438) -- 3:05:22
689500 -- (-23302.416) (-23262.976) (-23293.878) [-23257.607] * [-23223.288] (-23264.812) (-23268.873) (-23330.250) -- 3:05:04
690000 -- (-23296.350) (-23270.654) (-23311.836) [-23256.107] * [-23227.440] (-23257.037) (-23277.255) (-23314.779) -- 3:04:45

Average standard deviation of split frequencies: 0.018200

690500 -- (-23292.002) (-23278.345) (-23316.680) [-23260.366] * [-23229.967] (-23263.616) (-23271.755) (-23324.011) -- 3:04:27
691000 -- (-23297.018) (-23285.980) (-23326.575) [-23239.824] * [-23242.101] (-23263.985) (-23278.508) (-23291.875) -- 3:04:09
691500 -- (-23303.984) (-23274.531) (-23311.433) [-23224.128] * [-23244.210] (-23248.323) (-23281.545) (-23283.434) -- 3:03:51
692000 -- (-23298.825) (-23279.064) (-23302.476) [-23248.145] * (-23236.309) [-23242.228] (-23291.089) (-23296.201) -- 3:03:33
692500 -- (-23310.242) (-23272.908) (-23301.618) [-23253.360] * [-23243.521] (-23253.881) (-23306.173) (-23301.550) -- 3:03:15
693000 -- (-23295.334) (-23260.109) (-23305.712) [-23246.654] * [-23252.926] (-23266.485) (-23309.436) (-23312.873) -- 3:02:57
693500 -- (-23276.823) (-23271.379) (-23311.145) [-23248.339] * (-23259.810) [-23280.149] (-23284.923) (-23314.726) -- 3:02:39
694000 -- (-23270.915) (-23262.065) (-23283.767) [-23231.802] * (-23269.841) [-23251.508] (-23283.345) (-23319.986) -- 3:02:21
694500 -- (-23317.449) (-23253.427) (-23288.330) [-23239.938] * (-23266.717) [-23219.523] (-23265.306) (-23323.022) -- 3:02:03
695000 -- (-23314.240) (-23222.758) (-23285.296) [-23234.552] * (-23273.470) [-23222.493] (-23259.208) (-23321.817) -- 3:01:45

Average standard deviation of split frequencies: 0.017985

695500 -- (-23298.934) [-23217.696] (-23286.451) (-23241.286) * (-23253.899) [-23223.653] (-23270.631) (-23310.197) -- 3:01:27
696000 -- (-23312.095) [-23228.275] (-23290.585) (-23257.066) * (-23267.920) [-23215.033] (-23259.900) (-23321.833) -- 3:01:09
696500 -- (-23306.584) [-23225.252] (-23278.466) (-23263.891) * (-23263.285) [-23215.693] (-23249.379) (-23321.270) -- 3:00:52
697000 -- (-23302.373) [-23226.865] (-23283.458) (-23269.957) * (-23278.781) [-23219.644] (-23247.701) (-23302.314) -- 3:00:34
697500 -- (-23294.297) [-23218.558] (-23255.733) (-23275.467) * (-23269.180) [-23206.081] (-23242.526) (-23298.056) -- 3:00:16
698000 -- (-23300.222) [-23227.903] (-23247.908) (-23273.430) * (-23265.375) [-23218.733] (-23265.965) (-23277.668) -- 2:59:58
698500 -- (-23303.051) [-23217.134] (-23248.774) (-23261.924) * (-23278.129) [-23234.160] (-23243.964) (-23279.978) -- 2:59:41
699000 -- (-23336.945) [-23209.286] (-23252.026) (-23259.446) * (-23302.432) [-23243.264] (-23240.164) (-23287.210) -- 2:59:23
699500 -- (-23330.842) [-23228.972] (-23263.062) (-23259.228) * (-23308.290) [-23250.564] (-23244.435) (-23292.813) -- 2:59:05
700000 -- (-23321.076) [-23225.006] (-23276.809) (-23258.634) * (-23312.282) [-23253.042] (-23254.922) (-23283.192) -- 2:58:47

Average standard deviation of split frequencies: 0.017815

700500 -- (-23323.538) [-23215.584] (-23282.647) (-23267.951) * (-23306.460) [-23257.086] (-23249.360) (-23293.926) -- 2:58:30
701000 -- (-23329.233) [-23222.462] (-23280.962) (-23289.398) * (-23285.725) [-23257.530] (-23269.632) (-23286.763) -- 2:58:12
701500 -- (-23336.716) [-23238.382] (-23258.140) (-23277.810) * (-23293.852) [-23264.034] (-23277.779) (-23298.640) -- 2:57:54
702000 -- (-23315.834) [-23240.944] (-23256.039) (-23293.988) * (-23275.578) [-23243.777] (-23291.853) (-23287.771) -- 2:57:37
702500 -- (-23305.839) (-23260.778) [-23240.487] (-23295.682) * (-23263.518) [-23248.139] (-23288.001) (-23291.146) -- 2:57:19
703000 -- (-23302.235) (-23254.535) [-23243.306] (-23317.109) * (-23257.912) [-23246.235] (-23309.717) (-23291.211) -- 2:57:01
703500 -- (-23308.897) (-23247.743) [-23242.725] (-23326.308) * (-23255.422) [-23234.726] (-23307.002) (-23284.292) -- 2:56:43
704000 -- (-23313.226) [-23247.500] (-23253.506) (-23309.298) * (-23245.660) [-23238.485] (-23296.424) (-23294.334) -- 2:56:25
704500 -- (-23332.105) (-23251.949) [-23242.040] (-23316.335) * (-23241.673) [-23250.858] (-23288.887) (-23286.424) -- 2:56:07
705000 -- (-23339.600) [-23258.350] (-23263.490) (-23290.195) * [-23249.763] (-23258.699) (-23303.668) (-23291.002) -- 2:55:49

Average standard deviation of split frequencies: 0.017880

705500 -- (-23315.458) [-23255.720] (-23292.255) (-23306.059) * (-23258.490) [-23237.305] (-23305.714) (-23284.224) -- 2:55:31
706000 -- (-23291.688) [-23263.234] (-23273.291) (-23307.002) * (-23257.934) [-23227.761] (-23309.612) (-23285.820) -- 2:55:13
706500 -- (-23302.611) [-23251.927] (-23265.840) (-23311.073) * (-23251.743) [-23220.938] (-23304.874) (-23298.608) -- 2:54:56
707000 -- (-23307.898) [-23279.237] (-23280.858) (-23332.470) * (-23255.999) [-23231.526] (-23292.190) (-23293.323) -- 2:54:38
707500 -- (-23308.893) [-23246.658] (-23289.218) (-23340.736) * (-23262.826) [-23236.795] (-23297.712) (-23290.348) -- 2:54:20
708000 -- (-23287.289) [-23261.987] (-23298.889) (-23314.459) * (-23277.041) [-23242.404] (-23296.488) (-23292.600) -- 2:54:03
708500 -- (-23286.691) [-23260.837] (-23291.726) (-23314.695) * (-23291.240) [-23235.107] (-23291.425) (-23279.438) -- 2:53:45
709000 -- (-23280.647) [-23265.608] (-23278.739) (-23332.580) * (-23306.807) [-23232.849] (-23292.456) (-23277.508) -- 2:53:27
709500 -- (-23285.960) [-23272.557] (-23258.634) (-23326.212) * (-23300.699) [-23229.671] (-23298.473) (-23276.389) -- 2:53:09
710000 -- (-23282.819) (-23281.569) [-23245.464] (-23314.337) * (-23309.057) [-23235.720] (-23291.723) (-23282.053) -- 2:52:51

Average standard deviation of split frequencies: 0.017853

710500 -- (-23289.732) (-23269.492) [-23244.807] (-23283.465) * (-23301.021) [-23229.321] (-23277.256) (-23294.968) -- 2:52:33
711000 -- (-23288.312) (-23270.332) [-23239.675] (-23301.529) * (-23305.624) [-23221.713] (-23274.953) (-23275.837) -- 2:52:16
711500 -- (-23308.157) (-23269.899) [-23232.567] (-23310.406) * (-23306.162) [-23219.203] (-23278.545) [-23258.788] -- 2:51:58
712000 -- (-23308.805) (-23269.323) [-23260.185] (-23290.665) * (-23295.437) [-23220.183] (-23277.712) (-23253.952) -- 2:51:40
712500 -- (-23276.272) (-23280.708) [-23259.291] (-23326.323) * (-23320.980) [-23222.747] (-23274.269) (-23237.706) -- 2:51:22
713000 -- (-23257.387) (-23266.281) [-23257.359] (-23336.408) * (-23321.437) [-23231.314] (-23291.946) (-23238.564) -- 2:51:04
713500 -- [-23259.216] (-23265.169) (-23262.887) (-23340.519) * (-23307.876) [-23228.948] (-23291.816) (-23233.421) -- 2:50:46
714000 -- (-23249.219) (-23255.317) [-23249.552] (-23335.622) * (-23299.417) [-23211.340] (-23303.328) (-23235.178) -- 2:50:29
714500 -- (-23270.627) [-23242.473] (-23255.090) (-23339.550) * (-23289.780) [-23227.125] (-23296.388) (-23253.670) -- 2:50:11
715000 -- (-23270.635) [-23238.282] (-23263.826) (-23314.954) * (-23268.109) [-23239.262] (-23300.953) (-23269.550) -- 2:49:53

Average standard deviation of split frequencies: 0.017919

715500 -- (-23254.979) [-23249.102] (-23265.686) (-23293.832) * (-23270.530) [-23245.822] (-23295.391) (-23270.486) -- 2:49:35
716000 -- [-23258.337] (-23239.124) (-23274.898) (-23303.543) * (-23292.411) [-23239.150] (-23297.758) (-23259.896) -- 2:49:17
716500 -- (-23253.053) [-23250.286] (-23275.653) (-23312.095) * (-23264.298) [-23231.130] (-23297.287) (-23263.132) -- 2:48:59
717000 -- (-23237.608) [-23237.214] (-23269.855) (-23308.385) * (-23263.933) [-23226.870] (-23314.088) (-23248.494) -- 2:48:41
717500 -- (-23250.785) [-23238.709] (-23295.523) (-23316.003) * (-23270.478) (-23239.052) (-23324.481) [-23239.547] -- 2:48:23
718000 -- (-23263.383) [-23246.454] (-23296.626) (-23314.238) * (-23281.402) [-23237.693] (-23324.662) (-23240.128) -- 2:48:05
718500 -- (-23273.898) [-23237.378] (-23283.498) (-23324.295) * (-23285.934) [-23223.089] (-23323.067) [-23231.159] -- 2:47:47
719000 -- (-23277.670) [-23232.305] (-23299.206) (-23337.641) * (-23277.936) [-23218.608] (-23317.921) [-23228.810] -- 2:47:29
719500 -- (-23291.037) [-23217.693] (-23292.373) (-23320.732) * (-23268.265) [-23227.966] (-23342.616) (-23245.657) -- 2:47:10
720000 -- (-23281.307) [-23220.327] (-23289.246) (-23319.740) * (-23259.567) [-23221.139] (-23324.993) (-23254.605) -- 2:46:52

Average standard deviation of split frequencies: 0.017834

720500 -- (-23260.246) [-23232.237] (-23277.773) (-23321.878) * (-23257.690) [-23226.026] (-23315.540) (-23239.615) -- 2:46:34
721000 -- [-23239.021] (-23258.125) (-23290.474) (-23333.708) * (-23261.711) (-23232.438) (-23326.388) [-23231.721] -- 2:46:16
721500 -- (-23240.361] (-23271.758) (-23276.031) (-23318.867) * (-23280.115) [-23225.625] (-23325.325) (-23234.827) -- 2:45:58
722000 -- [-23241.225] (-23272.210) (-23263.469) (-23309.636) * (-23281.580) [-23233.591] (-23346.728) (-23227.250) -- 2:45:40
722500 -- [-23244.220] (-23279.467) (-23273.843) (-23326.363) * (-23292.322) [-23222.291] (-23341.833) (-23250.145) -- 2:45:22
723000 -- [-23263.467] (-23278.586) (-23297.538) (-23341.937) * (-23294.275) [-23250.406] (-23335.612) (-23261.872) -- 2:45:04
723500 -- [-23268.981] (-23263.216) (-23316.833) (-23330.313) * (-23285.083) [-23248.924] (-23323.212) (-23283.341) -- 2:44:47
724000 -- (-23260.140) [-23241.455] (-23329.538) (-23329.722) * (-23275.954) [-23251.787] (-23327.398) (-23298.114) -- 2:44:29
724500 -- (-23279.142) [-23212.251] (-23304.236) (-23307.222) * (-23266.984) [-23242.054] (-23302.157) (-23289.191) -- 2:44:11
725000 -- (-23290.281) [-23220.049] (-23287.575) (-23322.827) * [-23258.833] (-23229.323) (-23297.711) (-23290.217) -- 2:43:53

Average standard deviation of split frequencies: 0.017742

725500 -- (-23286.552) [-23245.689] (-23284.211) (-23315.246) * (-23270.121) [-23231.223] (-23303.577) (-23273.043) -- 2:43:35
726000 -- (-23255.041) [-23229.446] (-23275.746) (-23322.712) * (-23266.294) [-23232.666] (-23296.038) (-23284.588) -- 2:43:17
```

```
726500 -- (-23262.842) [-23225.496] (-23276.652) (-23309.462) * (-23281.459) [-23242.863] (-23294.976) (-23292.637) -- 2:42:59
727000 -- (-23271.664) [-23232.738] (-23265.630) (-23326.827) * (-23290.760) [-23240.242] (-23272.025) (-23278.957) -- 2:42:41
727500 -- (-23273.550) [-23230.938] (-23258.658) (-23337.340) * (-23281.843) [-23259.490] (-23265.606) (-23272.432) -- 2:42:23
728000 -- (-23297.163) [-23228.356] (-23264.703) (-23341.275) * (-23269.315) (-23260.894) (-23266.219) [-23253.936] -- 2:42:05
728500 -- (-23300.657) [-23216.232] (-23280.157) (-23324.335) * (-23291.624) [-23253.298] (-23271.323) (-23265.678) -- 2:41:47
729000 -- (-23285.156) [-23232.639] (-23260.035) (-23329.572) * (-23308.000) [-23247.050] (-23302.601) (-23265.015) -- 2:41:29
729500 -- (-23265.541) [-23247.053] (-23274.724) (-23310.580) * (-23275.864) [-23250.453] (-23308.171) (-23253.111) -- 2:41:11
730000 -- (-23258.898) [-23263.286] (-23266.651) (-23326.029) * (-23265.330) [-23255.773] (-23303.278) (-23266.510) -- 2:40:53

Average standard deviation of split frequencies: 0.017906

730500 -- [-23247.521] (-23259.137) (-23277.563) (-23326.737) * (-23265.372) [-23261.707] (-23302.649) (-23273.437) -- 2:40:35
731000 -- (-23250.305) (-23254.349) [-23261.269] (-23321.736) * (-23267.014) [-23262.565] (-23306.556) (-23280.990) -- 2:40:17
731500 -- [-23254.757] (-23253.062) (-23269.060) (-23337.356) * [-23272.145] (-23275.212) (-23283.237) (-23289.609) -- 2:39:58
732000 -- (-23268.198) (-23253.592) [-23264.753] (-23333.283) * [-23245.511] (-23280.124) (-23293.156) (-23297.100) -- 2:39:41
732500 -- (-23282.044) [-23242.127] (-23267.105) (-23313.084) * [-23230.054] (-23277.767) (-23299.829) (-23307.511) -- 2:39:22
733000 -- (-23266.399) [-23237.970] (-23248.860) (-23300.419) * [-23232.812] (-23287.763) (-23290.697) (-23309.008) -- 2:39:04
733500 -- (-23271.081) (-23264.726) [-23251.722] (-23303.031) * [-23233.301] (-23301.292) (-23274.093) (-23289.548) -- 2:38:46
734000 -- (-23266.161) (-23246.614) [-23249.510] (-23293.824) * [-23239.604] (-23316.614) (-23271.904) (-23291.737) -- 2:38:28
734500 -- (-23254.869) (-23252.076) [-23251.160] (-23304.016) * [-23243.836] (-23308.760) (-23249.623) (-23290.109) -- 2:38:10
735000 -- (-23258.702) [-23251.979] (-23264.464) (-23288.927) * [-23237.603] (-23320.548) (-23254.137) (-23296.381) -- 2:37:52

Average standard deviation of split frequencies: 0.017743

735500 -- (-23260.967) [-23236.149] (-23273.762) (-23294.309) * [-23244.713] (-23304.123) (-23249.531) (-23295.244) -- 2:37:34
736000 -- (-23254.405) [-23247.990] (-23271.229) (-23296.669) * [-23227.718] (-23298.369) (-23243.376) (-23280.024) -- 2:37:16
736500 -- (-23272.023) [-23252.771] (-23265.706) (-23296.451) * [-23223.993] (-23301.135) (-23250.182) (-23291.772) -- 2:36:58
737000 -- (-23296.531) (-23250.191) [-23250.485] (-23300.124) * [-23217.480] (-23287.269) (-23265.494) (-23283.665) -- 2:36:40
737500 -- (-23309.209) (-23248.252) [-23238.334] (-23281.783) * [-23228.176] (-23273.613) (-23277.129) (-23287.656) -- 2:36:22
738000 -- (-23309.533) (-23254.266) [-23237.995] (-23300.205) * [-23224.659] (-23301.476) (-23276.075) (-23288.387) -- 2:36:03
738500 -- (-23296.939) (-23255.688) [-23229.776] (-23312.916) * [-23231.957] (-23311.142) (-23286.152) (-23294.937) -- 2:35:45
739000 -- (-23278.204) [-23249.021] (-23241.010) (-23342.187) * [-23243.367] (-23269.095) (-23286.647) (-23286.345) -- 2:35:27
739500 -- (-23292.240) (-23245.279) [-23254.661] (-23348.297) * (-23261.321) [-23273.109] (-23268.248) (-23268.501) -- 2:35:09
740000 -- (-23275.995) [-23240.123] (-23256.564) (-23356.832) * (-23266.479) (-23300.728) [-23254.556] (-23269.165) -- 2:34:51

Average standard deviation of split frequencies: 0.017859

740500 -- (-23296.548) [-23239.658] (-23229.618) (-23321.990) * (-23260.598) (-23274.209) [-23239.993] (-23249.172) -- 2:34:32
741000 -- (-23300.256) (-23247.168) (-23218.980) (-23317.651) * (-23261.809) (-23288.356) [-23212.026] (-23256.061) -- 2:34:14
741500 -- (-23265.031) (-23256.646) [-23236.504] (-23308.315) * (-23253.111) (-23303.480) [-23210.969] (-23269.128) -- 2:33:56
742000 -- (-23274.059) (-23271.083) [-23251.880] (-23309.930) * (-23273.262) (-23306.556) [-23214.070] (-23275.235) -- 2:33:38
742500 -- (-23265.139) (-23275.870) [-23235.044] (-23294.548) * (-23263.453) (-23297.511) [-23220.835] (-23271.811) -- 2:33:20
743000 -- (-23273.899) (-23285.428) [-23232.010] (-23304.084) * (-23264.427) (-23292.361) [-23227.983] (-23285.120) -- 2:33:02
743500 -- (-23276.004) (-23287.217) [-23235.047] (-23287.528) * (-23251.070) (-23272.934) [-23244.832] (-23279.690) -- 2:32:45
744000 -- (-23271.576) (-23275.424) [-23231.295] (-23297.323) * (-23276.483) (-23273.054) [-23264.335] (-23292.647) -- 2:32:27
744500 -- (-23263.332) (-23272.297) [-23234.783] (-23311.532) * [-23256.127] (-23268.500) (-23277.539) (-23281.419) -- 2:32:09
745000 -- (-23293.954) (-23263.315) [-23246.817] (-23323.894) * [-23252.324] (-23265.804) (-23283.228) (-23306.419) -- 2:31:51

Average standard deviation of split frequencies: 0.017792

745500 -- (-23285.058) (-23275.624) [-23250.256] (-23327.610) * [-23238.407] (-23260.544) (-23286.680) (-23317.968) -- 2:31:33
746000 -- (-23290.102) (-23284.244) [-23248.248] (-23325.732) * [-23235.469] (-23250.172) (-23294.069) (-23315.098) -- 2:31:15
746500 -- (-23287.239) (-23267.980) [-23273.877] (-23319.368) * [-23236.172] (-23249.099) (-23306.606) (-23317.727) -- 2:30:57
747000 -- (-23299.710) (-23271.273) [-23279.889] (-23331.504) * (-23236.946) [-23240.856] (-23295.527) (-23305.691) -- 2:30:38
747500 -- (-23289.943) (-23265.473) [-23279.856] (-23327.001) * (-23263.903) [-23233.517] (-23300.152) (-23318.866) -- 2:30:20
748000 -- (-23314.589) (-23260.539) [-23271.165] (-23331.476) * (-23270.075) [-23251.614] (-23264.429) (-23327.280) -- 2:30:02
748500 -- (-23304.271) (-23275.579) [-23261.245] (-23329.709) * [-23252.983] (-23256.551) (-23267.859) (-23332.300) -- 2:29:45
749000 -- (-23287.419) (-23289.905) [-23251.706] (-23314.642) * [-23245.833] (-23260.732) (-23265.853) (-23312.495) -- 2:29:26
749500 -- (-23300.993) (-23313.185) [-23253.335] (-23301.586) * [-23237.446] (-23244.702) (-23275.299) (-23309.113) -- 2:29:09
750000 -- (-23289.610) (-23320.894) [-23240.756] (-23304.338) * [-23222.011] (-23245.019) (-23289.407) (-23302.434) -- 2:28:51

Average standard deviation of split frequencies: 0.017741

750500 -- (-23269.910) (-23327.709) [-23256.967] (-23302.635) * [-23235.970] (-23247.303) (-23279.092) (-23310.936) -- 2:28:32
751000 -- (-23267.053) (-23308.699) [-23267.895] (-23299.768) * [-23247.952] (-23241.371) (-23283.265) (-23319.465) -- 2:28:14
751500 -- [-23253.626] (-23298.392) (-23271.197) (-23270.474) * (-23270.855) [-23256.363] (-23290.094) (-23310.601) -- 2:27:56
752000 -- (-23260.352) (-23298.691) [-23261.809] (-23274.599) * (-23274.078) [-23252.931] (-23284.322) (-23320.801) -- 2:27:38
752500 -- (-23273.882) (-23297.534) [-23259.166] (-23276.000) * (-23270.150) [-23257.893] (-23290.847) (-23313.425) -- 2:27:20
753000 -- (-23263.910) (-23284.181) [-23229.526] (-23263.074) * (-23273.791) [-23265.541] (-23270.375) (-23312.638) -- 2:27:02
753500 -- (-23257.104) (-23287.277) [-23225.515] (-23255.768) * (-23272.891) [-23244.482] (-23270.535) (-23310.147) -- 2:26:44
754000 -- (-23265.932) (-23290.780) [-23237.801] (-23256.928) * [-23237.588] (-23276.291) (-23287.194) (-23315.360) -- 2:26:26
754500 -- (-23275.300) (-23294.921) [-23237.798] (-23266.786) * [-23229.051] (-23268.932) (-23281.136) (-23304.535) -- 2:26:08
755000 -- (-23278.882) (-23292.169) [-23236.528] (-23287.439) * [-23237.831] (-23264.860) (-23287.991) (-23315.200) -- 2:25:50

Average standard deviation of split frequencies: 0.017762

755500 -- (-23294.529) (-23292.940) [-23232.880] (-23271.184) * [-23227.611] (-23257.555) (-23295.366) (-23325.560) -- 2:25:32
756000 -- (-23279.428) (-23273.008) [-23237.243] (-23262.421) * [-23227.269] (-23276.326) (-23318.524) (-23306.758) -- 2:25:14
756500 -- (-23267.765) (-23271.614) [-23247.518] (-23275.109) * [-23233.694] (-23285.068) (-23307.811) (-23303.635) -- 2:24:56
757000 -- (-23288.157) (-23271.943) [-23250.785] [-23262.178] * [-23223.324] (-23265.856) (-23315.734) (-23302.867) -- 2:24:38
757500 -- (-23283.503) (-23249.500) [-23248.643] (-23276.381) * [-23214.640] (-23276.413) (-23303.159) (-23301.628) -- 2:24:20
758000 -- (-23291.609) (-23265.734) [-23242.814] (-23280.114) * [-23211.789] (-23278.743) (-23294.092) (-23301.881) -- 2:24:02
758500 -- (-23299.226) (-23269.591) [-23228.812] (-23273.242) * [-23215.805] (-23283.752) (-23285.175) (-23293.973) -- 2:23:43
759000 -- (-23306.685) (-23263.439) [-23220.959] (-23285.792) * [-23211.988] (-23290.725) (-23257.236) (-23295.719) -- 2:23:25
759500 -- (-23293.332) (-23278.740) [-23232.746] (-23272.163) * [-23220.659] (-23283.979) (-23256.217) (-23290.463) -- 2:23:07
760000 -- (-23280.951) (-23302.593) [-23231.262] (-23256.143) * [-23220.691] (-23295.859) (-23238.407) (-23301.022) -- 2:22:49

Average standard deviation of split frequencies: 0.017443

760500 -- (-23286.505) (-23324.594) [-23213.708] (-23252.046) * [-23221.484] (-23306.218) (-23234.011) (-23305.001) -- 2:22:31
761000 -- (-23300.784) (-23333.586) [-23224.605] (-23233.133) * [-23222.804] (-23290.613) (-23249.831) (-23307.046) -- 2:22:13
761500 -- (-23282.203) (-23348.742) [-23213.643] (-23257.983) * [-23225.361] (-23295.758) (-23255.320) (-23284.945) -- 2:21:55
762000 -- (-23282.113) (-23333.659) [-23228.762] (-23259.809) * [-23240.915] (-23319.952) (-23255.733) (-23275.739) -- 2:21:38
762500 -- (-23276.593) (-23317.467) [-23219.366] (-23263.894) * (-23246.159) (-23310.955) [-23240.294] (-23275.527) -- 2:21:20
763000 -- (-23247.474) (-23300.152) [-23218.136] (-23293.981) * (-23255.416) (-23297.598) [-23229.252] (-23276.315) -- 2:21:02
763500 -- (-23262.326) (-23308.173) [-23229.057] (-23301.497) * (-23245.834) (-23308.738) [-23238.091] (-23288.894) -- 2:20:44
764000 -- (-23259.669) (-23306.747) [-23201.605] (-23330.602) * [-23259.442] (-23298.217) (-23237.028) (-23277.288) -- 2:20:26
764500 -- (-23272.107) (-23306.857) [-23208.257] (-23315.834) * (-23254.677) (-23331.479) [-23239.858] (-23276.476) -- 2:20:08
765000 -- (-23254.188) (-23317.316) [-23225.108] (-23315.541) * (-23244.841) (-23317.869) [-23242.137] (-23294.510) -- 2:19:50

Average standard deviation of split frequencies: 0.017115

765500 -- (-23251.714) (-23304.868) [-23217.267] (-23337.907) * [-23217.854] (-23315.450) (-23246.662) (-23298.051) -- 2:19:32
766000 -- (-23266.267) (-23282.996) [-23216.822] (-23333.311) * [-23234.556] (-23292.826) [-23248.585] (-23314.873) -- 2:19:14
766500 -- (-23276.482) (-23276.357) [-23229.948] (-23336.174) * [-23247.917] (-23291.808) (-23245.743) (-23324.514) -- 2:18:57
767000 -- (-23270.649) (-23277.218) [-23259.557] (-23309.863) * [-23254.945] (-23285.270) [-23232.361] (-23308.545) -- 2:18:39
767500 -- [-23274.465] (-23293.390) (-23280.859) (-23317.222) * (-23262.751) (-23281.591) [-23231.509] (-23298.658) -- 2:18:21
768000 -- (-23286.113) (-23290.292) [-23269.878] (-23334.777) * (-23241.420) (-23322.228) [-23239.250] (-23311.686) -- 2:18:03
```

```
768500 -- [-23279.589] (-23274.559) (-23271.341) (-23344.287) * (-23245.272) (-23329.801) [-23234.676] (-23299.537) -- 2:17:45
769000 -- [-23259.417] (-23268.493) (-23283.389) (-23351.863) * (-23244.488) (-23336.210) [-23231.384] (-23291.276) -- 2:17:27
769500 -- (-23276.765) (-23263.699) [-23251.232] (-23324.786) * (-23246.732) (-23341.303) [-23236.158] (-23304.384) -- 2:17:09
770000 -- (-23274.712) (-23264.398) [-23257.251] (-23321.144) * (-23261.306) (-23317.805) [-23239.031] (-23296.910) -- 2:16:51

Average standard deviation of split frequencies: 0.017230

770500 -- [-23271.172] (-23267.341) (-23265.202) (-23329.208) * (-23251.298) (-23319.388) [-23229.127] (-23307.341) -- 2:16:33
771000 -- [-23264.542] (-23284.725) (-23252.461) (-23322.314) * (-23251.362) (-23317.843) [-23231.378] (-23328.277) -- 2:16:15
771500 -- [-23254.293] (-23285.504) (-23270.687) (-23320.565) * (-23267.465) (-23320.294) [-23227.228] (-23354.439) -- 2:15:57
772000 -- [-23252.627] (-23284.356) (-23294.911) (-23342.053) * (-23268.624) (-23289.074) [-23238.513] (-23332.775) -- 2:15:39
772500 -- [-23245.944] (-23290.755) (-23285.926) (-23338.849) * (-23263.676) (-23282.745) [-23244.781] (-23341.531) -- 2:15:21
773000 -- [-23251.254] (-23280.016) (-23282.326) (-23333.181) * (-23250.635) (-23284.111) [-23243.629] (-23331.534) -- 2:15:03
773500 -- [-23230.836] (-23280.654) (-23273.149) (-23331.225) * (-23247.635) (-23290.175) [-23242.136] (-23313.257) -- 2:14:45
774000 -- [-23222.416] (-23271.643) (-23263.302) (-23336.538) * (-23248.736) (-23281.198) [-23239.720] (-23303.850) -- 2:14:27
774500 -- [-23215.805] (-23264.512) (-23265.039) (-23320.594) * (-23273.392) (-23280.261) [-23221.852] (-23307.274) -- 2:14:09
775000 -- [-23213.045] (-23268.307) (-23270.568) (-23299.434) * (-23289.066) (-23288.126) [-23249.092] (-23312.936) -- 2:13:51

Average standard deviation of split frequencies: 0.017304

775500 -- [-23218.952] (-23277.323) (-23266.180) (-23321.599) * (-23288.947) (-23274.742) [-23244.985] (-23317.174) -- 2:13:33
776000 -- [-23219.837] (-23265.399) (-23284.382) (-23309.553) * (-23280.414) [-23264.026] (-23242.910) (-23314.370) -- 2:13:15
776500 -- [-23218.930] [-23246.414] (-23274.199) (-23320.832) * (-23278.031) (-23254.365) [-23241.415] (-23314.302) -- 2:12:58
777000 -- (-23224.480) [-23245.809] (-23274.137) (-23312.973) * (-23295.914) (-23238.708) [-23242.290] (-23312.207) -- 2:12:40
777500 -- (-23234.349) [-23238.936] (-23284.857) (-23301.253) * (-23281.962) [-23238.646] (-23253.442) (-23330.534) -- 2:12:22
778000 -- (-23248.348) [-23242.042] (-23256.735) (-23322.558) * (-23279.723) (-23260.282) [-23247.974] (-23333.074) -- 2:12:04
778500 -- (-23262.603) (-23234.506) [-23232.223] (-23336.906) * (-23277.598) (-23260.793) [-23242.460] (-23330.168) -- 2:11:47
779000 -- (-23253.896) (-23250.120) [-23240.013] (-23305.868) * (-23300.978) [-23263.666] (-23243.854) (-23335.057) -- 2:11:29
779500 -- (-23275.538) [-23249.398] (-23240.606) (-23307.476) * (-23305.440) (-23255.930) [-23238.523] (-23332.807) -- 2:11:11
780000 -- (-23277.320) [-23244.387] (-23260.288) (-23335.904) * (-23312.032) (-23263.476) [-23238.336] (-23348.694) -- 2:10:53

Average standard deviation of split frequencies: 0.017214

780500 -- [-23274.896] (-23250.606) (-23274.265) (-23328.872) * (-23302.058) (-23283.445) [-23234.817] (-23333.062) -- 2:10:35
781000 -- (-23261.059) [-23227.154] (-23280.078) (-23343.853) * (-23291.832) (-23279.948) [-23237.127] (-23349.416) -- 2:10:17
781500 -- (-23277.835) [-23241.503] (-23291.052) (-23348.538) * (-23276.059) (-23292.562) [-23243.094] (-23337.393) -- 2:09:59
782000 -- (-23280.032) [-23226.173] (-23282.159) (-23361.702) * (-23274.718) (-23281.754) [-23257.582] (-23333.975) -- 2:09:41
782500 -- (-23268.484) [-23232.385] (-23302.214) (-23349.452) * (-23266.545) (-23274.538) [-23245.445] (-23331.113) -- 2:09:23
783000 -- [-23254.747] (-23227.459) (-23302.932) (-23340.726) * [-23252.131] (-23273.631) (-23244.298) (-23318.583) -- 2:09:05
783500 -- (-23253.861) [-23219.609] (-23301.659) (-23319.134) * (-23249.663) (-23276.185) [-23244.405] (-23304.527) -- 2:08:47
784000 -- (-23252.370) [-23214.688] (-23316.277) (-23335.415) * (-23262.698) (-23268.764) [-23232.230] (-23293.417) -- 2:08:29
784500 -- (-23254.012) [-23226.203] (-23321.172) (-23343.784) * (-23279.010) (-23276.255) [-23232.277] (-23298.427) -- 2:08:12
785000 -- (-23254.230) [-23228.055] (-23318.507) (-23316.859) * (-23287.143) (-23282.245) [-23238.963] (-23326.586) -- 2:07:54

Average standard deviation of split frequencies: 0.016918

785500 -- (-23248.152) [-23224.806] (-23320.636) (-23283.821) * (-23322.123) (-23256.964) [-23240.868] (-23336.488) -- 2:07:36
786000 -- [-23239.103] (-23236.644) (-23307.771) (-23306.384) * (-23332.632) [-23249.388] (-23242.879) (-23338.256) -- 2:07:18
786500 -- (-23246.966) [-23240.089] (-23290.937) (-23329.050) * (-23318.196) (-23262.415) [-23248.080] (-23348.744) -- 2:07:00
787000 -- [-23251.193] (-23245.982) (-23288.316) (-23320.642) * (-23305.132) (-23265.241) [-23251.569] (-23335.915) -- 2:06:42
787500 -- (-23258.897) [-23240.569] (-23294.615) (-23321.760) * (-23324.355) (-23262.494) [-23243.925] (-23327.147) -- 2:06:25
788000 -- (-23247.809) [-23223.329] (-23317.911) (-23309.405) * (-23313.965) [-23258.440] (-23235.997) (-23325.910) -- 2:06:07
788500 -- (-23250.739) [-23230.570] (-23288.636) (-23305.514) * (-23297.322) (-23256.814) [-23244.625] (-23335.554) -- 2:05:49
789000 -- (-23267.616) [-23223.329] (-23272.377) (-23301.731) * (-23283.791) (-23252.202) [-23227.474] (-23334.090) -- 2:05:31
789500 -- (-23270.405) [-23218.328] (-23276.559) (-23304.586) * (-23277.269) (-23242.883) [-23252.493] (-23306.213) -- 2:05:14
790000 -- (-23267.399) [-23235.323] (-23283.524) (-23322.329) * (-23265.198) (-23243.668) [-23239.824] (-23309.609) -- 2:04:56

Average standard deviation of split frequencies: 0.016592

790500 -- (-23261.275) [-23224.833] (-23293.670) (-23335.912) * (-23263.069) [-23250.613] (-23231.420) (-23304.885) -- 2:04:38
791000 -- (-23248.752) [-23222.691] (-23291.032) (-23321.491) * (-23262.245) (-23259.637) [-23225.447] (-23293.938) -- 2:04:20
791500 -- (-23250.344) [-23233.708] (-23285.278) (-23325.744) * (-23278.612) (-23257.603) [-23225.582] (-23299.094) -- 2:04:02
792000 -- (-23242.203) [-23246.564] (-23289.531) (-23331.528) * (-23259.230) (-23280.643) [-23223.526] (-23308.438) -- 2:03:44
792500 -- (-23250.451) [-23242.552] (-23272.466) (-23318.065) * (-23265.852) (-23281.079) [-23229.152] (-23327.920) -- 2:03:27
793000 -- (-23246.151) [-23246.946] (-23263.020) (-23336.315) * (-23264.862) (-23285.394) [-23240.138] (-23321.409) -- 2:03:09
793500 -- [-23248.982] (-23263.898) (-23270.916) (-23330.801) * [-23251.688] (-23278.314) (-23241.846) (-23326.501) -- 2:02:51
794000 -- (-23273.226) [-23263.348] (-23289.246) (-23343.823) * [-23238.957] (-23271.898) (-23264.556) (-23321.920) -- 2:02:33
794500 -- [-23282.203] (-23247.786) (-23301.718) (-23331.313) * [-23232.590] (-23266.287) (-23271.593) (-23302.391) -- 2:02:16
795000 -- (-23293.950) [-23225.964] (-23308.025) (-23318.196) * [-23228.190] (-23259.567) (-23255.794) (-23296.178) -- 2:01:58

Average standard deviation of split frequencies: 0.016351

795500 -- [-23296.453] (-23244.430) (-23324.665) (-23301.373) * [-23231.327] (-23270.235) (-23261.332) (-23287.133) -- 2:01:40
796000 -- [-23265.865] (-23237.231) (-23309.300) (-23304.586) * [-23238.870] (-23269.941) (-23260.154) (-23279.528) -- 2:01:22
796500 -- (-23264.654) [-23253.999] (-23318.464) (-23293.105) * [-23230.795] (-23274.532) (-23262.318) (-23288.980) -- 2:01:04
797000 -- [-23258.022] (-23258.197) (-23340.814) (-23292.884) * [-23250.075] (-23297.004) (-23274.855) (-23279.876) -- 2:00:47
797500 -- [-23264.614] (-23247.841) (-23347.907) (-23302.225) * [-23237.200] (-23282.694) (-23278.730) (-23285.950) -- 2:00:29
798000 -- [-23254.522] (-23258.967) (-23345.488) (-23289.550) * [-23247.753] (-23277.522) (-23267.743) (-23315.031) -- 2:00:11
798500 -- [-23230.601] (-23259.768) (-23375.857) (-23284.854) * (-23253.089) (-23290.371) [-23256.819] (-23290.918) -- 1:59:53
799000 -- [-23236.278] (-23269.401) (-23381.480) (-23268.059) * [-23254.080] (-23284.760) (-23254.166) (-23290.480) -- 1:59:35
799500 -- [-23240.236] (-23259.396) (-23357.740) (-23288.014) * (-23249.365) (-23278.253) [-23265.930] (-23263.166) -- 1:59:18
800000 -- [-23246.744] (-23261.564) (-23365.147) (-23288.189) * (-23264.075) (-23283.246) [-23254.772] (-23281.947) -- 1:59:00

Average standard deviation of split frequencies: 0.016360

800500 -- (-23244.416) [-23250.265] (-23345.678) (-23291.586) * [-23269.461] (-23284.665) (-23263.205) (-23282.860) -- 1:58:42
801000 -- (-23234.727) [-23243.226] (-23361.468) (-23282.368) * [-23262.024] (-23264.602) (-23260.472) (-23269.513) -- 1:58:24
801500 -- (-23248.131) [-23274.043] (-23369.729) (-23284.807) * [-23270.808] (-23263.123) (-23259.286) (-23264.936) -- 1:58:06
802000 -- [-23221.316] (-23265.787) (-23347.445) (-23282.691) * (-23286.665) (-23260.019) [-23243.995] (-23255.810) -- 1:57:49
802500 -- [-23219.675] (-23266.656) (-23327.824) (-23275.921) * (-23307.438) (-23268.524) [-23247.316] (-23251.447) -- 1:57:31
803000 -- [-23225.092] (-23260.243) (-23304.227) (-23252.413) * (-23325.459) (-23272.252) (-23260.107) [-23237.877] -- 1:57:13
803500 -- [-23219.639] (-23275.902) (-23333.086) (-23251.538) * (-23312.731) (-23264.204) (-23276.655) [-23230.902] -- 1:56:55
804000 -- [-23206.667] (-23286.037) (-23333.197) (-23250.114) * (-23291.008) (-23281.358) (-23254.208) [-23229.825] -- 1:56:37
804500 -- [-23201.002] (-23277.035) (-23339.139) (-23253.111) * (-23276.355) (-23265.437) [-23240.522] (-23257.984) -- 1:56:20
805000 -- [-23209.463] (-23290.239) (-23333.151) (-23267.465) * (-23290.060) (-23258.339) [-23268.579] (-23268.730) -- 1:56:02

Average standard deviation of split frequencies: 0.016351

805500 -- [-23215.024] (-23279.787) (-23317.735) (-23263.884) * (-23302.077) [-23253.973] (-23256.560) (-23271.481) -- 1:55:44
806000 -- [-23221.581] (-23289.045) (-23341.706) (-23286.374) * (-23298.516) (-23259.056) [-23256.469] (-23288.100) -- 1:55:26
806500 -- [-23212.071] (-23304.140) (-23331.267) (-23275.187) * (-23288.485) [-23259.040] (-23265.573) (-23311.160) -- 1:55:09
807000 -- [-23221.180] (-23295.386) (-23325.381) (-23272.163) * (-23280.195) (-23262.231) [-23251.322] (-23308.023) -- 1:54:51
807500 -- [-23224.809] (-23288.452) (-23321.171) (-23245.875) * (-23285.498) [-23240.757] (-23246.795) (-23298.497) -- 1:54:33
808000 -- [-23210.474] (-23282.876) (-23304.727) (-23243.372) * (-23298.320) (-23262.153) [-23264.602] (-23291.779) -- 1:54:15
808500 -- [-23224.574] (-23291.908) (-23287.170) [-23227.354] * (-23274.363) (-23259.643) [-23244.083] (-23272.052) -- 1:53:57
809000 -- [-23225.747] (-23314.907) (-23289.652) [-23228.434] * [-23263.254] (-23269.150) (-23247.251) (-23280.992) -- 1:53:40
809500 -- [-23237.742] (-23311.526) (-23286.195) (-23231.690) * (-23250.772) (-23253.204) [-23259.676] (-23298.011) -- 1:53:21
810000 -- [-23232.958] (-23318.524) (-23283.713) (-23250.922) * (-23252.567) (-23271.053) [-23250.732] (-23287.922) -- 1:53:04
```

Average standard deviation of split frequencies: 0.016771

810500 -- [-23222.101] (-23303.310) (-23259.224) (-23257.801) * (-23260.745) (-23287.930) [-23248.987] (-23275.622) -- 1:52:46
811000 -- [-23235.635] (-23318.354) (-23262.815) (-23248.997) * (-23275.445) (-23279.334) [-23244.592] (-23286.606) -- 1:52:29
811500 -- [-23249.091] (-23282.118) (-23267.417) (-23238.484) * [-23262.456] (-23285.666) (-23232.350) (-23283.793) -- 1:52:11
812000 -- [-23239.600] (-23263.453) (-23299.847) (-23239.742) * (-23276.429) (-23298.210) [-23222.983] (-23272.597) -- 1:51:53
812500 -- (-23261.482) (-23277.077) (-23293.546) [-23229.210] * (-23260.981) (-23302.587) (-23235.981) [-23270.645] -- 1:51:35
813000 -- (-23268.473) (-23295.274) (-23281.934) [-23248.838] * (-23258.344) (-23277.561) [-23231.809] (-23296.417) -- 1:51:17
813500 -- (-23255.573) (-23284.265) (-23299.721) [-23254.800] * (-23254.542) (-23277.502) [-23248.897] (-23273.247) -- 1:51:00
814000 -- [-23256.198] (-23272.607) (-23303.266) (-23264.773) * (-23261.535) (-23298.917) [-23233.898] (-23269.716) -- 1:50:42
814500 -- [-23242.113] (-23271.363) (-23310.803) (-23268.810) * (-23249.274) (-23301.937) [-23246.703] (-23267.916) -- 1:50:24
815000 -- (-23235.869) (-23280.191) (-23322.403) [-23267.898] * [-23247.569] (-23314.689) (-23257.913) (-23259.009) -- 1:50:06

Average standard deviation of split frequencies: 0.016893

815500 -- (-23256.031) (-23281.827) (-23318.974) [-23267.712] * [-23242.049] (-23299.178) (-23242.284) (-23274.327) -- 1:49:48
816000 -- (-23273.395) (-23289.440) (-23308.398) [-23284.458] * (-23251.801) (-23285.305) [-23250.098] (-23279.392) -- 1:49:30
816500 -- (-23271.704) (-23295.651) (-23314.801) [-23261.228] * (-23243.087) (-23280.275) [-23235.917] (-23292.087) -- 1:49:12
817000 -- [-23257.640] (-23292.485) (-23323.017) [-23285.410] * [-23235.603] (-23287.440) (-23257.087) (-23308.903) -- 1:48:54
817500 -- (-23257.611) (-23302.047) (-23326.761) [-23266.561] * [-23244.289] (-23278.403) (-23278.458) (-23297.925) -- 1:48:36
818000 -- (-23266.635) (-23284.944) (-23314.004) [-23267.030] * [-23241.228] (-23267.123) (-23277.567) (-23306.756) -- 1:48:18
818500 -- (-23271.660) (-23296.269) (-23307.879) [-23262.214] * [-23228.907] (-23272.001) (-23271.173) (-23342.941) -- 1:48:00
819000 -- (-23259.114) (-23300.749) (-23302.666) [-23264.496] * [-23229.801] (-23260.154) (-23270.849) (-23322.536) -- 1:47:42
819500 -- [-23272.371] (-23290.129) (-23331.587) (-23275.740) * [-23231.163] (-23263.237) (-23277.245) (-23321.300) -- 1:47:24
820000 -- (-23255.230) (-23273.900) (-23307.570) [-23277.266] * [-23232.267] (-23265.965) (-23264.301) (-23325.905) -- 1:47:06

Average standard deviation of split frequencies: 0.017059

820500 -- [-23247.104] (-23276.471) (-23318.817) (-23277.582) * [-23229.346] (-23289.618) (-23254.158) (-23326.802) -- 1:46:48
821000 -- [-23240.196] (-23270.329) (-23280.075) (-23287.589) * [-23232.736] (-23315.587) (-23262.666) (-23306.318) -- 1:46:30
821500 -- [-23251.854] (-23278.578) (-23275.272) (-23282.638) * [-23245.628] (-23305.075) (-23261.424) (-23285.763) -- 1:46:12
822000 -- (-23243.007) [-23271.138] (-23279.291) (-23278.139) * [-23235.083] (-23320.976) (-23287.282) (-23307.901) -- 1:45:54
822500 -- [-23245.120] (-23297.148) (-23276.241) (-23286.264) * [-23239.892] (-23325.872) (-23266.527) (-23291.920) -- 1:45:36
823000 -- [-23241.752] (-23318.939) (-23264.319) (-23296.429) * [-23228.006] (-23331.541) (-23268.709) (-23263.908) -- 1:45:18
823500 -- [-23251.674] (-23299.651) (-23263.453) (-23317.350) * [-23223.936] (-23300.681) (-23247.305) (-23263.022) -- 1:45:01
824000 -- [-23258.894] (-23302.886) (-23306.663) (-23296.463) * [-23222.047] (-23309.589) (-23249.522) (-23269.546) -- 1:44:43
824500 -- (-23259.816) (-23308.501) [-23282.600] (-23305.863) * [-23217.742] (-23314.835) (-23256.164) (-23272.214) -- 1:44:25
825000 -- [-23271.930] (-23320.006) (-23309.650) (-23318.086) * [-23229.205] (-23308.252) (-23266.037) (-23257.026) -- 1:44:07

Average standard deviation of split frequencies: 0.016944

825500 -- [-23287.035] (-23293.761) (-23310.694) (-23314.216) * [-23249.529] (-23307.912) (-23268.478) (-23254.089) -- 1:43:49
826000 -- (-23289.384) (-23282.721) (-23281.750) (-23305.725) * [-23263.156] (-23307.720) (-23284.199) [-23270.765] -- 1:43:31
826500 -- [-23278.151] (-23292.794) (-23270.179) (-23311.269) * (-23257.070) (-23312.859) (-23277.592) [-23250.947] -- 1:43:14
827000 -- (-23288.887) (-23282.941) [-23266.039] (-23328.173) * (-23279.372) (-23310.974) (-23280.436) [-23246.527] -- 1:42:56
827500 -- (-23283.365) (-23275.903) [-23264.783] (-23338.809) * (-23264.991) (-23329.708) (-23286.770) [-23240.924] -- 1:42:38
828000 -- (-23281.428) (-23274.240) [-23249.213] (-23349.342) * (-23250.163) (-23331.458) (-23306.688) [-23236.926] -- 1:42:20
828500 -- (-23275.644) [-23257.151] (-23249.427) (-23330.602) * [-23261.232] (-23325.858) (-23301.758) (-23271.254) -- 1:42:03
829000 -- (-23288.502) (-23253.259) [-23249.858] (-23329.108) * [-23260.860] (-23326.685) (-23302.930) (-23262.426) -- 1:41:45
829500 -- (-23299.700) (-23237.430) [-23245.571] (-23321.883) * [-23278.222] (-23321.792) (-23299.295) (-23258.406) -- 1:41:27
830000 -- (-23293.425) [-23242.330] (-23254.277) (-23293.023) * (-23279.998) (-23304.308) (-23309.185) [-23253.673] -- 1:41:09

Average standard deviation of split frequencies: 0.016877

830500 -- (-23288.010) [-23242.712] (-23247.592) (-23318.804) * [-23272.345] (-23301.824) (-23307.005) (-23257.701) -- 1:40:51
831000 -- (-23285.239) (-23249.754) [-23247.061] (-23323.622) * [-23254.375] (-23309.474) (-23302.377) (-23291.045) -- 1:40:33
831500 -- (-23283.061) (-23251.226) [-23232.105] (-23320.011) * [-23245.473] (-23302.486) (-23303.341) (-23281.141) -- 1:40:15
832000 -- (-23293.050) (-23248.658) [-23237.846] (-23318.557) * [-23253.967] (-23317.295) (-23300.408) (-23266.563) -- 1:39:57
832500 -- (-23288.853) (-23250.358) [-23231.491] (-23311.941) * [-23252.679] (-23324.262) (-23311.456) (-23260.679) -- 1:39:40
833000 -- (-23267.907) (-23262.483) [-23237.131] (-23288.766) * (-23266.724) (-23314.523) (-23285.756) [-23253.811] -- 1:39:21
833500 -- (-23298.138) (-23270.143) [-23232.317] (-23312.129) * (-23272.783) (-23309.988) (-23273.440) [-23249.127] -- 1:39:03
834000 -- (-23294.820) (-23265.031) [-23231.256] (-23328.537) * (-23270.215) (-23313.089) (-23265.607) [-23252.279] -- 1:38:46
834500 -- (-23295.190) (-23263.630) [-23249.949] (-23314.166) * (-23258.181) (-23321.956) [-23260.922] (-23247.721) -- 1:38:28
835000 -- (-23295.435) [-23233.930] (-23240.562) (-23315.687) * (-23259.144) (-23298.705) (-23270.201) [-23257.093] -- 1:38:10

Average standard deviation of split frequencies: 0.016843

835500 -- (-23304.468) [-23246.194] (-23238.352) (-23318.486) * (-23252.633) (-23287.088) [-23245.535] (-23275.909) -- 1:37:52
836000 -- (-23322.690) (-23249.025) [-23235.935] (-23310.695) * [-23248.578] (-23307.192) (-23267.689) (-23284.075) -- 1:37:34
836500 -- (-23311.516) [-23247.639] (-23248.216) (-23295.675) * [-23243.621] (-23311.257) (-23251.063) (-23287.177) -- 1:37:16
837000 -- (-23322.823) [-23245.344] (-23246.689) (-23326.657) * [-23248.993] (-23310.500) (-23257.236) (-23286.770) -- 1:36:58
837500 -- (-23326.749) (-23273.784) [-23248.652] (-23318.757) * [-23246.093] (-23319.957) (-23260.513) (-23297.786) -- 1:36:40
838000 -- (-23337.207) (-23262.595) [-23258.412] (-23309.561) * (-23253.375) (-23297.780) [-23246.140] (-23314.819) -- 1:36:22
838500 -- (-23324.086) (-23256.051) [-23267.013] (-23324.692) * (-23259.822) (-23297.964) [-23230.363] (-23312.475) -- 1:36:04
839000 -- (-23312.612) [-23242.467] (-23242.957) (-23315.152) * (-23264.900) (-23305.974) [-23245.481] (-23313.549) -- 1:35:47
839500 -- (-23287.382) (-23259.349) [-23246.705] (-23328.612) * (-23262.486) (-23317.777) [-23242.664] (-23312.561) -- 1:35:29
840000 -- (-23282.019) (-23265.043) [-23253.843] (-23331.018) * (-23283.180) (-23334.684) [-23227.599] (-23305.707) -- 1:35:11

Average standard deviation of split frequencies: 0.016619

840500 -- (-23267.900) (-23258.097) [-23238.051] (-23325.477) * (-23259.483) (-23339.183) [-23228.495] (-23298.828) -- 1:34:53
841000 -- (-23274.456) (-23253.942) [-23233.118] (-23319.030) * (-23275.043) (-23330.751) [-23229.264] (-23285.322) -- 1:34:35
841500 -- (-23270.342) (-23263.555) [-23222.096] (-23325.948) * (-23254.223) (-23312.969) [-23242.417] (-23269.006) -- 1:34:18
842000 -- (-23292.119) (-23252.287) [-23227.650] (-23324.411) * [-23251.498] (-23296.736) (-23247.917) (-23263.133) -- 1:34:00
842500 -- (-23319.100) (-23243.996) [-23235.086] (-23325.685) * [-23242.230] (-23273.327) (-23242.311) (-23270.796) -- 1:33:42
843000 -- (-23287.439) (-23238.895) [-23236.205] (-23324.610) * [-23252.048] (-23277.510) (-23263.574) (-23264.335) -- 1:33:24
843500 -- (-23278.755) [-23251.234] (-23241.018) (-23317.117) * [-23260.997] (-23259.717) (-23282.961) (-23256.076) -- 1:33:07
844000 -- (-23262.048) (-23251.769) [-23241.666] (-23296.411) * [-23253.389] (-23261.740) (-23281.161) (-23274.026) -- 1:32:49
844500 -- (-23267.937) (-23252.708) [-23241.280] (-23314.921) * [-23243.628] (-23256.350) (-23276.659) (-23267.527) -- 1:32:31
845000 -- (-23277.009) (-23247.228) [-23245.093] (-23323.574) * [-23238.459] (-23262.743) (-23250.353) (-23273.626) -- 1:32:13

Average standard deviation of split frequencies: 0.016527

845500 -- (-23275.139) [-23238.210] (-23252.801) (-23316.459) * (-23250.912) (-23256.785) [-23267.470] (-23298.911) -- 1:31:56
846000 -- (-23295.092) [-23251.795] (-23258.304) (-23340.108) * [-23244.524] (-23258.748) (-23273.644) (-23309.846) -- 1:31:38
846500 -- (-23290.403) (-23236.425) [-23253.957] (-23322.563) * [-23239.350] (-23262.911) (-23273.881) (-23314.185) -- 1:31:20
847000 -- (-23288.610) [-23211.198] (-23261.689) (-23322.481) * [-23226.735] (-23267.527) (-23276.986) (-23314.743) -- 1:31:03
847500 -- (-23297.143) [-23231.718] (-23245.881) (-23313.548) * [-23231.185] (-23295.788) (-23286.223) (-23306.805) -- 1:30:45
848000 -- (-23280.324) [-23218.147] (-23248.763) (-23293.039) * [-23227.238] (-23285.546) (-23272.630) (-23314.753) -- 1:30:27
848500 -- (-23283.164) [-23226.484] (-23241.101) (-23299.997) * [-23222.535] (-23290.777) (-23276.897) (-23294.082) -- 1:30:09
849000 -- (-23286.890) [-23235.145] (-23256.637) (-23315.283) * [-23234.569] (-23269.338) (-23278.452) (-23294.260) -- 1:29:51
849500 -- (-23289.038) (-23246.239) [-23257.035] (-23293.469) * [-23222.312] (-23266.929) (-23273.271) (-23312.311) -- 1:29:33
850000 -- (-23273.184) [-23249.024] (-23261.046) (-23281.044) * [-23234.567] (-23271.737) (-23280.542) (-23337.271) -- 1:29:15

Average standard deviation of split frequencies: 0.016420

850500 -- (-23292.801) (-23253.753) [-23245.797] (-23281.318) * [-23249.060] (-23270.171) (-23297.803) (-23324.689) -- 1:28:58

851000 -- (-23314.537) [-23243.817] (-23261.293) (-23275.117) * [-23258.222] (-23271.387) (-23285.809) (-23315.995) -- 1:28:40
851500 -- (-23343.414) [-23243.910] (-23262.644) (-23277.050) * [-23254.260] (-23269.669) (-23269.065) (-23306.776) -- 1:28:22
852000 -- (-23311.793) [-23221.682] (-23266.418) (-23259.528) * [-23244.425] (-23272.004) (-23265.220) (-23310.332) -- 1:28:04
852500 -- (-23307.282) [-23206.707] (-23256.300) (-23262.411) * [-23244.190] (-23262.789) (-23271.175) (-23315.221) -- 1:27:46
853000 -- (-23298.760) [-23216.099] (-23265.015) (-23275.618) * [-23261.690] (-23270.754) (-23289.958) (-23320.365) -- 1:27:28
853500 -- (-23306.713) [-23222.723] (-23264.223) (-23287.376) * [-23266.392] (-23251.692) (-23298.749) (-23296.387) -- 1:27:10
854000 -- (-23302.387) [-23217.739] (-23265.879) (-23282.685) * [-23259.517] (-23242.743) (-23301.965) (-23292.394) -- 1:26:52
854500 -- (-23320.712) [-23214.215] (-23273.142) (-23301.011) * [-23256.625] (-23240.319) (-23298.769) (-23298.132) -- 1:26:35
855000 -- (-23347.391) [-23216.593] (-23259.550) (-23300.279) * (-23247.990) [-23220.391] (-23324.653) (-23291.386) -- 1:26:17

Average standard deviation of split frequencies: 0.016249

855500 -- (-23322.273) [-23221.148] (-23248.625) (-23285.980) * (-23242.378) [-23224.121] (-23331.489) (-23292.971) -- 1:25:59
856000 -- (-23314.110) [-23224.996] (-23266.319) (-23295.769) * (-23241.187) [-23238.026] (-23354.349) (-23297.701) -- 1:25:41
856500 -- (-23311.239) [-23222.701] (-23288.255) (-23294.178) * [-23238.296] (-23226.692) (-23325.392) (-23283.399) -- 1:25:23
857000 -- (-23325.522) [-23226.369] (-23288.086) (-23267.842) * [-23243.105] (-23233.683) (-23335.602) (-23273.883) -- 1:25:05
857500 -- (-23302.407) (-23235.832) (-23268.709) [-23247.665] * [-23249.794] (-23255.572) (-23335.720) (-23276.117) -- 1:24:47
858000 -- (-23325.034) [-23233.624] (-23266.071) (-23252.398) * [-23255.188] (-23261.340) (-23329.448) (-23303.719) -- 1:24:29
858500 -- (-23324.028) (-23256.855) (-23274.165) [-23229.911] * (-23283.184) [-23242.899] (-23302.077) (-23315.206) -- 1:24:12
859000 -- (-23307.247) [-23237.459] (-23298.430) (-23232.185) * (-23273.121) [-23232.101] (-23303.939) (-23315.022) -- 1:23:54
859500 -- (-23302.916) (-23225.132) (-23286.729) [-23207.975] * (-23270.172) [-23250.370] (-23278.852) (-23313.424) -- 1:23:36
860000 -- (-23305.323) (-23237.938) (-23268.459) [-23214.761] * [-23244.178] (-23268.733) (-23253.378) (-23340.535) -- 1:23:18

Average standard deviation of split frequencies: 0.016020

860500 -- (-23311.132) (-23245.712) (-23286.275) [-23226.657] * (-23257.461) (-23261.787) [-23239.420] (-23321.747) -- 1:23:00
861000 -- (-23305.042) (-23248.946) (-23305.143) [-23225.489] * (-23271.399) [-23236.343] (-23260.968) (-23348.065) -- 1:22:43
861500 -- (-23299.502) [-23237.788] (-23299.865) (-23243.387) * [-23255.786] (-23234.313) (-23274.670) (-23349.020) -- 1:22:25
862000 -- (-23284.288) [-23240.000] (-23313.042) (-23262.513) * (-23251.490) (-23232.089) [-23242.636] (-23344.560) -- 1:22:08
862500 -- (-23281.466) [-23240.786] (-23301.486) (-23287.387) * (-23251.371) [-23231.608] (-23250.163) (-23323.145) -- 1:21:50
863000 -- (-23269.543) [-23245.880] (-23295.501) (-23285.793) * (-23261.475) [-23245.182] (-23262.895) (-23313.923) -- 1:21:32
863500 -- (-23261.737) [-23255.166] (-23294.173) (-23297.688) * [-23262.852] (-23258.030) (-23264.762) (-23307.978) -- 1:21:15
864000 -- (-23252.174) (-23271.784) (-23285.137) (-23279.775) * (-23257.121) [-23251.872] (-23272.082) (-23297.843) -- 1:20:57
864500 -- [-23252.117] (-23278.136) (-23275.055) (-23271.142) * [-23242.168] (-23250.607) (-23278.607) (-23318.141) -- 1:20:39
865000 -- [-23243.515] (-23256.988) (-23288.190) (-23269.979) * (-23228.734) [-23238.690] (-23284.324) (-23318.660) -- 1:20:22

Average standard deviation of split frequencies: 0.015835

865500 -- [-23228.589] (-23268.234) (-23311.332) (-23272.734) * [-23235.871] (-23245.789) (-23288.408) (-23311.354) -- 1:20:04
866000 -- [-23226.817] (-23268.669) (-23300.067) (-23268.018) * [-23238.355] (-23258.372) (-23287.048) (-23293.063) -- 1:19:46
866500 -- [-23228.922] (-23258.894) (-23288.726) (-23271.477) * [-23226.615] (-23268.671) (-23296.986) (-23275.984) -- 1:19:28
867000 -- [-23220.953] (-23263.522) (-23275.487) (-23249.202) * [-23218.172] (-23262.048) (-23285.611) (-23263.444) -- 1:19:10
867500 -- [-23213.400] (-23281.715) (-23249.024) (-23248.694) * [-23228.086] (-23276.169) (-23291.212) (-23270.459) -- 1:18:53
868000 -- [-23224.164] (-23291.124) (-23254.597) (-23264.911) * [-23237.478] (-23274.510) (-23299.530) (-23278.504) -- 1:18:35
868500 -- [-23217.854] (-23285.110) (-23263.372) (-23269.540) * [-23225.558] (-23280.350) (-23306.980) (-23266.935) -- 1:18:17
869000 -- [-23225.913] (-23310.277) (-23267.588) (-23268.382) * [-23230.469] (-23300.872) (-23279.178) (-23254.153) -- 1:17:59
869500 -- [-23214.789] (-23311.390) (-23278.381) (-23266.412) * [-23239.969] (-23296.510) (-23288.585) (-23264.763) -- 1:17:41
870000 -- [-23223.821] (-23338.311) (-23278.816) (-23270.044) * [-23243.480] (-23294.548) (-23312.740) (-23263.296) -- 1:17:24

Average standard deviation of split frequencies: 0.015452

870500 -- [-23229.806] (-23344.401) (-23269.827) (-23257.315) * [-23246.279] (-23277.445) (-23323.404) (-23257.017) -- 1:17:06
871000 -- [-23225.068] (-23338.345) (-23263.585) (-23269.446) * [-23252.985] (-23281.137) (-23342.174) (-23262.388) -- 1:16:48
871500 -- [-23238.872] (-23337.720) (-23271.229) (-23283.004) * [-23241.343] (-23288.966) (-23357.027) (-23254.805) -- 1:16:30
872000 -- [-23255.510] (-23331.970) (-23287.135) (-23268.767) * [-23243.213] (-23274.268) (-23359.692) (-23257.368) -- 1:16:12
872500 -- [-23238.592] (-23328.619) (-23290.111) (-23263.503) * [-23246.076] (-23276.101) (-23304.320) (-23260.127) -- 1:15:54
873000 -- [-23222.587] (-23326.987) (-23273.607) (-23262.867) * [-23230.618] (-23289.374) (-23314.980) (-23269.525) -- 1:15:37
873500 -- [-23230.767] (-23305.111) (-23274.050) (-23252.721) * [-23222.602] (-23291.000) (-23283.521) (-23277.428) -- 1:15:19
874000 -- [-23233.469] (-23299.115) (-23272.793) (-23249.824) * [-23222.708] (-23279.848) (-23299.042) (-23283.467) -- 1:15:01
874500 -- [-23233.503] (-23294.221) (-23287.749) (-23252.482) * [-23218.163] (-23306.468) (-23299.681) (-23292.087) -- 1:14:43
875000 -- [-23233.035] (-23283.253) (-23295.300) (-23255.376) * [-23214.010] (-23289.967) (-23301.115) (-23279.787) -- 1:14:25

Average standard deviation of split frequencies: 0.015426

875500 -- [-23247.853] (-23290.313) (-23291.935) (-23249.397) * [-23218.247] (-23303.124) (-23325.563) (-23287.164) -- 1:14:07
876000 -- [-23236.675] (-23274.345) (-23295.111) (-23270.852) * [-23228.012] (-23317.168) (-23301.893) (-23273.390) -- 1:13:49
876500 -- [-23241.519] (-23279.064) (-23271.660) (-23260.062) * [-23230.768] (-23330.764) (-23284.785) (-23279.413) -- 1:13:31
877000 -- [-23224.428] (-23273.528) (-23267.890) (-23270.549) * [-23238.508] (-23327.579) (-23288.619) (-23272.593) -- 1:13:13
877500 -- [-23223.019] (-23264.413) (-23267.274) (-23278.599) * [-23246.911] (-23310.313) (-23267.338) (-23275.256) -- 1:12:55
878000 -- [-23240.495] (-23275.489) (-23310.927) (-23278.231) * [-23249.740] (-23302.875) (-23263.998) (-23283.786) -- 1:12:37
878500 -- [-23239.392] (-23276.245) (-23323.200) (-23263.108) * (-23252.618) (-23306.774) [-23262.541] (-23274.671) -- 1:12:19
879000 -- [-23238.848] (-23277.935) (-23330.212) (-23277.030) * [-23236.824] (-23309.994) (-23260.648) (-23270.811) -- 1:12:01
879500 -- [-23236.182] (-23274.576) (-23332.407) (-23279.365) * (-23253.140) (-23315.185) [-23228.505] (-23283.363) -- 1:11:44
880000 -- [-23238.924] (-23265.217) (-23342.723) (-23281.606) * [-23252.037] (-23326.646) (-23242.643) (-23306.073) -- 1:11:26

Average standard deviation of split frequencies: 0.015438

880500 -- [-23244.407] (-23262.709) (-23334.418) (-23284.395) * [-23243.908] (-23318.818) (-23229.606) (-23285.753) -- 1:11:08
881000 -- [-23258.427] (-23257.932) (-23311.012) (-23282.169) * [-23255.637] (-23288.396) [-23229.237] (-23278.231) -- 1:10:50
881500 -- (-23240.252) [-23239.918] (-23320.054) (-23280.115) * (-23256.111) (-23297.889) [-23212.174] (-23273.961) -- 1:10:32
882000 -- [-23238.255] (-23237.641) (-23335.043) (-23285.634) * (-23246.845) (-23300.260) [-23224.629] (-23279.629) -- 1:10:14
882500 -- (-23250.137) [-23241.199] (-23328.434) (-23306.086) * [-23246.358] (-23302.563) (-23251.271) (-23294.597) -- 1:09:57
883000 -- (-23279.046) [-23226.862] (-23345.114) (-23283.760) * [-23248.413] (-23296.613) (-23240.184) (-23299.762) -- 1:09:39
883500 -- (-23262.276) [-23231.265] (-23325.497) (-23297.821) * (-23251.616) (-23317.966) [-23225.192] (-23283.289) -- 1:09:21
884000 -- (-23269.020) [-23237.164] (-23343.405) (-23277.400) * (-23264.912) (-23333.455) [-23242.476] (-23290.422) -- 1:09:03
884500 -- (-23287.394) [-23243.114] (-23345.924) (-23304.041) * (-23274.613) (-23330.409) (-23266.245) [-23272.534] -- 1:08:45
885000 -- [-23274.440] (-23260.242) (-23349.304) (-23313.783) * (-23295.853) (-23327.808) [-23258.700] (-23276.478) -- 1:08:27

Average standard deviation of split frequencies: 0.015546

885500 -- [-23268.090] (-23274.867) (-23363.261) (-23304.951) * (-23275.063) (-23310.019) [-23253.794] (-23281.668) -- 1:08:09
886000 -- [-23264.424] (-23261.775) (-23348.419) (-23301.046) * (-23280.681) (-23302.927) [-23247.307] (-23278.222) -- 1:07:51
886500 -- [-23264.504] (-23273.512) (-23343.818) (-23300.729) * (-23262.466) (-23298.488) (-23241.877) (-23265.135) -- 1:07:33
887000 -- [-23271.915] (-23266.242) (-23346.757) (-23320.425) * (-23260.874) (-23288.757) [-23251.111] (-23284.814) -- 1:07:16
887500 -- [-23263.657] (-23283.907) (-23366.052) (-23300.757) * (-23261.789) (-23296.512) [-23247.155] (-23304.636) -- 1:06:58
888000 -- [-23257.840] (-23255.286) (-23326.252) (-23321.225) * (-23255.593) (-23318.520) [-23253.292] (-23286.220) -- 1:06:40
888500 -- (-23271.693) [-23252.839] (-23335.302) (-23321.819) * (-23262.724) (-23334.370) [-23257.612] (-23297.095) -- 1:06:22
889000 -- (-23268.197) [-23227.123] (-23337.940) (-23309.132) * (-23235.735) (-23343.053) [-23261.722] (-23314.999) -- 1:06:04
889500 -- (-23283.813) [-23234.811] (-23312.823) (-23321.312) * [-23253.722] (-23332.523) (-23271.493) (-23299.077) -- 1:05:46
890000 -- (-23290.980) [-23235.451] (-23323.892) (-23310.521) * [-23248.632] (-23336.240) (-23283.469) (-23279.682) -- 1:05:28

Average standard deviation of split frequencies: 0.015639

890500 -- (-23298.671) [-23231.152] (-23321.115) (-23318.009) * [-23244.606] (-23319.629) (-23276.622) (-23264.517) -- 1:05:11
891000 -- (-23276.237) [-23225.391] (-23313.307) (-23321.329) * [-23237.698] (-23303.292) (-23282.944) (-23258.140) -- 1:04:53
891500 -- (-23286.120) [-23233.768] (-23308.650) (-23322.854) * [-23248.262] (-23319.337) (-23282.951) (-23262.315) -- 1:04:35
892000 -- (-23262.682) [-23232.991] (-23299.525) (-23340.873) * [-23247.697] (-23302.687) (-23299.643) (-23259.304) -- 1:04:17
892500 -- (-23260.267) [-23231.071] (-23268.550) (-23324.951) * [-23251.702] (-23318.404) (-23311.486) (-23257.585) -- 1:03:59

```
893000 -- (-23254.682) [-23243.391] (-23285.346) (-23319.742) * [-23246.936] (-23341.905) (-23308.853) (-23256.878) -- 1:03:41
893500 -- (-23259.923) [-23258.718] (-23276.556) (-23309.727) * (-23243.954) (-23333.292) (-23299.662) [-23251.646] -- 1:03:23
894000 -- (-23256.153) [-23237.233] (-23273.288) (-23305.940) * [-23233.422] (-23323.934) (-23287.859) (-23265.437) -- 1:03:06
894500 -- (-23254.165) [-23245.925] (-23286.790) (-23298.999) * [-23225.251] (-23301.692) (-23280.240) (-23273.261) -- 1:02:48
895000 -- (-23257.756) [-23232.251] (-23280.227) (-23308.965) * [-23236.974] (-23291.863) (-23279.867) (-23267.795) -- 1:02:30

Average standard deviation of split frequencies: 0.015589

895500 -- (-23253.853) [-23219.584] (-23293.094) (-23288.454) * [-23240.286] (-23286.491) (-23292.713) (-23291.161) -- 1:02:12
896000 -- (-23258.094) [-23220.749] (-23262.693) (-23296.655) * [-23233.527] (-23283.268) (-23294.209) (-23306.291) -- 1:01:54
896500 -- (-23262.793) [-23226.454] (-23292.011) (-23295.390) * [-23225.916] (-23278.467) (-23298.322) (-23314.135) -- 1:01:37
897000 -- [-23258.686] (-23255.651) (-23281.616) (-23298.265) * [-23236.568] (-23282.672) (-23283.758) (-23308.050) -- 1:01:19
897500 -- [-23247.063] (-23267.800) (-23278.081) (-23321.263) * [-23236.431] (-23283.195) (-23281.076) (-23326.847) -- 1:01:01
898000 -- [-23243.815] (-23244.560) (-23269.055) (-23308.118) * [-23224.870] (-23281.739) (-23269.320) (-23321.737) -- 1:00:43
898500 -- [-23230.486] (-23266.894) (-23294.548) (-23325.425) * [-23224.389] (-23286.745) (-23266.647) (-23322.053) -- 1:00:25
899000 -- [-23226.064] (-23265.558) (-23275.288) (-23339.029) * [-23231.116] (-23293.988) (-23272.012) (-23332.724) -- 1:00:08
899500 -- [-23215.232] (-23279.160) (-23281.072) (-23321.060) * [-23239.251] (-23286.697) (-23274.376) (-23319.217) -- 0:59:50
900000 -- [-23227.458] (-23287.522) (-23293.874) (-23312.525) * [-23270.719] (-23281.183) (-23297.746) (-23320.407) -- 0:59:32

Average standard deviation of split frequencies: 0.015667

900500 -- [-23222.271] (-23289.699) (-23301.669) (-23311.388) * [-23274.841] (-23269.795) (-23313.399) (-23327.127) -- 0:59:14
901000 -- [-23224.226] (-23284.280) (-23307.083) (-23319.976) * [-23261.930] (-23263.014) (-23311.865) (-23307.785) -- 0:58:56
901500 -- [-23227.033] (-23250.837) (-23310.656) (-23326.365) * (-23243.641) [-23255.059] (-23284.846) (-23308.501) -- 0:58:39
902000 -- [-23210.390] (-23239.536) (-23294.103) (-23314.639) * [-23240.796] (-23233.860) (-23304.163) (-23312.278) -- 0:58:21
902500 -- [-23219.938] (-23241.495) (-23278.449) (-23312.572) * [-23222.529] (-23243.556) (-23319.763) (-23316.604) -- 0:58:03
903000 -- [-23223.837] (-23246.349) (-23297.333) (-23310.683) * (-23244.554) [-23236.003] (-23331.214) (-23312.732) -- 0:57:45
903500 -- [-23229.816] (-23260.084) (-23309.737) (-23297.375) * (-23265.136) [-23246.937] (-23334.148) (-23311.613) -- 0:57:27
904000 -- [-23220.964] (-23257.448) (-23327.007) (-23294.991) * [-23256.110] (-23260.144) (-23307.711) (-23297.032) -- 0:57:09
904500 -- [-23220.549] (-23269.584) (-23324.529) (-23283.425) * [-23256.883] (-23266.643) (-23302.092) (-23303.429) -- 0:56:51
905000 -- [-23227.736] (-23274.175) (-23320.560) (-23294.796) * (-23270.492) [-23261.187] (-23288.399) (-23298.919) -- 0:56:34

Average standard deviation of split frequencies: 0.015687

905500 -- [-23218.900] (-23256.694) (-23311.939) (-23298.066) * (-23270.689) [-23264.440] (-23300.142) (-23288.285) -- 0:56:16
906000 -- [-23224.199] (-23254.404) (-23339.647) (-23293.528) * (-23246.428) [-23272.257] (-23310.487) (-23280.275) -- 0:55:58
906500 -- [-23232.145] (-23236.207) (-23324.211) (-23284.791) * [-23222.962] (-23261.690) (-23305.955) (-23295.773) -- 0:55:40
907000 -- [-23219.345] (-23241.023) (-23310.407) (-23286.455) * [-23237.866] (-23250.372) (-23318.487) (-23295.536) -- 0:55:22
907500 -- [-23214.744] (-23256.974) (-23331.331) (-23282.188) * [-23245.903] (-23243.719) (-23298.789) (-23286.576) -- 0:55:04
908000 -- [-23232.874] (-23253.830) (-23305.055) (-23276.370) * [-23232.988] (-23235.249) (-23289.025) (-23298.375) -- 0:54:46
908500 -- [-23232.995] (-23244.275) (-23309.661) (-23281.062) * (-23217.940) [-23233.274] (-23284.088) (-23292.197) -- 0:54:28
909000 -- [-23229.616] (-23253.787) (-23310.679) (-23289.086) * (-23227.401) [-23228.685] (-23283.034) (-23280.949) -- 0:54:10
909500 -- (-23243.798) [-23251.724] (-23316.271) (-23283.260) * (-23264.927) [-23239.178] (-23283.106) (-23269.408) -- 0:53:53
910000 -- [-23251.132] (-23255.621) (-23293.714) (-23287.648) * (-23254.252) [-23270.617] (-23291.412) (-23270.131) -- 0:53:35

Average standard deviation of split frequencies: 0.015709

910500 -- (-23254.293) [-23241.643] (-23273.723) (-23282.660) * [-23232.832] (-23263.129) (-23313.952) (-23267.024) -- 0:53:17
911000 -- (-23257.238) [-23248.774] (-23264.764) (-23272.482) * [-23240.620] (-23270.728) (-23312.476) (-23273.034) -- 0:52:59
911500 -- (-23268.696) [-23265.138] (-23287.920) (-23262.825) * [-23234.035] (-23274.447) (-23317.654) (-23265.570) -- 0:52:41
912000 -- (-23287.085) (-23273.583) (-23278.604) [-23241.891] * [-23235.236] (-23297.623) (-23324.649) (-23282.589) -- 0:52:23
912500 -- (-23289.445) (-23284.553) (-23268.571) [-23234.421] * [-23233.637] (-23279.168) (-23311.821) (-23285.041) -- 0:52:05
913000 -- (-23293.431) (-23281.109) [-23262.851] (-23247.844) * [-23222.224] (-23278.740) (-23284.566) (-23281.004) -- 0:51:48
913500 -- (-23304.430) (-23266.979) [-23238.460] (-23244.161) * [-23219.988] (-23290.375) (-23296.600) (-23278.464) -- 0:51:30
914000 -- (-23291.152) (-23267.530) (-23253.930) [-23233.423] * [-23220.445] (-23296.152) (-23304.261) (-23267.515) -- 0:51:12
914500 -- (-23303.365) [-23259.161] (-23258.023) (-23232.409) * [-23230.762] (-23307.607) (-23323.802) (-23255.767) -- 0:50:54
915000 -- (-23317.112) (-23265.887) [-23243.861] (-23241.974) * [-23265.056] (-23315.234) (-23307.100) (-23280.139) -- 0:50:36

Average standard deviation of split frequencies: 0.015717

915500 -- (-23299.258) (-23257.104) [-23247.687] (-23259.032) * [-23260.432] (-23290.038) (-23300.738) (-23258.995) -- 0:50:18
916000 -- (-23286.447) [-23242.103] (-23263.794) (-23256.452) * (-23257.675) (-23308.882) (-23299.386) [-23248.871] -- 0:50:00
916500 -- (-23277.988) [-23258.259] (-23272.700) (-23246.465) * (-23267.938) (-23314.913) (-23285.233) [-23241.951] -- 0:49:43
917000 -- (-23276.045) [-23269.537] (-23306.232) (-23247.266) * (-23247.870) (-23298.832) (-23302.956) [-23235.394] -- 0:49:25
917500 -- (-23280.951) [-23266.847] (-23298.424) (-23246.640) * (-23262.365) (-23281.313) (-23294.317) [-23251.367] -- 0:49:07
918000 -- (-23297.754) (-23280.641) (-23299.776) [-23236.209] * (-23261.865) (-23291.081) (-23309.935) (-23263.985) -- 0:48:49
918500 -- (-23294.202) (-23290.667) (-23302.055) [-23243.795] * (-23256.053) (-23281.682) (-23291.933) (-23247.782) -- 0:48:31
919000 -- (-23303.570) (-23280.290) (-23290.894) [-23235.638] * [-23241.855] (-23273.222) (-23297.600) (-23263.199) -- 0:48:13
919500 -- (-23291.245) (-23267.906) (-23300.018) [-23254.470] * [-23244.150] (-23275.622) (-23317.486) (-23275.785) -- 0:47:55
920000 -- [-23272.752] (-23279.993) (-23293.334) (-23267.026) * [-23245.375] (-23244.862) (-23322.064) (-23272.155) -- 0:47:38

Average standard deviation of split frequencies: 0.015751

920500 -- [-23252.033] (-23294.101) (-23281.696) (-23281.806) * (-23238.010) [-23241.682] (-23312.336) (-23275.584) -- 0:47:20
921000 -- (-23240.188) (-23280.961) [-23262.298] (-23283.743) * (-23242.373) [-23233.345] (-23315.752) (-23271.192) -- 0:47:02
921500 -- [-23231.802] (-23278.341) (-23249.286) (-23267.951) * [-23241.120] (-23240.266) (-23319.585) (-23280.028) -- 0:46:44
922000 -- [-23223.821] (-23290.992) (-23259.767) (-23269.548) * (-23242.262) [-23216.831] (-23316.755) (-23290.006) -- 0:46:26
922500 -- [-23229.210] (-23279.341) (-23262.731) (-23275.433) * (-23258.206) [-23225.840] (-23320.630) (-23310.103) -- 0:46:08
923000 -- [-23256.104] (-23292.813) (-23278.054) (-23294.813) * (-23250.200) [-23220.497] (-23302.242) (-23307.006) -- 0:45:50
923500 -- [-23248.622] (-23279.465) (-23274.659) (-23291.243) * (-23255.097) [-23232.879] (-23315.321) (-23306.664) -- 0:45:33
924000 -- [-23249.331] (-23265.631) (-23255.855) (-23279.586) * (-23267.708) [-23243.407] (-23326.360) (-23318.135) -- 0:45:15
924500 -- (-23258.232) [-23257.073] (-23265.632) (-23260.263) * (-23266.391) [-23220.084] (-23350.773) (-23309.913) -- 0:44:57
925000 -- (-23261.702) [-23264.030] (-23275.587) (-23273.467) * (-23266.450) [-23222.465] (-23344.876) (-23277.743) -- 0:44:39

Average standard deviation of split frequencies: 0.015749

925500 -- [-23268.903] (-23263.989) (-23266.921) (-23288.695) * (-23276.544) [-23230.669] (-23335.701) (-23271.281) -- 0:44:21
926000 -- (-23279.168) [-23253.117] (-23269.200) (-23281.848) * (-23271.833) [-23238.121] (-23331.419) (-23286.478) -- 0:44:03
926500 -- (-23260.076) [-23249.944] (-23262.034) (-23273.816) * (-23264.202) [-23235.996] (-23327.275) (-23271.998) -- 0:43:45
927000 -- [-23235.501] (-23257.667) (-23276.793) (-23290.700) * (-23266.707) [-23235.047] (-23311.467) (-23275.975) -- 0:43:28
927500 -- [-23221.312] (-23253.951) (-23259.054) (-23305.370) * (-23283.792) [-23254.338] (-23303.251) (-23271.427) -- 0:43:10
928000 -- [-23233.885] (-23274.100) (-23268.865) (-23298.127) * (-23277.863) [-23254.757] (-23305.545) (-23282.408) -- 0:42:52
928500 -- [-23234.982] (-23286.622) (-23267.393) (-23302.992) * (-23273.444) [-23246.511] (-23311.778) (-23268.597) -- 0:42:34
929000 -- (-23235.894) [-23260.145] (-23263.584) (-23301.458) * (-23278.502) (-23263.882) (-23326.282) [-23271.192] -- 0:42:16
929500 -- (-23231.882) [-23251.954] (-23253.724) (-23294.288) * (-23275.722) [-23244.285] (-23313.074) (-23290.457) -- 0:41:58
930000 -- (-23241.072) [-23239.273] (-23269.648) (-23282.391) * (-23252.236) [-23253.916] (-23298.529) (-23278.034) -- 0:41:40

Average standard deviation of split frequencies: 0.015808

930500 -- (-23255.417) [-23258.595] (-23311.371) (-23273.299) * [-23275.284] (-23252.501) (-23292.514) (-23267.655) -- 0:41:22
931000 -- [-23251.902] (-23264.599) (-23334.958) (-23285.528) * (-23251.977) [-23245.431] (-23276.532) (-23288.046) -- 0:41:04
931500 -- [-23254.652] (-23276.111) (-23309.106) (-23295.445) * (-23249.947) [-23248.077] (-23274.542) (-23302.645) -- 0:40:47
932000 -- [-23242.435] (-23260.778) (-23285.639) (-23303.304) * (-23250.609) [-23238.978] (-23267.604) (-23314.701) -- 0:40:29
932500 -- [-23243.565] (-23265.733) (-23288.940) (-23353.036) * [-23242.940] (-23243.609) (-23270.317) (-23314.851) -- 0:40:11
933000 -- [-23241.399] (-23273.283) (-23278.002) (-23336.042) * (-23260.728) [-23240.747] (-23289.383) (-23319.870) -- 0:39:53
933500 -- [-23232.154] (-23272.359) (-23295.766) (-23317.726) * (-23273.631) [-23239.011] (-23276.494) (-23312.094) -- 0:39:35
934000 -- [-23241.718] (-23286.202) (-23274.079) (-23312.993) * (-23265.705) [-23232.917] (-23265.896) (-23294.292) -- 0:39:17
934500 -- [-23241.169] (-23284.091) (-23279.466) (-23320.619) * (-23274.670) (-23260.635) [-23259.739] (-23289.159) -- 0:38:59
```

935000 -- [-23239.350] (-23274.089) (-23273.322) (-23312.805) * (-23283.438) (-23262.424) [-23251.647] (-23288.791) -- 0:38:41

Average standard deviation of split frequencies: 0.015835

935500 -- (-23251.624) [-23272.821] (-23262.113) (-23320.135) * (-23296.334) (-23266.424) [-23246.342] (-23275.398) -- 0:38:24
936000 -- [-23240.119] (-23270.052) (-23277.525) (-23316.894) * (-23312.498) (-23272.882) [-23248.298] (-23285.682) -- 0:38:06
936500 -- (-23270.652) [-23260.742] (-23271.945) (-23311.166) * (-23299.671) (-23267.078) [-23256.117] (-23287.836) -- 0:37:48
937000 -- (-23277.595) [-23252.221] (-23278.125) (-23310.730) * (-23300.392) (-23284.169) [-23260.438] (-23285.462) -- 0:37:30
937500 -- [-23271.623] (-23268.826) (-23289.617) (-23318.388) * (-23304.427) (-23293.766) [-23251.991] (-23285.963) -- 0:37:12
938000 -- (-23282.307) [-23283.863] (-23291.571) (-23343.588) * (-23303.203) (-23287.216) [-23248.056] (-23296.570) -- 0:36:54
938500 -- (-23289.661) [-23281.648] (-23313.253) (-23336.807) * (-23310.164) (-23275.991) [-23262.082] (-23323.917) -- 0:36:36
939000 -- (-23290.081) [-23289.226] (-23304.765) (-23344.353) * (-23302.240) (-23287.215) [-23256.574] (-23348.049) -- 0:36:18
939500 -- (-23264.733) (-23300.901) [-23292.209] (-23332.237) * (-23314.254) (-23277.762) [-23256.037] (-23352.041) -- 0:36:01
940000 -- [-23263.750] (-23281.014) (-23310.825) (-23321.034) * (-23299.642) (-23252.044) [-23247.254] (-23352.193) -- 0:35:43

Average standard deviation of split frequencies: 0.015514

940500 -- [-23265.324] (-23285.149) (-23316.485) (-23312.835) * (-23303.721) (-23269.984) [-23267.311] (-23330.850) -- 0:35:25
941000 -- [-23261.869] (-23275.191) (-23302.085) (-23344.633) * (-23284.805) (-23288.218) [-23256.417] (-23335.203) -- 0:35:07
941500 -- (-23248.894) [-23258.861] (-23297.875) (-23341.297) * (-23271.896) (-23261.269) [-23266.075] (-23328.456) -- 0:34:49
942000 -- [-23230.004] (-23263.660) (-23287.931) (-23338.263) * (-23254.783) (-23275.117) [-23272.716] (-23329.087) -- 0:34:31
942500 -- [-23235.842] (-23282.878) (-23286.838) (-23340.038) * [-23254.161] (-23287.738) (-23286.786) (-23322.953) -- 0:34:13
943000 -- (-23251.205) [-23274.334] (-23281.272) (-23340.052) * [-23256.180] (-23299.800) (-23284.881) (-23319.518) -- 0:33:56
943500 -- (-23262.767) [-23253.415] (-23281.635) (-23318.525) * [-23259.587] (-23299.430) (-23282.123) (-23332.047) -- 0:33:38
944000 -- (-23287.415) [-23271.218] (-23280.983) (-23335.415) * [-23248.279] (-23293.650) (-23283.832) (-23338.191) -- 0:33:20
944500 -- (-23292.715) (-23276.781) [-23276.573] (-23330.950) * [-23249.823] (-23304.460) (-23285.610) (-23303.476) -- 0:33:02
945000 -- (-23287.350) [-23262.064] (-23277.308) (-23318.447) * [-23237.544] (-23285.945) (-23263.353) (-23294.781) -- 0:32:44

Average standard deviation of split frequencies: 0.015289

945500 -- (-23304.286) [-23261.664] (-23263.667) (-23304.495) * [-23239.287] (-23304.382) (-23265.030) (-23279.132) -- 0:32:26
946000 -- (-23297.499) [-23253.480] (-23273.128) (-23286.976) * (-23244.844) (-23308.134) [-23268.957] (-23290.545) -- 0:32:08
946500 -- (-23306.533) [-23256.458] (-23260.166) (-23290.304) * (-23272.726) (-23300.222) (-23275.046) [-23277.066] -- 0:31:50
947000 -- (-23303.288) [-23247.648] (-23242.938) (-23296.827) * [-23252.870] (-23314.456) (-23252.808) (-23301.866) -- 0:31:33
947500 -- (-23299.991) (-23245.003) [-23240.544] (-23290.039) * (-23259.743) (-23303.318) [-23240.899] (-23297.566) -- 0:31:15
948000 -- (-23302.900) [-23249.047] (-23251.063) (-23295.163) * (-23255.755) (-23292.511) [-23246.922] (-23308.161) -- 0:30:57
948500 -- (-23304.637) [-23245.913] (-23256.219) (-23296.447) * (-23263.776) (-23273.726) [-23224.911] (-23307.299) -- 0:30:39
949000 -- (-23293.284) [-23240.659] (-23258.503) (-23296.220) * (-23277.017) (-23272.296) [-23236.772] (-23311.667) -- 0:30:21
949500 -- (-23305.226) (-23258.008) [-23243.103] (-23327.129) * (-23269.990) (-23262.758) [-23222.139] (-23315.420) -- 0:30:03
950000 -- (-23288.909) [-23253.809] (-23242.836) (-23304.132) * (-23265.204) (-23261.925) [-23231.682] (-23311.607) -- 0:29:45

Average standard deviation of split frequencies: 0.014889

950500 -- (-23279.815) [-23255.770] (-23264.933) (-23316.210) * (-23279.121) (-23261.221) [-23233.172] (-23317.708) -- 0:29:27
951000 -- (-23278.025) [-23244.726] (-23281.017) (-23323.677) * (-23276.893) (-23255.268) [-23263.770] (-23312.752) -- 0:29:09
951500 -- (-23278.419) [-23240.938] (-23273.496) (-23319.357) * (-23281.186) (-23257.803) [-23257.196] (-23320.560) -- 0:28:51
952000 -- (-23276.630) [-23250.600] (-23281.954) (-23319.675) * (-23273.234) [-23252.121] (-23260.096) (-23314.480) -- 0:28:33
952500 -- (-23283.162) [-23247.587] (-23260.842) (-23320.154) * (-23262.317) [-23225.361] (-23260.003) (-23290.732) -- 0:28:16
953000 -- (-23260.694) [-23254.789] (-23256.178) (-23328.997) * (-23284.660) [-23239.037] (-23250.767) (-23287.964) -- 0:27:58
953500 -- [-23243.882] (-23246.889) (-23277.433) (-23330.289) * (-23274.013) [-23255.192] (-23249.773) (-23308.700) -- 0:27:40
954000 -- (-23260.590) [-23233.550] (-23284.121) (-23298.896) * (-23291.876) [-23262.313] (-23250.960) (-23322.862) -- 0:27:22
954500 -- (-23260.230) [-23241.719] (-23271.720) (-23297.879) * (-23284.955) (-23257.825) [-23244.266] (-23316.059) -- 0:27:04
955000 -- (-23266.469) [-23259.702] (-23284.191) (-23309.982) * (-23306.674) (-23271.805) [-23245.801] (-23306.829) -- 0:26:46

Average standard deviation of split frequencies: 0.014515

955500 -- [-23263.541] (-23254.917) (-23281.340) (-23292.987) * (-23296.060) (-23269.433) [-23240.154] (-23308.689) -- 0:26:28
956000 -- (-23275.003) [-23254.574] (-23287.151) (-23312.849) * (-23299.056) (-23271.318) [-23239.487] (-23304.059) -- 0:26:10
956500 -- (-23282.671) [-23247.246] (-23263.567) (-23309.962) * (-23314.570) (-23263.366) [-23240.201] (-23294.484) -- 0:25:52
957000 -- (-23278.037) [-23246.393] (-23256.717) (-23318.162) * (-23310.460) (-23282.442) [-23260.182] (-23282.117) -- 0:25:35
957500 -- (-23283.220) [-23259.351] (-23272.108) (-23348.022) * (-23300.517) (-23270.172) [-23259.555] (-23297.036) -- 0:25:17
958000 -- (-23276.240) (-23237.602) [-23257.825] (-23332.096) * (-23296.443) [-23259.913] (-23264.301) (-23289.738) -- 0:24:59
958500 -- (-23277.701) [-23235.288] (-23272.294) (-23317.073) * (-23309.170) [-23261.818] (-23254.512) (-23312.257) -- 0:24:41
959000 -- (-23296.045) [-23239.571] (-23288.046) (-23306.947) * (-23299.882) (-23263.616) [-23257.985] (-23331.666) -- 0:24:23
959500 -- (-23318.122) [-23222.023] (-23283.136) (-23306.808) * (-23305.245) [-23256.292] (-23250.243) (-23328.376) -- 0:24:05
960000 -- (-23315.987) [-23224.672] (-23271.883) (-23299.864) * (-23269.716) (-23253.171) [-23254.861] (-23308.459) -- 0:23:47

Average standard deviation of split frequencies: 0.014359

960500 -- (-23303.823) [-23223.674] (-23266.424) (-23309.996) * (-23265.799) (-23255.991) [-23253.792] (-23320.722) -- 0:23:30
961000 -- (-23276.117) [-23217.788] (-23272.341) (-23317.602) * (-23269.824) (-23254.948) [-23247.914] (-23329.168) -- 0:23:12
961500 -- (-23285.688) [-23232.804] (-23274.827) (-23310.316) * (-23241.613) [-23244.974] (-23270.320) (-23313.624) -- 0:22:54
962000 -- (-23267.788) [-23228.959] (-23277.467) (-23299.382) * (-23240.730) [-23252.568] (-23277.102) (-23307.679) -- 0:22:36
962500 -- (-23279.172) [-23227.804] (-23300.518) (-23308.912) * [-23242.339] (-23262.712) (-23260.254) (-23309.664) -- 0:22:18
963000 -- (-23271.247) [-23228.712] (-23301.214) (-23300.122) * (-23248.177) [-23269.244] (-23265.347) (-23290.958) -- 0:22:00
963500 -- (-23254.039) [-23222.536] (-23292.819) (-23320.585) * (-23258.842) [-23266.160] (-23274.717) (-23305.327) -- 0:21:42
964000 -- (-23257.823) [-23204.790] (-23294.235) (-23310.151) * (-23259.336) [-23254.636] (-23293.497) (-23299.349) -- 0:21:24
964500 -- (-23253.081) [-23218.376] (-23280.857) (-23320.390) * (-23269.925) [-23255.475] (-23266.735) (-23309.233) -- 0:21:07
965000 -- (-23248.917) [-23225.900] (-23285.141) (-23313.503) * (-23281.716) [-23262.903] (-23277.130) (-23317.074) -- 0:20:49

Average standard deviation of split frequencies: 0.014325

965500 -- (-23265.406) [-23221.984] (-23292.421) (-23298.875) * (-23273.626) (-23253.560) [-23262.392] (-23309.270) -- 0:20:31
966000 -- (-23268.505) [-23225.313] (-23299.833) (-23306.096) * (-23289.297) [-23226.431] (-23276.193) (-23325.210) -- 0:20:13
966500 -- (-23271.077) [-23254.546] (-23294.549) (-23292.616) * (-23267.550) [-23241.631] (-23277.778) (-23339.957) -- 0:19:55
967000 -- (-23292.905) [-23268.601] (-23274.604) (-23292.845) * (-23293.079) [-23237.892] (-23278.919) (-23336.420) -- 0:19:37
967500 -- (-23282.613) [-23251.452] (-23258.099) (-23294.449) * (-23278.817) [-23230.966] (-23275.885) (-23323.846) -- 0:19:20
968000 -- (-23281.238) [-23241.802] (-23251.413) (-23306.344) * (-23257.320) [-23228.663] (-23278.377) (-23331.659) -- 0:19:02
968500 -- (-23287.564) [-23254.923] (-23257.170) (-23303.908) * (-23269.767) [-23243.575] (-23301.795) (-23323.657) -- 0:18:44
969000 -- (-23308.103) (-23272.355) [-23261.691] (-23285.146) * (-23260.028) [-23245.497] (-23315.711) (-23302.269) -- 0:18:26
969500 -- (-23320.779) (-23283.063) [-23240.980] (-23275.564) * [-23246.715] (-23252.878) (-23316.622) (-23287.440) -- 0:18:08
970000 -- (-23300.654) (-23264.998) [-23244.960] (-23283.320) * [-23236.485] (-23267.675) (-23309.530) (-23292.756) -- 0:17:50

Average standard deviation of split frequencies: 0.014405

970500 -- (-23313.387) (-23266.192) [-23229.388] (-23283.556) * [-23242.069] (-23261.576) (-23318.378) (-23296.026) -- 0:17:33
971000 -- (-23312.187) (-23268.292) [-23213.605] (-23251.521) * [-23239.337] (-23257.143) (-23333.154) (-23308.401) -- 0:17:15
971500 -- (-23298.915) (-23278.562) [-23216.784] (-23256.972) * [-23233.738] (-23257.858) (-23315.537) (-23300.095) -- 0:16:57
972000 -- (-23310.166) (-23287.268) [-23217.872] (-23255.531) * [-23232.565] (-23264.678) (-23321.144) (-23296.087) -- 0:16:39
972500 -- (-23307.015) (-23267.470) [-23226.679] (-23237.277) * [-23216.128] (-23268.006) (-23313.850) (-23256.643) -- 0:16:21
973000 -- (-23307.419) (-23275.470) [-23226.525] (-23250.067) * [-23230.822] (-23255.589) (-23313.205) (-23248.533) -- 0:16:03
973500 -- (-23329.327) (-23264.517) [-23247.681] (-23249.686) * [-23226.311] (-23258.861) (-23309.877) (-23249.022) -- 0:15:46
974000 -- (-23338.653) (-23271.036) [-23255.429] (-23265.401) * [-23238.860] (-23279.474) (-23302.871) (-23249.438) -- 0:15:28
974500 -- (-23337.551) (-23269.881) [-23242.608] (-23265.650) * [-23249.175] (-23269.336) (-23292.231) (-23266.012) -- 0:15:10
975000 -- (-23326.176) (-23258.616) [-23261.257] (-23265.497) * [-23245.325] (-23265.231) (-23305.777) (-23263.849) -- 0:14:52

Average standard deviation of split frequencies: 0.014421

```
   975500 -- (-23342.443) (-23266.158) (-23253.446) [-23251.916] * [-23248.724] (-23269.798) (-23290.442) (-23292.938) -- 0:14:34
   976000 -- (-23330.470) (-23252.229) (-23254.290) [-23238.363] * [-23247.643] (-23282.451) (-23307.182) (-23269.702) -- 0:14:16
   976500 -- (-23322.767) (-23272.512) [-23242.199] (-23264.684) * [-23266.215] (-23283.779) (-23296.018) (-23236.244) -- 0:13:59
   977000 -- (-23317.910) (-23285.276) (-23250.128) [-23256.483] * (-23261.957) (-23284.988) (-23290.893) [-23243.609] -- 0:13:41
   977500 -- (-23327.766) (-23278.872) (-23248.430) [-23240.447] * (-23256.910) (-23285.060) (-23289.947) [-23245.885] -- 0:13:23
   978000 -- (-23340.644) (-23286.551) (-23242.370) [-23257.244] * [-23248.648] (-23270.099) (-23280.266) [-23239.644] -- 0:13:05
   978500 -- (-23345.811) (-23280.938) (-23244.405) [-23258.527] * [-23252.654] (-23266.874) (-23278.058) (-23242.643) -- 0:12:47
   979000 -- (-23315.428) (-23272.697) [-23238.581] (-23263.455) * [-23251.531] (-23255.702) (-23294.927) (-23249.562) -- 0:12:29
   979500 -- (-23317.848) (-23259.032) (-23252.557) [-23256.893] * [-23253.781] (-23261.099) (-23279.215) (-23260.412) -- 0:12:11
   980000 -- (-23312.044) (-23254.268) (-23259.432) [-23263.601] * (-23267.975) (-23270.424) (-23268.967) [-23245.360] -- 0:11:54

   Average standard deviation of split frequencies: 0.014426

   980500 -- (-23324.802) [-23251.142] (-23272.462) (-23271.346) * [-23260.284] (-23301.224) (-23264.062) (-23250.126) -- 0:11:36
   981000 -- (-23320.131) [-23246.564] (-23260.464] (-23276.853) * [-23239.750] (-23317.709) (-23269.338) (-23239.322) -- 0:11:18
   981500 -- (-23328.430) [-23258.741] (-23255.031) (-23291.661) * [-23227.488] (-23322.482) (-23281.183) (-23241.229) -- 0:11:00
   982000 -- (-23350.924) (-23276.050) [-23256.616] (-23297.927) * [-23225.864] (-23318.155) (-23282.151) (-23260.788) -- 0:10:42
   982500 -- (-23354.744) (-23265.880) [-23264.790] (-23274.243) * (-23241.477) (-23299.063) (-23284.768) [-23256.930] -- 0:10:24
   983000 -- (-23351.197) [-23250.914] (-23268.871) (-23290.837) * (-23240.220) (-23295.994) (-23270.608) [-23255.794] -- 0:10:07
   983500 -- (-23335.003) [-23236.600] (-23279.189) (-23287.242) * (-23232.357) (-23291.252) (-23269.805) [-23256.574] -- 0:09:49
   984000 -- (-23328.637) [-23244.617] (-23269.500) (-23297.576) * (-23224.195] (-23292.295) (-23282.423) (-23259.219) -- 0:09:31
   984500 -- (-23315.056) [-23259.335] (-23258.878) (-23298.554) * [-23236.031] (-23287.048) (-23294.479) (-23274.139) -- 0:09:13
   985000 -- (-23309.244) [-23244.358] (-23245.823) (-23296.875) * [-23237.673] (-23274.123) (-23296.282) (-23285.580) -- 0:08:55

   Average standard deviation of split frequencies: 0.014269

   985500 -- (-23319.580) (-23240.074) [-23241.143] (-23296.738) * [-23244.907] (-23271.801) (-23298.917) (-23290.318) -- 0:08:37
   986000 -- (-23349.605) (-23271.203) [-23245.971] (-23295.096) * [-23275.371] (-23264.735) (-23293.369) (-23293.242) -- 0:08:19
   986500 -- (-23362.682) (-23266.015) [-23252.734] (-23280.158) * [-23264.740] (-23259.493) (-23296.524) (-23280.241) -- 0:08:02
   987000 -- (-23349.664) (-23270.735) (-23249.877) [-23270.127] * (-23264.261) (-23272.469) (-23300.229) (-23260.419) -- 0:07:44
   987500 -- (-23353.251) (-23271.539) (-23250.978) [-23252.761] * [-23260.881] (-23271.997) (-23317.774) (-23256.937) -- 0:07:26
   988000 -- (-23344.651) (-23280.274) [-23243.903] (-23275.032) * (-23285.791) [-23262.518] (-23314.030) (-23263.761) -- 0:07:08
   988500 -- (-23325.967) (-23279.289) [-23254.822] (-23272.672) * (-23276.232) [-23267.649] (-23303.211) (-23251.304) -- 0:06:50
   989000 -- (-23341.474) (-23273.754) (-23255.506) [-23266.317] * (-23254.780) (-23284.064) (-23308.178) [-23247.371] -- 0:06:32
   989500 -- (-23335.303) (-23284.590) (-23244.200) [-23241.871] * [-23237.859] (-23304.787) (-23275.914) (-23239.852) -- 0:06:14
   990000 -- (-23320.049) (-23282.902) [-23223.797] (-23225.935) * [-23257.995] (-23317.885) (-23283.295) (-23271.260) -- 0:05:57

   Average standard deviation of split frequencies: 0.014083

   990500 -- (-23325.264) (-23275.108) [-23231.374] (-23258.847) * [-23257.144] (-23323.614) (-23291.328) (-23256.540) -- 0:05:39
   991000 -- (-23306.104) (-23293.967) [-23231.590] (-23255.435) * (-23254.323) (-23317.957) (-23293.031) [-23254.067] -- 0:05:21
   991500 -- (-23288.576) (-23304.964) [-23237.026] (-23265.343) * [-23256.011] (-23310.437) (-23294.974) [-23258.229] -- 0:05:03
   992000 -- (-23304.487) (-23277.749) [-23228.157] (-23264.201) * [-23249.415] (-23289.655) (-23295.243) (-23261.407) -- 0:04:45
   992500 -- (-23298.904) (-23267.504) [-23227.309] (-23278.022) * (-23254.356) (-23313.572) (-23294.399) [-23268.131] -- 0:04:27
   993000 -- (-23291.028) (-23279.032) [-23219.027] (-23283.336) * [-23248.816] (-23322.321) (-23297.498) (-23274.845) -- 0:04:09
   993500 -- (-23275.627) (-23276.628) [-23262.782] (-23284.157) * [-23240.511] (-23312.644) (-23301.344) (-23276.236) -- 0:03:52
   994000 -- (-23297.854) (-23261.817) [-23233.949] (-23280.106) * [-23220.351] (-23286.348) (-23302.923) (-23272.856) -- 0:03:34
   994500 -- (-23315.221) (-23258.357) [-23233.368] (-23284.666) * [-23210.871] (-23289.195) (-23293.268) (-23255.800) -- 0:03:16
   995000 -- (-23309.484) [-23263.302] (-23235.555) (-23295.904) * [-23220.325] (-23283.432) (-23289.651) (-23269.267) -- 0:02:58

   Average standard deviation of split frequencies: 0.013975

   995500 -- (-23313.149) (-23275.394) [-23236.330] (-23290.623) * [-23222.253] (-23288.873) (-23317.687) (-23264.911) -- 0:02:40
   996000 -- (-23312.767) (-23275.824) [-23243.058] (-23333.910) * [-23241.488] (-23316.086) (-23305.000) (-23267.091) -- 0:02:22
   996500 -- (-23305.131) [-23274.571] (-23265.961) (-23326.877) * (-23246.608) (-23305.317) (-23296.698) [-23278.422] -- 0:02:04
   997000 -- (-23300.533) [-23248.724] (-23279.401) (-23311.796) * [-23242.016] (-23308.661) (-23312.369) (-23267.051) -- 0:01:47
   997500 -- (-23289.379) (-23264.517) [-23248.535] (-23313.272) * [-23231.236] (-23307.655) (-23299.056) (-23250.530) -- 0:01:29
   998000 -- (-23290.863) (-23269.890) [-23241.940] (-23329.976) * [-23231.999] (-23300.142) (-23285.294) (-23266.153) -- 0:01:11
   998500 -- (-23272.847) [-23263.854] (-23235.366) (-23315.233) * [-23229.961] (-23293.933) (-23290.052) (-23274.127) -- 0:00:53
   999000 -- (-23293.243) (-23252.630) [-23239.228] (-23315.671) * [-23226.124] (-23296.839) (-23271.843) (-23268.602) -- 0:00:35
   999500 -- (-23304.581) [-23239.779] (-23243.926) (-23297.370) * [-23240.159] (-23277.292) (-23268.596) (-23293.720) -- 0:00:17
   1000000 -- (-23330.264) [-23251.354] (-23230.773) (-23294.003) * [-23234.609] (-23265.566) (-23271.204) (-23313.849) -- 0:00:00

   Average standard deviation of split frequencies: 0.014065
   Continue with analysis? (yes/no):

RECEIVED CMD:  no


   Analysis completed in 9 hours 55 mins 1 seconds
   Analysis used 35616.63 seconds of CPU time
   Likelihood of best state for "cold" chain of run 1 was -23191.65
   Likelihood of best state for "cold" chain of run 2 was -23198.89

   Acceptance rates for the moves in the "cold" chain of run 1:
      With prob.   (last 100)   chain accepted proposals by move
         19.2 %     ( 24 %)     ExtSPR(Tau,V)
          6.6 %     (  1 %)     ExtTBR(Tau,V)
         22.8 %     ( 19 %)     NNI(Tau,V)
         19.8 %     ( 18 %)     ParsSPR(Tau,V)
         26.5 %     ( 24 %)     Multiplier(V)
         44.3 %     ( 41 %)     Nodeslider(V)

   Acceptance rates for the moves in the "cold" chain of run 2:
      With prob.   (last 100)   chain accepted proposals by move
         19.0 %     ( 11 %)     ExtSPR(Tau,V)
          6.6 %     ( 11 %)     ExtTBR(Tau,V)
         22.8 %     ( 23 %)     NNI(Tau,V)
         19.6 %     ( 20 %)     ParsSPR(Tau,V)
         26.6 %     ( 30 %)     Multiplier(V)
         44.1 %     ( 44 %)     Nodeslider(V)

   Chain swap information for run 1:

                1       2       3       4
        ----------------------------------
      1 |            0.29    0.04    0.00
```

```
      2 |  166416            0.28    0.05
      3 |  166925  167041            0.33
      4 |  167046  166207  166365

   Chain swap information for run 2:

                1       2       3       4
        --------------------------------
      1 |            0.29    0.04    0.00
      2 |  166331            0.33    0.04
      3 |  166435  166874            0.26
      4 |  166345  166567  167448

   Upper diagonal: Proportion of successful state exchanges between chains
   Lower diagonal: Number of attempted state exchanges between chains

   Chain information:

     ID -- Heat
    -----------
      1 -- 1.00  (cold chain)
      2 -- 0.91
      3 -- 0.83
      4 -- 0.77

   Heat = 1 / (1 + T * (ID - 1))
      (where T = 0.10 is the temperature and ID is the chain number)

MrBayes >

RECEIVED CMD:  sumt


   Summarizing trees in files "/infile_converted.phy.run1.t" and "/infile_converted.phy.run2.t"
   Using relative burnin ('relburnin=yes'), discarding the first 25 % of sampled trees
   Writing statistics to files /infile_converted.phy.parts|tstat|vstat|trprobs|con>
   Examining first file ...
   Found one tree block in file "/infile_converted.phy.run1.t" with 2001 trees in last block
   Expecting the same number of trees in the last tree block of all files

   Tree reading status:

   0      10      20      30      40      50      60      70      80      90     100
   v-------v-------v-------v-------v-------v-------v-------v-------v-------v-------v
   *********
*********
*********
********
*****
**
************
*********
********
*********
*

   Read a total of 4002 trees in 2 files (sampling 3002 of them)
      (Each file contained 2001 trees of which 1501 were sampled)

   General explanation:

   In an unrooted tree, a taxon bipartition (split) is specified by removing a
   branch, thereby dividing the species into those to the left and those to the
   right of the branch. Here, taxa to one side of the removed branch are denoted
   '.' and those to the other side are denoted '*'. Specifically, the '.' symbol
   is used for the taxa on the same side as the outgroup.

   In a rooted or clock tree, the tree is rooted using the model and not by
   reference to an outgroup. Each bipartition therefore corresponds to a clade,
   that is, a group that includes all the descendants of a particular branch in
   the tree.  Taxa that are included in each clade are denoted using '*', and
   taxa that are not included are denoted using the '.' symbol.

   The output first includes a key to all the bipartitions with frequency larger
   or equal to (Minpartfreq) in at least one run. Minpartfreq is a paramiter to
   sumt command and currently it is set to 0.10.  This is followed by a table
   with statistics for the informative bipartitions (those including at least
   two taxa), sorted from highest to lowest probability. For each bipartition,
   the table gives the number of times the partition or split was observed in all
   runs (#obs) and the posterior probability of the bipartition (Probab.), which
   is the same as the split frequency. If several runs are summarized, this is
```

followed by the minimum split frequency (Min(s)), the maximum frequency (Max(s)), and the standard deviation of frequencies (Stddev(s)) across runs. The latter value should approach 0 for all bipartitions as MCMC runs converge.

This is followed by a table summarizing branch lengths, node heights (if a clock model was used) and relaxed clock parameters (if a relaxed clock model was used). The mean, variance, and 95 % credible interval are given for each of these parameters. If several runs are summarized, the potential scale reduction factor (PSRF) is also given; it should approach 1 as runs converge. Node heights will take calibration points into account, if such points were used in the analysis.

Note that Stddev may be unreliable if the partition is not present in all runs (the last column indicates the number of runs that sampled the partition if more than one run is summarized). The PSRF is not calculated at all if the partition is not present in all runs.The PSRF is also sensitive to small sample sizes and it should only be considered a rough guide to convergence since some of the assumptions allowing one to interpret it as a true potential scale reduction factor are violated in MrBayes.

List of taxa in bipartitions:

```
   1 -- 139_vCCI_V
   2 -- 140_vCCI_V
   3 -- 129_vCCI_V
   4 -- 130_vCCI_V
   5 -- 155_vCCI_V
   6 -- 156_vCCI_V
   7 -- 143_vCCI_C
   8 -- 144_vCCI_C
   9 -- 133_vCCI_C
  10 -- 141_vCCI_C
  11 -- 134_vCCI_V
  12 -- 135_vCCI_V
  13 -- 142_vCCI_R
  14 -- 136_vCCI_V
  15 -- 131_vCCI_E
  16 -- 132_vCCI_E
  17 -- 149_vCCI_C
  18 -- 152_vCCI_C
  19 -- 153_vCCI_C
  20 -- 154_vCCI_C
  21 -- 137_vCCI_H
  22 -- 138_vCCI_H
  23 -- 150_vCCI_M
  24 -- 151_vCCI_M
  25 -- 147_vCCI_M
  26 -- 148_vCCI_M
  27 -- 145_vCCI_M
  28 -- 146_vCCI_M
  29 -- 3_A41like_
  30 -- 4_A41like_
  31 -- 5_A41_MPXV
  32 -- 6_A41_MPXV
  33 -- 17_A41_CPX
  34 -- 18_A41_CPX
  35 -- 7_A41_VARV
  36 -- 10_A41_VAR
  37 -- 11_A41_VAR
  38 -- 12_A41_VAR
  39 -- 9_A41_VARV
  40 -- 8_A41_VARV
  41 -- 13_A41_TAT
  42 -- 16_A41_CPX
  43 -- 15_A41_HSP
  44 -- 20_A41_VAC
  45 -- 14_A41_CML
  46 -- 19_A41_ECT
  47 -- 80_GIF_ORF
  48 -- 82_GIF_PCP
  49 -- 21_BPSVgOR
  50 -- 81_GIF_SB8
  51 -- 75_CBP_ORF
  52 -- 79_CPB_PCP
  53 -- 76_CBP_BPS
  54 -- 77_CPB_SB8
  55 -- 78_CPB_SB8
  56 -- 109_T4like
  57 -- 112_T4like
  58 -- 110_T4like
  59 -- 111_T4like
```

60 -- 113_T4like
61 -- 114_T4like
62 -- 115_T4like
63 -- 116_T4like
64 -- 117_T4_MYX
65 -- 118_T4_MYX
66 -- 119_T4_DPX
67 -- 120_T4_DPX
68 -- 121_T4_LSD
69 -- 122_T4_LSD
70 -- 123_T4_SPP
71 -- 124_T4_SPP
72 -- 125_T4_GTP
73 -- 126_T4_GTP
74 -- 127_T4_GTP
75 -- 128_T4_GTP
76 -- 24_C8_VACV
77 -- 25_C8_HSPV
78 -- 22_C8_CPXV
79 -- 23_C8_CMLV
80 -- 26_C8_CPXV
81 -- 27_C8_CPXV
82 -- 98_SCP3_MP
83 -- 99_SCP3_MP
84 -- 101_SCP3_H
85 -- 106_SCP3_V
86 -- 102_SCP3_C
87 -- 105_SCP3_E
88 -- 100_SCP3_T
89 -- 104_SCP3_C
90 -- 103_SCP3_C
91 -- 84_SCP1_MP
92 -- 85_SCP1_MP
93 -- 90_SCP1_CP
94 -- 87_SCP1_VA
95 -- 86_SCP1_CP
96 -- 91_SCP1_HS
97 -- 88_SCP1_CP
98 -- 89_SCP1_TA
99 -- 92_SCP1_CM
100 -- 107_SCPlik
101 -- 108_SCPlik
102 -- 30_CrmB_MP
103 -- 31_CrmB_MP
104 -- 32_CrmB_MP
105 -- 33_CrmB_MP
106 -- 40_CrmB_CM
107 -- 41_CrmB_CM
108 -- 44_CrmB_CP
109 -- 45_CrmB_CP
110 -- 42_CrmB_HS
111 -- 43_CrmB_HS
112 -- 48_CrmB_CP
113 -- 49_CrmB_CP
114 -- 36_CrmB_VA
115 -- 38_CrmB_VA
116 -- 34_CrmB_VA
117 -- 37_CrmB_VA
118 -- 35_CrmB_VA
119 -- 39_CrmB_VA
120 -- 46_CrmB_CP
121 -- 47_CrmB_CP
122 -- 52_CrmD_CP
123 -- 53_CrmD_CP
124 -- 54_CrmD_EC
125 -- 55_CrmD_EC
126 -- 93_SCP2_CP
127 -- 95_SCP2_CP
128 -- 96_SCP2_CP
129 -- 94_SCP2_EC
130 -- 50_CrmB_MY
131 -- 51_CrmB_MY
132 -- 97_SCP2_CO
133 -- 1_M2_CPXV_
134 -- 2_M2_CPXV_
135 -- 61_M2_VARV
136 -- 64_M2_VARV
137 -- 63_M2_VARV
138 -- 71_M2_VARV
139 -- 69_M2_VARV
140 -- 70_M2_VARV

```
 141 -- 65_M2_TATV
 142 -- 62_M2_CMLV
 143 -- 66_M2_MPXV
 144 -- 67_M2_MPXV
 145 -- 68_M2_HSPV
 146 -- 72_M2_VACV
 147 -- 60_M2_COTV
 148 -- 73_M2_YKV_
 149 -- 74_M2_MYXV
 150 -- 58_M2like_
 151 -- 59_M2like_
 152 -- 83_SB87_gp
 153 -- 29_CPXV_GE
 154 -- 57_M2like_
 155 -- 28_COTV030
 156 -- 56_DPXV_W8

Key to taxon bipartitions (saved to file "/infile_converted.phy.parts"):

 ID -- Partition
--------------------------------------------------------------------------------
  1 -- .**********************************************************************
  2 -- .*.....................................................................
  3 -- ..*....................................................................
  4 -- ...*...................................................................
  5 -- ....*..................................................................
  6 -- .....*.................................................................
  7 -- ......*................................................................
  8 -- .......*...............................................................
  9 -- ........*..............................................................
 10 -- .........*.............................................................
 11 -- ..........*............................................................
 12 -- ...........*...........................................................
 13 -- ............*..........................................................
 14 -- .............*.........................................................
 15 -- ..............*........................................................
 16 -- ...............*.......................................................
 17 -- ................*......................................................
 18 -- .................*.....................................................
 19 -- ..................*....................................................
 20 -- ...................*...................................................
 21 -- ....................*..................................................
 22 -- .....................*.................................................
 23 -- ......................*................................................
 24 -- .......................*...............................................
 25 -- ........................*..............................................
 26 -- .........................*.............................................
 27 -- ..........................*............................................
 28 -- ...........................*...........................................
 29 -- ............................*..........................................
 30 -- .............................*.........................................
 31 -- ..............................*........................................
 32 -- ...............................*.......................................
 33 -- ................................*......................................
 34 -- .................................*.....................................
 35 -- ..................................*....................................
 36 -- ...................................*...................................
 37 -- ....................................*..................................
 38 -- .....................................*.................................
 39 -- ......................................*................................
 40 -- .......................................*...............................
 41 -- ........................................*..............................
 42 -- .........................................*.............................
 43 -- ..........................................*............................
 44 -- ...........................................*...........................
 45 -- ............................................*..........................
 46 -- .............................................*.........................
 47 -- ..............................................*........................
 48 -- ...............................................*.......................
 49 -- ................................................*......................
 50 -- .................................................*.....................
 51 -- ..................................................*....................
 52 -- ...................................................*...................
 53 -- ....................................................*..................
 54 -- .....................................................*.................
 55 -- ......................................................*................
 56 -- .......................................................*...............
 57 -- ........................................................*..............
 58 -- .........................................................*.............
 59 -- ..........................................................*............
 60 -- ...........................................................*...........
```

```
 61 -- ............................................................*..........
 62 -- .............................................................*.........
 63 -- ..............................................................*........
 64 -- ...............................................................*.......
 65 -- ................................................................*......
 66 -- .................................................................*.....
 67 -- ..................................................................*....
 68 -- ...................................................................*...
 69 -- ....................................................................*..
 70 -- .....................................................................*.
 71 -- ......................................................................*
 72 -- .......................................................................
 73 -- .......................................................................
 74 -- .......................................................................
 75 -- .......................................................................
 76 -- .......................................................................
 77 -- .......................................................................
 78 -- .......................................................................
 79 -- .......................................................................
 80 -- .......................................................................
 81 -- .......................................................................
 82 -- .......................................................................
 83 -- .......................................................................
 84 -- .......................................................................
 85 -- .......................................................................
 86 -- .......................................................................
 87 -- .......................................................................
 88 -- .......................................................................
 89 -- .......................................................................
 90 -- .......................................................................
 91 -- .......................................................................
 92 -- .......................................................................
 93 -- .......................................................................
 94 -- .......................................................................
 95 -- .......................................................................
 96 -- .......................................................................
 97 -- .......................................................................
 98 -- .......................................................................
 99 -- .......................................................................
100 -- .......................................................................
101 -- .......................................................................
102 -- .......................................................................
103 -- .......................................................................
104 -- .......................................................................
105 -- .......................................................................
106 -- .......................................................................
107 -- .......................................................................
108 -- .......................................................................
109 -- .......................................................................
110 -- .......................................................................
111 -- .......................................................................
112 -- .......................................................................
113 -- .......................................................................
114 -- .......................................................................
115 -- .......................................................................
116 -- .......................................................................
117 -- .......................................................................
118 -- .......................................................................
119 -- .......................................................................
120 -- .......................................................................
121 -- .......................................................................
122 -- .......................................................................
123 -- .......................................................................
124 -- .......................................................................
125 -- .......................................................................
126 -- .......................................................................
127 -- .......................................................................
128 -- .......................................................................
129 -- .......................................................................
130 -- .......................................................................
131 -- .......................................................................
132 -- .......................................................................
133 -- .......................................................................
134 -- .......................................................................
135 -- .......................................................................
136 -- .......................................................................
137 -- .......................................................................
138 -- .......................................................................
139 -- .......................................................................
140 -- .......................................................................
141 -- .......................................................................
```

```
142 -- .......................................................................
143 -- .......................................................................
144 -- .......................................................................
145 -- .......................................................................
146 -- .......................................................................
147 -- .......................................................................
148 -- .......................................................................
149 -- .......................................................................
150 -- .......................................................................
151 -- .......................................................................
152 -- .......................................................................
153 -- .......................................................................
154 -- .......................................................................
155 -- .......................................................................
156 -- .......................................................................
157 -- ....................******.............................................
158 -- .......................................................................
159 -- .......................................................................
160 -- .......................................................................
161 -- ..........................**...........................................
162 -- .......................................................****************
163 -- .......................................................********........
164 -- .............................................................**........
165 -- ................****...................................................
166 -- ..............*********************************************************
167 -- ......................****.............................................
168 -- ..............................................*********................
169 -- ..........................*********************************************
170 -- ....................**.................................................
171 -- .................................................................**....
172 -- ..............................................**.......................
173 -- ............................**.........................................
174 -- ..............................................****.....................
175 -- .......................................................................
176 -- .......................................................................
177 -- .......................................................................
178 -- ......**...............................................................
179 -- ......*****************************************************************
180 -- .......................................................................
181 -- .......................................................................
182 -- ..............................****************.........................
183 -- .......................................................................
184 -- .......................................................................
185 -- .......................................................................
186 -- .......................................................................
187 -- .......................................................................
188 -- ........***************************************************************
189 -- .......................................................................
190 -- .......................................................**..............
191 -- .......................................................................
192 -- .......................................................................
193 -- ...............................................................**......
194 -- .......................................................................
195 -- .......................................................................
196 -- .......................................................................
197 -- ..................**...................................................
198 -- ..................................................**...................
199 -- .....................................................**................
200 -- .......................................................................
201 -- .......................................................................
202 -- ..............**.......................................................
203 -- .......................................................................
204 -- ...**..................................................................
205 -- .......................................................................
206 -- .........................................................****..........
207 -- .......................................................................
208 -- .......................................................................
209 -- .......................................................................
210 -- .......................................................................
211 -- .......................................................................
212 -- ..................................**...................................
213 -- .......................................................................
214 -- .......................................................................
215 -- .......................................................................
216 -- .......................................................................
217 -- ...................................................................****
218 -- .......................................................................
219 -- .......................................................................
220 -- ................................**.....................................
221 -- ..........................................**...........................
222 -- .......................................................................
```

```
223 -- ..............................**.......................................
224 -- .......................................................................
225 -- .........................................................**............
226 -- ................**.....................................................
227 -- .......................................................******..........
228 -- .......................................................................
229 -- ............................******************.........................
230 -- .......................................................................
231 -- ..........**...........................................................
232 -- .......................................................................
233 -- ............................*******************************************
234 -- .......................................................****************
235 -- .......................................................................
236 -- .......................................................................
237 -- .......................................................................
238 -- .......................................................................
239 -- .......................................................................
240 -- .......................................................................
241 -- .......................................................................
242 -- ..............................****.....................................
243 -- .......................................................................
244 -- ..............................................***......................
245 -- .......................................................................
246 -- ..............................****.......***.*.........................
247 -- .......................................................................
248 -- ........**.............................................................
249 -- .....................................................................**
250 -- .......................................................................
251 -- ..................................******...............................
252 -- .......................................................................
253 -- .......................................................................
254 -- .......................................................................
255 -- .......................................................................
256 -- .......................................................................
257 -- .......................................................................
258 -- ..................................................*****................
259 -- .......................................................................
260 -- ........*****.*********************************************************
261 -- .......................................................................
262 -- .........................................................***...........
263 -- .................................................................******
264 -- .......................................................................
265 -- ..............................**************.*.........................
266 -- .......................................................................
267 -- .......................................................................
268 -- ................**********.............................................
269 -- .......................................................................
270 -- ...............................................................********
271 -- .......................................................................
272 -- .......................................................................
273 -- .......................................................................
274 -- .......................................................................
275 -- .......................................................................
276 -- .......................................................................
277 -- ..............................................****.....................
278 -- ..............************.............................................
279 -- ........*****..........................................................
280 -- ..............................****...........*.........................
281 -- ........**....*********************************************************
282 -- .......................................................................
283 -- .......................................................................
284 -- .......................................................................
285 -- ............................******************.........****************
286 -- ..............................................*********................
287 -- .......................................................................
288 -- ..................................................***..................
289 -- .......................................................................
290 -- .......................................................................
291 -- ...................................................................**..
292 -- .......................................................................
293 -- .......................................................................
294 -- ...................................................................****
295 -- .......................................................................
296 -- .......................................................................
297 -- .......................................................................
298 -- .......................................................................
299 -- .......................................................................
300 -- .......................................................................
301 -- .......................................................................
302 -- .......................................................................
303 -- .......................................................................
```

```
304 -- .......................................................................
305 -- .......................................................................
306 -- .......................................................................
307 -- .......................................................................
308 -- ..................................................**.**................
309 -- .......................................................................
310 -- ..........***..........................................................
311 -- ..............................................*************************
312 -- .......................................................................
313 -- ..............................****......****.*.........................
314 -- .......................................................................
315 -- ...................................................................**..
316 -- ................*******************************************************
317 -- .....******************************************************************
318 -- .......................................................................
319 -- ...........................................................**..........
320 -- .......................................................................
321 -- ..................................*******..............................
322 -- .......................................................................
323 -- .........................................***...........................
324 -- .......................................................................
325 -- ..............................****........**.*.........................
326 -- .......................................................................
327 -- .......................................................................
328 -- ..............................****......******.........................
329 -- .......................................................................
330 -- .......................................................................
331 -- .......................................................................
332 -- ..............................................******.**................
333 -- ..............................****.......*...*.........................
334 -- .......................................................................
335 -- .......................................................................
336 -- .......................**..............................................
337 -- .......................................................................
338 -- ......................***..............................................
339 -- ..............................**********.***.*.........................
340 -- ............................***************************................
341 -- ......................**...............................................
342 -- .......................................................................
343 -- ......................*..*.............................................
344 -- .......................................................................
345 -- .......................................................................
346 -- .......................................................................
347 -- .......................................................................
348 -- .......................................................................
349 -- .......................................................**********......
350 -- .......................................................********..******
351 -- ......................**.*.............................................
352 -- .......................................................................
353 -- .......................................................................
354 -- .......................................................................
355 -- .......................................................................
356 -- .......................***.............................................
357 -- .......................................................................
358 -- .......................*.*.............................................
359 -- .......................................................................
360 -- ......................*.*..............................................
361 -- ......................*.**.............................................
362 -- .......................................................................
363 -- .......................................................................
364 -- .......................................................................
365 -- .......................................................................
366 -- .......................................................................
367 -- .......................................................................
368 -- ........................**.............................................
369 -- ...................................................................*...
370 -- .......................................................................
371 -- .......................................................................
372 -- .......................................................********..**....
373 -- .......................................................................
374 -- .......................................................................
375 -- .......................................................................
376 -- .......................................................................
377 -- .......................................................................
378 -- .......................................................................
379 -- ....................................................................*..
380 -- ........****..*********************************************************
381 -- .......................................................................
382 -- ........**..*..........................................................
383 -- ...................................................................*.**
384 -- .......................................................................
```

```
385 -- .............**********************************************************
386 -- .......................................................................
387 -- .......................................................................
388 -- ....................................................................***
389 -- .......................................................................
390 -- ........****...........................................................
391 -- .......................................................................
392 -- .*.**..................................................................
393 -- ........******.........................................................
394 -- ..*********************************************************************
395 -- ........**..*.*********************************************************
396 -- ..............**..........*********************************************
397 -- ..............******...................................................
398 -- .......................................................................
399 -- .......................................................................
400 -- .......................................................................
401 -- ..***..................................................................
402 -- ...............................................................**..****
403 -- ..................................**.***...............................
404 -- .....................................**................................
405 -- ..................................**.*.................................
406 -- .*.********************************************************************
407 -- .**..******************************************************************
408 -- ....................................*..*...............................
409 -- ....................................**.................................
410 -- ......................................**...............................
411 -- ..................................**...*...............................
412 -- .**....................................................................
413 -- ..................................*******...*..........................
414 -- ..................................**..*................................
415 -- ..................................***..................................
416 -- ..............******......*********************************************
417 -- .*...*.................................................................
418 -- ..............................****........**...........................
419 -- .......................................................................
420 -- ..*..*.................................................................
421 -- ..................................*****................................
422 -- .......................................................................
423 -- ..*...*****************************************************************
424 -- ....................................*.*................................
425 -- .****.*****************************************************************
426 -- .*....*****************************************************************
427 -- .....................................*.*...............................
428 -- ..............................................****...**................
429 -- .*****.................................................................
430 -- ................****......*********************************************
431 -- .......................................................................
432 -- ........................................*...*..........................
433 -- ..............................****.......***...........................
434 -- ...***.................................................................
435 -- ....................................****...............................
436 -- ..................................***.**...............................
437 -- ...**.*****************************************************************
438 -- ..*..******************************************************************
439 -- .........................................*...*.........................
440 -- ..................................****.*...............................
441 -- ..............................****.......*.............................
442 -- .........................................***.*.........................
443 -- .......................................................................
444 -- .......................................................................
445 -- .......................................................************....
446 -- .......................................................................
447 -- .........................................................**.*..........
448 -- ...********************************************************************
449 -- ....................***************************************************
450 -- .......................................................................
451 -- .****..................................................................
452 -- .......................................................................
453 -- .....................................................................**
454 -- ....................................................................***
455 -- ....................................................***................
456 -- .......................................................................
457 -- ..................................***********..........................
--------------------------------------------------------------------------------

 ID -- Partition (continued)
--------------------------------------------------------------------------------
  1 -- ***********************************************************************
  2 -- .......................................................................
  3 -- .......................................................................
  4 -- .......................................................................
```

```
 5 -- .......................................................................
 6 -- .......................................................................
 7 -- .......................................................................
 8 -- .......................................................................
 9 -- .......................................................................
10 -- .......................................................................
11 -- .......................................................................
12 -- .......................................................................
13 -- .......................................................................
14 -- .......................................................................
15 -- .......................................................................
16 -- .......................................................................
17 -- .......................................................................
18 -- .......................................................................
19 -- .......................................................................
20 -- .......................................................................
21 -- .......................................................................
22 -- .......................................................................
23 -- .......................................................................
24 -- .......................................................................
25 -- .......................................................................
26 -- .......................................................................
27 -- .......................................................................
28 -- .......................................................................
29 -- .......................................................................
30 -- .......................................................................
31 -- .......................................................................
32 -- .......................................................................
33 -- .......................................................................
34 -- .......................................................................
35 -- .......................................................................
36 -- .......................................................................
37 -- .......................................................................
38 -- .......................................................................
39 -- .......................................................................
40 -- .......................................................................
41 -- .......................................................................
42 -- .......................................................................
43 -- .......................................................................
44 -- .......................................................................
45 -- .......................................................................
46 -- .......................................................................
47 -- .......................................................................
48 -- .......................................................................
49 -- .......................................................................
50 -- .......................................................................
51 -- .......................................................................
52 -- .......................................................................
53 -- .......................................................................
54 -- .......................................................................
55 -- .......................................................................
56 -- .......................................................................
57 -- .......................................................................
58 -- .......................................................................
59 -- .......................................................................
60 -- .......................................................................
61 -- .......................................................................
62 -- .......................................................................
63 -- .......................................................................
64 -- .......................................................................
65 -- .......................................................................
66 -- .......................................................................
67 -- .......................................................................
68 -- .......................................................................
69 -- .......................................................................
70 -- .......................................................................
71 -- .......................................................................
72 -- *......................................................................
73 -- .*.....................................................................
74 -- ..*....................................................................
75 -- ...*...................................................................
76 -- ....*..................................................................
77 -- .....*.................................................................
78 -- ......*................................................................
79 -- .......*...............................................................
80 -- ........*..............................................................
81 -- .........*.............................................................
82 -- ..........*............................................................
83 -- ...........*...........................................................
84 -- ............*..........................................................
85 -- .............*.........................................................
```

```
 86 -- ..............*........................................................
 87 -- ...............*.......................................................
 88 -- ................*......................................................
 89 -- .................*.....................................................
 90 -- ..................*....................................................
 91 -- ...................*...................................................
 92 -- ....................*..................................................
 93 -- .....................*.................................................
 94 -- ......................*................................................
 95 -- .......................*...............................................
 96 -- ........................*..............................................
 97 -- .........................*.............................................
 98 -- ..........................*............................................
 99 -- ...........................*...........................................
100 -- ............................*..........................................
101 -- .............................*.........................................
102 -- ..............................*........................................
103 -- ...............................*.......................................
104 -- ................................*......................................
105 -- .................................*.....................................
106 -- ..................................*....................................
107 -- ...................................*...................................
108 -- ....................................*..................................
109 -- .....................................*.................................
110 -- ......................................*................................
111 -- .......................................*...............................
112 -- ........................................*..............................
113 -- .........................................*.............................
114 -- ..........................................*............................
115 -- ...........................................*...........................
116 -- ............................................*..........................
117 -- .............................................*.........................
118 -- ..............................................*........................
119 -- ...............................................*.......................
120 -- ................................................*......................
121 -- .................................................*.....................
122 -- ..................................................*....................
123 -- ...................................................*...................
124 -- ....................................................*..................
125 -- .....................................................*.................
126 -- ......................................................*................
127 -- .......................................................*...............
128 -- ........................................................*..............
129 -- .........................................................*.............
130 -- ..........................................................*............
131 -- ...........................................................*...........
132 -- ............................................................*..........
133 -- .............................................................*.........
134 -- ..............................................................*........
135 -- ...............................................................*.......
136 -- ................................................................*......
137 -- .................................................................*.....
138 -- ..................................................................*....
139 -- ...................................................................*...
140 -- ....................................................................*..
141 -- .....................................................................*.
142 -- ......................................................................*
143 -- .......................................................................
144 -- .......................................................................
145 -- .......................................................................
146 -- .......................................................................
147 -- .......................................................................
148 -- .......................................................................
149 -- .......................................................................
150 -- .......................................................................
151 -- .......................................................................
152 -- .......................................................................
153 -- .......................................................................
154 -- .......................................................................
155 -- .......................................................................
156 -- .......................................................................
157 -- .......................................................................
158 -- ................................................**.....................
159 -- ...............................................................******..
160 -- ..............................................................*********
161 -- .......................................................................
162 -- ****...................................................................
163 -- .......................................................................
164 -- .......................................................................
165 -- .......................................................................
166 -- ***********************************************************************
```

```
167 -- .......................................................................
168 -- .......................................................................
169 -- ***********************************************************************
170 -- .......................................................................
171 -- .......................................................................
172 -- .......................................................................
173 -- .......................................................................
174 -- .......................................................................
175 -- ..........................................................**...........
176 -- ..........*********....................................................
177 -- .......................................................................
178 -- .......................................................................
179 -- ***********************************************************************
180 -- ..............................************************....**...........
181 -- ............................**.........................................
182 -- .......................................................................
183 -- .............................................................**********
184 -- ........................................**.............................
185 -- ......................................**...............................
186 -- ..........................................******.......................
187 -- .......................................................................
188 -- ***********************************************************************
189 -- ..............................********************.....................
190 -- .......................................................................
191 -- ..................................................****.................
192 -- ...............................................................********
193 -- .......................................................................
194 -- ...............................................................********
195 -- ......................................................****.............
196 -- .......................................................................
197 -- .......................................................................
198 -- .......................................................................
199 -- .......................................................................
200 -- ....******.............................................................
201 -- ...................*********...........................................
202 -- .......................................................................
203 -- .............................................................**********
204 -- .......................................................................
205 -- .............................................................**********
206 -- .......................................................................
207 -- ..................................**...................................
208 -- ..............................................**.......................
209 -- ..................................**..**..******.......................
210 -- .................................................................**....
211 -- ......................................................**.*.............
212 -- .......................................................................
213 -- .............................................................**********
214 -- ..............................*******************************..........
215 -- ..........................................**...........................
216 -- ..............................****.....................................
217 -- ****...................................................................
218 -- .............................................................**********
219 -- ....*********************************************************..........
220 -- .......................................................................
221 -- .......................................................................
222 -- .................................................................****..
223 -- .......................................................................
224 -- ........................................**......**.....................
225 -- .......................................................................
226 -- .......................................................................
227 -- .......................................................................
228 -- ................***....................................................
229 -- .......................................................................
230 -- ....*******************************************************************
231 -- .......................................................................
232 -- ...................**..................................................
233 -- ***********************************************************************
234 -- ***********************************************************************
235 -- ......................................................**...............
236 -- ..................................****************.....................
237 -- ..............................******************************...........
238 -- .........................***...........................................
239 -- ......................................**..******.......................
240 -- ....................................**..**......**.....................
241 -- ....................................**.................................
242 -- .......................................................................
243 -- ...............****....................................................
244 -- .......................................................................
245 -- .......................*.***...........................................
246 -- .......................................................................
247 -- ....**.................................................................
```

```
248 -- ........................................................................
249 -- ........................................................................
250 -- ..........**............................................................
251 -- ........................................................................
252 -- ****....................................................................
253 -- ............**..........................................................
254 -- .....................................................**.................
255 -- .......*.*..............................................................
256 -- .................**.....................................................
257 -- ..............................*************************.................
258 -- ........................................................................
259 -- ...................***.*.***............................................
260 -- ************************************************************************
261 -- ...................***********..........................................
262 -- ........................................................................
263 -- ****....................................................................
264 -- ..............................********************************..........
265 -- ........................................................................
266 -- .......***..............................................................
267 -- ....***.................................................................
268 -- ........................................................................
269 -- ..........**...****.....................................................
270 -- ****....................................................................
271 -- ..........................**............................................
272 -- ....******....................********************************..........
273 -- ...............................................................*********
274 -- ............***.........................................................
275 -- ...................*****.***............................................
276 -- ...................................................**...................
277 -- ........................................................................
278 -- ........................................................................
279 -- ........................................................................
280 -- ........................................................................
281 -- ************************************************************************
282 -- ......................*.*...............................................
283 -- .........................**.............................................
284 -- ...................***********..........................................
285 -- ************************************************************************
286 -- ........................................................................
287 -- ..........********************..........................................
288 -- ........................................................................
289 -- ....................................................***.................
290 -- ..........********************..........................................
291 -- ****....................................................................
292 -- .....................*.*.***............................................
293 -- ..........********************..........................................
294 -- ........................................................................
295 -- ..........**..*****.....................................................
296 -- ........................................................................
297 -- ....................................................................**..
298 -- ..................................................................**.*..
299 -- ....***.*...............................................................
300 -- ................................................................******.*
301 -- .................................................................*****..
302 -- ................................................................*******.
303 -- ................................................................*.****..
304 -- ................................................................**......
305 -- ......................................................................**
306 -- ........................................................................
307 -- ........................................................................
308 -- ........................................................................
309 -- ....******..............................................................
310 -- ........................................................................
311 -- ************************************************************************
312 -- ..................................................................***...
313 -- ........................................................................
314 -- ......****..............................................................
315 -- ........................................................................
316 -- ************************************************************************
317 -- ************************************************************************
318 -- ..............................................................*.********
319 -- ........................................................................
320 -- ................**......................................................
321 -- ........................................................................
322 -- ............*******.....................................................
323 -- ........................................................................
324 -- ...................***********..........................................
325 -- ........................................................................
326 -- ....***************.....................................................
327 -- ..............................*********************........**...........
328 -- ........................................................................
```

```
329 -- ..........................................****.........................
330 -- .............................................................**........
331 -- ..............................*..*.....................................
332 -- .......................................................................
333 -- .......................................................................
334 -- ...................***.................................................
335 -- *..*...................................................................
336 -- .......................................................................
337 -- ..........................................***..........................
338 -- .......................................................................
339 -- .......................................................................
340 -- .......................................................................
341 -- .......................................................................
342 -- ..........................................***.**.......................
343 -- .......................................................................
344 -- ...............................**......................................
345 -- ..........................................**.*.........................
346 -- ............................................**.........................
347 -- ..............................*.**.....................................
348 -- ...................****.*..............................................
349 -- .......................................................................
350 -- ****...................................................................
351 -- .......................................................................
352 -- ...............................*.*.....................................
353 -- ................................**.....................................
354 -- ............................................*.**.......................
355 -- ..............................**.*.....................................
356 -- .......................................................................
357 -- ..............................***......................................
358 -- .......................................................................
359 -- ..............................*.*......................................
360 -- .......................................................................
361 -- .......................................................................
362 -- ..**...................................................................
363 -- ..........................................**..**.......................
364 -- ...............................***.....................................
365 -- .**....................................................................
366 -- ..............................**.......................................
367 -- .*.*...................................................................
368 -- .......................................................................
369 -- ****...................................................................
370 -- ............................................****.......................
371 -- .............................................***.......................
372 -- .......................................................................
373 -- *.**...................................................................
374 -- *.*....................................................................
375 -- ....*****..............................................................
376 -- ...................**..*.***...........................................
377 -- ..........................................**.***.......................
378 -- ..........***************************************************..........
379 -- ****...................................................................
380 -- ***********************************************************************
381 -- ...................*********...........................................
382 -- .......................................................................
383 -- .......................................................................
384 -- ***....................................................................
385 -- ***********************************************************************
386 -- **.*...................................................................
387 -- .***...................................................................
388 -- .......................................................................
389 -- ..........*****........................................................
390 -- .......................................................................
391 -- **.....................................................................
392 -- .......................................................................
393 -- .......................................................................
394 -- ***********************************************************************
395 -- ***********************************************************************
396 -- ***********************************************************************
397 -- .......................................................................
398 -- ....*********************************************************..........
399 -- ..........*********.........**.........................................
400 -- ...................**.*.*..............................................
401 -- .......................................................................
402 -- ****...................................................................
403 -- .......................................................................
404 -- .......................................................................
405 -- .......................................................................
406 -- ***********************************************************************
407 -- ***********************************************************************
408 -- .......................................................................
409 -- .......................................................................
```

```
410 -- ........................................................................
411 -- ........................................................................
412 -- ........................................................................
413 -- ........................................................................
414 -- ........................................................................
415 -- ........................................................................
416 -- ************************************************************************
417 -- ........................................................................
418 -- ........................................................................
419 -- ..........****.****.....................................................
420 -- ........................................................................
421 -- ........................................................................
422 -- ...................*******************************************..........
423 -- ************************************************************************
424 -- ........................................................................
425 -- ************************************************************************
426 -- ************************************************************************
427 -- ........................................................................
428 -- ........................................................................
429 -- ........................................................................
430 -- ************************************************************************
431 -- ....**.***..............................................................
432 -- ........................................................................
433 -- ........................................................................
434 -- ........................................................................
435 -- ........................................................................
436 -- ........................................................................
437 -- ************************************************************************
438 -- ************************************************************************
439 -- ........................................................................
440 -- ........................................................................
441 -- ........................................................................
442 -- ........................................................................
443 -- ..................................................***...................
444 -- ..................................................**.*..................
445 -- ........................................................................
446 -- ..........*.*******.....................................................
447 -- ........................................................................
448 -- ************************************************************************
449 -- ************************************************************************
450 -- ..........***.*****.....................................................
451 -- ........................................................................
452 -- ..........**.******.....................................................
453 -- ****....................................................................
454 -- ****....................................................................
455 -- ........................................................................
456 -- ....*.****..............................................................
457 -- ........................................................................
--------------------------------------------------------------------------------

 ID -- Partition (continued)
--------------------------------------------------------------------------------
  1 -- **************
  2 -- ..............
  3 -- ..............
  4 -- ..............
  5 -- ..............
  6 -- ..............
  7 -- ..............
  8 -- ..............
  9 -- ..............
 10 -- ..............
 11 -- ..............
 12 -- ..............
 13 -- ..............
 14 -- ..............
 15 -- ..............
 16 -- ..............
 17 -- ..............
 18 -- ..............
 19 -- ..............
 20 -- ..............
 21 -- ..............
 22 -- ..............
 23 -- ..............
 24 -- ..............
 25 -- ..............
 26 -- ..............
 27 -- ..............
 28 -- ..............
 29 -- ..............
```

30 -- ....
31 -- ....
32 -- ....
33 -- ....
34 -- ....
35 -- ....
36 -- ....
37 -- ....
38 -- ....
39 -- ....
40 -- ....
41 -- ....
42 -- ....
43 -- ....
44 -- ....
45 -- ....
46 -- ....
47 -- ....
48 -- ....
49 -- ....
50 -- ....
51 -- ....
52 -- ....
53 -- ....
54 -- ....
55 -- ....
56 -- ....
57 -- ....
58 -- ....
59 -- ....
60 -- ....
61 -- ....
62 -- ....
63 -- ....
64 -- ....
65 -- ....
66 -- ....
67 -- ....
68 -- ....
69 -- ....
70 -- ....
71 -- ....
72 -- ....
73 -- ....
74 -- ....
75 -- ....
76 -- ....
77 -- ....
78 -- ....
79 -- ....
80 -- ....
81 -- ....
82 -- ....
83 -- ....
84 -- ....
85 -- ....
86 -- ....
87 -- ....
88 -- ....
89 -- ....
90 -- ....
91 -- ....
92 -- ....
93 -- ....
94 -- ....
95 -- ....
96 -- ....
97 -- ....
98 -- ....
99 -- ....
100 -- ....
101 -- ....
102 -- ....
103 -- ....
104 -- ....
105 -- ....
106 -- ....
107 -- ....
108 -- ....
109 -- ....
110 -- ....

```
111 -- ..............
112 -- ..............
113 -- ..............
114 -- ..............
115 -- ..............
116 -- ..............
117 -- ..............
118 -- ..............
119 -- ..............
120 -- ..............
121 -- ..............
122 -- ..............
123 -- ..............
124 -- ..............
125 -- ..............
126 -- ..............
127 -- ..............
128 -- ..............
129 -- ..............
130 -- ..............
131 -- ..............
132 -- ..............
133 -- ..............
134 -- ..............
135 -- ..............
136 -- ..............
137 -- ..............
138 -- ..............
139 -- ..............
140 -- ..............
141 -- ..............
142 -- ..............
143 -- *.............
144 -- .*............
145 -- ..*...........
146 -- ...*..........
147 -- ....*.........
148 -- .....*........
149 -- ......*.......
150 -- .......*......
151 -- ........*.....
152 -- .........*....
153 -- ..........*...
154 -- ...........*..
155 -- ............*.
156 -- .............*
157 -- ..............
158 -- ..............
159 -- ..............
160 -- ******........
161 -- ..............
162 -- ..............
163 -- ..............
164 -- ..............
165 -- ..............
166 -- **************
167 -- ..............
168 -- .........*....
169 -- **************
170 -- ..............
171 -- ..............
172 -- ..............
173 -- ..............
174 -- ..............
175 -- ..............
176 -- ..............
177 -- .......**.....
178 -- ..............
179 -- **************
180 -- ..............
181 -- ..............
182 -- ..............
183 -- *******.......
184 -- ..............
185 -- ..............
186 -- ..............
187 -- **............
188 -- **************
189 -- ..............
190 -- ..............
191 -- ..............
```

```
192 -- ..............
193 -- ..............
194 -- ****..........
195 -- ..............
196 -- ****..........
197 -- ..............
198 -- ..............
199 -- ..............
200 -- ..............
201 -- ..............
202 -- ..............
203 -- ****..........
204 -- ..............
205 -- *****.........
206 -- ..............
207 -- ..............
208 -- ..............
209 -- ..............
210 -- ..............
211 -- ..............
212 -- ..............
213 -- *********.....
214 -- ..............
215 -- ..............
216 -- ..............
217 -- ..............
218 -- *********..*..
219 -- ..........*.**
220 -- ..............
221 -- ..............
222 -- ..............
223 -- ..............
224 -- ..............
225 -- ..............
226 -- ..............
227 -- ..............
228 -- ..............
229 -- ..............
230 -- *********.****
231 -- ..............
232 -- ..............
233 -- **************
234 -- *********.****
235 -- ..............
236 -- ..............
237 -- ..............
238 -- ..............
239 -- ..............
240 -- ..............
241 -- ..............
242 -- ..............
243 -- ..............
244 -- ..............
245 -- ..............
246 -- ..............
247 -- ..............
248 -- ..............
249 -- ..............
250 -- ..............
251 -- ..............
252 -- ..............
253 -- ..............
254 -- ..............
255 -- ..............
256 -- ..............
257 -- ..............
258 -- ..............
259 -- ..............
260 -- **************
261 -- ..............
262 -- ..............
263 -- ..............
264 -- .............*
265 -- ..............
266 -- ..............
267 -- ..............
268 -- ..............
269 -- ..............
270 -- ..............
271 -- ..............
272 -- .............*
```

```
273 -- ****..........
274 -- ..............
275 -- ..............
276 -- ..............
277 -- .........*....
278 -- ..............
279 -- ..............
280 -- ..............
281 -- **************
282 -- ..............
283 -- ..............
284 -- ..........*...
285 -- *********.****
286 -- ..............
287 -- ..........*.*.
288 -- ..............
289 -- ..............
290 -- ..............
291 -- ..............
292 -- ..............
293 -- ..........*...
294 -- ..............
295 -- ..............
296 -- **.*..........
297 -- ..............
298 -- ..............
299 -- ..............
300 -- ..............
301 -- ..............
302 -- ..............
303 -- ..............
304 -- ..............
305 -- ..............
306 -- ..**..........
307 -- ***...........
308 -- ..............
309 -- .............*
310 -- ..............
311 -- **************
312 -- ..............
313 -- ..............
314 -- ..............
315 -- ..............
316 -- **************
317 -- **************
318 -- ****..........
319 -- ..............
320 -- ..............
321 -- ..............
322 -- ..............
323 -- ..............
324 -- ..........*.*.
325 -- ..............
326 -- ..............
327 -- ..............
328 -- ..............
329 -- ..............
330 -- ..............
331 -- ..............
332 -- .........*....
333 -- ..............
334 -- ..............
335 -- ..............
336 -- ..............
337 -- ..............
338 -- ..............
339 -- ..............
340 -- .........*....
341 -- ..............
342 -- ..............
343 -- ..............
344 -- ..............
345 -- ..............
346 -- ..............
347 -- ..............
348 -- ..............
349 -- ..............
350 -- ..............
351 -- ..............
352 -- ..............
353 -- ..............
```

```
354 -- ..............
355 -- ..............
356 -- ..............
357 -- ..............
358 -- ..............
359 -- ..............
360 -- ..............
361 -- ..............
362 -- ..............
363 -- ..............
364 -- ..............
365 -- ..............
366 -- ..............
367 -- ..............
368 -- ..............
369 -- ..............
370 -- ..............
371 -- ..............
372 -- ..............
373 -- ..............
374 -- ..............
375 -- ..............
376 -- ..............
377 -- ..............
378 -- ..........*.**
379 -- ..............
380 -- **************
381 -- ..........*...
382 -- ..............
383 -- ..............
384 -- ..............
385 -- **************
386 -- ..............
387 -- ..............
388 -- ..............
389 -- ..............
390 -- ..............
391 -- ..............
392 -- ..............
393 -- ..............
394 -- **************
395 -- **************
396 -- **************
397 -- ..............
398 -- ..........*..*
399 -- ..............
400 -- ..............
401 -- ..............
402 -- ..............
403 -- ..............
404 -- ..............
405 -- ..............
406 -- **************
407 -- **************
408 -- ..............
409 -- ..............
410 -- ..............
411 -- ..............
412 -- ..............
413 -- ..............
414 -- ..............
415 -- ..............
416 -- **************
417 -- ..............
418 -- ..............
419 -- ..............
420 -- ..............
421 -- ..............
422 -- ..........*.**
423 -- **************
424 -- ..............
425 -- **************
426 -- **************
427 -- ..............
428 -- .........*....
429 -- ..............
430 -- **************
431 -- ..............
432 -- ..............
433 -- ..............
434 -- ..............
```

```
435 -- ..............
436 -- ..............
437 -- **************
438 -- **************
439 -- ..............
440 -- ..............
441 -- ..............
442 -- ..............
443 -- ..............
444 -- ..............
445 -- ..............
446 -- ..............
447 -- ..............
448 -- **************
449 -- **************
450 -- ..............
451 -- ..............
452 -- ..............
453 -- ..............
454 -- ..............
455 -- ..............
456 -- ..............
457 -- ..............
--------------------------------------------------------------------------------
```

Summary statistics for informative taxon bipartitions
(saved to file "/infile_converted.phy.tstat"):

ID	#obs	Probab.	Sd(s)+	Min(s)	Max(s)	Nruns
157	3002	1.000000	0.000000	1.000000	1.000000	2
158	3002	1.000000	0.000000	1.000000	1.000000	2
159	3002	1.000000	0.000000	1.000000	1.000000	2
160	3002	1.000000	0.000000	1.000000	1.000000	2
161	3002	1.000000	0.000000	1.000000	1.000000	2
162	3002	1.000000	0.000000	1.000000	1.000000	2
163	3002	1.000000	0.000000	1.000000	1.000000	2
164	3002	1.000000	0.000000	1.000000	1.000000	2
165	3002	1.000000	0.000000	1.000000	1.000000	2
166	3002	1.000000	0.000000	1.000000	1.000000	2
167	3002	1.000000	0.000000	1.000000	1.000000	2
168	3002	1.000000	0.000000	1.000000	1.000000	2
169	3002	1.000000	0.000000	1.000000	1.000000	2
170	3002	1.000000	0.000000	1.000000	1.000000	2
171	3002	1.000000	0.000000	1.000000	1.000000	2
172	3002	1.000000	0.000000	1.000000	1.000000	2
173	3002	1.000000	0.000000	1.000000	1.000000	2
174	3002	1.000000	0.000000	1.000000	1.000000	2
175	3002	1.000000	0.000000	1.000000	1.000000	2
176	3002	1.000000	0.000000	1.000000	1.000000	2
177	3002	1.000000	0.000000	1.000000	1.000000	2
178	3002	1.000000	0.000000	1.000000	1.000000	2
179	3002	1.000000	0.000000	1.000000	1.000000	2
180	3002	1.000000	0.000000	1.000000	1.000000	2
181	3002	1.000000	0.000000	1.000000	1.000000	2
182	3002	1.000000	0.000000	1.000000	1.000000	2
183	3002	1.000000	0.000000	1.000000	1.000000	2
184	3002	1.000000	0.000000	1.000000	1.000000	2
185	3002	1.000000	0.000000	1.000000	1.000000	2
186	3002	1.000000	0.000000	1.000000	1.000000	2
187	3002	1.000000	0.000000	1.000000	1.000000	2
188	3002	1.000000	0.000000	1.000000	1.000000	2
189	3002	1.000000	0.000000	1.000000	1.000000	2
190	3002	1.000000	0.000000	1.000000	1.000000	2
191	3002	1.000000	0.000000	1.000000	1.000000	2
192	3002	1.000000	0.000000	1.000000	1.000000	2
193	3002	1.000000	0.000000	1.000000	1.000000	2
194	3002	1.000000	0.000000	1.000000	1.000000	2
195	3002	1.000000	0.000000	1.000000	1.000000	2
196	3002	1.000000	0.000000	1.000000	1.000000	2
197	3002	1.000000	0.000000	1.000000	1.000000	2
198	3002	1.000000	0.000000	1.000000	1.000000	2
199	3002	1.000000	0.000000	1.000000	1.000000	2
200	3002	1.000000	0.000000	1.000000	1.000000	2
201	3002	1.000000	0.000000	1.000000	1.000000	2
202	3002	1.000000	0.000000	1.000000	1.000000	2
203	3002	1.000000	0.000000	1.000000	1.000000	2
204	3002	1.000000	0.000000	1.000000	1.000000	2
205	3001	0.999667	0.000471	0.999334	1.000000	2
206	3001	0.999667	0.000471	0.999334	1.000000	2
207	3001	0.999667	0.000471	0.999334	1.000000	2

208	3000	0.999334	0.000942	0.998668	1.000000	2
209	2999	0.999001	0.001413	0.998001	1.000000	2
210	2999	0.999001	0.000471	0.998668	0.999334	2
211	2998	0.998668	0.001884	0.997335	1.000000	2
212	2996	0.998001	0.001884	0.996669	0.999334	2
213	2996	0.998001	0.000942	0.997335	0.998668	2
214	2995	0.997668	0.002355	0.996003	0.999334	2
215	2994	0.997335	0.001884	0.996003	0.998668	2
216	2987	0.995003	0.007066	0.990007	1.000000	2
217	2986	0.994670	0.007537	0.989340	1.000000	2
218	2986	0.994670	0.002827	0.992672	0.996669	2
219	2979	0.992338	0.002355	0.990673	0.994004	2
220	2978	0.992005	0.010364	0.984677	0.999334	2
221	2977	0.991672	0.007066	0.986676	0.996669	2
222	2973	0.990340	0.001413	0.989340	0.991339	2
223	2972	0.990007	0.001884	0.988674	0.991339	2
224	2971	0.989674	0.005182	0.986009	0.993338	2
225	2969	0.989007	0.003298	0.986676	0.991339	2
226	2966	0.988008	0.005653	0.984011	0.992005	2
227	2963	0.987009	0.007066	0.982012	0.992005	2
228	2960	0.986009	0.013191	0.976682	0.995336	2
229	2957	0.985010	0.002355	0.983344	0.986676	2
230	2954	0.984011	0.004711	0.980680	0.987342	2
231	2942	0.980013	0.008480	0.974017	0.986009	2
232	2907	0.968354	0.024026	0.951366	0.985343	2
233	2900	0.966023	0.022612	0.950033	0.982012	2
234	2895	0.964357	0.007066	0.959360	0.969354	2
235	2892	0.963358	0.000000	0.963358	0.963358	2
236	2886	0.961359	0.009422	0.954697	0.968021	2
237	2883	0.960360	0.020257	0.946036	0.974684	2
238	2854	0.950700	0.003769	0.948035	0.953364	2
239	2824	0.940706	0.016959	0.928714	0.952698	2
240	2804	0.934044	0.033919	0.910060	0.958028	2
241	2798	0.932045	0.000000	0.932045	0.932045	2
242	2744	0.914057	0.014133	0.904064	0.924051	2
243	2667	0.888408	0.006124	0.884077	0.892738	2
244	2646	0.881412	0.013191	0.872085	0.890740	2
245	2645	0.881079	0.005182	0.877415	0.884744	2
246	2632	0.876749	0.065010	0.830779	0.922718	2
247	2613	0.870420	0.034390	0.846103	0.894737	2
248	2584	0.860759	0.025439	0.842771	0.878748	2
249	2565	0.854430	0.011777	0.846103	0.862758	2
250	2545	0.847768	0.047580	0.814124	0.881412	2
251	2464	0.820786	0.014133	0.810793	0.830779	2
252	2428	0.808794	0.051820	0.772152	0.845436	2
253	2408	0.802132	0.038629	0.774817	0.829447	2
254	2360	0.786143	0.026381	0.767488	0.804797	2
255	2316	0.771486	0.006595	0.766822	0.776149	2
256	2227	0.741839	0.008009	0.736176	0.747502	2
257	2177	0.725183	0.008951	0.718854	0.731512	2
258	2161	0.719853	0.021199	0.704863	0.734843	2
259	2100	0.699534	0.079143	0.643571	0.755496	2
260	1998	0.665556	0.035803	0.640240	0.690873	2
261	1907	0.635243	0.001413	0.634244	0.636243	2
262	1903	0.633911	0.015546	0.622918	0.644903	2
263	1879	0.625916	0.017430	0.613591	0.638241	2
264	1865	0.621252	0.020257	0.606929	0.635576	2
265	1850	0.616256	0.072548	0.564957	0.667555	2
266	1797	0.598601	0.020257	0.584277	0.612925	2
267	1779	0.592605	0.024968	0.574950	0.610260	2
268	1775	0.591272	0.016488	0.579614	0.602931	2
269	1714	0.570953	0.050878	0.534977	0.606929	2
270	1566	0.521652	0.032976	0.498334	0.544970	2
271	1547	0.515323	0.047580	0.481679	0.548967	2
272	1538	0.512325	0.042398	0.482345	0.542305	2
273	1531	0.509993	0.014604	0.499667	0.520320	2
274	1521	0.506662	0.073961	0.454364	0.558961	2
275	1495	0.498001	0.052291	0.461026	0.534977	2
276	1489	0.496003	0.003298	0.493671	0.498334	2
277	1459	0.486009	0.004240	0.483011	0.489007	2
278	1446	0.481679	0.035803	0.456362	0.506995	2
279	1442	0.480346	0.050878	0.444370	0.516322	2
280	1434	0.477682	0.031092	0.455696	0.499667	2
281	1432	0.477015	0.032976	0.453698	0.500333	2
282	1416	0.471686	0.068779	0.423051	0.520320	2
283	1408	0.469021	0.043340	0.438374	0.499667	2
284	1402	0.467022	0.019786	0.453031	0.481013	2
285	1401	0.466689	0.017430	0.454364	0.479014	2
286	1382	0.460360	0.016959	0.448368	0.472352	2
287	1297	0.432045	0.029679	0.411059	0.453031	2
288	1266	0.421719	0.016959	0.409727	0.433711	2

289	1232	0.410393	0.004711	0.407062	0.413724	2
290	1149	0.382745	0.055118	0.343771	0.421719	2
291	1147	0.382079	0.011777	0.373751	0.390406	2
292	1144	0.381079	0.042398	0.351099	0.411059	2
293	1127	0.375416	0.048522	0.341106	0.409727	2
294	1070	0.356429	0.033919	0.332445	0.380413	2
295	1046	0.348434	0.049936	0.313125	0.383744	2
296	1032	0.343771	0.012248	0.335110	0.352432	2
297	1031	0.343438	0.014604	0.333111	0.353764	2
298	1030	0.343105	0.008480	0.337109	0.349101	2
299	1025	0.341439	0.013662	0.331779	0.351099	2
300	1013	0.337442	0.011777	0.329114	0.345769	2
301	1009	0.336109	0.026852	0.317122	0.355097	2
302	997	0.332112	0.011777	0.323784	0.340440	2
303	996	0.331779	0.007537	0.326449	0.337109	2
304	993	0.330779	0.034390	0.306462	0.355097	2
305	992	0.330446	0.000000	0.330446	0.330446	2
306	989	0.329447	0.014604	0.319121	0.339773	2
307	981	0.326782	0.002355	0.325117	0.328448	2
308	970	0.323118	0.017901	0.310460	0.335776	2
309	954	0.317788	0.017901	0.305130	0.330446	2
310	949	0.316123	0.008009	0.310460	0.321785	2
311	940	0.313125	0.020728	0.298468	0.327781	2
312	933	0.310793	0.007066	0.305796	0.315789	2
313	883	0.294137	0.007066	0.289141	0.299134	2
314	866	0.288474	0.038629	0.261159	0.315789	2
315	856	0.285143	0.005653	0.281146	0.289141	2
316	807	0.268821	0.038158	0.241839	0.295803	2
317	799	0.266156	0.042869	0.235843	0.296469	2
318	788	0.262492	0.002827	0.260493	0.264490	2
319	788	0.262492	0.009422	0.255829	0.269154	2
320	759	0.252831	0.005182	0.249167	0.256496	2
321	742	0.247169	0.014133	0.237175	0.257162	2
322	733	0.244171	0.059828	0.201865	0.286476	2
323	717	0.238841	0.009893	0.231845	0.245836	2
324	712	0.237175	0.016017	0.225849	0.248501	2
325	711	0.236842	0.014604	0.226516	0.247169	2
326	705	0.234843	0.057944	0.193871	0.275816	2
327	702	0.233844	0.004711	0.230513	0.237175	2
328	698	0.232512	0.017901	0.219853	0.245170	2
329	693	0.230846	0.008009	0.225183	0.236509	2
330	683	0.227515	0.017430	0.215190	0.239840	2
331	659	0.219520	0.010835	0.211859	0.227182	2
332	651	0.216855	0.024968	0.199201	0.234510	2
333	651	0.216855	0.029679	0.195869	0.237841	2
334	651	0.216855	0.026852	0.197868	0.235843	2
335	643	0.214191	0.008009	0.208528	0.219853	2
336	633	0.210859	0.010835	0.203198	0.218521	2
337	632	0.210526	0.013191	0.201199	0.219853	2
338	630	0.209860	0.013191	0.200533	0.219187	2
339	630	0.209860	0.027323	0.190540	0.229181	2
340	628	0.209194	0.003769	0.206529	0.211859	2
341	625	0.208195	0.002355	0.206529	0.209860	2
342	622	0.207195	0.000942	0.206529	0.207861	2
343	621	0.206862	0.008009	0.201199	0.212525	2
344	620	0.206529	0.009422	0.199867	0.213191	2
345	619	0.206196	0.003298	0.203864	0.208528	2
346	618	0.205863	0.021670	0.190540	0.221186	2
347	617	0.205530	0.007066	0.200533	0.210526	2
348	617	0.205530	0.039101	0.177881	0.233178	2
349	602	0.200533	0.002827	0.198534	0.202532	2
350	601	0.200200	0.042869	0.169887	0.230513	2
351	597	0.198867	0.005182	0.195203	0.202532	2
352	597	0.198867	0.009893	0.191872	0.205863	2
353	595	0.198201	0.004240	0.195203	0.201199	2
354	594	0.197868	0.010364	0.190540	0.205197	2
355	592	0.197202	0.000000	0.197202	0.197202	2
356	592	0.197202	0.010364	0.189873	0.204530	2
357	589	0.196203	0.010835	0.188541	0.203864	2
358	586	0.195203	0.014133	0.185210	0.205197	2
359	583	0.194204	0.003298	0.191872	0.196536	2
360	582	0.193871	0.006595	0.189207	0.198534	2
361	579	0.192871	0.004240	0.189873	0.195869	2
362	579	0.192871	0.006124	0.188541	0.197202	2
363	579	0.192871	0.000471	0.192538	0.193205	2
364	576	0.191872	0.009422	0.185210	0.198534	2
365	564	0.187875	0.005653	0.183877	0.191872	2
366	564	0.187875	0.004711	0.184544	0.191206	2
367	561	0.186875	0.004240	0.183877	0.189873	2
368	559	0.186209	0.010835	0.178548	0.193871	2
369	559	0.186209	0.006124	0.181879	0.190540	2

370	558	0.185876	0.011306	0.177881	0.193871	2
371	555	0.184877	0.006124	0.180546	0.189207	2
372	546	0.181879	0.033919	0.157895	0.205863	2
373	545	0.181546	0.014604	0.171219	0.191872	2
374	543	0.180879	0.006124	0.176549	0.185210	2
375	541	0.180213	0.002355	0.178548	0.181879	2
376	537	0.178881	0.005182	0.175217	0.182545	2
377	534	0.177881	0.008480	0.171885	0.183877	2
378	531	0.176882	0.011777	0.168554	0.185210	2
379	530	0.176549	0.024497	0.159227	0.193871	2
380	527	0.175550	0.008951	0.169221	0.181879	2
381	525	0.174883	0.026852	0.155896	0.193871	2
382	525	0.174883	0.041927	0.145237	0.204530	2
383	524	0.174550	0.007537	0.169221	0.179880	2
384	522	0.173884	0.014133	0.163891	0.183877	2
385	521	0.173551	0.040043	0.145237	0.201865	2
386	509	0.169554	0.023083	0.153231	0.185876	2
387	507	0.168887	0.000471	0.168554	0.169221	2
388	493	0.164224	0.019315	0.150566	0.177881	2
389	491	0.163558	0.008951	0.157229	0.169887	2
390	490	0.163225	0.016017	0.151899	0.174550	2
391	486	0.161892	0.006595	0.157229	0.166556	2
392	478	0.159227	0.003769	0.156562	0.161892	2
393	474	0.157895	0.002827	0.155896	0.159893	2
394	469	0.156229	0.000471	0.155896	0.156562	2
395	463	0.154231	0.030621	0.132578	0.175883	2
396	463	0.154231	0.001413	0.153231	0.155230	2
397	459	0.152898	0.024968	0.135243	0.170553	2
398	459	0.152898	0.024968	0.135243	0.170553	2
399	457	0.152232	0.011777	0.143904	0.160560	2
400	453	0.150899	0.034390	0.126582	0.175217	2
401	444	0.147901	0.003769	0.145237	0.150566	2
402	439	0.146236	0.034390	0.121919	0.170553	2
403	432	0.143904	0.011306	0.135909	0.151899	2
404	432	0.143904	0.002827	0.141905	0.145903	2
405	432	0.143904	0.016017	0.132578	0.155230	2
406	430	0.143238	0.002827	0.141239	0.145237	2
407	428	0.142572	0.001884	0.141239	0.143904	2
408	424	0.141239	0.016017	0.129913	0.152565	2
409	422	0.140573	0.002827	0.138574	0.142572	2
410	422	0.140573	0.001884	0.139241	0.141905	2
411	421	0.140240	0.008951	0.133911	0.146569	2
412	415	0.138241	0.010835	0.130580	0.145903	2
413	414	0.137908	0.059357	0.095936	0.179880	2
414	411	0.136909	0.001413	0.135909	0.137908	2
415	400	0.133245	0.015075	0.122585	0.143904	2
416	396	0.131912	0.003769	0.129247	0.134577	2
417	392	0.130580	0.016017	0.119254	0.141905	2
418	390	0.129913	0.020728	0.115256	0.144570	2
419	388	0.129247	0.023555	0.112592	0.145903	2
420	387	0.128914	0.001413	0.127915	0.129913	2
421	386	0.128581	0.017901	0.115923	0.141239	2
422	385	0.128248	0.040043	0.099933	0.156562	2
423	385	0.128248	0.005182	0.124584	0.131912	2
424	380	0.126582	0.014133	0.116589	0.136576	2
425	374	0.124584	0.005653	0.120586	0.128581	2
426	371	0.123584	0.022141	0.107928	0.139241	2
427	370	0.123251	0.006595	0.118588	0.127915	2
428	366	0.121919	0.011306	0.113924	0.129913	2
429	363	0.120919	0.005182	0.117255	0.124584	2
430	360	0.119920	0.034861	0.095270	0.144570	2
431	357	0.118921	0.013662	0.109260	0.128581	2
432	356	0.118588	0.033919	0.094604	0.142572	2
433	354	0.117921	0.018844	0.104597	0.131246	2
434	353	0.117588	0.000471	0.117255	0.117921	2
435	349	0.116256	0.010835	0.108594	0.123917	2
436	345	0.114923	0.008009	0.109260	0.120586	2
437	344	0.114590	0.011306	0.106596	0.122585	2
438	340	0.113258	0.001884	0.111925	0.114590	2
439	338	0.112592	0.003769	0.109927	0.115256	2
440	332	0.110593	0.006595	0.105929	0.115256	2
441	324	0.107928	0.000942	0.107262	0.108594	2
442	324	0.107928	0.005653	0.103931	0.111925	2
443	322	0.107262	0.010364	0.099933	0.114590	2
444	320	0.106596	0.016017	0.095270	0.117921	2
445	320	0.106596	0.008480	0.100600	0.112592	2
446	314	0.104597	0.028265	0.084610	0.124584	2
447	310	0.103264	0.024497	0.085943	0.120586	2
448	305	0.101599	0.024968	0.083944	0.119254	2
449	298	0.099267	0.009422	0.092605	0.105929	2
450	292	0.097268	0.020728	0.082612	0.111925	2

451	290	0.096602	0.006595	0.091939	0.101266	2
452	284	0.094604	0.016017	0.083278	0.105929	2
453	278	0.092605	0.012248	0.083944	0.101266	2
454	277	0.092272	0.018373	0.079280	0.105263	2
455	264	0.087941	0.032034	0.065290	0.110593	2
456	251	0.083611	0.032505	0.060626	0.106596	2
457	186	0.061959	0.056531	0.021985	0.101932	2

+ Convergence diagnostic (standard deviation of split frequencies)
  should approach 0.0 as runs converge.

Summary statistics for branch and node parameters
(saved to file "/infile_converted.phy.vstat"):

Parameter	Mean	Variance	95% HPD Interval Lower	Upper	Median	PSRF+	Nruns
length[1]	0.003898	0.000016	0.000005	0.011906	0.002585	1.002	2
length[2]	0.003963	0.000016	0.000001	0.011944	0.002700	1.000	2
length[3]	0.004167	0.000017	0.000005	0.012563	0.002847	1.002	2
length[4]	0.003887	0.000014	0.000000	0.011430	0.002709	1.004	2
length[5]	0.003976	0.000015	0.000001	0.011918	0.002811	1.000	2
length[6]	0.007399	0.000029	0.000048	0.018008	0.006318	1.000	2
length[7]	0.004277	0.000018	0.000003	0.013335	0.002959	1.001	2
length[8]	0.004304	0.000017	0.000012	0.012554	0.002857	1.000	2
length[9]	0.003967	0.000014	0.000000	0.011936	0.002764	1.000	2
length[10]	0.004166	0.000018	0.000001	0.013269	0.002933	1.001	2
length[11]	0.003873	0.000015	0.000009	0.011328	0.002614	1.000	2
length[12]	0.003723	0.000013	0.000005	0.010983	0.002618	1.000	2
length[13]	0.007668	0.000028	0.000372	0.017515	0.006646	1.000	2
length[14]	0.003813	0.000014	0.000002	0.011150	0.002796	1.008	2
length[15]	0.004461	0.000019	0.000005	0.013563	0.003231	1.001	2
length[16]	0.004119	0.000017	0.000005	0.012343	0.002839	1.003	2
length[17]	0.004329	0.000017	0.000000	0.013142	0.003026	1.001	2
length[18]	0.004170	0.000017	0.000003	0.013018	0.002804	1.001	2
length[19]	0.004033	0.000016	0.000004	0.012407	0.002838	1.000	2
length[20]	0.004115	0.000016	0.000003	0.011826	0.002784	1.000	2
length[21]	0.004377	0.000021	0.000016	0.013003	0.003026	1.000	2
length[22]	0.003974	0.000015	0.000006	0.011634	0.002749	1.000	2
length[23]	0.004093	0.000017	0.000008	0.012927	0.002756	1.001	2
length[24]	0.004423	0.000020	0.000008	0.014039	0.003007	1.011	2
length[25]	0.004192	0.000019	0.000003	0.012897	0.002882	1.000	2
length[26]	0.003974	0.000015	0.000001	0.011712	0.002857	1.000	2
length[27]	0.004016	0.000016	0.000005	0.012234	0.002700	1.000	2
length[28]	0.004087	0.000016	0.000001	0.012144	0.002857	1.000	2
length[29]	0.004056	0.000016	0.000003	0.012213	0.002803	1.001	2
length[30]	0.003907	0.000015	0.000003	0.011894	0.002656	1.000	2
length[31]	0.009424	0.000041	0.000354	0.021476	0.007935	1.000	2
length[32]	0.009285	0.000047	0.000118	0.022402	0.007798	1.000	2
length[33]	0.014830	0.000069	0.001183	0.029545	0.013191	1.003	2
length[34]	0.017974	0.000099	0.002184	0.039031	0.016116	1.004	2
length[35]	0.004942	0.000027	0.000009	0.015127	0.003281	1.000	2
length[36]	0.004384	0.000019	0.000000	0.012497	0.003058	1.000	2
length[37]	0.004745	0.000023	0.000001	0.013758	0.003394	1.000	2
length[38]	0.004728	0.000026	0.000001	0.014818	0.003157	1.000	2
length[39]	0.004640	0.000021	0.000001	0.013896	0.003279	1.000	2
length[40]	0.004790	0.000023	0.000005	0.014338	0.003353	1.000	2
length[41]	0.009407	0.000043	0.000283	0.022357	0.007889	1.006	2
length[42]	0.009743	0.000050	0.000555	0.024446	0.007940	1.000	2
length[43]	0.009544	0.000051	0.000175	0.024038	0.007789	1.007	2
length[44]	0.004400	0.000018	0.000001	0.013461	0.003160	1.000	2
length[45]	0.021525	0.000278	0.000015	0.051944	0.018544	1.004	2
length[46]	0.065997	0.000376	0.028167	0.105710	0.064040	1.000	2
length[47]	0.023408	0.000323	0.000006	0.058804	0.020455	1.002	2
length[48]	0.107057	0.000679	0.057153	0.152646	0.106215	1.005	2
length[49]	0.436896	0.004190	0.302049	0.551248	0.438574	1.000	2
length[50]	0.378842	0.007084	0.223030	0.559129	0.376026	1.000	2
length[51]	0.361918	0.002732	0.260636	0.451162	0.358330	1.000	2
length[52]	0.284029	0.002145	0.196731	0.376941	0.283634	1.000	2
length[53]	0.368093	0.006744	0.178521	0.510033	0.377503	1.004	2
length[54]	0.367328	0.002843	0.270131	0.472313	0.370290	1.000	2
length[55]	0.388522	0.003149	0.283190	0.495083	0.388923	1.001	2
length[56]	0.028661	0.000149	0.007265	0.051581	0.026377	1.001	2
length[57]	0.007511	0.000049	0.000010	0.022176	0.005246	1.001	2
length[58]	0.017662	0.000086	0.002771	0.036850	0.015581	1.000	2
length[59]	0.013485	0.000066	0.001376	0.031075	0.011487	1.001	2
length[60]	0.017853	0.000093	0.001927	0.037421	0.016989	1.013	2
length[61]	0.011076	0.000060	0.000220	0.026464	0.009421	1.000	2
length[62]	0.004541	0.000022	0.000006	0.012928	0.003207	1.000	2

length[63]	0.004498	0.000023	0.000003	0.013995	0.002977	1.000	2
length[64]	0.004374	0.000019	0.000005	0.013576	0.003220	1.000	2
length[65]	0.004647	0.000020	0.000016	0.013424	0.003596	1.000	2
length[66]	0.004055	0.000015	0.000004	0.011990	0.002902	1.001	2
length[67]	0.004335	0.000017	0.000002	0.012194	0.003018	1.000	2
length[68]	0.004331	0.000016	0.000007	0.012116	0.003369	1.002	2
length[69]	0.004394	0.000020	0.000001	0.013219	0.003094	1.000	2
length[70]	0.004539	0.000020	0.000002	0.014033	0.003301	1.002	2
length[71]	0.004381	0.000018	0.000003	0.012965	0.003092	1.002	2
length[72]	0.004039	0.000016	0.000001	0.011907	0.002831	1.000	2
length[73]	0.004159	0.000018	0.000000	0.012371	0.002849	1.001	2
length[74]	0.004614	0.000019	0.000002	0.012986	0.003348	1.000	2
length[75]	0.004285	0.000018	0.000003	0.013020	0.002977	1.002	2
length[76]	0.006016	0.000039	0.000005	0.018833	0.003913	1.002	2
length[77]	0.017696	0.000110	0.001671	0.039681	0.015721	1.002	2
length[78]	0.035004	0.000270	0.002562	0.066915	0.033556	1.000	2
length[79]	0.032101	0.000169	0.011521	0.058314	0.030136	1.009	2
length[80]	0.011464	0.000080	0.000085	0.027748	0.009370	1.000	2
length[81]	0.028565	0.000218	0.001031	0.057146	0.027482	1.009	2
length[82]	0.005399	0.000029	0.000021	0.015908	0.003559	1.000	2
length[83]	0.017309	0.000105	0.000750	0.037273	0.015402	1.002	2
length[84]	0.005672	0.000033	0.000001	0.016447	0.003959	1.005	2
length[85]	0.006018	0.000037	0.000007	0.018110	0.004267	1.000	2
length[86]	0.011137	0.000078	0.000005	0.028052	0.008658	1.004	2
length[87]	0.020440	0.000130	0.002113	0.040794	0.018624	1.000	2
length[88]	0.022522	0.000147	0.002748	0.046780	0.020593	1.002	2
length[89]	0.006192	0.000035	0.000001	0.018333	0.004640	1.000	2
length[90]	0.040073	0.000229	0.015971	0.071946	0.037785	1.003	2
length[91]	0.011082	0.000060	0.000294	0.026168	0.009357	1.000	2
length[92]	0.011230	0.000059	0.000666	0.026244	0.009392	1.000	2
length[93]	0.018879	0.000113	0.000494	0.038807	0.017309	1.000	2
length[94]	0.011426	0.000063	0.000334	0.026894	0.009667	1.000	2
length[95]	0.031182	0.000246	0.004968	0.060993	0.029373	1.001	2
length[96]	0.052668	0.000871	0.000851	0.102201	0.053333	1.000	2
length[97]	0.065381	0.000538	0.021907	0.107725	0.063840	1.004	2
length[98]	0.019219	0.000139	0.001673	0.043514	0.016434	1.000	2
length[99]	0.054614	0.000359	0.022341	0.092707	0.052237	1.004	2
length[100]	0.005988	0.000037	0.000000	0.018325	0.004259	1.000	2
length[101]	0.006276	0.000034	0.000014	0.017707	0.004505	1.001	2
length[102]	0.005583	0.000033	0.000013	0.017042	0.003751	1.000	2
length[103]	0.005269	0.000027	0.000020	0.016435	0.003650	1.000	2
length[104]	0.005355	0.000026	0.000016	0.014685	0.003954	1.000	2
length[105]	0.005346	0.000032	0.000004	0.016694	0.003530	1.000	2
length[106]	0.005334	0.000029	0.000002	0.015738	0.003727	1.000	2
length[107]	0.005428	0.000027	0.000006	0.015707	0.003945	1.000	2
length[108]	0.005333	0.000027	0.000024	0.016105	0.003719	1.000	2
length[109]	0.005565	0.000030	0.000001	0.015992	0.003952	1.000	2
length[110]	0.005502	0.000034	0.000001	0.016700	0.003760	1.000	2
length[111]	0.005562	0.000027	0.000020	0.016184	0.003979	1.000	2
length[112]	0.005326	0.000029	0.000001	0.017666	0.003540	1.000	2
length[113]	0.005348	0.000026	0.000005	0.015188	0.003916	1.002	2
length[114]	0.005278	0.000026	0.000009	0.015731	0.003771	1.005	2
length[115]	0.005682	0.000034	0.000001	0.016574	0.003895	1.000	2
length[116]	0.005066	0.000026	0.000000	0.015794	0.003505	1.000	2
length[117]	0.005391	0.000026	0.000008	0.015565	0.003986	1.007	2
length[118]	0.005036	0.000028	0.000002	0.015056	0.003310	1.000	2
length[119]	0.010886	0.000062	0.000322	0.026251	0.008887	1.000	2
length[120]	0.005068	0.000028	0.000007	0.016180	0.003462	1.003	2
length[121]	0.005096	0.000025	0.000008	0.016022	0.003542	1.001	2
length[122]	0.021206	0.000175	0.000492	0.046323	0.018663	1.000	2
length[123]	0.011917	0.000072	0.000103	0.028188	0.009977	1.000	2
length[124]	0.006041	0.000033	0.000002	0.017335	0.004289	1.001	2
length[125]	0.005974	0.000034	0.000011	0.018023	0.004235	1.000	2
length[126]	0.018966	0.000110	0.003865	0.039900	0.017046	1.003	2
length[127]	0.017615	0.000109	0.002520	0.037818	0.015414	1.000	2
length[128]	0.012663	0.000082	0.000223	0.029847	0.010574	1.001	2
length[129]	0.058599	0.000370	0.021292	0.093233	0.055785	1.002	2
length[130]	0.006176	0.000037	0.000003	0.018194	0.004390	1.000	2
length[131]	0.005784	0.000032	0.000024	0.017320	0.004200	1.000	2
length[132]	0.379502	0.006305	0.232917	0.538957	0.381223	1.009	2
length[133]	0.011857	0.000066	0.000017	0.026892	0.010973	1.000	2
length[134]	0.013573	0.000062	0.001434	0.029983	0.012102	1.000	2
length[135]	0.004808	0.000022	0.000008	0.013926	0.003452	1.000	2
length[136]	0.004496	0.000019	0.000001	0.013543	0.003251	1.000	2
length[137]	0.004877	0.000023	0.000011	0.013894	0.003295	1.000	2
length[138]	0.004669	0.000020	0.000001	0.013878	0.003260	1.002	2
length[139]	0.004770	0.000023	0.000002	0.013790	0.003381	1.000	2
length[140]	0.004835	0.000023	0.000018	0.014383	0.003246	1.000	2
length[141]	0.014066	0.000062	0.002044	0.029878	0.012819	1.000	2
length[142]	0.013874	0.000057	0.001899	0.028124	0.012439	1.000	2
length[143]	0.014231	0.000061	0.001365	0.028381	0.012709	1.000	2

length[144]	0.004926	0.000024	0.000014	0.014689	0.003348	1.000	2
length[145]	0.004722	0.000021	0.000034	0.014662	0.003339	1.000	2
length[146]	0.004484	0.000021	0.000004	0.013280	0.003102	1.000	2
length[147]	0.223004	0.001420	0.148786	0.288250	0.221469	1.006	2
length[148]	0.350171	0.002196	0.259485	0.433146	0.350765	1.004	2
length[149]	0.270848	0.002916	0.171376	0.379631	0.268795	1.000	2
length[150]	0.443348	0.005644	0.327136	0.631259	0.440925	1.008	2
length[151]	0.452591	0.006586	0.302525	0.584862	0.452473	1.015	2
length[152]	0.640462	0.016376	0.388918	0.876406	0.645539	1.011	2
length[153]	0.880776	0.023880	0.604346	1.233317	0.875358	1.000	2
length[154]	0.504157	0.013251	0.307186	0.753410	0.496469	1.000	2
length[155]	0.935632	0.019772	0.666107	1.218521	0.933123	1.000	2
length[156]	0.871013	0.025240	0.506874	1.164429	0.869220	1.001	2
length[157]	0.044916	0.000232	0.017034	0.074865	0.043664	1.000	2
length[158]	0.065426	0.000390	0.031192	0.106823	0.063812	1.001	2
length[159]	0.013834	0.000067	0.001745	0.030459	0.012047	1.000	2
length[160]	0.168143	0.003100	0.048524	0.266233	0.163592	1.002	2
length[161]	0.531138	0.008039	0.358416	0.681560	0.541387	1.004	2
length[162]	0.780860	0.024043	0.485247	1.070619	0.781009	1.000	2
length[163]	0.475458	0.016354	0.225335	0.702162	0.468207	1.001	2
length[164]	0.265645	0.002865	0.169828	0.376032	0.262783	1.019	2
length[165]	0.059422	0.000363	0.020778	0.094431	0.057744	1.007	2
length[166]	0.074235	0.000587	0.026418	0.120808	0.072809	1.000	2
length[167]	0.031162	0.000127	0.014364	0.055950	0.029746	1.000	2
length[168]	0.817806	0.027913	0.472144	1.128785	0.808016	1.000	2
length[169]	0.344302	0.007082	0.160456	0.482099	0.343271	1.002	2
length[170]	0.043352	0.000219	0.015641	0.069331	0.040910	1.002	2
length[171]	0.431412	0.005513	0.278163	0.563007	0.428096	1.001	2
length[172]	0.489287	0.004851	0.356996	0.621989	0.486262	1.002	2
length[173]	0.719693	0.020435	0.459668	1.007544	0.705608	1.001	2
length[174]	0.697234	0.011198	0.487095	0.886044	0.686237	1.007	2
length[175]	0.466003	0.005125	0.328230	0.617040	0.463353	1.008	2
length[176]	1.022299	0.028458	0.723635	1.353031	1.017192	1.001	2
length[177]	0.384823	0.008728	0.219907	0.558458	0.378956	1.001	2
length[178]	0.015688	0.000061	0.003866	0.031036	0.014184	1.002	2
length[179]	0.041425	0.000169	0.020432	0.069009	0.040251	1.000	2
length[180]	0.181177	0.003128	0.070048	0.283030	0.181415	1.008	2
length[181]	0.588477	0.014005	0.373040	0.837870	0.583864	1.007	2
length[182]	0.842678	0.018171	0.584329	1.094627	0.840597	1.010	2
length[183]	0.442993	0.007576	0.287864	0.621108	0.442111	1.009	2
length[184]	0.021460	0.000125	0.004024	0.042882	0.019631	1.001	2
length[185]	0.032962	0.000198	0.010073	0.060264	0.031160	1.009	2
length[186]	0.069919	0.000427	0.031388	0.106302	0.069194	1.001	2
length[187]	0.024151	0.000109	0.006739	0.044788	0.022641	1.000	2
length[188]	0.015964	0.000063	0.002263	0.032652	0.014356	1.000	2
length[189]	0.227597	0.002835	0.133962	0.331000	0.223166	1.001	2
length[190]	0.351940	0.002406	0.260094	0.445153	0.349662	1.000	2
length[191]	0.416049	0.004888	0.297055	0.566138	0.416820	1.001	2
length[192]	0.019064	0.000083	0.004221	0.036953	0.017860	1.003	2
length[193]	0.653020	0.009171	0.485807	0.847602	0.646655	1.019	2
length[194]	0.022936	0.000100	0.006855	0.043998	0.022139	1.002	2
length[195]	0.337523	0.003412	0.223385	0.457982	0.335049	1.004	2
length[196]	0.019699	0.000085	0.004989	0.038130	0.018201	1.000	2
length[197]	0.046883	0.000258	0.018576	0.079022	0.045681	1.000	2
length[198]	0.212821	0.002360	0.117784	0.299184	0.211314	1.007	2
length[199]	0.250393	0.004108	0.123719	0.374853	0.246169	1.000	2
length[200]	1.008987	0.027530	0.741681	1.334311	0.989002	1.000	2
length[201]	0.737297	0.015652	0.515754	0.978037	0.728489	1.020	2
length[202]	0.074379	0.000389	0.037683	0.115207	0.072731	1.004	2
length[203]	0.084482	0.000799	0.028327	0.135752	0.083525	1.007	2
length[204]	0.012452	0.000045	0.001844	0.026322	0.011522	1.000	2
length[205]	0.104526	0.001230	0.041256	0.176754	0.104282	1.001	2
length[206]	0.089404	0.001022	0.029348	0.147478	0.087560	1.004	2
length[207]	0.016100	0.000097	0.001074	0.036222	0.013929	1.000	2
length[208]	0.016547	0.000092	0.001817	0.034378	0.015133	1.000	2
length[209]	0.019617	0.000127	0.001586	0.042358	0.017342	1.001	2
length[210]	0.009413	0.000048	0.000298	0.022687	0.007936	1.002	2
length[211]	0.028059	0.000204	0.004121	0.055871	0.025910	1.000	2
length[212]	0.009642	0.000045	0.000724	0.022752	0.008172	1.000	2
length[213]	0.347937	0.013270	0.129550	0.590315	0.343743	1.000	2
length[214]	0.354580	0.010307	0.171372	0.580068	0.349393	1.000	2
length[215]	0.011316	0.000067	0.000309	0.026679	0.009272	1.009	2
length[216]	0.048750	0.000419	0.010333	0.088701	0.046086	1.000	2
length[217]	0.285255	0.004512	0.156671	0.412762	0.284244	1.001	2
length[218]	0.449109	0.023650	0.136222	0.723458	0.436197	1.001	2
length[219]	0.453392	0.027917	0.118278	0.756067	0.445215	1.002	2
length[220]	0.018646	0.000089	0.003892	0.039068	0.016887	1.001	2
length[221]	0.014671	0.000077	0.002183	0.033338	0.012912	1.000	2
length[222]	0.008800	0.000042	0.000124	0.021383	0.007419	1.002	2
length[223]	0.014384	0.000066	0.001646	0.029721	0.013139	1.002	2
length[224]	0.016205	0.000093	0.001298	0.033884	0.013987	1.001	2

length[225]	0.017241	0.000090	0.002160	0.034688	0.015587	1.000	2
length[226]	0.015229	0.000093	0.000487	0.034378	0.013434	1.000	2
length[227]	0.161543	0.002384	0.062204	0.246836	0.159123	1.014	2
length[228]	0.040158	0.000245	0.014094	0.071303	0.038373	1.003	2
length[229]	0.397313	0.017088	0.156329	0.675968	0.391415	1.000	2
length[230]	0.407809	0.025899	0.116828	0.734238	0.398505	1.000	2
length[231]	0.007899	0.000033	0.000310	0.019264	0.006618	1.000	2
length[232]	0.010822	0.000058	0.000218	0.026445	0.008919	1.000	2
length[233]	0.401383	0.015404	0.170234	0.645756	0.395718	1.005	2
length[234]	0.360656	0.019075	0.094208	0.611737	0.356135	1.000	2
length[235]	0.016431	0.000096	0.001702	0.035205	0.014870	1.000	2
length[236]	0.035216	0.000426	0.002923	0.076293	0.031568	1.000	2
length[237]	0.154557	0.004404	0.028576	0.281645	0.152379	1.003	2
length[238]	0.039272	0.000277	0.011456	0.075955	0.036806	1.000	2
length[239]	0.012859	0.000082	0.000148	0.031220	0.010815	1.000	2
length[240]	0.013285	0.000079	0.000662	0.031489	0.011268	1.000	2
length[241]	0.010356	0.000056	0.000482	0.025342	0.008622	1.005	2
length[242]	0.024712	0.000169	0.000086	0.049700	0.023264	1.006	2
length[243]	0.015581	0.000105	0.000626	0.035804	0.013532	1.001	2
length[244]	0.175423	0.004025	0.046579	0.282341	0.177478	1.000	2
length[245]	0.046122	0.000367	0.012550	0.088902	0.044504	1.003	2
length[246]	0.014024	0.000074	0.001311	0.029834	0.012237	1.003	2
length[247]	0.025379	0.000220	0.000602	0.054190	0.022787	1.005	2
length[248]	0.010189	0.000049	0.000094	0.023027	0.008893	1.002	2
length[249]	0.012178	0.000061	0.000099	0.027450	0.010872	1.000	2
length[250]	0.011454	0.000067	0.000084	0.027035	0.009591	1.000	2
length[251]	0.018726	0.000096	0.001106	0.036841	0.017339	1.000	2
length[252]	0.012699	0.000059	0.000412	0.026680	0.011259	1.000	2
length[253]	0.011278	0.000069	0.000001	0.027529	0.009530	1.000	2
length[254]	0.015864	0.000106	0.000019	0.034783	0.014566	1.001	2
length[255]	0.023139	0.000154	0.000002	0.045738	0.021486	1.000	2
length[256]	0.014750	0.000098	0.000756	0.033885	0.012529	1.000	2
length[257]	0.109999	0.002309	0.026538	0.208542	0.105621	1.000	2
length[258]	0.227817	0.008196	0.043484	0.389319	0.230682	1.007	2
length[259]	0.026174	0.000161	0.003477	0.049899	0.024253	1.000	2
length[260]	0.007069	0.000030	0.000012	0.017638	0.005730	1.000	2
length[261]	0.269919	0.017174	0.025879	0.523383	0.255417	1.011	2
length[262]	0.009444	0.000043	0.000531	0.023179	0.008033	1.008	2
length[263]	0.159385	0.004175	0.020985	0.269871	0.162101	1.004	2
length[264]	0.306984	0.019463	0.013050	0.549110	0.301512	1.001	2
length[265]	0.030641	0.000198	0.001080	0.055297	0.029470	1.001	2
length[266]	0.027486	0.000249	0.001005	0.056284	0.025494	1.000	2
length[267]	0.026499	0.000217	0.000065	0.054060	0.025101	1.006	2
length[268]	0.022878	0.000141	0.000116	0.043722	0.021818	1.001	2
length[269]	0.011562	0.000071	0.000543	0.029045	0.009682	1.003	2
length[270]	0.172310	0.009572	0.000882	0.355866	0.158927	1.002	2
length[271]	0.026411	0.000205	0.002494	0.053147	0.024991	1.003	2
length[272]	0.331751	0.023295	0.055469	0.616983	0.323331	1.001	2
length[273]	0.009746	0.000059	0.000012	0.024660	0.007925	1.002	2
length[274]	0.011006	0.000066	0.000009	0.027179	0.009291	1.001	2
length[275]	0.043128	0.000581	0.000002	0.083136	0.040705	1.035	2
length[276]	0.015192	0.000112	0.000020	0.036034	0.013224	1.000	2
length[277]	0.254862	0.009736	0.026394	0.403456	0.266955	1.000	2
length[278]	0.029147	0.000241	0.001922	0.056291	0.027594	1.000	2
length[279]	0.008208	0.000033	0.000072	0.019432	0.006908	1.004	2
length[280]	0.014614	0.000111	0.000039	0.034241	0.013074	1.000	2
length[281]	0.009820	0.000042	0.000610	0.022316	0.008468	1.000	2
length[282]	0.023876	0.000212	0.000002	0.050085	0.021592	1.002	2
length[283]	0.023255	0.000147	0.003187	0.046409	0.021639	0.999	2
length[284]	0.246496	0.017796	0.010766	0.486796	0.246717	1.006	2
length[285]	0.196716	0.018998	0.002463	0.453938	0.162744	1.002	2
length[286]	0.247207	0.013280	0.026740	0.456811	0.242675	1.034	2
length[287]	0.250129	0.016959	0.032853	0.504329	0.235359	1.002	2
length[288]	0.111150	0.002274	0.024659	0.207549	0.108136	1.014	2
length[289]	0.012406	0.000093	0.000021	0.031520	0.010252	1.000	2
length[290]	0.282748	0.016587	0.046017	0.529803	0.274200	1.000	2
length[291]	0.010814	0.000058	0.000045	0.025151	0.009673	1.000	2
length[292]	0.010291	0.000075	0.000020	0.026404	0.008032	0.999	2
length[293]	0.214623	0.015547	0.001086	0.411968	0.205919	1.002	2
length[294]	0.010562	0.000051	0.000045	0.024225	0.009981	1.013	2
length[295]	0.010946	0.000070	0.000099	0.027447	0.008818	0.999	2
length[296]	0.004573	0.000022	0.000003	0.014195	0.002995	1.000	2
length[297]	0.004473	0.000020	0.000000	0.013325	0.003179	1.000	2
length[298]	0.004749	0.000020	0.000005	0.013683	0.003437	1.000	2
length[299]	0.019823	0.000136	0.000006	0.040476	0.018778	1.016	2
length[300]	0.004694	0.000022	0.000006	0.014409	0.003018	0.999	2
length[301]	0.004994	0.000023	0.000006	0.014994	0.003580	1.000	2
length[302]	0.004599	0.000023	0.000003	0.013284	0.003069	1.000	2
length[303]	0.004900	0.000024	0.000015	0.014459	0.003268	1.002	2
length[304]	0.004932	0.000026	0.000010	0.016892	0.003177	0.999	2
length[305]	0.004769	0.000027	0.000004	0.014745	0.003306	0.999	2

length[306]	0.004361	0.000019	0.000002	0.012980	0.003116	1.001	2
length[307]	0.004394	0.000021	0.000004	0.014035	0.002925	0.999	2
length[308]	0.115711	0.002223	0.026091	0.205092	0.114760	1.012	2
length[309]	0.266732	0.022242	0.008414	0.540830	0.241178	1.006	2
length[310]	0.003971	0.000015	0.000001	0.011135	0.002765	1.000	2
length[311]	0.140025	0.009918	0.002164	0.333426	0.125035	0.999	2
length[312]	0.004362	0.000018	0.000003	0.012568	0.003149	0.999	2
length[313]	0.004766	0.000023	0.000010	0.014532	0.003381	0.999	2
length[314]	0.024089	0.000205	0.000315	0.050050	0.022611	1.001	2
length[315]	0.004452	0.000020	0.000000	0.013501	0.003002	1.005	2
length[316]	0.021474	0.000129	0.002322	0.044069	0.019033	0.999	2
length[317]	0.005704	0.000025	0.000009	0.015606	0.004313	1.001	2
length[318]	0.007018	0.000040	0.000008	0.020151	0.005158	0.999	2
length[319]	0.010107	0.000053	0.000220	0.025164	0.008299	0.999	2
length[320]	0.012189	0.000081	0.000592	0.029877	0.010114	1.001	2
length[321]	0.004686	0.000021	0.000010	0.014044	0.003126	0.999	2
length[322]	0.010565	0.000061	0.000068	0.024510	0.008839	1.000	2
length[323]	0.004750	0.000022	0.000010	0.013170	0.003520	1.004	2
length[324]	0.193970	0.013529	0.011138	0.415758	0.179179	1.004	2
length[325]	0.004613	0.000020	0.000006	0.013006	0.003258	0.999	2
length[326]	0.293956	0.020465	0.045256	0.570054	0.283076	1.013	2
length[327]	0.089943	0.001590	0.013183	0.162428	0.088281	0.999	2
length[328]	0.018938	0.000090	0.002967	0.037769	0.017854	1.006	2
length[329]	0.006274	0.000038	0.000014	0.017453	0.004527	0.999	2
length[330]	0.005659	0.000034	0.000005	0.016171	0.003753	0.999	2
length[331]	0.005334	0.000031	0.000018	0.015975	0.003526	1.000	2
length[332]	0.138111	0.005145	0.001594	0.259920	0.122954	1.026	2
length[333]	0.004993	0.000025	0.000001	0.015306	0.003534	1.011	2
length[334]	0.007545	0.000051	0.000020	0.021812	0.005564	0.999	2
length[335]	0.004450	0.000020	0.000003	0.012984	0.003210	1.004	2
length[336]	0.004165	0.000019	0.000006	0.011660	0.002928	0.999	2
length[337]	0.005088	0.000027	0.000013	0.014207	0.003513	0.999	2
length[338]	0.004274	0.000017	0.000009	0.012419	0.003171	1.001	2
length[339]	0.004430	0.000020	0.000001	0.013251	0.002914	0.999	2
length[340]	0.114147	0.006816	0.000041	0.259294	0.097155	0.999	2
length[341]	0.003975	0.000015	0.000009	0.011874	0.002815	1.014	2
length[342]	0.005377	0.000027	0.000014	0.016225	0.003758	0.998	2
length[343]	0.003926	0.000014	0.000007	0.011337	0.002852	1.000	2
length[344]	0.005402	0.000031	0.000010	0.016119	0.003606	0.999	2
length[345]	0.005196	0.000027	0.000001	0.014602	0.003502	0.999	2
length[346]	0.005622	0.000030	0.000011	0.016655	0.003926	0.998	2
length[347]	0.005596	0.000027	0.000010	0.016831	0.004278	0.999	2
length[348]	0.037733	0.000390	0.000061	0.066762	0.039619	1.013	2
length[349]	0.114516	0.005359	0.000270	0.239548	0.111762	1.003	2
length[350]	0.115182	0.007693	0.000262	0.281746	0.094088	1.084	2
length[351]	0.004188	0.000018	0.000011	0.013426	0.002901	1.005	2
length[352]	0.005286	0.000027	0.000002	0.016349	0.003638	0.998	2
length[353]	0.005175	0.000023	0.000071	0.014519	0.003957	0.999	2
length[354]	0.005025	0.000021	0.000012	0.014272	0.003684	0.998	2
length[355]	0.005892	0.000033	0.000010	0.017163	0.004167	1.000	2
length[356]	0.004132	0.000015	0.000001	0.011278	0.002975	1.000	2
length[357]	0.005254	0.000028	0.000001	0.015433	0.003540	1.005	2
length[358]	0.004188	0.000017	0.000015	0.012216	0.003119	0.998	2
length[359]	0.005165	0.000026	0.000001	0.014415	0.003673	0.998	2
length[360]	0.004170	0.000019	0.000008	0.013942	0.002937	0.999	2
length[361]	0.003751	0.000013	0.000011	0.011758	0.002619	0.999	2
length[362]	0.004478	0.000021	0.000004	0.013558	0.003065	1.006	2
length[363]	0.005479	0.000028	0.000013	0.016115	0.003731	0.999	2
length[364]	0.005422	0.000030	0.000002	0.016561	0.003625	0.998	2
length[365]	0.004169	0.000017	0.000002	0.011887	0.002866	1.002	2
length[366]	0.005245	0.000028	0.000005	0.015693	0.003703	0.998	2
length[367]	0.004263	0.000017	0.000003	0.012691	0.002995	0.998	2
length[368]	0.004052	0.000013	0.000010	0.011092	0.003022	0.999	2
length[369]	0.004508	0.000022	0.000001	0.012472	0.003100	1.007	2
length[370]	0.005274	0.000030	0.000013	0.016778	0.003731	0.998	2
length[371]	0.005368	0.000025	0.000014	0.015488	0.003961	0.999	2
length[372]	0.153254	0.005000	0.041899	0.313732	0.149463	1.006	2
length[373]	0.004141	0.000016	0.000001	0.012462	0.002780	0.999	2
length[374]	0.003961	0.000016	0.000002	0.012324	0.002608	1.007	2
length[375]	0.020452	0.000199	0.000006	0.048424	0.017814	0.998	2
length[376]	0.006517	0.000039	0.000017	0.020049	0.004512	1.001	2
length[377]	0.005475	0.000030	0.000014	0.015644	0.003911	1.002	2
length[378]	0.240035	0.015360	0.024791	0.459381	0.223867	1.010	2
length[379]	0.004590	0.000018	0.000021	0.012280	0.003326	0.998	2
length[380]	0.004087	0.000015	0.000006	0.010756	0.002843	0.998	2
length[381]	0.233890	0.010667	0.042657	0.439108	0.233670	1.027	2
length[382]	0.004045	0.000016	0.000011	0.011732	0.002680	1.000	2
length[383]	0.004297	0.000017	0.000018	0.012355	0.003030	1.000	2
length[384]	0.004221	0.000021	0.000021	0.012628	0.002767	0.999	2
length[385]	0.003961	0.000016	0.000017	0.012377	0.002851	1.001	2
length[386]	0.004120	0.000016	0.000004	0.011328	0.002685	0.998	2

length[387]	0.004188	0.000016	0.000020	0.012203	0.002742	1.012	2
length[388]	0.004362	0.000018	0.000029	0.012579	0.003029	0.998	2
length[389]	0.011568	0.000080	0.000046	0.028419	0.009834	0.998	2
length[390]	0.003854	0.000014	0.000001	0.011745	0.002725	0.999	2
length[391]	0.004343	0.000018	0.000006	0.013634	0.002917	0.999	2
length[392]	0.003808	0.000015	0.000001	0.011694	0.002623	1.000	2
length[393]	0.003774	0.000013	0.000008	0.011283	0.002746	0.998	2
length[394]	0.004055	0.000018	0.000013	0.012780	0.002875	0.998	2
length[395]	0.003971	0.000013	0.000012	0.011155	0.002763	0.998	2
length[396]	0.023681	0.000252	0.000014	0.053970	0.021723	1.009	2
length[397]	0.020194	0.000144	0.000184	0.043387	0.017889	1.004	2
length[398]	0.154208	0.010402	0.001352	0.328365	0.140167	0.999	2
length[399]	0.193948	0.013554	0.001794	0.413797	0.178772	1.001	2
length[400]	0.009440	0.000056	0.000147	0.022853	0.006960	1.002	2
length[401]	0.004201	0.000016	0.000022	0.012712	0.002987	0.998	2
length[402]	0.124438	0.003869	0.012112	0.233010	0.119195	0.998	2
length[403]	0.004900	0.000023	0.000002	0.013714	0.003397	1.001	2
length[404]	0.004866	0.000024	0.000011	0.013760	0.003631	1.000	2
length[405]	0.004724	0.000021	0.000003	0.013804	0.003469	1.018	2
length[406]	0.004035	0.000013	0.000023	0.011418	0.003041	0.998	2
length[407]	0.004256	0.000019	0.000002	0.012938	0.002991	0.999	2
length[408]	0.004658	0.000024	0.000002	0.013325	0.003206	0.998	2
length[409]	0.004812	0.000028	0.000008	0.016134	0.002951	1.006	2
length[410]	0.004890	0.000025	0.000001	0.014857	0.003528	0.998	2
length[411]	0.005094	0.000025	0.000007	0.014541	0.003535	1.002	2
length[412]	0.003935	0.000015	0.000010	0.012484	0.002838	0.999	2
length[413]	0.015317	0.000085	0.000382	0.033296	0.013827	1.007	2
length[414]	0.005119	0.000024	0.000028	0.015124	0.003527	1.000	2
length[415]	0.005068	0.000023	0.000000	0.014591	0.003435	0.998	2
length[416]	0.016759	0.000105	0.001915	0.037318	0.015236	1.007	2
length[417]	0.004144	0.000017	0.000018	0.012355	0.002838	0.998	2
length[418]	0.005428	0.000026	0.000039	0.015509	0.004151	1.020	2
length[419]	0.009142	0.000072	0.000011	0.026805	0.006377	1.017	2
length[420]	0.004291	0.000017	0.000007	0.012858	0.003146	0.999	2
length[421]	0.005582	0.000029	0.000081	0.018143	0.004070	1.000	2
length[422]	0.195271	0.012692	0.009928	0.403113	0.172987	1.013	2
length[423]	0.004508	0.000021	0.000008	0.013150	0.003205	0.998	2
length[424]	0.004935	0.000026	0.000000	0.014356	0.003444	1.001	2
length[425]	0.003995	0.000016	0.000007	0.011271	0.002797	1.001	2
length[426]	0.004035	0.000017	0.000017	0.012317	0.002668	1.006	2
length[427]	0.004808	0.000025	0.000032	0.016611	0.003343	0.998	2
length[428]	0.110369	0.002569	0.018765	0.209898	0.107755	1.009	2
length[429]	0.003970	0.000016	0.000002	0.011701	0.002625	0.997	2
length[430]	0.014181	0.000125	0.000337	0.035854	0.011897	0.999	2
length[431]	0.019344	0.000294	0.000068	0.052581	0.015623	0.999	2
length[432]	0.004469	0.000022	0.000010	0.015023	0.002878	1.004	2
length[433]	0.005461	0.000035	0.000006	0.017629	0.003691	0.997	2
length[434]	0.004276	0.000018	0.000017	0.013174	0.003122	0.997	2
length[435]	0.004351	0.000022	0.000013	0.013113	0.002811	1.005	2
length[436]	0.005099	0.000025	0.000007	0.014948	0.003596	0.998	2
length[437]	0.003555	0.000011	0.000005	0.009649	0.002601	0.997	2
length[438]	0.003746	0.000015	0.000026	0.011858	0.002594	1.019	2
length[439]	0.004884	0.000024	0.000009	0.015669	0.003201	1.002	2
length[440]	0.004623	0.000022	0.000019	0.013489	0.003028	0.997	2
length[441]	0.005459	0.000033	0.000012	0.016599	0.003735	1.008	2
length[442]	0.004635	0.000018	0.000031	0.012651	0.003186	0.998	2
length[443]	0.006253	0.000045	0.000019	0.018441	0.003937	0.998	2
length[444]	0.005123	0.000028	0.000016	0.016401	0.003416	0.997	2
length[445]	0.108275	0.006267	0.007105	0.285127	0.084360	1.014	2
length[446]	0.011120	0.000074	0.000032	0.026988	0.010060	0.999	2
length[447]	0.012678	0.000070	0.000729	0.028999	0.011143	0.997	2
length[448]	0.003928	0.000015	0.000005	0.010999	0.002986	1.005	2
length[449]	0.012954	0.000120	0.000014	0.035277	0.009114	1.000	2
length[450]	0.006012	0.000034	0.000001	0.015875	0.004128	1.019	2
length[451]	0.003858	0.000014	0.000008	0.012246	0.002803	1.000	2
length[452]	0.005887	0.000039	0.000029	0.021211	0.003417	1.020	2
length[453]	0.004375	0.000020	0.000003	0.012484	0.002987	1.002	2
length[454]	0.004365	0.000018	0.000008	0.012564	0.003295	0.997	2
length[455]	0.085286	0.001640	0.016877	0.157607	0.081810	0.996	2
length[456]	0.011204	0.000097	0.000012	0.029463	0.007819	1.012	2
length[457]	0.014391	0.000134	0.000481	0.040026	0.011070	0.995	2

\+ Convergence diagnostic (PSRF = Potential Scale Reduction Factor; Gelman and Rubin, 1992) should approach 1.0 as runs converge. NA is reported when deviation of parameter values within all runs is 0 or when a parameter value (a branch length, for instance) is not sampled in all runs.

Summary statistics for partitions with frequency >= 0.10 in at least one run:

Average standard deviation of split frequencies = 0.014065
Maximum standard deviation of split frequencies = 0.079143
Average PSRF for parameter values (excluding NA and >10.0) = 1.002
Maximum PSRF for parameter values = 1.084

Clade credibility values:
Subtree rooted at node 232:

```
                                               /----- 30_CrmB_MPXV_SL_176 (120)
                                               |
                                               |----- 31_CrmB_MPXV_SL_002 (130)
                     /-----------100-----------+
                     |                         |----- 32_CrmB_MPXV_Z96_J2R (140)
                     |                         |
                     |                         \----- 33_CrmB_MPXV_Z96_J2L (150)
                     |
                     |                         /----- 40_CrmB_CMLV_M96_CrmB (160)
                     |         /------100------+
                     |         |               \----- 41_CrmB_CMLV_M96a_CrmB (170)
                     |         |
                     |         |               /----- 42_CrmB_HSPV_205 (110)
                /-100+    /-100+     /---100---+
                |    |    |    |     |         \----- 43_CrmB_HSPV_003 (111)
                |    |    |    |     |
                |    |    |    |     |         /----- 36_CrmB_VARV_GUI69_205 (114)
                |    |    |    \--94-+    /-100+
                |    |    |          |    |    \----- 38_CrmB_VARV_SLN68_205 (115)
                |    |    |          |    |
                |    |    |          |    |---------- 34_CrmB_VARV_GAR_G2R (116)
                |    |    |          \-100+
                |    |    |               |---------- 37_CrmB_VARV_BRZ66_205 (117)
                |    \-96-+               |
                |         |               |    /----- 35_CrmB_VARV_IND64_205 (118)
                |         |               \-100+
                |         |                    \----- 39_CrmB_VARV_CNG70_205 (119)
                |         |
           /-73-+         |                    /----- 44_CrmB_CPXV_GRI_D2L (180)
           |    |         |          /----93---+
           |    |         |          |         \----- 45_CrmB_CPXV_GRI_I4R (190)
           |    |         |          |
           |    |         \----93----+         /----- 48_CrmB_CPXV_GER91_216 (112)
           |    |                    |    /-100+
           |    |                    |    |    \----- 49_CrmB_CPXV_GER91_002 (113)
           |    |                    \-99-+
           |    |                         |    /----- 46_CrmB_CPXV_BRI_230 (120)
           |    |                         \-100+
           |    |                              \----- 47_CrmB_CPXV_BRI_005 (121)
      /-100+    |
      |    |    |                         /---------- 52_CrmD_CPXV_GRI_K2R (122)
      |    |    |                         |
      |    |    |                         |---------- 53_CrmD_CPXV_BRI_221 (123)
      |    |    \-----------100-----------+
      |    |                              |    /----- 54_CrmD_ECTV_MOS_I1R (124)
      |    |                              \-79-+
      |    |                                   \----- 55_CrmD_ECTV_MOS_003 (125)
      |    |
/--96-+    |                                   /----- 50_CrmB_MYXV_LAU_MT2 (130)
|     |    \----------------100----------------+
|     |                                        \----- 51_CrmB_MYXV_LAU_m002L (131)
```

```
          |     |
          |     |                                        /----- 93_SCP2_CPXV_GRI_D12L (126)
          |     |                                   /-96-+
     /-100+     |                                   |    \----- 95_SCP2_CPXV_GER_12 (127)
     |    |     |                              /-100+
     |    |     |                              |    \---------- 94_SCP2_ECTV_MOS_008 (129)
     |    |     \--------------100-------------+
--62-+    |                                    \--------------- 96_SCP2_CPXV_BRI_014 (128)
     |    |
     |    \---------------------------------------------------- 97_SCP2_COTV168 (132)
     |
     \--------------------------------------------------------- 56_DPXV_W83_gp016 (156)
```

Subtree rooted at node 264:

```
                          /------------------------------------ 1_M2_CPXV_BRI_040 (133)
                          |
                          |    /------------------------------- 2_M2_CPXV_GER91_038 (134)
                          |    |
                          |    |               /--------------- 61_M2_VARV_IND64_022 (135)
                          |    |               |
                     /-100+    |               |--------------- 64_M2_VARV_CNG70_022 (136)
                     |    |    |               |
                     |    |    |         /-100-+         /----- 63_M2_VARV_SLN68_22 (137)
                     |    |    |         |     |    /-100+
                     |    |    |         |     |    |    \----- 71_M2_VARV_GUI69_22 (138)
                     |    \-51-+         |     |    |
                     |         |         |     \-99-+---------- 69_M2_VARV_BRZ66_26 (139)
                     |         |    /-100+          |
                     |         |    |    |          \---------- 70_M2_VARV_GAR_Q2L (140)
                     |         |    |    |
                     |         |    |    |--------------------- 65_M2_TATV_DAH68_033 (141)
                /-100+         |    |    |
                |    |         |    |    \--------------------- 62_M2_CMLV_M96_029 (142)
                |    |         \-100+
                |    |              |                    /----- 66_M2_MPXVSL21 (143)
                |    |              |               /-100+
                |    |              |               |    \----- 67_M2_MPXV_Z96_O2L (144)
                |    |              |               |
          /-100-+    |              \------100------+---------- 68_M2_HSPV_034 (145)
          |     |    |                              |
          |     |    |                              \---------- 72_M2_VACV_WR_M2L (146)
          |     |    |
     /-100+     |    \----------------------------------------- 60_M2_COTV028 (147)
     |    |     |
     |    |     \---------------------------------------------- 73_M2_YKV_170 (148)
     |    |
--100+    \---------------------------------------------------- 74_M2_MYXV_LAU_m154L (149)
     |
     |                                                   /----- 58_M2like_YMTV_YLD_9L (150)
     \------------------------100------------------------+
                                                         \----- 59_M2like_DPXV_W83_17 (151)
```

Subtree rooted at node 269:

```
                                                         /----- 145_vCCI_MYXV_LAU_MT1 (27)
     /------------------------100------------------------+
     |                                                   \----- 146_vCCI_MYXV_LAU_m001L (28)
     |
     |                                                   /----- 3_A41like_COTV011 (29)
     |                         /-----------100-----------+
     |                         |                         \----- 4_A41like_COTV175 (30)
     |                         |
     |                         |                         /----- 5_A41_MPXV_SL_145 (31)
     |                         |                    /-99-+
     |                         |                    |    \----- 6_A41_MPXV_Z96_A41L (32)
     |                         |               /-91-+
     |                         |               |    |    /----- 17_A41_CPXV_BRI_178 (33)
```

```
     |                        |               |    \-99-+
     |                        |               |         \----- 18_A41_CPXV_GER91_166 (34)
     |                        |               |
     |                        |               |--------------- 16_A41_CPXV_GRI_A43L (42)
     |                        |         /--88-+
     |                        |         |     |         /----- 15_A41_HSPV_163 (43)
     |    /---------99--------+         |     |----99---+
     |    |                   |         |     |         \----- 20_A41_VACV_WR_A41 (44)
     |    |                   |         |     |
     |    |                   |         |     \--------------- 19_A41_ECTV_MOS_140 (46)
     |    |                   |         |
     |    |                   |         |               /---- 7_A41_VARV_GAR_A49L (35)
     |    |                   |         |          /-100+
     |    |                   |         |          |    \----- 10_A41_VARV_BRZ66_157 (36)
     |    |                   |    /-62-+          |
     |    |                   |    |    |          |---------- 11_A41_VARV_IND64_158 (37)
     |    |                   |    |    |          |
     |    |                   |    |    |----82----+---------- 12_A41_VARV_SLN68_157 (38)
     |    |                   |    |    |          |
     |    |                   |    |    |          |---------- 9_A41_VARV_CNG70_157 (39)
--100+    |                   \-100+    |          |
     |    |                        |    |          \---------- 8_A41_VARV_GUI69_157 (40)
     |    |                        |    |
     |    |                        |    \--------------------- 13_A41_TATV_DAH68_A43L (41)
     |    |                        |
     |    |                        \-------------------------- 14_A41_CMLV_M96_160 (45)
     |    |
     |    |                                             /----- 80_GIF_ORFVgORF117 (47)
     |    |                                        /-100+
     |    |                                        |    \----- 82_GIF_PCPV_gp121 (48)
     |    |                                   /-88-+
     |    |                                   |    \---------- 21_BPSVgORF117 (49)
     |    |                             /-100-+
     |    |                             |     \--------------- 81_GIF_SB87_117 (50)
     |    |                             |
     |    |                             |               /----- 75_CBP_ORFVgORF112 (51)
     |    |                             |          /-100+
     |    |                             |          |    \----- 79_CPB_PCPV_gp116 (52)
     |    |                             |          |
     |    |-------------100-------------+----72----+---------- 76_CBP_BPSVgORF112 (53)
     |    |                             |          |
     |    |                             |          |    /----- 77_CPB_SB87_gp112 (54)
     |    |                             |          \-100+
     |    |                             |               \----- 78_CPB_SB87_gp111 (55)
     |    |                             |
     |    |                             \--------------------- 83_SB87_gp113 (152)
     |    |
     |    |                                             /----- 109_T4like_MPXV_Z96_B10R (56)
     |    |                             /------100------+
     |    |                             |               \----- 112_T4like_CPXV_GRI_B8R (57)
     |    |                             |
     |    |                             |               /----- 110_T4like_CMLV_M96_MT4like (58)
     \-97-+                        /-99-+          /-99-+
          |                        |    |          |    \----- 111_T4like_TATV_DAH68_195 (59)
          |                        |    |     /-63-+
          |                        |    |     |    \---------- 113_T4like_CPXV_BRI_203 (60)
          |                   /-100+    \-100-+
          |                   |    |          \--------------- 114_T4like_CPXV_GER91_191 (61)
          |                   |    |
          |                   |    |                    /----- 115_T4like_YKV002c (62)
          |                   |    \---------100--------+
          |                   |                         \----- 116_T4like_YKV185 (63)
          |                   |
          |     /------100-----+                         /----- 117_T4_MYXV_LAU_MT4 (64)
          |     |             |         /------100------+
          |     |             |         |               \----- 118_T4_MYXV_LAU_m004L (65)
          |     |             |         |
          |     |             |         |               /----- 119_T4_DPXV_W83_004 (66)
          |     |             |         |     /---100---+
          |     |             \----52---+     |         \----- 120_T4_DPXV_W83_166 (67)
          |     |                       |     |
          |     |                       |     |    /---------- 121_T4_LSDV_2490_003 (68)
```

```
|     |                        |     |    |
|     |                        \--63-+    |---------- 122_T4_LSDV_2490_154 (69)
|     |                              |    |
|     |                              |    |    /----- 123_T4_SPPV_A_002 (70)
|     |                              |    |-85-+
|     |                              \-99-+    \----- 124_T4_SPPV_A_129 (71)
|     |                                   |
|     |                                   |    /----- 125_T4_GTPV_G20_002 (72)
|     |                                   |    |
|     |                                   |    |----- 126_T4_GTPV_G20_130 (73)
|     |                                   \-81-+
|     |                                        |----- 127_T4_GTP_PEL_002 (74)
|     |                                        |
|     |                                        \----- 128_T4_GTP_PEL_146 (75)
|     |
|     |                                        /----- 24_C8_VACV_WR_C8L (76)
|     |                                   /-87-+
|     |                                   |    \----- 25_C8_HSPV9_023 (77)
|     |                              /-59-+
|     |                              |    \---------- 22_C8_CPXV_BRI_028 (78)
|     |                              |
|     |                        /-100-+         /----- 23_C8_CMLV_M96_18 (79)
|     |                        |     |    /-77-+
|     |                        |     |    |    \----- 27_C8_CPXV_GRI_C12L (81)
|     |         /------51------+     \-60-+
|     |         |              |          \---------- 26_C8_CPXV_GER91_026 (80)
\--96-+         |              |
      |         |              \--------------------- (232)
      |         |
      |         |                              /----- 98_SCP3_MPXVSL161 (82)
      |         |              /--------85-----+
      |         |              |               \----- 99_SCP3_MPXV_Z96_B8R (83)
      |         |              |
      |         |         /-57-+     /--------------- 105_SCP3_ECTV_MOS_C3R (87)
      |         |         |    |     |
      |         |         |    \--89-+    /---------- 100_SCP3_TATV_DAH68_190 (88)
      |         |         |          |    |
      |         |         |          \-99-+    /----- 104_SCP3_CPXV_GER91_188 (89)
      |         |         |               \-74-+
      |         |---100---+                    \----- 103_SCP3_CPXV_BRI_201 (90)
      |         |         |
      |         |         |                    /----- 101_SCP3_HSPV184 (84)
      |         |         |               /-80-+
      |         |         |               |    \----- 106_SCP3_VACV_WR_B7R (85)
      |         |         \--------51-----+
      |         |                         \---------- 102_SCP3_CPXV_GRI_B6R (86)
      |         |
      |         |                              /----- 84_SCP1_MPXV_SL_170 (91)
      |    /-99-+              /--------97-----+
      |    |    |              |               \----- 85_SCP1_MPXV_Z96_B20R (92)
      |    |    |              |
      |    |    |              |--------------------- 90_SCP1_CPXV_GRI_B21R (93)
      |    |    |         /-70-+
      |    |    |         |    |     /--------------- 86_SCP1_CPXV_GER91_204 (95)
      |    |    |         |    |     |
      |    |    |         |    \--88-+    /---------- 88_SCP1_CPXV_BRI_218 (97)
      |    |    |         |          |    |
      |    |    |         |          \-95-+    /----- 89_SCP1_TATV_DAH68_218 (98)
      |    |    |    /-100+               \-52-+
      |    |    |    |    |                    \----- 92_SCP1_CMLV_M96_206 (99)
      |    |    |    |    |
      \-98-+    |    |    |-------------------------- 87_SCP1_VACV_WR_C13L (94)
           |    |-64-+    |
           |    |    |    \-------------------------- 91_SCP1_HSPV199 (96)
           |    |    |
           |    |    |                         /----- 107_SCPlike_COTV179 (100)
           |    |    \-----------100-----------+
           |    |                              \----- 108_SCPlike_COTV007 (110)
           |    |
           |    |------------------------------------ 29_CPXV_GER91_3_007 (153)
           |    |
           |    \------------------------------------ 28_COTV030 (155)
           |
```

```
                        |
                        |                                   /----- (264)
                        \-----------------99----------------+
                                                            \----- 57_M2like_YKV175 (154)
```

Root part of tree:

```
   /-------------------------------------------------------------- 139_vCCI_VARV_GAR_G3R (1)
   |
   |-------------------------------------------------------------- 140_vCCI_VARV_CNG70_206 (2)
   |
   |-------------------------------------------------------------- 129_vCCI_VARV_BRZ66_206 (3)
   |
   |-------------------------------------------------------------- 156_vCCI_VARV_IND64_206 (6)
   |
   |                                                     /-------- 130_vCCI_VARV_SLN68_206 (4)
   |-------------------------100-------------------------+
   |                                                     \-------- 155_vCCI_VARV_GUI69_206 (5)
   |
   |                                                     /-------- 143_vCCI_CMLV_M96_211 (7)
   |       /---------------------100---------------------+
   +       |                                             \-------- 144_vCCI_CMLV_M96_1 (8)
   |       |                                             /-------- 133_vCCI_CPXV_GRI_D1L (9)
   |       |              /--------------86--------------+
   |       |              |                              \-------- 141_vCCI_CPXV_GRI_I5R (10)
   |       |              |                              /-------- 134_vCCI_VACV_WR_B29R (11)
   |       |              |--------------98--------------+
   |       |              |                              \-------- 135_vCCI_VACV_WR_C23L (12)
   |       |              |--------------------------------------- 142_vCCI_RPXV_Utrecht_35kDa (13)
   |       |              |                              /-------- 131_vCCI_ECTV_MOS_K2R (15)
   \--100--+              |       /----------100---------+
           |       /--67--+       |                      \-------- 132_vCCI_ECTV_MOS_001 (16)
           |       |      |       |                      /-------- 149_vCCI_CPXV_BRI_003 (17)
           |       |      |       |               /--99--+
           |       |      |       |               |      \-------- 152_vCCI_CPXV_BRI_227 (18)
           |       |      |       |       /--100--+
           |       |      |       |       |       |      /-------- 153_vCCI_CPXV_GER91_001 (19)
           |       |      |       |       |       \--100-+
           |       |      |       |       |              \-------- 154_vCCI_CPXV_GER91_217 (20)
           |       |      |       |       |
           |       |      |       |---59--+              /-------- 137_vCCI_HSPV002 (21)
           |       |      \--100--+       |       /--100-+
           \--100--+              |       |       |      \-------- 138_vCCI_HSPV206 (22)
                   |              |       |       |
                   |              |       \--100--+      /-------- 150_vCCI_MPXV_Z96_J3R (23)
                   |              |               |      |
                   |              |               |      |-------- 151_vCCI_MPXVSL_001 (24)
                   |              |               \--100-+
                   |              |                      |-------- 147_vCCI_MPXVSL_177 (25)
                   |              |                      |
                   |              |                      \-------- 148_vCCI_MPXV_Z96_J1L (26)
                   |              |
                   |              \------------------------------- (269)
                   |
                   \---------------------------------------------- 136_vCCI_VACV_Lister_vCCI (14)
```

```
Phylogram (based on average branch lengths):

/ 139_vCCI_VARV_GAR_G3R (1)
|
| 140_vCCI_VARV_CNG70_206 (2)
|
| 129_vCCI_VARV_BRZ66_206 (3)
|
| 156_vCCI_VARV_IND64_206 (6)
|
| 130_vCCI_VARV_SLN68_206 (4)
|
| 155_vCCI_VARV_GUI69_206 (5)
|
|/ 143_vCCI_CMLV_M96_211 (7)
||
|| 144_vCCI_CMLV_M96_1 (8)
||
|| 133_vCCI_CPXV_GRI_D1L (9)
||
|| 141_vCCI_CPXV_GRI_I5R (10)
||
|| 134_vCCI_VACV_WR_B29R (11)
||
|| 135_vCCI_VACV_WR_C23L (12)
||
|| 142_vCCI_RPXV_Utrecht_35kDa (13)
||
|| / 131_vCCI_ECTV_MOS_K2R (15)
||/+
|||\ 132_vCCI_ECTV_MOS_001 (16)
|||
||| / 149_vCCI_CPXV_BRI_003 (17)
|||/+
||||\ 152_vCCI_CPXV_BRI_227 (18)
|||+
||||/ 153_vCCI_CPXV_GER91_001 (19)
|||\+
||| \ 154_vCCI_CPXV_GER91_217 (20)
|||
||| / 137_vCCI_HSPV002 (21)
|||/+
||||\ 138_vCCI_HSPV206 (22)
||||
|||+/ 150_vCCI_MPXV_Z96_J3R (23)
|||||
||||| 151_vCCI_MPXVSL_001 (24)
|||\+
||| | 147_vCCI_MPXVSL_177 (25)
+|| |
||| \ 148_vCCI_MPXV_Z96_J1L (26)
|||
|||             / 145_vCCI_MYXV_LAU_MT1 (27)
|||     /-------+
|||     |       \ 146_vCCI_MYXV_LAU_m001L (28)
```

```
|||     |
|||     |                      / 3_A41like_COTV011 (29)
|||     |           /----------+
|||     |           |          \ 4_A41like_COTV175 (30)
|||     |           |
|||     |           |              / 5_A41_MPXV_SL_145 (31)
|||     |           |             /+
||+     |           |             |\ 6_A41_MPXV_Z96_A41L (32)
|||     |           |             |
|||     |           |             |/ 17_A41_CPXV_BRI_178 (33)
|||     |           |             |+
|||     |           |             |\ 18_A41_CPXV_GER91_166 (34)
|||     |           |             |
|||     |           |             | 16_A41_CPXV_GRI_A43L (42)
|||     |           |             |
|||     |           |             | 15_A41_HSPV_163 (43)
|||     |     /-----+             |
|||     |     |     |             | 20_A41_VACV_WR_A41 (44)
|||     |     |     |             |
|||     |     |     |             |- 19_A41_ECTV_MOS_140 (46)
|||     |     |     |             |
|||     |     |     |             | 7_A41_VARV_GAR_A49L (35)
|||     |     |     |             |
|||     |     |     |             | 10_A41_VARV_BRZ66_157 (36)
|||     |     |     |            /+
|||     |     |     |            || 11_A41_VARV_IND64_158 (37)
|||     |     |     |            ||
|||     |     |     |            || 12_A41_VARV_SLN68_157 (38)
|||     |     |     |            ||
|||     |     |     |            || 9_A41_VARV_CNG70_157 (39)
|||     |     |     \------------+|
|||     |     |                  || 8_A41_VARV_GUI69_157 (40)
|||     |     |                  ||
|||     |     |                  |\ 13_A41_TATV_DAH68_A43L (41)
|||     |     |                  |
|||     |     |                  \- 14_A41_CMLV_M96_160 (45)
|||     |     |
|||     |     |                                 / 80_GIF_ORFVgORF117 (47)
|||     |     |                         /-------+
||\-----+     |                         |       \-- 82_GIF_PCPV_gp121 (48)
||      |     |                      /--+
\+      |     |                      |  \------- 21_BPSVgORF117 (49)
 |      |     |            /---------+
 |      |     |            |         \------ 81_GIF_SB87_117 (50)
 |      |     |            |
 |      |     |            |      /----- 75_CBP_ORFVgORF112 (51)
 |      |     |            |  /---+
 |      |     |            |  |   \---- 79_CPB_PCPV_gp116 (52)
 |      |     |            |  |
 |      |     |------------+--+------ 76_CBP_BPSVgORF112 (53)
 |      |     |            |  |
 |      |     |            |  |   /------ 77_CPB_SB87_gp112 (54)
 |      |     |            |  \---+
 |      |     |            |      \------ 78_CPB_SB87_gp111 (55)
 |      |     |            |
 |      |     |            \---------- 83_SB87_gp113 (152)
 |      |     |
 |      |     |                                /- 109_T4like_MPXV_Z96_B10R (56)
 |      |     |                           /----+
 |      |     |                           |    \ 112_T4like_CPXV_GRI_B8R (57)
 |      |     |                           |
 |      |     |                           |/- 110_T4like_CMLV_M96_MT4like (58)
 |      |     |                        /--+|
 |      |     |                        |  ||- 111_T4like_TATV_DAH68_195 (59)
 |      |     |                        |  ||
 |      |     |                        |  || 113_T4like_CPXV_BRI_203 (60)
 |      |     |                 /------+  \+
 |      |     |                 |      |   \ 114_T4like_CPXV_GER91_191 (61)
 |      |     |                 |      |
 |      |     |                 |      |   / 115_T4like_YKV002c (62)
 |      |     |                 |      \---+
 |      |     |                 |          \ 116_T4like_YKV185 (63)
```

```
|      |     |                 |
|      |     |    /------------+            / 117_T4_MYXV_LAU_MT4 (64)
|      |     |    |            | /----------+
|      |     |    |            | |          \ 118_T4_MYXV_LAU_m004L (65)
|      |     |    |            | |
|      |     |    |            | |         / 119_T4_DPXV_W83_004 (66)
|      |     |    |            | |  /------+
|      |     |    |            \-+  |      \ 120_T4_DPXV_W83_166 (67)
|      \-----+    |              |  |
|            |    |              |  |   / 121_T4_LSDV_2490_003 (68)
|            |    |              |  |   |
|            |    |              \--+   | 122_T4_LSDV_2490_154 (69)
|            |    |                 |   |
|            |    |                 |   |/ 123_T4_SPPV_A_002 (70)
|            |    |                 |   |+
|            |    |                 \---+\ 124_T4_SPPV_A_129 (71)
|            |    |                     |
|            |    |                     |/ 125_T4_GTPV_G20_002 (72)
|            |    |                     ||
|            |    |                     || 126_T4_GTPV_G20_130 (73)
|            |    |                     \+
|            |    |                      | 127_T4_GTP_PEL_002 (74)
|            |    |                      |
|            |    |                      \ 128_T4_GTP_PEL_146 (75)
|            |    |
|            |    |                                  / 24_C8_VACV_WR_C8L (76)
|            |    |                                  |
|            |    |                                  |- 25_C8_HSPV9_023 (77)
|            |    |                                  |
|            |    |                                  | 22_C8_CPXV_BRI_028 (78)
|            |    |                                  |
|            |    |                  /---------------+- 23_C8_CMLV_M96_18 (79)
|            |    |                  |               |
|            |    |                  |               |- 27_C8_CPXV_GRI_C12L (81)
|            |    |                  |               |
|            |    |                  |               \ 26_C8_CPXV_GER91_026 (80)
|            |    |                  |
|            |    |                  |                     / 30_CrmB_MPXV_SL_176 (120)
|            |    |                  |                     |
|            |    |                  |                     | 31_CrmB_MPXV_SL_002 (130)
|            |    |                  |                     |
|            |    |                  |                     | 32_CrmB_MPXV_Z96_J2R (140)
|            |    |                  |                     |
|            |    |                  |                     | 33_CrmB_MPXV_Z96_J2L (150)
|            |    |                  |                     |
|            |    |                  |                     |/ 40_CrmB_CMLV_M96_CrmB (160)
|            |    |                  |                     |+
|            |    |                  |                     |\ 41_CrmB_CMLV_M96a_CrmB (170)
|            |    |                  |                     |
|            |    |                  |                     |/ 42_CrmB_HSPV_205 (110)
|            |    |                  |                 /---+|
|            |    |                  |                 |   || 43_CrmB_HSPV_003 (111)
|            |    |                  |                 |   ||
|            |    |                  |                 |   ||/ 36_CrmB_VARV_GUI69_205 (114)
|            |    |                  |                 |   |+|
|            |    |                  |                 |   ||| 38_CrmB_VARV_SLN68_205 (115)
|            |    |                  |                 |   |||
|            |    |                  |                 |   ||| 34_CrmB_VARV_GAR_G2R (116)
|            |    |                  |                 |   |\+
|            |    |                  |                 |   | | 37_CrmB_VARV_BRZ66_205 (117)
|            |    |                  |                 |   | |
|            |    |                  |                 |   | | 35_CrmB_VARV_IND64_205 (118)
|            |    |                  |                 |   | |
|            |    |             /----+                 |   | \ 39_CrmB_VARV_CNG70_205 (119)
|            |    |             |    |                 |   |
|            |    |             |    |               /-+   |- 44_CrmB_CPXV_GRI_D2L (180)
|            |    |             |    |               | |   |
|            |    |             |    |               | |   |- 45_CrmB_CPXV_GRI_I4R (190)
|            |    |             |    |               | |   |
|            |    |             |    |               | |   |/ 48_CrmB_CPXV_GER91_216 (112)
|            |    |             |    |               | |   ||
|            |    |             |    |               | |   || 49_CrmB_CPXV_GER91_002 (113)
```

```
|            \----+             |    |              | |   \+
|                 |             |    |              | |    |/ 46_CrmB_CPXV_BRI_230 (120)
|                 |             |    |              | |    \+
|                 |             |    |              | |     \ 47_CrmB_CPXV_BRI_005 (121)
|                 |             |    |            /-+ |
|                 |             |    |            | | |      / 52_CrmD_CPXV_GRI_K2R (122)
|                 |             |    |            | | |      |
|                 |             |    |            | | |      | 53_CrmD_CPXV_BRI_221 (123)
|                 |             |    |            | | \------+
|                 |             |    |            | |        | 54_CrmD_ECTV_MOS_I1R (124)
|                 |             |    |            | |        |
|                 |             |    |            | |        \ 55_CrmD_ECTV_MOS_003 (125)
|                 |             |    |            | |
|                 |             |    |         /--+ |       / 50_CrmB_MYXV_LAU_MT2 (130)
|                 |             |    |         |  | \-------+
|                 |             |    |         |  |         \ 51_CrmB_MYXV_LAU_m002L (131)
|                 |             |    |         |  |
|                 |             |    |         |  |     / 93_SCP2_CPXV_GRI_D12L (126)
|                 |             |    |         |  |    /+
|                 |             |    |    /----+  |    |\ 95_SCP2_CPXV_GER_12 (127)
|                 |             |    |    |    |  |    |
|                 |             |    |    |    |  |    |- 94_SCP2_ECTV_MOS_008 (129)
|                 |             |    |    |    |  \----+
|                 |             |    \----+    |       \ 96_SCP2_CPXV_BRI_014 (128)
|                 |             |         |    |
|                 |             |         |    \------ 97_SCP2_COTV168 (132)
|                 |             |         |
|                 |             |         \------------- 56_DPXV_W83_gp016 (156)
|                 |             |
|                 |             |               / 98_SCP3_MPXVSL161 (82)
|                 |             |               |
|                 |      /------+               | 99_SCP3_MPXV_Z96_B8R (83)
|                 |      |      |               |
|                 |      |      |               |- 105_SCP3_ECTV_MOS_C3R (87)
|                 |      |      |               |
|                 |      |      |               |/ 100_SCP3_TATV_DAH68_190 (88)
|                 |      |      |               ||
|                 |      |      |               |+ 104_SCP3_CPXV_GER91_188 (89)
|                 |      |      |               ||
|                 |      |      |---------------+\- 103_SCP3_CPXV_BRI_201 (90)
|                 |      |      |               |
|                 |      |      |               | 101_SCP3_HSPV184 (84)
|                 |      |      |               |
|                 |      |      |               | 106_SCP3_VACV_WR_B7R (85)
|                 |      |      |               |
|                 |      |      |               \ 102_SCP3_CPXV_GRI_B6R (86)
|                 |      |      |
|                 |      |      |               / 84_SCP1_MPXV_SL_170 (91)
|                 |      |      |               |
|                 |      |      |               | 85_SCP1_MPXV_Z96_B20R (92)
|                 |      |      |               |
|                 |      |      |               | 90_SCP1_CPXV_GRI_B21R (93)
|                 |      |      |              /+
|                 |      |      |              ||- 86_SCP1_CPXV_GER91_204 (95)
|                 |      |      |              ||
|                 |      |      |              ||/- 88_SCP1_CPXV_BRI_218 (97)
|                 |      |      |              |||
|                 |      |      |              |\+- 89_SCP1_TATV_DAH68_218 (98)
|                 |      |      |   /----------+ |
|                 |      |      |   |          | \- 92_SCP1_CMLV_M96_206 (99)
|                 |      |      |   |          |
|                 |      |      |   |          | 87_SCP1_VACV_WR_C13L (94)
|                 |      |      |---+          |
|                 |      |      |   |          \- 91_SCP1_HSPV199 (96)
|                 |      |      |   |
|                 |      |      |   |        / 107_SCPlike_COTV179 (100)
|                 |      |      |   \--------+
|                 |      |      |            \ 108_SCPlike_COTV007 (110)
|                 |      |      |
|                 |      |      |-------------- 29_CPXV_GER91_3_007 (153)
|                 \------+      |
|                        |      \--------------- 28_COTV030 (155)
```

```
   |                       |
   |                       |                      / 1_M2_CPXV_BRI_040 (133)
   |                       |                      |
   |                       |                      | 2_M2_CPXV_GER91_038 (134)
   |                       |                      |
   |                       |                      |/ 61_M2_VARV_IND64_022 (135)
   |                       |                      ||
   |                       |                    /-+| 64_M2_VARV_CNG70_022 (136)
   |                       |                    | ||
   |                       |                    | |+ 63_M2_VARV_SLN68_22 (137)
   |                       |                    | ||
   |                       |                    | || 71_M2_VARV_GUI69_22 (138)
   |                       |                    | ||
   |                       |                    | || 69_M2_VARV_BRZ66_26 (139)
   |                       |                    | ||
   |                       |                    | |\ 70_M2_VARV_GAR_Q2L (140)
   |                       |                    | |
   |                       |                    | |- 65_M2_TATV_DAH68_033 (141)
   |                       |                   /+ |
   |                       |                   || |- 62_M2_CMLV_M96_029 (142)
   |                       |                   || |
   |                       |                   || |/ 66_M2_MPXVSL21 (143)
   |                       |                   || |+
   |                       |                   || |\ 67_M2_MPXV_Z96_O2L (144)
   |                       |                   || |
   |                       |                /--+| | 68_M2_HSPV_034 (145)
   |                       |                |  || |
   |                       |                |  || \ 72_M2_VACV_WR_M2L (146)
   |                       |                |  ||
   |                       |         /------+  |\---- 60_M2_COTV028 (147)
   |                       |         |      |  |
   |                       |         |      |  \----- 73_M2_YKV_170 (148)
   |                       |         |      |
   |                       |    /----+      \---- 74_M2_MYXV_LAU_m154L (149)
   |                       |    |    |
   |                       |    |    |     /------- 58_M2like_YMTV_YLD_9L (150)
   |                       \------+    \-----+
   |                            |          \------- 59_M2like_DPXV_W83_17 (151)
   |                            |
   |                            \------- 57_M2like_YKV175 (154)
   |
   \ 136_vCCI_VACV_Lister_vCCI (14)

   |--------------| 1.000 expected changes per site

   Calculating tree probabilities...

   Credible sets of trees (3002 trees sampled):
      50 % credible set contains 1501 trees
      90 % credible set contains 2702 trees
      95 % credible set contains 2852 trees
      99 % credible set contains 2972 trees


MrBayes > quit
```
