# Supplementary material for: Structural Conservation and Functional Diversity of the Poxvirus Immune Evasion (PIE) Domain Superfamily
Source: Viruses. 2015 Aug 28;7(9):4873–93. doi: 10.3390/v7092848 (PMC4584292; doi:10.3390/v7092848)
Supplement: Supplementary File 1 [file viruses-07-02848-s001.zip › Nelson_Supplements/Figure_S2_A41.pdf]

A41 Family Alignment

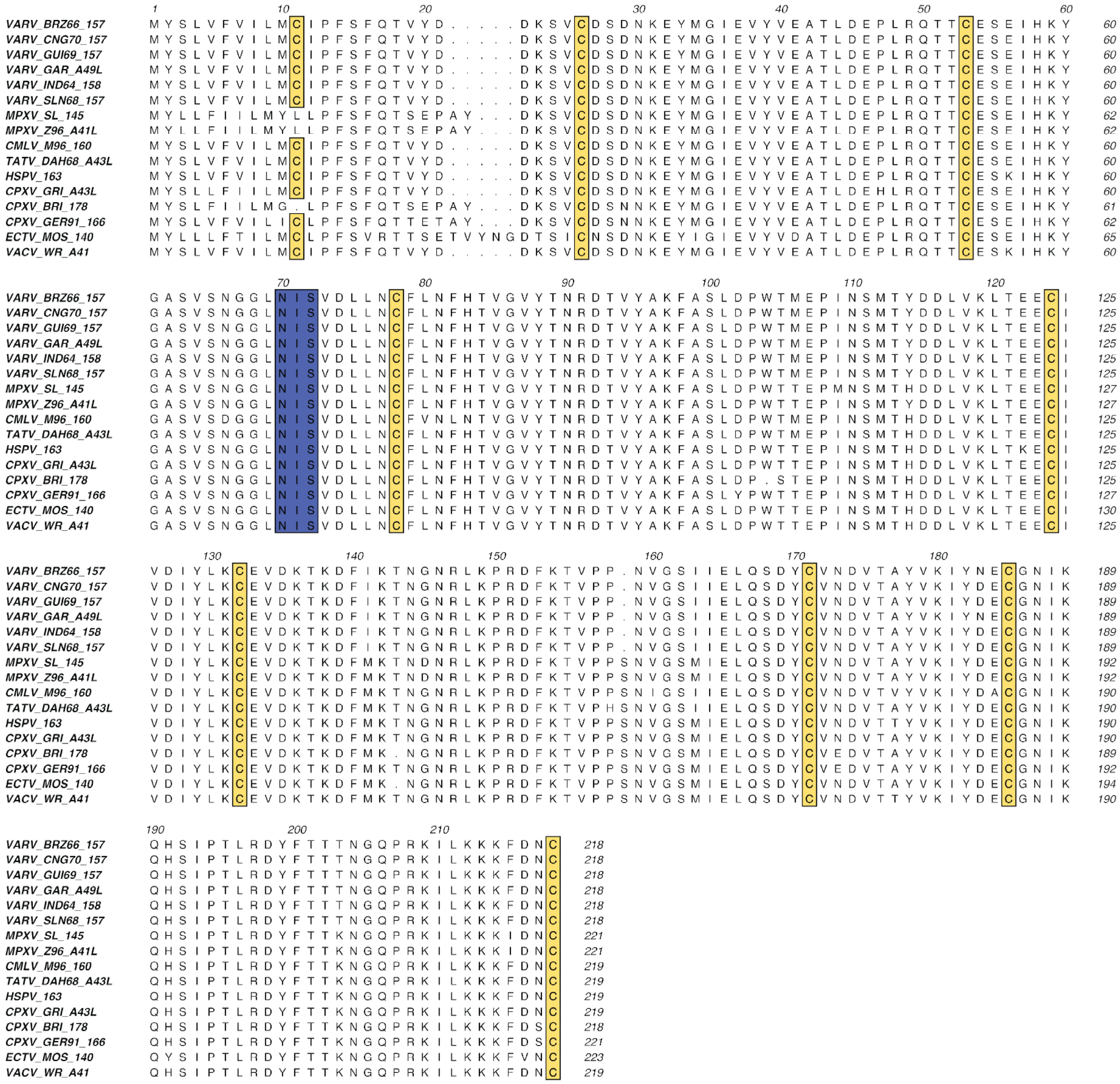

Figure S2. Sequence alignment of the A41 family members. Cysteine residues are boxed in yellow Predicted N-linked glycosylation sites are boxed in blue.
