# Supplementary material for: Structural Conservation and Functional Diversity of the Poxvirus Immune Evasion (PIE) Domain Superfamily
Source: Viruses. 2015 Aug 28;7(9):4873–93. doi: 10.3390/v7092848 (PMC4584292; doi:10.3390/v7092848)
Supplement: Supplementary File 1 [file viruses-07-02848-s001.zip › Nelson_Supplements/Figure_S6+S7_SCP-2&-3.pdf]

SCP-2 Family Alignment

```
                     1        10        20        30        40
SCP-2_CPXV_GRI_D12L  MINININTILIFASLFVASFANDYPPPGFFEDKYITDTFNYISLDFELYPVNVSSCNRLSTKQS.  64
SCP-2_CPXV_BRI_014   MINININTILIFASLFVASFANDYPPPGFFEDKYITNTFNYISIDFELYPVNVSSCNRLSTKQS.  64
SCP-2_CPXV_GER_12    MINININTILIFASLFVASFANDYPPPGFFEDKYITDTFNYISLDFELYPVNVSSCNRLSTKQS.  64
SCP-2_ECTV_MOS_008   MINININTILIFASLFVASFANDYPPPGFFENKYITDTFNYISIDFELYPVNVSSCNRLSTKQS.  64
SCP-2_SCP-2_COTV168  .......MIKIFFMFVMCQLSLIYP..YEYEDKYISNTFNYISIDIEMSNVNKTSCTKEINDNGK  56

                     50        60        70        80        90        100        110
SCP-2_CPXV_GRI_D12L  SDVITTSELTITVNSTDCDPVFVTEYYSVKGKTAIAGLFTDTTKKQNTSKMCTLNIEVKCNAETE  129
SCP-2_CPXV_BRI_014   SDVISTSELTITVNSTDCDPVFVTEYYSVKDKTAIAGLFTDTTKKQNTSKMCTLNIEVKCNAETE  129
SCP-2_CPXV_GER_12    SDVITTSELTITVNSTDCDPVFVTEYYSVKGKTAIAGLFTDTTKKQNTSKTCTLNIEVKCNSETE  129
SCP-2_ECTV_MOS_008   SDIITTSELTITVNSTDCDPVFVTEYYSIKDKTAVAGLFTDTTKKQNTSKMCTLNVEVKCNAETE  129
SCP-2_SCP-2_COTV168  SQIINTSELSITLNITDCDPKFTTGYYSTNNKNSMAGFFTSGDTFQDKSKKCKLSMEIKCIGKDV  121

                     120        130        140        150
SCP-2_CPXV_GRI_D12L  PVLIGNFTRVPEKASTHAENFTLIGNCLSDLHLYIAYVNTDEG.FEEDTATIHIGNIIDISYIPP  193
SCP-2_CPXV_BRI_014   PVLIGNFTRVPEKASTHAENFTLIGNCLSDLHLYIAYVNTDEE.FEEDTATVHIGNKLDINGIPP  193
SCP-2_CPXV_GER_12    PVLIGNFTRVPEKASTHAENFTLIGNCLSDLHLYIAYVNTDEE.FEEDTATIHIGNKIDISGIPP  193
SCP-2_ECTV_MOS_008   PVLIGNFTRVPETASTHAENFTLIGNCLSDLHLYIVYVNTDEG.FEEDTATIHIGNMIDISGIPP  193
SCP-2_SCP-2_COTV168  SYLKTDLVTTPPNPKIHSEDLIIIGSCLKNIDVYFLYLYLDDNKLEKDYTNYHIGNNINTSGILP  186

SCP-2_CPXV_GRI_D12L  NMCATRTIN  202
SCP-2_CPXV_BRI_014   NMCATRTIN  202
SCP-2_CPXV_GER_12    NMCATRTIN  202
SCP-2_ECTV_MOS_008   NTCATRTIN  202
SCP-2_SCP-2_COTV168  QECNIV...  192
```

Figure S6. Sequence alignment of the SCP-2 family members. Cysteine residues are boxed in yellow. Predicted N-linked glycosylation sites are boxed in blue.

SCP-3 Family Alignment

```
                 1        10        20        30        40        50        60
MPXV-SL-161      MYKKLITFLFVIGAVASYSNNEYTPFNKLSVKLYIDGVDNIENSYTDDNNELVLNFKEYTISIIT  65
MPXV_Z96_B8R     MYKKLITFLFVIGAVASYSNNEYTPFNKLSVKLYIDGVDNIENSYTDDNNELVLNFKEYTISIIT  65
TATV_DAH68_190   MYKKLITFLFVIGAIASYSNNEYTPFNKLSVKLYINGVDNIENSYTD.NNELVLIFKEYTVSIIT  64
HSPV184          MYKKLITFLFVIGALASYSNNEYTPFNKLSVKLYIDGVDNIENSYTDDNNELVLNFKEYTISIIT  65
CPXV_BRI_201     MYKKLITFLFVIGAIASYSNNEYTPFNKLSVKLYIDGVDNIENSYTD.NNELVLNFKEYTVSIIT  64
CPXV_GRI_B6R     MYKNLITFLFVIGAVASYSNNEYTPFNKLSVKLYIDGVDNIENSYTDDNNELVLNFKEYTISIIT  65
CPXV_GER91_188   MYKKLITFLFVIGAIASYSNNEYTPFNKLSVKLYIDGVDNIENSYTD.NNELVLIFKEYTVSIIT  64
ECTV_MOS_C3R     MYKKLITFLFVIGAVASYSNNEYTPFNKLSVKLYIDGVDNIENSYTD.NNELVLNFKEYTISIIT  64
VACV_WR_B7R      MYKKLITFLFVIGALASYSNNEYTPFNKLSVKLYIDGVDNIENSYTDDNNELVLNFKEYTISIIT  65

                 70        80        90        100        110        120        130
MPXV-SL-161      ESCDVGFDSIDIDVINDYKIIDMYTIDSSTIQRRGHTCRISTKLSCHYDKYPYIHKYEGDERQYS  130
MPXV_Z96_B8R     ESCDVGFDSIDIDVINDYKIIDMYTIDSSTIQRREHTCRISTKLSCYYDKYPYIHKYEGDERQYS  130
TATV_DAH68_190   ESCDVGFDSIDIDVINDYKIIDMYTIDSSTIQRRGHTCRISTKLSCHYDKHPYIHKYEGDERQYS  129
HSPV184          ESCDVGFDSIDIDVINDYKIIDMYTIDSSTIQRRGHTCRISTKLSCHYDKYPYIHKYDGDERQYS  130
CPXV_BRI_201     ESCDIGFDSIDIDVIDDYKILDMYTIGSSTIQRRGHTCRISTKLSCHYDKHPYIHKYEGDERQYS  129
CPXV_GRI_B6R     ESCDVGFDSIDIDVINDYKIIDMYTIDSSTIQRRGHTCKISTKLSCHYDKYPYIHKYDGDERQYS  130
CPXV_GER91_188   ESCDVGFDSIDIDVINDYKILDMYTIGSSTIQRRGHTCRISTKLSCHYDKHPYIHKYEGDERQYS  129
ECTV_MOS_C3R     ESCDVGFDSIDIDVINDYKILDMYTIDSSTIQRRGHTCKISTKLSCHYDKHPYIHKYEGDERQYS  129
VACV_WR_B7R      ESCDVGFDSIDIDVINDYKIIDMYTIDSSTIQRRGHTCRISTKLSCHYDKYPYIHKYDGDERQYS  130

                 140        150        160        170        180
MPXV-SL-161      ITAEGKCYKGIKYEISMMNDDTLLRKHTLKIGFTYIFDRHGHSNTYYSKYDF  182
MPXV_Z96_B8R     ITAEGKCYKGIKYEISMMNDDTLLRKHTLKIGFTYIFDRHGHSNTYYSKYDF  182
TATV_DAH68_190   ITAEGKCYKGIKYEISMMNDDILLRQHTLKIGSTNIFDSHGHSNTYYSKYDL  181
HSPV184          ITAEGKCYKGIKYEISMINDDTLLRKHTLKIGSTYIFDRHGHSNTYYSKYDF  182
CPXV_BRI_201     ITAEGKCYKGIKYEISMMNDDILLRQHTLKIGSTNIFDRHEHSNIYYPKYDL  181
CPXV_GRI_B6R     ITAEGKCYKGIKYEISMMNDDTLLRKHTLKIGSTYIFDRHGHSNTYYSKYDF  182
CPXV_GER91_188   ITAEGKCYKGIKYEISMMNDDILLRQHTLKIGSTNIFDRHGHSNTYYSKYDL  181
ECTV_MOS_C3R     ITAEGKCYKGIKYEISMMHDDTLLRKHTLKIGSTYIFDRHGHSNTYYSKYDF  181
VACV_WR_B7R      ITAEGKCYKGIKYEISMINDDTLLRKHTLKIGSTYIFDRHGHSNTYYSKYDF  182
```

Figure S7. Sequence alignment of the SCP-3 family members. Cysteine residues are boxed in yellow.
