# Supplementary material for: Structural Conservation and Functional Diversity of the Poxvirus Immune Evasion (PIE) Domain Superfamily
Source: Viruses. 2015 Aug 28;7(9):4873–93. doi: 10.3390/v7092848 (PMC4584292; doi:10.3390/v7092848)
Supplement: Supplementary File 1 [file viruses-07-02848-s001.zip › Nelson_Supplements/Figure_S8_T4.pdf]

T4 Family Alignment

```
                                 1        10        20        30        40        50
MPXV_Z96_B10R       ............MRSLIIVLLFPSIIYSMSIRRCEKTEEETWGLKIGLCIIAKD.FYPERTDCSV   52
CMLV_M96_M-T4-like  ............MRSLIIFLLFPSIIYSMVIRRCEKMEEETWKLKIGMCIQAKD.FYSKRTDCSV   52
TATV_DAH68_195      ............MRSLIIFLLFPSIIYSMVIRRCEKMEEETWKLKIGMCIQAKD.FYSKRTDCSV   52
CPXV_GRI_B8R        ............MRSLIIVLLFPSIIYSMSIRRCEKTEEETWGLKIGLCIIAKD.FYPERTDCSV   52
CPXV_BRI_203        ............MRSLVIVLLFPSIIYSMVIRRCEKMEEETWKLKIGMCIQAKD.FYSKRTDCSV   52
CPXV_GER91_191      ............MRSLIIFLLFPSIIYSMVIRRCEKMEEETWKLKIGMCIQAKD.FYSARTDCSV   52
YKV002c             ..............MFWLLLLLLPLSDSMVIRRCDDKDSSVWELDIGICVKVKKGFISKRTECSV   51
YKV185              ..............MFWLLLLLLPLSDSMVIRRCDDKDSSVWELDIGICVKVKKGFISKRTECSV   51
MYXV_LAU_m004L      MKMYSRLSRVGTMESFVLLLAFLSLVGGYVIDPCTSQERSTWHVSIKLCIYV.QEHKVSANGCRL   64
MYXV_LAU_M-T4       MKMYSRLSRVGTMESFVLLLAFLSLVGGYVIDPCTSQERSTWHVSIKLCIYV.QEHKVSANGCRL   64
DPXV_W83_004        ......MDN.TLLLLLLNFIGLIHYSNSIAIQRCNEDEETTWEIEVGLCITTIDDFKASNTGCNM   58
DPXV_W83_166        ......MDN.TLLLLLLNFIGLIHYSNSIAIQRCNEDEETTWEIEVGLCITTIDDFKASNTGCNM   58
LSDV_2490_003       ......MSSLTLFIFFLQLFIFTSSVSGISIKRCTEEENNTWEIEVGLCIQT.ENFRAIKTGCYK   58
LSDV_2490_154       ......MSSLTLFIFFLQLFIFTSSVSGISIKRCTEEENNTWEIEVGLCIQT.ENFRAIKTGCYK   58
SPPV_A              ......MSSLTFFIFCLQLFIFTSTVSGISIKRCTEEENNTWEIEVGLCIQT.ENFRAIKTGCYK   58
SPPV_A_129          ......MSSLTFFIFCLQLFIFTSTVSGISIKRCTEEENNTWEIEVGLCIQT.ENFRAIKTGCYK   58
GTPV_G20_002        ......MSSLTLFIFFLQLFIFTSSVSGISIKRCTEEENNTWEIEVGLCIQT.DNFRAIKTGCYK   58
GTPV_G20_130        ......MSSLTLFIFFLQLFIFTSSVSGISIKRCTEEENNTWEIEVGLCIQT.DNFRAIKTGCYK   58
GTP_PEL_002         ......MSSLTLFIFFLQLFIFTSSVSGISIKRCTEEENNTWEIEVGLCIQT.DNFRAIKTGCYK   58
GTP_PEL_146         ......MSSLTLFIFFLQLFIFTSSVSGISIKRCTEEENNTWEIEVGLCIQT.DNFRAIKTGCYK   58

                              60        70        80        90       100       110
MPXV_Z96_B10R       HRPTASGGLITEGNGFRVVIYDQCT...EPHDFIITDTQQTRLGS.SHTYIKFSNMNTGVPSSIP  113
CMLV_M96_M-T4-like  HRSDVGGGLITEGNGYRVVVHDQCE...EPNPFIIATTKQTHFGV.THSYIEFSNSNTGVPENIP  113
TATV_DAH68_195      HRSGVGGGLITEGNGYRVVVHDQCE...EPNPFIIATTKQTHFGV.THSYIEFSNSNTGVPENIP  113
CPXV_GRI_B8R        HRPTASGGLITEGNGFRVVIHDQCT...EPHDFIITDTQQTRLGS.SHVYIKFSNMNTGAPSSIP  113
CPXV_BRI_203        HRPDVGGGLITEGNGYRVVVHDQCE...EPNPFIIATTKQTHFGV.THSYIEFSNSNTGAPENIP  113
CPXV_GER91_191      HRPDVGGGLITEGNGFRVVVHDQCE...EPNPFIIATTKQTHFGV.THSYIEFSNSNTGAPENIP  113
YKV002c             YRPDIGGGLITKSNGIKVVVHDKCV...EPGDFIITTTKQAHFGI.TKTYIKLSNHNIQSPENIS  112
YKV185              YRPDIGGGLITKSNGIKVVVHDKCV...EPGDFIITTTKQAHFGI.TKTYIKLSNHNIQSPENIS  112
MYXV_LAU_m004L      EQ..GPGGFIATGNGFKIFAHDECSHNEHS..FLLTDVREAVYASGHGMYVEISG.NVPYLDSVH  124
MYXV_LAU_M-T4       EQ..GPGGFIATGNGFKIFAHDECSHNEHS..FLLTDVREAVYASGHGMYVEISG.NVPYLDSVH  124
DPXV_W83_004        VY..GPGGLIKEGNGFRIFIHDECD.DTNTNEFIVKNVDEAVYGLGRYVYTEMYTRNLSHIELAP  120
DPXV_W83_166        VY..GPGGLIKEGNGFRIFIHDECD.DTNTNEFIVKNVDEAVYGLGRYVYTEMYTRNLSHIELAP  120
LSDV_2490_003       IQ..GPGGLLTEGNGFKIFAHDDCSKEETQNNFILDSVNEAVYALGKYVYMEISTSNITTLYSLP  121
LSDV_2490_154       IQ..GPGGLLTEGNGFKIFAHDDCSKEETQNNFILDSVNEAVYALGKYVYMEISTSNITTLYSLP  121
SPPV_A              IQ..GPGGLLTEGNGFKIFAHDDCSKEKTQNNFILDSVNEAVYALGKYVYMEISTSNITTLNSLP  121
SPPV_A_129          IQ..GPGGLLTEGNGFKIFAHDDCSKEKTQNNFILDSVNEAVYALGKYVYMEISTSNITTLNSLP  121
GTPV_G20_002        IQ..GPGGLLTEGNGFKIFAHDDCSKEETQNNFILDSVNEAVYSLGKYVYMEISTSNITTLYSLP  121
GTPV_G20_130        IQ..GPGGLLTEGNGFKIFAHDDCSKEETQNNFILDSVNEAVYSLGKYVYMEISTSNITTLYSLP  121
GTP_PEL_002         IQ..GPGGLLTEGNGFKIFAHDDCSKEETQNNFILDSVNEAVYSLGKYVYMEISTSNITTLYSLP  121
GTP_PEL_146         IQ..GPGGLLTEGNGFKIFAHDDCSKEETQNNFILDSVNEAVYSLGKYVYMEISTSNITTLYSLP  121

                        120       130       140       150       160       170
MPXV_Z96_B10R       KCSRTLSISVYCDQEAGDIKFEEYT.QESSD.ISIRVKYDSSCIDYLGINQSFMNECIRRIT...  173
CMLV_M96_M-T4-like  DCSKHILISIYCDQEASGLDFHTLKYLESNY.LHITVKYDTSCINHLGVNYSFMNECNRKLSSVY  177
TATV_DAH68_195      DCSKHILISVYCDQEASGLDFHTLKYVESNY.LHITVKYDTSCINHLGVNYSFMNECDRKLSSVY  177
CPXV_GRI_B8R        KCSRTLSISVYCDQEAGDLKFEEYT.QESSD.ISIRVKYDSSCIDYLGINQSFMNECIRRIT...  173
CPXV_BRI_203        DCSKHILISVYCDQEASGLDFHTLKYVESNY.LHITVKYDTSCINHLGVNYSFMNECERKLTSIY  177
CPXV_GER91_191      DCSKHILISVYCDQEASGLDFHTLKYVESNY.LHITVKYDTSCINHLGVNYSFMNECDRKLSSIY  177
YKV002c             ECSKNILVSVYCEQDVSELDFNMLNYVETDN.LNIKVKYDISCIDHIAVNYNFMNKCSKKLSHVH  176
YKV185              ECSKNILVSVYCEQDVSELDFNMLNYVETDN.LNIKVKYDISCIDHIAVNYNFMNKCSKKLSHVH  176
MYXV_LAU_m004L      QCARNMTVAVSCDNPVPNAYG..KNTDKLRPGVAIATLIDTSCVRSHSFAYSVNTLCTERLSGES  187
MYXV_LAU_M-T4       QCARNMTVAVSCDNPVPNAYG..KNTDKLRPGVAIATLIDTSCVRSHSFAYSVNTLCTERLSGES  187
DPXV_W83_004        KCSKKIHVIVSCDEVSSELTYRQEKHEFGESEVEVTIKIDNTCIQHSSSLVQVRSLCEKKLKPVA  185
DPXV_W83_166        KCSKKIHVIVSCDEVSSELTYRQEKHEFGESEVEVTIKIDNTCIQHSSSLVQVRSLCEKKLKPVA  185
LSDV_2490_003       QCAKRISLSISCDQVTTEMKSYVESVSFKDYDLEFVITTDISCVKHVSSSVIVRNECEKKYISTG  186
LSDV_2490_154       QCAKRISLSISCDQVTTEMKSYVESVSFKDYDLEFVITTDISCVKHVSSSVIVRNECEKKYISTG  186
SPPV_A              QCAKRISLSISCDQVTTEMKSYVESVSFKDYDLEFVITTDISCVKHVSSSVIVRNECEKKYISTG  186
SPPV_A_129          QCAKRISLSISCDQVTTEMKSYVESVSFKDYDLEFVITTDISCVKHVSSSVIVRNECEKKYISTG  186
GTPV_G20_002        QCAKRISLSISCDQVTTEMKSYVESVSFKDYDLEFVITTDISCVKHVSSSVIVRNECEKKYISTG  186
GTPV_G20_130        QCAKRISLSISCDQVTTEMKSYVESVSFKDYDLEFVITTDISCVKHVSSSVIVRNECEKKYISTG  186
GTP_PEL_002         QCAKRISLSISCDQVTTEMKSYVESVSFKDYDLEFVITTDISCVKHVSSSVIVRNECEKKYISTG  186
GTP_PEL_146         QCAKRISLSISCDQVTTEMKSYVESVSFKDYDLEFVITTDISCVKHVSSSVIVRNECEKKYISTG  186

                                 180       190       200       210       220
MPXV_Z96_B10R       .......TWDRESCVRIDTQTINKYLKSCTNTKFDR...NVYKRYILKSKALHAKTEL.  221
CMLV_M96_M-T4-like  .......ESDTLTCGAKDIQTRDKYLKTCTNTKFDR...SVYKTHMQKSKILHVKTEL.  225
TATV_DAH68_195      .......ERDTLTCGAKDIQTRDKYLKTCTNTKFDR...SVYKTHMQKSKILHVKTEL.  225
CPXV_GRI_B8R        .......TWDRESCVGIDTQTINKYLKSCTNTKFDR...SVYKRYILKSKALHAKTEL.  221
CPXV_BRI_203        .......ETDTLTCGAKDIQTRDKYLKTCTNTKFDR...SVYKTHMQKSKILHVKTEL.  225
CPXV_GER91_191      .......ESDTLTCGAKDIQTRDKYLKTCTNTKFDR...SVYKTHMQKSKILHVKTEL.  225
YKV002c             .......DRDILNCGAPDVSTRDKYLKTCSNDEFNR...IVYKKYIMKSKNLHSKTEL.  224
YKV185              .......DRDILNCGAPDVSTRDKYLKTCSNDEFNR...IVYKKYIMKSKNLHSKTEL.  224
MYXV_LAU_m004L      ........CEQLDCHMVKGSQHEHYLQTCDRNVPDRRTFKAYKPHQRPYVAKVLRDEL.  237
MYXV_LAU_M-T4       ........CEQLDCHMVKGSQHEHYLQTCDRNVPDRRTFKAYKPHQRPYVAKVLRDEL.  237
DPXV_W83_004        SKYIFSSNIGKLTCGFGTVENHNKYLKPCHHGSFDR...SKYNKHQHEHIKKIFYYDEL  241
DPXV_W83_166        SKYIFSSNIGKLTCGFGTVENHNKYLKPCHHGSFDR...SKYNKHQHEHIKKIFYYDEL  241
LSDV_2490_003       .KKIFGFNN.KIDCSAVKFSEHVNYLKTCSVGKFDR...KKYYEHQHNYIKKIFHHNEL  240
LSDV_2490_154       .KKIFGFNN.KIDCSAVKFSEHVNYLKTCSVGKFDR...KKYYEHQHNYIKKIFHHNEL  240
SPPV_A              .KKIFGFNN.KIDCSAVKFSEHVNYLKTCSVGKFDR...KKYYEHQHNYIKKIFHHNEL  240
SPPV_A_129          .KKIFGFNN.KIDCSAVKFSEHVNYLKTCSVGKFDR...KKYYEHQHNYIKKIFHHNEL  240
GTPV_G20_002        .KKIFGFNN.KIDCSAVKFSEHVNYLKTCSVGKFDR...KKYYEHQHNYIKKIFHHNEL  240
GTPV_G20_130        .KKIFGFNN.KIDCSAVKFSEHVNYLKTCSVGKFDR...KKYYEHQHNYIKKIFHHNEL  240
GTP_PEL_002         .KKIFGFNN.KIDCSAVKFSEHVNYLKTCSVGKFDR...KKYYEHQHNYIKKIFHHNEL  240
GTP_PEL_146         .KKIFGFNN.KIDCSAVKFSEHVNYLKTCSVGKFDR...KKYYEHQHNYIKKIFHHNEL  240
```

Figure S8. Sequence alignment of the T4 family members. Cysteine residues are boxed in yellow. Predicted N-linked glycosylation sites are boxed in blue.
