# Supplementary material for: Structural Conservation and Functional Diversity of the Poxvirus Immune Evasion (PIE) Domain Superfamily
Source: Viruses. 2015 Aug 28;7(9):4873–93. doi: 10.3390/v7092848 (PMC4584292; doi:10.3390/v7092848)
Supplement: Supplementary File 1 [file viruses-07-02848-s001.zip › Nelson_Supplements/Figure_S9_M2.pdf]

# M2 family Alignment

```
                  1        10        20        30        40        50
VARV_SLN68_22     ......MVYKLVLLFCIASLGYSVE..YKNTICPPRQD.....YRYWYFAAELTIGVNYDINSTI   52
CMLV_M96_029      ......MVYKLVLLFCIASLGCSVE..YKNTICPPRQD.....YRYWYFAAELTIGVNYDINSTI   52
VARV_CNG70_022    ......MVYKLVLLFCIASLGYSVE..YKNTICPPRQD.....YRYWYFAAELTIGVNYDINSTI   52
VARV_IND64_022    ......MVYKLVLLFCIASLGYSVE..YKNTICPPRQD.....YRYWYFAAELTIGVNYDINSTI   52
VARV_GUI69_22     ......MVYKLVLLFCIASLGYSVE..YKNTICPPRQD.....YRYWYFAAELTIGVNYDINSTI   52
TATV_DAH68_033    ......MVYKLVLLFCIASLGYSVE..YKNTICPPRQD.....YRYWYFAAELTIGVNYDINSTI   52
CPXV_GER91_038    ......MVYKLVLLFCAVSLGYSAE..YKNTICPPRQD.....YRYWYFAAELTIGVNYDINSTI   52
HSPV_034          ......MVYKLVLLFCIASLGYSVE..YKNTICPPRQD.....YRYWYFAAELTIGVNYDINSTI   52
CPXV_BRI_040      ......MVYKLVLLLCVVSLGYSAE..YKNTICPPRQD.....YRYWYFAAELTIGVNYDINSTI   52
MPXV-SL-21        ......MVYKLVLLFCIASLGYSVE..YKNTICPHRQD.....YRYWYFVAELTIGVNYDINSTI   52
MPXV_Z96_O2L      ......MVYKLVLLFCIASLGYSVE..YKNTICPPRQD.....YRYWYFVAELTIGVNYDINSTI   52
VACV_WR_M2L       ......MVYKLVLLFCIASLGYSVE..YKNTICPPRQD.....YRYWYFAAELTIGVNYDINSTI   52
VARV_BRZ66_26     ......MVYKLVLLFCIASLGYSVE..YKNTICPPRQD.....YRYWYFAAELTIGVNYDINSTI   52
VARV_GAR_Q2L      ......MVYKLVLLFCIASLGYSVE..YKNTICPPRQD.....YRYWYFAAELTIGVNYDINSTI   52
YYMTV_YLD_9L      MEGVKKLFNFLILFFFVSTTLSYILKQDH....KDFGKCVSKKYRYWNLAAVLTLGLTYTIP...   58
COTV028           ......MFSIIYLLFCVISLCYSSD..YQDMQCPTRSDNN...YRYWYFATELTIGVNYNIK.TI   53
YKV_170           .....MNKNILPLLMCILSVCYCVETSYKPLQCQVRDP......RYWYLAAELTIGLNYNITSTI   54
MYXV_LAU_m154L    .....MARYIIIVLACLVATST..........CATYPK......KYWHLAAELTIGLNRYVE.TV   43
DPXV_W83_017      ...MAKLFYFILISICISSCLSSPLSNRNGKLSSSLGKCNSHRYRFWNLASSLVIGLTYPIK...   59

                         60        70        80        90        100       110
VARV_SLN68_22     IGECYMSESYIDRNANIVLTGYGLEINMTIMDTDQR.FVAAAEGVGKDNKL.SVMLFTTQR....  111
CMLV_M96_029      IGECHMSESYIDRNANIVLTGYGLEINMTIMDTDQR.FVAAAEGVGKDNKL.SVMLFTTQR....  111
VARV_CNG70_022    IGECYMSESYIDRNANIVLTGYGLEINMTIMDTDQR.FVAAAEGVGKDNKL.SVMLFTTQR....  111
VARV_IND64_022    IGECYMSESYIDRNANIVLTGYGLEINMTIMDTDQR.FVAAAEGVGKDNKL.SVMLFTTQR....  111
VARV_GUI69_22     IGECYMSESYIDRNANIVLTGYGLEINMTIMDTDQR.FVAAAEGVGKDNKL.SVMLFTTQR....  111
TATV_DAH68_033    IGECHMSESYIDRNANIMLTGYGLEINMTIMDTDQR.FVAAAEGVGKDNKL.SVMLFTTQR....  111
CPXV_GER91_038    MGECHMSESYIDRNANIVLTGYGLEINMTIMDTDQR.FVAAAEGVGKDNKL.SVLLFTTQR....  111
HSPV_034          IGECHMSESYIDRNANIVLTGYGLEINMTIMDTDQR.FVAAAEGVGKDNKL.SVLLFTTQR....  111
CPXV_BRI_040      MGECHMSESYIDRNANIVLTGYGLEINMTIMDTDQR.FVAAAEGVGKDNKL.SVLLFTTQR....  111
MPXV-SL-21        IGECHMSESYIDRNANIVLTGYGLKVNMTIMDTDQR.FVAAAEGVGKDNKL.SVLLFTTQR....  111
MPXV_Z96_O2L      IGECHMSESYIDRNANIVLTGYGLKINMTIMDTDQR.FVAAAEGVGKDNKL.SVLLFTTQR....  111
VACV_WR_M2L       IGECHMSESYIDRNANIVLTGYGLEINMTIMDTDQR.FVAAAEGVGKDNKL.SVLLFTTQR....  111
VARV_BRZ66_26     IGECYMSESYIDRNANIVLTGYGLEINMTIMDTDQR.FVAAAEGVGKDNKL.SVMLFTTQR....  111
VARV_GAR_Q2L      IGECYMSESYIDRNANIVLTGYGLEINMTIMDTDQR.FVAAAEGVGKDNKL.SVMLFTTQR....  111
YYMTV_YLD_9L      ..EKEQCFAHIHLDTTL.VIGYGLSIEIEITNKIDGQVVSVVEGLYNNNTI.ILLLFIANDRSDY  119
COTV028           VGECRITEGYVDRNANIVLSGYGLVINMTLTDTDQR.FVAAAEGVGKDNKL.SVLLFTSQR....  112
YKV_170           QGECHMEYNYIDRNARITLTGYGLRITMDIINTDQR.FVGAAEGIGENNKL.SVLLFTTEK....  113
MYXV_LAU_m154L    MGECHMKERYDHKTSTLILTGYGLMINITITNVVQR.FVAASAGAGDGNKL.SIMLFTTHP....  102
DPXV_W83_017      ..EQEECKIDIISNSHISITGYGINVDFEFTTEITKPLVASAEGFVN.NTL.VILIFMADDRSSN  120

                        120       130       140       150       160       170
VARV_SLN68_22     LDKVHHNISVIITCMEMNCGTTKY.NSDLPESIYHKSSCDITINGSCVTCVDLETDPTKINPHYL  175
CMLV_M96_029      LDKVHHNTSVIITCMEMNCGTTKY.DSDLPESIHHKSSCDITINGSCVTCVNLETDPTKINPHYL  175
VARV_CNG70_022    LDKVHHNISVIITCMEMNCGTTKY.NSDLPESIHHKSSCDITINGSCVTCVNLETDPTKINPHYL  175
VARV_IND64_022    LDKVHHNISVIITCMEMNCGTTKY.NSDLPESIHHKSSCDITINGSCVTCVNLETDPTKINPHYL  175
VARV_GUI69_22     LDKVHHNISVIITCMEMNCGTTKY.NSDLPESIYHKSSCDITINGSCVTCVDLETDPTKINPHYL  175
TATV_DAH68_033    LDKVHHNISVIITCMEMNCGTTKY.DSELPESIHHKSSCDITINGSCVTCVNLETDPTKINPHYL  175
CPXV_GER91_038    LDKVHHNISVTITCMEMNCGTTKY.DSDLPESIHHKSSCDITINGSCVTCVNLETDPTKINPHYI  175
HSPV_034          LDKVHHNISVTITCMEMNCGTTKY.DSDLPESIHKSSSCDITINGSCVTCVNLETDPTKINPHYL  175
CPXV_BRI_040      LDKVHHNISVTITCMEMNCGTTKY.DSDLPESIHHKSSCDITINGSCVTCVNLETDPTKINPHYL  175
MPXV-SL-21        LDKVHHNISVTITCMEMNCGTTKY.NSDLPESIHKSSSCDITINGSCVTCVNLETDPTKINPHYL  175
MPXV_Z96_O2L      LDKVHHNISVTITCMEMNCGTTKY.NSDLPESIHKSSSCDITINGSCVTCVNLETDPTKINPHYL  175
VACV_WR_M2L       LDKVHHNISVTITCMEMNCGTTKY.DSDLPESIHKSSSCDITINGSCVTCVNLETDPTKINPHYL  175
VARV_BRZ66_26     LDKVHHNISVIITCMEMNCGTTKY.NSDLPESIHHKSSCDITINGSCVTCVDLETDPTKINPHYL  175
VARV_GAR_Q2L      LDKVHHNISVIITCMEMNCGTTKY.NSDLPESIHHKSSCDITINGSCVTCVDLETDPTKINPHYL  175
YYMTV_YLD_9L      ENSVIPNTKISVTCTDIDCDNNPT.RQVLNGKVSKN...ELIIFGSCLTCVLLDTYPNSIKGFLP  180
COTV028           LDNKQHNISLTITCMEMNCGTTKF.NGSLPESMHHGSSCDITINGSCVTCVNLQMDPITINHNFK  176
YKV_170           LYKISHNISLTITCLEYNCGNTWYKNNKLSEAIHHRSVCDITINGSCVRCVSIFMDPLNTHSFYY  178
MYXV_LAU_m154L    LTK.YSDIYLTITCLEPEGDVGNY.GNQLPDSLHHNKDVSITILGSCVTCVNLETNPIKVNPHFT  165
DPXV_W83_017      ANNSIPDTKVTITCLDVDCDIVSK.RQNIDNLSTKN...ELIIVGSCVTCVDLNVYPNSLYGFKS  181

                      180       190       200       210       220
VARV_SLN68_22     HPKDKYLYHNSKYGMRGSYGVTFIDELNQCLLDIKELSYDICYRE.....  220
CMLV_M96_029      HPKDKYLYHNSKYGMRGSYGVTLIDELNQCLLDIKELSYDICYRE.....  220
VARV_CNG70_022    HPKDKYLYHNSKYGMRGSYGVTFIDELNQCLLDIKELSYDICYRE.....  220
VARV_IND64_022    HPKDKYLYHNSKYGMRGSYGVTFIDELNQCLLDIKELSYDICYRE.....  220
VARV_GUI69_22     HPKDKYLYHNSKYGMRGSYGVTFIDELNQCLLDIKELSYDICYRE.....  220
TATV_DAH68_033    HPKDKYLYHNSKYGMRGSYGVTFIDELNQCLLDIKELSYDICYRE.....  220
CPXV_GER91_038    HPKDKYLYHNSEYGMRGSYGMTFIDELNQCFLDIKEVSYDICYRE.....  220
HSPV_034          HPKDKYLYHNSEYSMRGSYGVTFIDELNQCLLDIKELSYDICYRE.....  220
CPXV_BRI_040      HPKDKYLYRNSEYGMRGSYGVTFMDELNQCFLDIKEVSYDICYRE.....  220
MPXV-SL-21        HPKNKYLYHNSEYSMRGSYGVTFIDELNQCLLDIKELSYDICYRE.....  220
MPXV_Z96_O2L      HPKNKYLYHNSEYSMRGSYGVTFIDELNQCLLDIKELSYDICYRE.....  220
VACV_WR_M2L       HPKDKYLYHNSEYSMRGSYGVTFIDELNQCLLDIKELSYDICYRE.....  220
VARV_BRZ66_26     HPKDKYLYHNSKYGMRGSYGVTFIDELNQCLLDIKELSYDICYRE.....  220
VARV_GAR_Q2L      HPKDKYLYHNSKYGMRGSYGVTFIDELNQCLLDIKELSYDICYRE.....  220
YYMTV_YLD_9L      PSGIVAKPYSKGVNEDNGYL.WYRHKKDSCNVDFLNTSYSICSKIRR...  226
COTV028           YPSDKYLYKNHNYDGQGSYGLTFKDELNQCFLVIKEVSYDMCYR......  220
YKV_170           QG..KYITNNYEYNQRGTYGVAFGDDTIACLTNIDKIRYDICYRQ.....  221
MYXV_LAU_m154L    HPISMFVYDNKED.VRGSYGVTFEDELNVCFLDIKKVSYDLCYRQTRYLI  214
DPXV_W83_017      ANSIIARPYSGTSG.GYTWR.NLNTSKRQCDVDTEKIDYYVCDKKSN...  226
```

**Figure S9.** Sequence alignment of the M2 family members. Cysteine residues are boxed in yellow. Predicted N-linked glycosylation sites are boxed in blue.
